# The global burden of tuberculosis: results from the Global Burden of Disease Study 2015

**DOI:** 10.1016/S1473-3099(17)30703-X

**Published:** 2018-03

**Authors:** GBD Tuberculosis Collaborators, Hmwe H Kyu, Emilie R Maddison, Nathaniel J Henry, John Everett Mumford, Ryan Barber, Chloe Shields, Jonathan C Brown, Grant Nguyen, Austin Carter, Timothy M Wolock, Haidong Wang, Patrick Y Liu, Marissa Reitsma, Jennifer M Ross, Amanuel Alemu Abajobir, Kalkidan Hassen Abate, Kaja Abbas, Mubarek Abera, Semaw Ferede Abera, Habtamu Abera Hareri, Muktar Ahmed, Kefyalew Addis Alene, Nelson Alvis-Guzman, Joshua Amo-Adjei, Jason Andrews, Hossein Ansari, Carl Abelardo Antonio, Palwasha Anwari, Hamid Asayesh, Tesfay Mehari Atey, Sachin Atre, Aleksandra Barac, Justin Beardsley, Neeraj Bedi, Isabela Bensenor, Addisu Shunu Beyene, Zahid Ahmad Butt, Pere-Joan Cardona, Devasahayam Christopher, Lalit Dandona, Rakhi Dandona, Kebede Deribe, Amare Deribew, Rebecca Ehrenkranz, Maysaa El Sayed Zaki, Aman Endries, Tesfaye R Feyissa, Florian Fischer, Ruoyan Gai, Alberto L Garcia-Basteiro, Tsegaye Tewelde Gebrehiwot, Hailay Gesesew, Belete Getahun, Philimon Gona, Amador Goodridge, Harish Gugnani, Hassan Haghparast-Bidgoli, Gessessew Bugssa Hailu, Hamid Yimam Hassen, Esayas Hilawe, Nobuyuki Horita, Kathryn H Jacobsen, Jost B Jonas, Amir Kasaeian, Muktar Sano Kedir, Laura Kemmer, Yousef Khader, Ejaz Khan, Young-Ho Khang, Abdullah T Khoja, Yun Jin Kim, Parvaiz Koul, Ai Koyanagi, Kristopher J Krohn, G Anil Kumar, Michael Kutz, Rakesh Lodha, Hassan Magdy And El Razek, Reza Majdzadeh, Tsegahun Manyazewal, Ziad Memish, Walter Mendoza, Haftay Berhane Mezgebe, Shafiu Mohammed, Felix Akpojene Ogbo, In-Hwan Oh, Eyal Oren, Aaron Osgood-Zimmerman, David Pereira, Dietrich Plass, Farshad Pourmalek, Mostafa Qorbani, Anwar Rafay, Mahfuzar Rahman, Rajesh Kumar Rai, Puja C Rao, Sarah E Ray, Robert Reiner, Nickolas Reinig, Saeid Safiri, Joshua A Salomon, Logan Sandar, Benn Sartorius, Morteza Shamsizadeh, Muki Shey, Desalegn Markos Shifti, Hirbo Shore, Jasvinder Singh, Chandrashekhar T Sreeramareddy, Soumya Swaminathan, Scott J Swartz, Fentaw Tadese, Bemnet Amare Tedla, Balewgizie Sileshi Tegegne, Belay Tessema, Roman Topor-Madry, Kingsley Nnanna Ukwaja, Olalekan A. Uthman, Vasiliy Vlassov, Stein Emil Vollset, Tolassa Wakayo, Solomon Weldegebreal, Ronny Westerman, Abdulhalik Workicho, Naohiro Yonemoto, Seok-Jun Yoon, Marcel Yotebieng, Mohsen Naghavi, Simon I Hay, Theo Vos, Christopher JL Murray

## Abstract

**Background:**

An understanding of the trends in tuberculosis incidence, prevalence, and mortality is crucial to tracking of the success of tuberculosis control programmes and identification of remaining challenges. We assessed trends in the fatal and non-fatal burden of tuberculosis over the past 25 years for 195 countries and territories.

**Methods:**

We analysed 10 691 site-years of vital registration data, 768 site-years of verbal autopsy data, and 361 site-years of mortality surveillance data using the Cause of Death Ensemble model to estimate tuberculosis mortality rates. We analysed all available age-specific and sex-specific data sources, including annual case notifications, prevalence surveys, and estimated cause-specific mortality, to generate internally consistent estimates of incidence, prevalence, and mortality using DisMod-MR 2.1, a Bayesian meta-regression tool. We assessed how observed tuberculosis incidence, prevalence, and mortality differed from expected trends as predicted by the Socio-demographic Index (SDI), a composite indicator based on income per capita, average years of schooling, and total fertility rate. We also estimated tuberculosis mortality and disability-adjusted life-years attributable to the independent effects of risk factors including smoking, alcohol use, and diabetes.

**Findings:**

Globally, in 2015, the number of tuberculosis incident cases (including new and relapse cases) was 10·2 million (95% uncertainty interval 9·2 million to 11·5 million), the number of prevalent cases was 10·1 million (9·2 million to 11·1 million), and the number of deaths was 1·3 million (1·1 million to 1·6 million). Among individuals who were HIV negative, the number of incident cases was 8·8 million (8·0 million to 9·9 million), the number of prevalent cases was 8·9 million (8·1 million to 9·7 million), and the number of deaths was 1·1 million (0·9 million to 1·4 million). Annualised rates of change from 2005 to 2015 showed a faster decline in mortality (−4·1% [−5·0 to −3·4]) than in incidence (−1·6% [−1·9 to −1·2]) and prevalence (−0·7% [−1·0 to −0·5]) among HIV-negative individuals. The SDI was inversely associated with HIV-negative mortality rates but did not show a clear gradient for incidence and prevalence. Most of Asia, eastern Europe, and sub-Saharan Africa had higher rates of HIV-negative tuberculosis burden than expected given their SDI. Alcohol use accounted for 11·4% (9·3–13·0) of global tuberculosis deaths among HIV-negative individuals in 2015, diabetes accounted for 10·6% (6·8–14·8), and smoking accounted for 7·8% (3·8–12·0).

**Interpretation:**

Despite a concerted global effort to reduce the burden of tuberculosis, it still causes a large disease burden globally. Strengthening of health systems for early detection of tuberculosis and improvement of the quality of tuberculosis care, including prompt and accurate diagnosis, early initiation of treatment, and regular follow-up, are priorities. Countries with higher than expected tuberculosis rates for their level of sociodemographic development should investigate the reasons for lagging behind and take remedial action. Efforts to prevent smoking, alcohol use, and diabetes could also substantially reduce the burden of tuberculosis.

**Funding:**

Bill & Melinda Gates Foundation.

## Introduction

Tuberculosis kills more than 1 million people every year, most of them in low-income and middle-income countries.^1^, ^2^, ^3^ An understanding of the trends in tuberculosis incidence, prevalence, and mortality is crucial to track the success of tuberculosis control programmes and to identify remaining intervention challenges for tuberculosis care and prevention. Rigorous evaluation of these trends is, however, challenging.^1^ The primary data sources used to estimate the epidemiological burden of tuberculosis, including annual case notifications, prevalence surveys, and cause of death data, have various shortcomings.^1^, ^4^, ^5^ Also, their availability differs across regions and time periods.

In countries where tuberculosis is endemic, health and surveillance systems are usually weak, with underdiagnosis and under-reporting common.^5^ Prevalence surveys are designed to provide unbiased measures of tuberculosis prevalence, but low response rates and contamination of tuberculosis specimens affect the quality of these surveys.^4^, ^6^ The validity of imputation methods to correct for low response rates in prevalence surveys has been questioned; even in countries with a more than 90% response rate, imputation can increase the prevalence of smear-positive tuberculosis by 6–13%.^4^ The need for large sample sizes makes prevalence surveys expensive and hence they are carried out only intermittently or not at all by countries with a substantial burden. In many tuberculosis-endemic countries where reliable vital registration systems are unavailable, verbal autopsy is commonly used to measure cause-specific mortality. Verbal autopsy studies are prone to misclassification errors as they have to rely on information recalled by family members of the deceased.^7^ Given the imperfections in data sources, we propose that statistical triangulation of multiple data sources could provide a more robust assessment of tuberculosis epidemiology than has been done so far.^1^

**Research in context**

**Evidence before this study**

Tuberculosis is a leading cause of morbidity and mortality, especially in low-income and middle-income countries. The global burden of tuberculosis has been estimated by several groups, including the WHO Global TB Programme and the Global Burden of Diseases, Injuries, and Risk Factors Study 2013. However, the contribution of potentially modifiable risk factors to tuberculosis burden and how the burden changes as countries progress through the epidemiological transition have not been well characterised. We searched PubMed with the search terms “tuberculosis” AND (“burden” OR “estimates”) AND “trend”, with no language restrictions, for articles published up to Nov 21, 2017, which produced 17 studies that provided population-wide tuberculosis burden time trends (incidence, prevalence, or deaths), of which ten were at the country level, six were at the subnational level, and one was at the regional and country level. Of all studies, the most recent period assessed was 1999–2013 in Lebanon. None of these studies assessed the tuberculosis burden attributable to risk factors over time or the epidemiological transition.

**Added value of this study**

This study provides a comprehensive assessment of the trends in tuberculosis burden and the burden attributable to risk factors (smoking, alcohol use, and diabetes). Moreover, it includes analysis of the relationship between tuberculosis burden and Socio-demographic Index (a composite indicator based on income, education, and fertility developed for the Global Burden of Diseases, Injuries, and Risk Factors Study 2015) to enhance the understanding of a country's tuberculosis status in the context of its sociodemographic position. It identifies key areas for prioritisation of resources and areas for further research and interventions.

**Implications of all the available evidence**

Whereas progress is being made in reduction of tuberculosis mortality, tuberculosis is still responsible for an enormous disease burden worldwide. Moreover, incidence is declining more slowly than mortality in many countries. Strengthening of health systems for early detection of tuberculosis and improvements in diagnostics, treatment, and follow-up should therefore be priorities. Countries where the burden of tuberculosis is higher than predicted by their sociodemographic development should work to investigate the reasons for the discrepancy and address them as appropriate. Efforts to prevent smoking, alcohol use, diabetes, and HIV are also likely to substantially reduce the global burden of tuberculosis.

An assessment of the contribution of potentially modifiable risk factors is also a crucial input into tuberculosis control policy. Moreover, an assessment of how incidence, prevalence, and mortality change as countries progress through the epidemiological transition (ie, an epidemiological shift from communicable to non-communicable causes of disease burden related to sociodemographic development)^3^, ^8^, ^9^ can enhance understanding of a country's tuberculosis status in the context of its sociodemographic position. Knowledge of which countries lag behind the sociodemographic development trajectory for these measures can inform both investments in research and subsequent intervention efforts that aim to meet the Sustainable Development Goal to end tuberculosis by 2030.^10^

For the Global Burden of Diseases, Injuries, and Risk Factors Study 2015 (GBD 2015),^3^, ^8^, ^9^ we assessed the levels and trends in the fatal and non-fatal burden of tuberculosis over the past 25 years for 195 countries and territories. We also analysed the relationship between tuberculosis burden and Socio-demographic Index (SDI), a composite indicator based on income, education, and fertility and developed for GBD 2015. We also estimated tuberculosis deaths and disability-adjusted life-years (DALYs) attributable to the independent effects of risk factors including smoking, alcohol use, and diabetes.

## Methods

### Overview

The Global Burden of Disease (GBD) is a systematic, scientific effort to quantify the comparative magnitude of health loss due to diseases, injuries, and risk factors by age, sex, and geography over time. GBD 2015 includes 195 countries and territories, 11 of which (Brazil, China, India, Japan, Kenya, Mexico, Saudi Arabia, South Africa, Sweden, the UK, and the USA) were analysed at the subnational level. The conceptual and analytical framework for GBD, with details of the hierarchy of causes and risk factors, data inputs and processing, and analytical methods, has been published elsewhere.^3^, ^8^, ^9^, ^11^ We summarise the methods used for analysis of the burden of tuberculosis.

### Case definition

Tuberculosis is an infectious disease caused by *Mycobacterium tuberculosis* complex. The case definition includes all forms of tuberculosis, including pulmonary and extrapulmonary tuberculosis, which are bacteriologically confirmed or clinically diagnosed. The International Classification of Diseases (ICD)-10 codes are A10–19.9, B90–90.9, K67.3, K93.0, M49.0, and P37.0, and the ICD-9 codes are 010–19.9, 137–37.9, 138.0–38.9, and 730.4–30.6. For HIV–tuberculosis, the ICD 10 code is B20.0.

### Tuberculosis mortality among HIV-negative individuals

The appendix shows the input data, analytical process, and output from the analysis of tuberculosis mortality among HIV-negative individuals. Input data for this analysis included 10 691 site-years of vital registration data, 768 site-years of verbal autopsy data, and 361 site-years of mortality surveillance data. Country-specific data sources and citations are available online. The assessment and adjustment of vital registration data for completeness have been reported in detail previously.^3^ Vital registration data were adjusted for garbage coding (including ill-defined codes and use of intermediate causes) following GBD algorithms and misclassified HIV deaths (ie, HIV deaths being assigned to other underlying causes of death, such as tuberculosis or diarrhoea because of stigma or misdiagnosis). Country-specific data before and after garbage code redistribution are available in the online data visualisation tool. Verbal autopsy data in countries with high HIV prevalence (using an arbitrary cutoff of 5% age-standardised HIV prevalence) were removed because of a high probability of misclassification, as verbal autopsy studies have a poor ability to distinguish HIV deaths from HIV–tuberculosis deaths (ie, tuberculosis deaths among HIV-positive people).

We used our Cause of Death Ensemble modelling (CODEm) strategy,^2^, ^12^, ^13^, ^14^ which has been widely used to generate global estimates of cause-specific mortality. The CODEm strategy evaluates potential models that apply different functional forms (mixed-effects models and spatiotemporal Gaussian process regression models) to mortality rates or cause fractions with varying combinations of predictive covariates.^2^ These covariates consist of alcohol consumption (litres of pure alcohol per person per year), diabetes (fasting plasma glucose concentration in mmol/L), education (years per person), health system access, lag-distributed income (LDI; gross domestic product per capita that has been smoothed over the preceding 10 years), the proportion of malnutrition (children younger than 5 years of age who are underweight), indoor air pollution prevalence, population density (people per km^2^), smoking prevalence, sociodemographic status, and a summary exposure variable (SEV) scalar. The SEV scalar reflects the exposure to risk factors related to tuberculosis weighted by their relative risk value. The methods used to develop the SEV scalar covariate for GBD 2015 have been described in detail elsewhere.^11^ The ensemble of CODEm models that performed best on out-of-sample predictive validity tests was then selected.

### HIV–tuberculosis mortality

To establish tuberculosis deaths in HIV-positive individuals, we first computed the fraction of HIV–tuberculosis deaths among all tuberculosis deaths using 144 country-years of high-quality vital registration data (appendix). Second, we calculated the proportion of HIV–tuberculosis cases among all tuberculosis cases with an HIV test result as reported in the WHO tuberculosis register. We used a mixed-effects regression on the logit of the proportion of HIV–tuberculosis cases among all tuberculosis cases to predict the proportions of HIV-positive tuberculosis cases for all locations and years, using an adult HIV death rate covariate and country random effects. Third, we assumed that the fraction of HIV–tuberculosis deaths among all tuberculosis deaths in each location and year (D_c,y_) is a function of the prevalence of HIV–tuberculosis among tuberculosis cases (P_c,y_) and that the relative risk (RR) of tuberculosis death among patients with HIV infection and tuberculosis can be generalised over time and between locations:

$$D_{c,y}=\frac{P_{c,y}RR}{P_{c,y}RR+1-P_{c,y}}$$

Solving the equation for RR gives:

$$RR=\frac{D_{c,y}P_{c,y}-D_{c,y}}{D_{c,y}P_{c,y}-P_{c,y}}$$

We took the RR from each location and year for which we had data for the fraction of HIV–tuberculosis deaths among all tuberculosis deaths to estimate a median RR. We then applied that median RR to the predicted proportions of HIV–tuberculosis cases among all tuberculosis cases to estimate the fraction of HIV–tuberculosis deaths among all tuberculosis deaths for all locations and years. Next, we calculated location-year-specific HIV–tuberculosis deaths (Deaths_HIV–TBc,y_) using the following equation:

$$Deaths_{HIV-TB_{c,y}}=\frac{D_{c,y}}{1-D_{c,y}}Deaths_{TB_{c,y}}$$

where Deaths_TBc,y_ is location-year-specific deaths from the CODEm tuberculosis HIV-negative model. Finally, we applied the age-sex pattern of the HIV mortality estimates to these HIV–tuberculosis deaths to generate HIV–tuberculosis deaths for all locations and years by age and sex. Since the HIV–tuberculosis deaths were estimated on the basis of the fraction of HIV–tuberculosis deaths among all tuberculosis deaths, the total number of HIV–tuberculosis deaths could exceed the total number of HIV deaths in some locations. To avoid this occurrence, we applied a cap of 45% to the fraction of HIV–tuberculosis deaths among HIV deaths on the basis of the largest fraction reported in a review by Cox and colleagues^15^ and a systematic review and meta-analysis by Ford and colleagues.^16^

### Non-fatal tuberculosis and HIV–tuberculosis

We used all available cause of death data, case notifications, and data from prevalence surveys to produce consistent estimates of tuberculosis epidemiology (appendix). From these inputs, we calculated priors (expected values) on excess mortality and remission to guide the model. We used DisMod-MR 2.1,^17^ the GBD Bayesian meta-regression tool that adjusts for differences in methods between data sources and imposes consistency between data for different parameters. We explain in detail below the preparation of each of these data sources and the modelling in DisMod-MR 2.1.

We used the age-specific and sex-specific notifications (from WHO and our network of collaborators) in our modelling of tuberculosis incidence. Our definition of incident cases include new and relapse cases diagnosed within a given calendar year. If the notification data represented new and relapse cases combined, we used the data as they were. If cases were broken down by case type (new pulmonary smear-positive, new pulmonary smear-negative, new extrapulmonary, and relapse), we summed them to represent all forms of tuberculosis. Smear-positive notification data were missing for at least one age group for at least 1 year in 41 countries. These countries were from sub-Saharan Africa, Asia, Latin America and the Caribbean, north Africa and the Middle East, eastern, central, and western Europe, and high-income north America. Smear-negative and extrapulmonary tuberculosis data were missing for at least one age group for at least 1 year in almost all countries. We imputed missing age groups for three forms of tuberculosis notifications (pulmonary smear-positive, pulmonary smear-negative, and extrapulmonary). We increased smear-positive age-specific notifications by the proportions of smear-unknown and relapsed cases that were only reported at the country-year level. Some countries reported pulmonary smear-positive cases only for selected years (eg, 67 countries in 2006 and 33 in 2012). Most of these countries were from sub-Saharan Africa and southeast Asia). We predicted missing smear-negative and extrapulmonary cases from adjusted smear-positive cases using a seemingly unrelated regression approach.^18^ We then added all three types of notifications. We categorised countries on the basis of WHO's estimates of country-year-specific case detection rates (CDRs) into ten bins using a 5 year moving average. We assumed all high-income countries to be in the highest decile of CDR. For all other countries, we used covariates for their CDR decile as an initial guide for how much notifications need to be increased in DisMod-MR 2.1 to reflect the incidence of all tuberculosis. We then generated a final incidence estimate that is consistent with prevalence data and cause-specific mortality estimates using Bayesian meta-regression. We included SEV as a location-level covariate to help inform variation over year and geography, with priors that at higher SEV values, incidence increases.

We estimate point prevalence for tuberculosis. Point prevalent cases represent people in the population who at any point during a given calendar year have active tuberculosis. We included data from prevalence surveys reporting on pulmonary smear-positive tuberculosis and bacteriologically positive tuberculosis. Because all forms of tuberculosis are included in notification data, we adjusted prevalence surveys to account for extrapulmonary cases. We predicted proportions of extrapulmonary tuberculosis among all tuberculosis cases for all locations and years by age and sex using data for the three forms of tuberculosis from the notification data and LDI as a covariate and applied them to data from prevalence surveys. We included a covariate to adjust smear-positive tuberculosis estimates to the value of bacteriologically positive tuberculosis. We found no systematic bias comparing data from studies that used both symptoms and chest x-rays as screening methods and studies that used only one of these methods. We therefore did not adjust these data but allowed DisMod-MR 2.1 to estimate the additional uncertainty associated with datapoints from studies that had used only one of the screening methods. Similarly, we added uncertainty to datapoints from subnational surveys. The method used to increase the uncertainty around datapoints in the dataset has been described in detail elsewhere.^19^ We also included the SEV scalar as a covariate for prevalence.

We matched each prevalence survey datapoint and tuberculosis cause-specific mortality rate (CSMR) among HIV-positive and HIV-negative individuals by location, year, age, and sex to calculate the excess mortality rate (EMR) as the ratio of CSMR to prevalence. We also matched each notification datapoint and tuberculosis CSMR by location, year, age, and sex to calculate EMR for data-rich countries (defined as countries with vital registration more than 95% complete for more than 25 years^3^ [appendix]), assuming a remission of 2—ie, an average duration of 6 months (1/0·5 years). We estimated priors on remission for countries where both incidence and prevalence data were available. We matched incidence and prevalence data by location, year, age, and sex and calculated remission as the ratio of incidence to prevalence minus the EMR. We ran two DisMod-MR 2.1 models, one for data-rich countries using the assumed remission, and another for remaining countries for which we used the estimated priors on remission. To reflect a gradient in EMR and remission, we added the log-transformed LDI as a covariate, with priors that as LDI values increase, EMR decreases and remission increases. For final results, we combined results from the two DisMod-MR 2.1 models. β coefficients and exponentiated values for covariates from the two models are shown in the appendix.

For each location, we included the following inputs in the DisMod model: case notifications representing all forms of tuberculosis, prevalence survey data (adjusted for extrapulmonary tuberculosis) if available, excess mortality priors, remission priors, and cause-specific mortality estimates (tuberculosis and HIV–tuberculosis combined) by age and sex. DisMod-MR 2.1 generated internally consistent estimates for each 5 year interval between 1990 and 2015 for 195 countries and territories.

As an example, the internally consistent modelling of tuberculosis (all forms) for male individuals in rural Gujarat, India, in 2015 is shown in the appendix. Statistical triangulation of death, prevalence, and adjusted notifications shows inconsistencies between data sources, as evident in the incidence model, showing a pattern in under-reporting increasing with age. The internally consistent modelling for each country and territory is available online.

The output from the DisMod-MR 2.1 model described above is for all forms of tuberculosis in HIV-negative and HIV-positive individuals. We applied the predicted location-specific and year-specific proportions of HIV–tuberculosis cases among all tuberculosis cases (as described in the HIV–tuberculosis mortality section above) to tuberculosis incident and prevalent cases from DisMod-MR 2.1 to generate HIV–tuberculosis incident and prevalent cases by location and year. Subsequently, we split the estimates on the basis of the age-sex pattern of estimated HIV prevalence by country-year to generate HIV–tuberculosis incident and prevalent cases for all locations and years by age and sex.

### SDI

The methods used to develop the SDI for GBD 2015 have been described in detail elsewhere.^3^, ^8^, ^9^, ^11^ Briefly, the SDI was computed on the basis of the geometric mean of three indicators: income per capita, average years of schooling, and total fertility rates. SDI scores were scaled from 0 (lowest income, lowest average years of schooling, and highest fertility) to 1 (highest income, highest average years of schooling, and lowest fertility), and each location was assigned an SDI score for each year. Average relationships between SDI and rates of tuberculosis incidence, prevalence, and mortality were estimated using spline regressions, which were then used to estimate expected values at each level of SDI. Five SDI quintiles were also created for country-year combinations. The results presented for SDI quintiles in this study reflect each country's position based on its SDI values in 2015.

### Comparative risk assessment

The basic approach for the GBD 2015 comparative risk assessment was to calculate the proportion of deaths and DALYs attributable to risk factors (eg, tuberculosis attributable to smoking) as a counterfactual to the hypothetical situation that populations had been exposed to a theoretical minimum level of exposure in the past. As in previous GBD studies, a set of behavioural, environmental and occupational, and metabolic risks were evaluated in GBD 2015. Inclusion of a risk–outcome pair was based on the evidence of convincing or probable causal relationship between the risk and the outcome. We had evidence for such a relationship between diabetes, alcohol use, and smoking and risk of tuberculosis.^11^, ^20^ Some risk factors (eg, indoor air pollution and malnutrition) have been hypothesised to have a strong link with tuberculosis, but we did not quantify the burden attributable to these risk factors because of insufficient evidence of a causal relationship.^21^, ^22^, ^23^ For example, evidence for indoor air pollution was based on cross-sectional studies (which are limited by their inability to establish a temporal relationship) and case-control studies (which are prone to recall bias as none of the studies measured indoor air pollution objectively).^23^ To date, we have not quantified the contribution of other classes of risk factors (eg, social, cultural, economic, and genetic factors).

DALYs were computed as the sum of years of life lost and years lived with disability for each location, age, sex, and year. Estimates of attributable DALYs (or number of deaths) were computed by multiplying DALYs (or number of deaths) for the outcome by the population-attributable fraction (PAF) for the risk-outcome pair for a given age, sex, location, and year. Full details of methods used in the comparative risk assessment have been reported elsewhere^11^ and are also provided in the appendix. To generate estimates of alcohol consumption in g per day, data from population surveys were used in combination with estimates of per-person consumption from the Food and Agriculture Organization^24^ and Global Information System on Alcohol and Health.^25^ For smoking, we included 2818 sources of primary data from the Global Health Data Exchange database.^26^ In addition to these primary data sources, we supplemented these data with secondary database estimates from the WHO InfoBase and International Smoking Statistics databases for sources for which primary data were unavailable. We included 281 sources from WHO InfoBase and 313 sources from International Smoking Statistics. For diabetes, we included 717 sources of population-based survey data identified through our systematic search of PubMed and the Global Health Data Exchange. A full list of data sources and citations for the three risk factors and RRs for the associations between risk factors and tuberculosis are provided in the appendix.

### Role of the funding source

The funder of the study had no role in study design, data collection, data analysis, data interpretation, or writing of the report. The corresponding author had full access to all the data in the study and had final responsibility for the decision to submit for publication.

## Results

### Levels and trends of tuberculosis incidence, prevalence, and mortality

**Figure 1:**
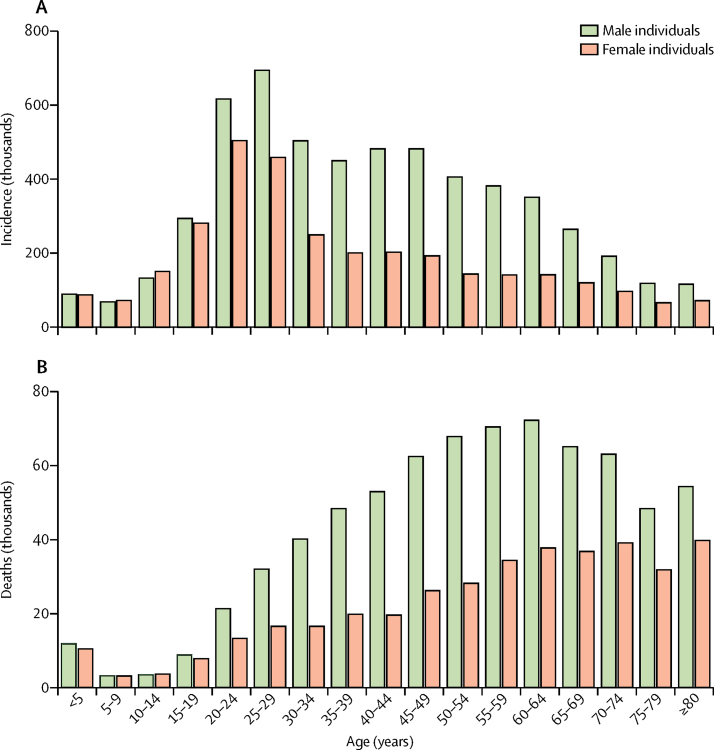
Global age-sex distribution of tuberculosis incidence (A) and deaths (B) in HIV-negative individuals in 2015

Globally, in 2015, 10·2 million (95% uncertainty interval [95% UI] 9·2 million to 11·5 million) tuberculosis incident cases occurred, 10·1 million (9·2 million to 11·1 million) prevalent cases occurred, and 1·3 million (1·1 million to 1·6 million) deaths from tuberculosis (HIV negative and HIV positive combined) occurred. Among individuals who were HIV negative, the number of incident cases was 8·8 million (8·0 million to 9·9 million), the number of prevalent cases was 8·9 million (8·1 million to 9·7 million), and the number of deaths was 1·1 million (0·9 million to 1·4 million). Globally, among HIV-negative individuals, more incident cases and deaths occurred in men than in women in most age groups (figure 1). The age-standardised tuberculosis incidence rate (per 100 000 people) among men (154·4 [140·0–172·2]) was 1·8 times higher than that among women (86·3 [78·0–97·4]), and the age-standardised tuberculosis mortality rate (per 100 000 people) among men (21·9 [16·5–29·5]) was about twice as high as that among women (10·8 [8·5–13·1]). We estimated that 690 262 (551 275–859 100) incident cases of tuberculosis, 612 183 (498 242–744 815) prevalent cases, and 69 681 (57 982–88 962) deaths from tuberculosis occurred among children younger than 15 years in 2015.

Age-standardised tuberculosis mortality rates (HIV negative and HIV positive combined) changed at −1·8% (95% UI −2·4 to −1·4) per year from 1990 to 2005, with accelerated improvements from 2005 to 2015 (−4·6% [−5·4 to −3·9] per year; appendix). The corresponding change among individuals who were HIV negative was −3·1% (−3·6 to −2·6) per year from 1990 to 2005 and −4·1% (−5·0 to −3·4) per year from 2005 to 2015 (table 1). A much slower decrease has occurred in global age-standardised tuberculosis incidence and prevalence annualised rates of change (ARCs) than in mortality rates among HIV-negative individuals. We observed a similar pattern when including HIV-positive individuals (appendix).

**Table 1:** Age-standardised rates of tuberculosis incidence, prevalence, and mortality per 100 000 population and annualised rates of change in HIV-negative individuals

	**Age-standardised rates in 2015 (per 100 000 population)**			**Annualised rate of change (%)**					
	Incidence	Prevalence	Mortality	1990–2005			2005–15		
				Incidence	Prevalence	Mortality	Incidence	Prevalence	Mortality
Global	119·6 (108·1 to 134·0)	120·3 (110·0 to 131·6)	16·0 (13·1 to 20·1)	−1·5% (−1·7 to −1·3)	−1·2% (−1·4 to −1·1)	−3·1% (−3·6 to −2·6)	−1·6% (−1·9 to −1·2)	−0·7% (−1·0 to −0·5)	−4·1% (−5·0 to −3·4)
High SDI	28·2 (25·8 to 30·2)	16·3 (15·2 to 17·5)	1·3 (1·3 to 1·4)	−1·1% (−1·5 to −0·8)	−0·6% (−0·9 to −0·3)	−1·1% (−1·5 to −0·8)	−3·1% (−3·6 to −2·5)	−3·1% (−3·5 to −2·7)	−7·2% (−7·9 to −6·5)
High-middle SDI	89·1 (80·1 to 101·3)	84·7 (76·5 to 93·6)	5·7 (4·4 to 6·7)	−0·8% (−1·0 to −0·5)	−0·7% (−1·0 to −0·5)	−3·9% (−4·7 to −3·1)	−1·3% (−1·7 to −0·9)	−0·2% (−0·5 to 0·0)	−5·3% (−6·0 to −4·6)
Middle SDI	157·8 (143·2 to 176·3)	178·6 (163·9 to 194·1)	17·3 (14·6 to 22·7)	−2·0% (−2·2 to −1·8)	−1·8% (−2·0 to −1·6)	−4·3% (−5·2 to −3·4)	−2·0% (−2·4 to −1·6)	−1·1% (−1·3 to −0·8)	−5·4% (−6·6 to −4·2)
Low-middle SDI	189·2 (170·3 to 210·8)	187·8 (168·8 to 207·1)	44·6 (35·4 to 55·5)	−2·4% (−2·7 to −2·2)	−1·9% (−2·0 to −1·7)	−3·2% (−4·1 to −2·6)	−2·0% (−2·4 to −1·5)	−1·2% (−1·5 to −0·8)	−4·0% (−5·5 to −2·8)
Low SDI	191·0 (175·6 to 210·8)	163·0 (149·4 to 177·4)	71·6 (51·8 to 98·2)	−1·0% (−1·2 to −0·8)	−1·3% (−1·4 to −1·2)	−1·5% (−2·7 to 0·1)	−1·5% (−1·8 to −1·1)	−1·1% (−1·4 to −0·9)	−2·8% (−4·8 to −0·9)
High-income Asia Pacific	31·2 (28·7 to 33·8)	15·1 (13·8 to 16·6)	1·8 (1·7 to 1·9)	−5·1% (−5·5 to −4·8)	−5·3% (−5·7 to −5·0)	−6·3% (−6·6 to −5·9)	−1·4% (−2·0 to −0·9)	−1·4% (−2·1 to −0·9)	−4·6% (−5·3 to −4·0)
Central Asia	96·6 (88·7 to 105·4)	74·6 (68·7 to 81·2)	7·5 (5·4 to 8·5)	0·1% (−0·2 to 0·4)	0·5% (0·4 to 0·7)	1·5% (−0·5 to 2·4)	−3·4% (−4·0 to −2·9)	−2·8% (−3·2 to −2·3)	−6·8% (−7·7 to −6·0)
East Asia	97·5 (88·1 to 110·8)	123·6 (112·4 to 135·3)	3·5 (3·0 to 5·3)	−1·0% (−1·5 to −0·5)	−1·2% (−1·5 to −0·9)	−6·8% (−7·9 to −4·5)	−1·9% (−2·4 to −1·5)	−0·5% (−0·8 to −0·1)	−7·7% (−9·0 to −5·9)
South Asia	204·4 (181·1 to 231·2)	210·9 (188·3 to 235·5)	44·2 (34·0 to 54·0)	−2·7% (−2·9 to −2·4)	−1·9% (−2·1 to −1·7)	−3·6% (−4·3 to −2·9)	−2·5% (−3·0 to −2·0)	−1·4% (−1·8 to −1·0)	−4·6% (−6·0 to −3·6)
Southeast Asia	208·7 (192·8 to 229·7)	228·7 (213·4 to 245·6)	35·3 (29·4 to 46·5)	−2·9% (−3·1 to −2·6)	−2·8% (−3·0 to −2·6)	−4·0% (−5·2 to −2·7)	−1·8% (−2·2 to −1·3)	−1·4% (−1·7 to −1·1)	−4·8% (−6·6 to −3·3)
Australasia	7·5 (6·1 to 9·2)	3·8 (3·0 to 4·7)	0·2 (0·2 to 0·2)	−2·0% (−2·7 to −1·4)	−2·0% (−2·7 to −1·3)	−5·1% (−5·7 to −4·5)	0·3% (−0·4 to 1·1)	0·4% (−0·3 to 1·2)	−3·9% (−4·9 to −2·8)
The Caribbean	34·6 (31·6 to 37·8)	21·8 (19·9 to 23·8)	3·9 (2·9 to 6·1)	−2·8% (−3·1 to −2·6)	−2·9% (−3·1 to −2·7)	−4·0% (−5·5 to −2·2)	−0·8% (−1·2 to −0·3)	−0·7% (−1·2 to −0·3)	−2·5% (−4·0 to −1·1)
Central Europe	31·8 (29·2 to 34·4)	16·2 (14·9 to 17·6)	1·4 (1·3 to 1·5)	−1·9% (−2·1 to −1·6)	−1·8% (−2·0 to −1·6)	−3·6% (−4·0 to −3·3)	−2·8% (−3·2 to −2·4)	−2·7% (−3·1 to −2·3)	−6·5% (−7·2 to −5·6)
Eastern Europe	116·9 (106·5 to 125·3)	63·5 (59·4 to 67·8)	5·8 (5·3 to 6·3)	2·5% (2·0 to 2·9)	3·4% (3·0 to 3·8)	5·3% (4·8 to 5·8)	−3·5% (−4·1 to −2·7)	−3·9% (−4·5 to −3·4)	−8·4% (−9·3 to −7·5)
Western Europe	10·6 (8·8 to 12·6)	5·3 (4·4 to 6·3)	0·4 (0·4 to 0·5)	−5·0% (−5·3 to −4·6)	−5·0% (−5·4 to −4·6)	−6·1% (−6·4 to −5·8)	−1·5% (−2·2 to −0·9)	−1·5% (−2·3 to −0·8)	−4·6% (−5·1 to −4·0)
Andean Latin America	72·7 (65·1 to 82·3)	48·0 (42·7 to 54·2)	8·0 (6·4 to 13·8)	−7·0% (−7·2 to −6·6)	−7·2% (−7·5 to −6·9)	−8·3% (−10·2 to −2·9)	−2·3% (−2·8 to −1·6)	−2·0% (−2·7 to −1·4)	−4·8% (−5·9 to −3·8)
Central Latin America	26·7 (24·9 to 28·6)	12·7 (11·7 to 13·7)	2·7 (2·6 to 3·0)	−4·6% (−4·8 to −4·4)	−4·5% (−4·7 to −4·3)	−7·2% (−7·6 to −6·8)	−2·3% (−2·7 to −1·9)	−2·2% (−2·6 to −1·9)	−4·6% (−5·1 to −4·1)
Southern Latin America	23·5 (21·4 to 25·6)	12·9 (11·8 to 14·1)	1·6 (1·5 to 1·8)	−3·8% (−4·2 to −3·3)	−2·8% (−3·2 to −2·5)	−5·6% (−6·0 to −5·2)	−1·5% (−2·1 to −1·0)	−1·8% (−2·3 to −1·3)	−4·5% (−5·3 to −3·6)
Tropical Latin America	35·0 (30·9 to 38·7)	25·1 (21·9 to 28·0)	3·0 (2·1 to 3·9)	−1·3% (−1·7 to −0·9)	−1·4% (−1·8 to −1·0)	−3·9% (−5·4 to −2·9)	−1·6% (−2·0 to −1·2)	−1·4% (−1·8 to −1·0)	−4·3% (−5·3 to −2·9)
North Africa and the Middle East	36·7 (32·7 to 41·2)	26·3 (23·4 to 29·4)	5·0 (4·1 to 6·8)	−2·9% (−3·1 to −2·8)	−3·1% (−3·2 to −3·0)	−3·4% (−4·4 to −2·0)	−1·0% (−1·3 to −0·6)	−0·9% (−1·3 to −0·5)	−3·5% (−4·6 to −2·4)
High-income North America	3·8 (3·2 to 4·5)	2·0 (1·6 to 2·4)	0·2 (0·2 to 0·2)	−5·6% (−6·3 to −4·8)	−5·5% (−6·2 to −4·8)	−7·1% (−7·4 to −6·8)	−2·0% (−2·5 to −1·6)	−1·9% (−2·5 to −1·5)	−3·3% (−3·8 to −2·8)
Oceania	67·4 (61·0 to 75·3)	66·0 (59·8 to 72·7)	11·1 (6·9 to 16·7)	−1·4% (−1·6 to −1·1)	−1·5% (−1·7 to −1·3)	−2·7% (−4·4 to −0·9)	0·1% (−0·4 to 0·7)	0·4% (−0·1 to 0·9)	−3·2% (−5·3 to −1·0)
Central sub-Saharan Africa	270·2 (241·6 to 300·4)	219·0 (194·9 to 243·7)	90·3 (44·7 to 190·3)	−0·7% (−0·9 to −0·4)	−1·3% (−1·6 to −1·0)	−0·3% (−3·6 to 3·1)	−1·1% (−1·5 to −0·7)	−0·9% (−1·3 to −0·5)	−2·6% (−6·6 to 0·7)
Eastern sub-Saharan Africa	186·5 (171·5 to 205·7)	156·4 (144·6 to 169·0)	60·1 (38·8 to 80·1)	−1·0% (−1·2 to −0·7)	−1·2% (−1·4 to −1·0)	−2·0% (−3·7 to −0·6)	−1·5% (−1·9 to −1·0)	−1·0% (−1·4 to −0·7)	−3·1% (−5·9 to −0·6)
Southern sub-Saharan Africa	724·6 (621·4 to 860·7)	630·6 (547·5 to 718·3)	68·4 (48·0 to 83·5)	2·6% (1·8 to 3·3)	2·5% (2·0 to 3·1)	0·3% (−2·7 to 2·3)	−0·7% (−1·5 to 0·1)	−0·5% (−1·1 to 0·1)	−3·7% (−5·5 to −1·7)
Western sub-Saharan Africa	146·7 (133·4 to 162·7)	134·3 (122·4 to 147·8)	40·3 (32·2 to 60·6)	−1·1% (−1·3 to −1·0)	−1·2% (−1·3 to −1·1)	−2·2% (−3·5 to −1·0)	−0·8% (−1·2 to −0·4)	−0·6% (−1·0 to −0·2)	−2·9% (−4·6 to −1·3)

Data in parentheses are 95% uncertainty intervals. SDI=Socio-demographic Index.

When examining ARCs by SDI quintile, we observed a gradient in ARCs for tuberculosis age-standardised mortality rates among HIV-negative individuals during the period 2005–15: ARCs ranged from −2·8% (95% UI −4·8 to −0·9) in the lowest SDI quintile to −7·2% (−7·9 to −6·5) in the highest quintile. We did not see a clear gradient, however, in ARCs for tuberculosis incidence and prevalence among HIV-negative individuals across the SDI quintiles (table 1). Across regions, in the period 2005–15, incidence ARCs among people who were HIV negative ranged from 0·3% (−0·4 to 1·1) in Australasia to −3·5% (−4·1 to −2·7) in eastern Europe (table 2). South Asia accounted for 35·8% of incident cases and 49·2% of deaths in 2015. Southeast Asia accounted for 14·6% of incident cases and 15·5% of deaths in 2015. In eastern Europe, during the period 1990–2005, mortality, incidence, and prevalence all increased. In the period 2005–15, however, the trends for all three indicators reversed to show decreasing trends.

**Table 2:** Tuberculosis incidence, prevalence, and deaths and annualised rates of change of age-standardised rates in HIV-negative individuals

		**Incidence, prevalence, and deaths in 2015**			**Annualised rate of change (%)**					
		Incidence	Prevalence	Deaths	1990–2005			2005–15		
					Incidence	Prevalence	Deaths	Incidence	Prevalence	Deaths
Global		8 832 342 (7 968 649 to 9 924 953)	8 861 169 (8 076 335 to 9 707 220)	1 112 607 (909 769 to 1 392 789)	−1·5% (−1·7 to −1·3)	−1·2% (−1·4 to −1·1)	−3·1% (−3·6 to −2·6)	−1·6% (−1·9 to −1·2)	−0·7% (−1·0 to −0·5)	−4·1% (−5·0 to −3·4)
High-income Asia Pacific		76 150 (71 440 to 81 143)	35 162 (32 781 to 37 782)	7270 (6806 to 7785)	−5·1% (−5·5 to −4·8)	−5·3% (−5·7 to −5·0)	−6·3% (−6·6 to −5·9)	−1·4% (−2·0 to −0·9)	−1·4% (−2·1 to −0·9)	−4·6% (−5·3 to −4·0)
	Brunei	277 (244 to 318)	191 (168 to 218)	13 (7 to 15)	−3·2% (−3·5 to −2·8)	−3·2% (−3·5 to −2·9)	−3·4% (−4·9 to −1·5)	−0·7% (−1·3 to −0·1)	−0·7% (−1·3 to −0·2)	−1·3% (−2·9 to 0·4)
	Japan	31 729 (29 609 to 33 916)	14 820 (13 424 to 16 361)	3971 (3672 to 4294)	−3·9% (−4·3 to −3·5)	−3·8% (−4·3 to −3·3)	−5·1% (−5·4 to −4·8)	−1·8% (−3·2 to −0·5)	−1·4% (−2·9 to −0·2)	−4·7% (−5·5 to −3·9)
	Singapore	1983 (1765 to 2220)	948 (838 to 1058)	63 (54 to 74)	−4·3% (−4·8 to −3·9)	−4·5% (−4·9 to −4·0)	−7·5% (−8·3 to −6·7)	1·1% (0·3 to 2·0)	1·0% (0·1 to 1·9)	−6·4% (−8·1 to −4·8)
	South Korea	42 161 (39 134 to 45 224)	19 203 (17 867 to 20 650)	3224 (2871 to 3638)	−6·2% (−6·7 to −5·9)	−6·5% (−6·9 to −6·2)	−7·6% (−8·3 to −6·9)	−2·1% (−2·6 to −1·6)	−2·4% (−3·0 to −2·0)	−5·6% (−6·8 to −4·3)
Central Asia		88 915 (81 027 to 97 149)	69 400 (63 414 to 75 835)	6167 (4375 to 7043)	0·1% (−0·2 to 0·4)	0·5% (0·4 to 0·7)	1·5% (−0·5 to 2·4)	−3·4% (−4·0 to −2·9)	−2·8% (−3·2 to −2·3)	−6·8% (−7·7 to −6·0)
	Armenia	1158 (997 to 1343)	1041 (908 to 1199)	106 (46 to 137)	1·4% (1·0 to 1·9)	1·2% (0·8 to 1·6)	1·9% (−2·9 to 3·8)	−0·0% (−0·8 to 0·6)	0·7% (−0·1 to 1·4)	−4·9% (−6·6 to −3·0)
	Azerbaijan	11 126 (9597 to 13 006)	9933 (8806 to 11 316)	692 (502 to 986)	−1·2% (−1·7 to −0·7)	−1·4% (−1·8 to −1·1)	−0·5% (−2·7 to 1·2)	−1·2% (−2·2 to −0·2)	−0·5% (−1·3 to 0·3)	−6·6% (−9·7 to −3·7)
	Georgia	2251 (1984 to 2583)	1953 (1742 to 2188)	202 (152 to 282)	−2·9% (−3·5 to −2·3)	−3·2% (−3·6 to −2·8)	−1·6% (−3·1 to 1·4)	0·9% (0·2 to 1·6)	1·5% (0·9 to 2·1)	−2·4% (−5·3 to −0·5)
	Kazakhstan	33 265 (30 078 to 36 389)	18 837 (17 254 to 20 424)	1519 (1271 to 1815)	1·3% (0·7 to 1·8)	2·5% (2·1 to 2·9)	3·6% (3·0 to 4·2)	−5·6% (−6·6 to −4·7)	−6·2% (−6·9 to −5·5)	−10·3% (−12·1 to −8·4)
	Kyrgyzstan	4422 (3884 to 5038)	3915 (3478 to 4410)	546 (329 to 681)	0·1% (−0·3 to 0·7)	−0·0% (−0·4 to 0·4)	2·6% (−2·3 to 4·4)	−2·2% (−2·9 to −1·5)	−1·7% (−2·3 to −1·0)	−4·4% (−6·0 to −2·3)
	Mongolia	4363 (3803 to 5030)	3949 (3522 to 4422)	322 (212 to 386)	−1·1% (−1·6 to −0·7)	−1·4% (−1·7 to −1·1)	−1·3% (−4·4 to 0·3)	−0·8% (−1·6 to −0·1)	−0·4% (−1·0 to 0·1)	−5·5% (−6·9 to −4·2)
	Tajikistan	5083 (4334 to 5994)	4598 (3971 to 5272)	501 (279 to 671)	−0·6% (−1·1 to −0·1)	−0·7% (−1·1 to −0·3)	2·7% (−2·6 to 4·9)	−1·5% (−2·3 to −0·7)	−1·2% (−1·8 to −0·5)	−5·4% (−6·9 to −4·0)
	Turkmenistan	5026 (4341 to 5846)	4658 (4149 to 5253)	333 (240 to 426)	−0·6% (−1·2 to −0·1)	−0·7% (−1·1 to −0·3)	−0·3% (−2·3 to 1·3)	−2·1% (−3·0 to −1·4)	−1·5% (−2·3 to −0·9)	−6·3% (−7·9 to −4·6)
	Uzbekistan	22 222 (19 151 to 25 563)	20 515 (18 098 to 23 191)	1946 (920 to 2498)	0·6% (0·2 to 1·1)	0·6% (0·3 to 1·0)	0·4% (−3·5 to 1·9)	−1·9% (−2·6 to −1·2)	−1·4% (−2·0 to −0·8)	−5·7% (−7·0 to −4·3)
East Asia		1 540 724 (1 391 577 to 1 749 147)	1 940 482 (1 757 081 to 2 126 769)	51 814 (44 920 to 79 180)	−1·0% (−1·5 to −0·5)	−1·2% (−1·5 to −0·9)	−6·8% (−7·9 to −4·5)	−1·9% (−2·4 to −1·5)	−0·5% (−0·8 to −0·1)	−7·7% (−9·0 to −5·9)
	China	1 513 259 (1 366 963 to 1 717 735)	1 908 212 (1 728 325 to 2 090 065)	48 922 (41 055 to 76 344)	−1·0% (−1·5 to −0·5)	−1·2% (−1·5 to −0·9)	−6·9% (−8·1 to −4·5)	−2·0% (−2·4 to −1·5)	−0·5% (−0·8 to −0·1)	−7·9% (−9·3 to −6·0)
	North Korea	17 438 (15 322 to 20 010)	19 392 (17 004 to 22 306)	2145 (865 to 3929)	−2·2% (−2·6 to −1·9)	−2·7% (−3·0 to −2·4)	−1·0% (−4·0 to 3·3)	0·8% (0·2 to 1·4)	1·1% (0·5 to 1·7)	−3·2% (−6·4 to 0·9)
	Taiwan	10 028 (8701 to 11 625)	12 878 (11 268 to 14 838)	746 (262 to 1026)	−1·2% (−1·6 to −0·9)	−1·4% (−1·7 to −1·0)	−5·9% (−7·1 to −4·4)	−1·0% (−1·6 to −0·5)	−0·6% (−1·1 to −0·0)	−4·7% (−6·8 to −2·6)
South Asia		3 166 338 (2 784 304 to 3 618 869)	3 260 702 (2 893 539 to 3 666 211)	547 710 (425 307 to 675 823)	−2·7% (−2·9 to −2·4)	−1·9% (−2·1 to −1·7)	−3·6% (−4·3 to −2·9)	−2·5% (−3·0 to −2·0)	−1·4% (−1·8 to −1·0)	−4·6% (−6·0 to −3·6)
	Afghanistan	24 513 (21 596 to 27 834)	17 666 (15 508 to 19 984)	4536 (2258 to 7069)	−2·0% (−2·3 to −1·7)	−2·6% (−2·9 to −2·3)	−1·4% (−3·7 to 1·5)	−1·3% (−1·8 to −0·8)	−0·9% (−1·4 to −0·3)	−4·3% (−7·3 to −1·2)
	Bangladesh	150 804 (135 802 to 168 781)	106 507 (95 917 to 119 086)	23 070 (10 213 to 30 278)	−3·8% (−4·1 to −3·4)	−3·7% (−4·1 to −3·5)	−5·1% (−6·4 to −3·1)	0·5% (−0·2 to 1·2)	1·1% (0·4 to 1·7)	−0·6% (−6·8 to 2·0)
	Bhutan	1510 (1296 to 1767)	1314 (1144 to 1514)	149 (41 to 258)	−2·0% (−2·4 to −1·8)	−1·8% (−2·1 to −1·5)	−4·5% (−6·6 to −2·8)	−0·9% (−1·5 to −0·3)	−0·6% (−1·1 to −0·1)	−4·1% (−6·4 to −1·6)
	India	2 667 141 (2 320 632 to 3 081 220)	2 803 442 (2 462 533 to 3 190 619)	466 837 (366 635 to 594 312)	−2·7% (−2·9 to −2·4)	−1·9% (−2·1 to −1·7)	−3·6% (−4·4 to −2·8)	−2·8% (−3·3 to −2·2)	−1·5% (−1·9 to −1·0)	−4·9% (−6·5 to −3·8)
	Nepal	50 082 (45 111 to 55 995)	44 109 (39 465 to 49 137)	11 242 (5319 to 16 746)	−2·8% (−3·1 to −2·6)	−2·6% (−2·9 to −2·4)	−3·4% (−5·3 to −1·7)	−2·1% (−2·5 to −1·7)	−1·8% (−2·2 to −1·4)	−3·5% (−6·3 to −0·8)
	Pakistan	296 802 (268 904 to 330 818)	305 330 (282 192 to 331 130)	46 413 (37 031 to 58 669)	−1·5% (−2·0 to −1·1)	−1·6% (−1·9 to −1·2)	−2·0% (−4·1 to −0·2)	−1·7% (−2·3 to −1·1)	−1·5% (−1·9 to −1·0)	−3·1% (−5·1 to −1·0)
Southeast Asia		1 287 016 (1 178 203 to 1 425 788)	1 387 082 (1 289 248 to 1 494 382)	172 531 (143 821 to 228 717)	−2·9% (−3·1 to −2·6)	−2·8% (−3·0 to −2·6)	−4·0% (−5·2 to −2·7)	−1·8% (−2·2 to −1·3)	−1·4% (−1·7 to −1·1)	−4·8% (−6·6 to −3·3)
	Cambodia	18 017 (16 324 to 20 270)	25 941 (23 668 to 28 565)	3135 (2377 to 4593)	−2·1% (−2·4 to −1·8)	−1·7% (−2·0 to −1·4)	−3·8% (−5·2 to −2·1)	−2·2% (−2·7 to −1·7)	−1·7% (−2·2 to −1·3)	−5·5% (−7·7 to −3·7)
	Indonesia	814 823 (737 504 to 912 701)	860 743 (795 915 to 932 476)	96 294 (74 720 to 140 800)	−2·9% (−3·2 to −2·5)	−2·9% (−3·1 to −2·6)	−3·3% (−4·7 to −2·1)	−1·8% (−2·3 to −1·1)	−1·3% (−1·8 to −0·9)	−4·2% (−6·9 to −1·9)
	Laos	4916 (4403 to 5500)	6056 (5425 to 6782)	883 (575 to 1555)	−3·8% (−4·1 to −3·5)	−3·6% (−3·9 to −3·4)	−5·0% (−7·3 to −2·7)	−2·4% (−3·0 to −2·0)	−1·7% (−2·2 to −1·2)	−5·8% (−7·9 to −3·7)
	Malaysia	24 219 (21 229 to 27 250)	23 778 (21 175 to 26 507)	1248 (926 to 2179)	−1·9% (−2·2 to −1·5)	−1·8% (−2·1 to −1·6)	−4·7% (−6·0 to −3·4)	0·6% (−0·1 to 1·5)	0·3% (−0·3 to 0·9)	−3·4% (−5·2 to −1·5)
	Maldives	148 (124 to 179)	141 (119 to 170)	8 (5 to 10)	−4·5% (−4·8 to −3·9)	−4·5% (−4·9 to −4·1)	−8·3% (−9·9 to −6·1)	−0·1% (−0·8 to 0·5)	−0·2% (−0·8 to 0·5)	−5·5% (−7·8 to −3·2)
	Myanmar	62 175 (56 667 to 68 658)	100 992 (92 304 to 110 317)	20 549 (11 832 to 33 014)	−3·2% (−3·5 to −2·8)	−2·3% (−2·7 to −2·0)	−3·9% (−7·0 to −0·6)	−1·5% (−2·0 to −1·1)	−0·4% (−0·9 to 0·0)	−5·6% (−9·4 to −1·9)
	Philippines	199 719 (182 790 to 220 668)	197 313 (184 853 to 210 837)	23 378 (21 009 to 26 006)	−2·6% (−3·0 to −2·2)	−2·6% (−2·9 to −2·3)	−2·9% (−3·4 to −2·5)	−2·7% (−3·5 to −1·8)	−2·8% (−3·3 to −2·3)	−5·7% (−6·8 to −4·4)
	Sri Lanka	12 919 (11 989 to 13 950)	6619 (6188 to 7094)	729 (563 to 938)	−4·3% (−4·7 to −3·9)	−4·0% (−4·3 to −3·6)	−5·1% (−5·7 to −4·4)	−2·0% (−2·6 to −1·3)	−2·5% (−3·0 to −1·9)	−7·9% (−10·4 to −5·4)
	Thailand	64 696 (58 286 to 71 984)	66 215 (60 795 to 71 796)	7408 (5171 to 9563)	−3·1% (−3·5 to −2·7)	−3·3% (−3·6 to −3·0)	−7·9% (−9·5 to −3·1)	−0·6% (−1·3 to −0·0)	−0·5% (−1·0 to −0·0)	−2·9% (−4·8 to −1·1)
	Timor-Leste	1574 (1373 to 1820)	1501 (1305 to 1731)	152 (90 to 320)	−0·7% (−1·0 to −0·4)	−0·6% (−0·9 to −0·3)	−3·7% (−5·9 to −1·7)	−0·2% (−0·8 to 0·3)	0·7% (0·1 to 1·2)	−5·7% (−8·9 to −3·0)
	Vietnam	81 371 (73 978 to 89 808)	95 416 (87 914 to 103 582)	18 409 (11 243 to 24 214)	−3·2% (−3·4 to −2·8)	−2·7% (−2·9 to −2·3)	−5·1% (−7·6 to −2·8)	−1·9% (−2·4 to −1·3)	−1·5% (−1·9 to −1·0)	−5·3% (−9·0 to −2·2)
Australasia		2133 (1770 to 2542)	1060 (865 to 1265)	82 (73 to 92)	−2·0% (−2·7 to −1·4)	−2·0% (−2·7 to −1·3)	−5·1% (−5·7 to −4·5)	0·3% (−0·4 to 1·1)	0·4% (−0·3 to 1·2)	−3·9% (−4·9 to −2·8)
	Australia	1766 (1455 to 2111)	879 (713 to 1057)	66 (58 to 76)	−1·8% (−2·6 to −1·1)	−1·8% (−2·6 to −1·1)	−5·0% (−5·8 to −4·2)	0·7% (−0·1 to 1·6)	0·8% (0·1 to 1·7)	−3·4% (−4·7 to −2·1)
	New Zealand	367 (304 to 430)	181 (147 to 213)	15 (14 to 17)	−2·8% (−3·3 to −2·3)	−2·7% (−3·3 to −2·2)	−5·4% (−6·1 to −4·7)	−1·1% (−1·8 to −0·6)	−1·0% (−1·7 to −0·5)	−5·5% (−6·6 to −4·4)
The Caribbean		15 798 (14 392 to 17 277)	10 003 (9085 to 10 956)	1713 (1306 to 2698)	−2·8% (−3·1 to −2·6)	−2·9% (−3·1 to −2·7)	−4·0% (−5·5 to −2·2)	−0·8% (−1·2 to −0·3)	−0·7% (−1·2 to −0·3)	−2·5% (−4·0 to −1·1)
	Antigua and Barbuda	27 (22 to 33)	13 (11 to 16)	1 (1 to 1)	−0·3% (−0·6 to 0·0)	−0·2% (−0·6 to 0·1)	−1·4% (−2·3 to −0·5)	−0·2% (−1·1 to 0·6)	−0·1% (−1·0 to 0·7)	−5·2% (−7·0 to −3·7)
	The Bahamas	110 (96 to 127)	78 (68 to 90)	9 (7 to 14)	−2·9% (−3·2 to −2·5)	−2·8% (−3·2 to −2·5)	−3·5% (−5·0 to −2·1)	−2·6% (−3·4 to −1·9)	−2·6% (−3·3 to −1·8)	−2·7% (−5·0 to −0·4)
	Barbados	65 (54 to 78)	31 (26 to 38)	3 (3 to 3)	0·1% (−0·2 to 0·5)	0·1% (−0·2 to 0·5)	−1·5% (−2·3 to −0·8)	−0·1% (−1·1 to 0·8)	0·0% (−1·0 to 1·0)	−3·7% (−5·2 to −2·1)
	Belize	158 (141 to 178)	111 (100 to 123)	15 (11 to 21)	0·6% (0·2 to 1·1)	1·0% (0·6 to 1·4)	−0·5% (−4·4 to 1·0)	−2·4% (−3·1 to −1·8)	−2·6% (−3·3 to −2·0)	−3·7% (−5·7 to −1·6)
	Bermuda	21 (16 to 26)	10 (8 to 13)	0 (0 to 0)	−0·1% (−0·4 to 0·3)	−0·1% (−0·4 to 0·3)	−3·8% (−4·6 to −3·0)	1·8% (1·1 to 2·7)	1·9% (1·2 to 2·8)	−4·5% (−5·8 to −3·1)
	Cuba	1327 (1093 to 1576)	625 (511 to 742)	42 (38 to 47)	−2·5% (−3·0 to −2·0)	−2·4% (−2·9 to −1·9)	−5·3% (−6·1 to −4·6)	1·1% (0·5 to 1·6)	1·3% (0·7 to 1·8)	−3·7% (−4·9 to −2·5)
	Dominica	27 (24 to 30)	18 (16 to 21)	3 (2 to 4)	−1·1% (−1·4 to −0·7)	−0·9% (−1·2 to −0·6)	−2·0% (−3·8 to −0·6)	−0·4% (−1·1 to 0·2)	−0·3% (−0·9 to 0·4)	−2·4% (−4·2 to −0·5)
	Dominican Republic	6278 (5609 to 7043)	4272 (3807 to 4763)	528 (403 to 1016)	−3·8% (−4·2 to −3·4)	−3·9% (−4·2 to −3·5)	−5·8% (−7·5 to −2·8)	−1·3% (−1·9 to −0·6)	−1·2% (−1·9 to −0·6)	−3·5% (−4·8 to −2·4)
	Grenada	23 (19 to 29)	16 (13 to 21)	1 (1 to 2)	−0·0% (−0·3 to 0·3)	0·2% (−0·0 to 0·5)	−1·7% (−5·5 to −0·2)	0·5% (−0·2 to 1·1)	0·6% (−0·1 to 1·3)	−3·5% (−4·9 to −2·1)
	Guyana	635 (583 to 692)	432 (401 to 466)	66 (46 to 88)	1·8% (1·4 to 2·2)	2·1% (1·7 to 2·5)	−0·4% (−4·0 to 1·5)	−1·5% (−2·0 to −0·9)	−1·4% (−1·9 to −0·8)	−3·7% (−5·7 to −1·6)
	Haiti	5235 (4716 to 5774)	3317 (2996 to 3658)	891 (551 to 1435)	−3·2% (−3·6 to −2·9)	−3·7% (−4·0 to −3·3)	−3·3% (−5·5 to −0·8)	−1·5% (−1·9 to −1·0)	−1·5% (−2·0 to −1·0)	−2·5% (−5·2 to −0·1)
	Jamaica	229 (182 to 289)	163 (128 to 209)	18 (12 to 37)	−3·2% (−3·8 to −2·9)	−3·1% (−3·5 to −2·7)	−4·5% (−6·3 to −1·7)	0·2% (−0·5 to 0·9)	0·3% (−0·6 to 1·1)	−2·4% (−4·8 to −0·1)
	Puerto Rico	421 (346 to 503)	210 (171 to 251)	23 (20 to 26)	−7·9% (−8·7 to −7·2)	−8·0% (−8·7 to −7·4)	−8·5% (−9·2 to −7·8)	−1·7% (−2·8 to −0·6)	−1·9% (−3·1 to −0·8)	−4·5% (−5·7 to −3·3)
	Saint Lucia	80 (72 to 88)	38 (34 to 41)	7 (6 to 7)	−0·9% (−1·2 to −0·5)	−0·8% (−1·1 to −0·5)	−2·0% (−2·8 to −1·2)	−3·5% (−4·1 to −2·9)	−3·4% (−4·1 to −2·8)	−4·6% (−5·9 to −3·2)
	Saint Vincent and the Grenadines	47 (42 to 53)	22 (19 to 24)	3 (3 to 3)	0·5% (0·2 to 0·9)	0·6% (0·3 to 0·9)	−1·0% (−1·9 to −0·1)	−1·9% (−2·6 to −1·3)	−1·8% (−2·5 to −1·2)	−4·0% (−5·3 to −2·8)
	Suriname	132 (115 to 152)	91 (79 to 105)	8 (6 to 11)	−1·1% (−1·4 to −0·7)	−0·9% (−1·2 to −0·7)	−1·8% (−3·4 to −0·7)	−1·6% (−2·3 to −0·8)	−1·4% (−2·2 to −0·7)	−4·8% (−6·8 to −2·9)
	Trinidad and Tobago	381 (336 to 424)	185 (165 to 205)	18 (15 to 21)	−2·1% (−2·4 to −1·7)	−2·0% (−2·3 to −1·6)	−4·0% (−4·8 to −3·3)	−1·5% (−2·2 to −0·9)	−1·5% (−2·2 to −0·8)	−4·3% (−5·8 to −2·6)
	Virgin Islands	29 (23 to 36)	20 (16 to 25)	1 (1 to 1)	−0·3% (−0·7 to −0·0)	−0·2% (−0·6 to 0·2)	−3·5% (−4·5 to −2·5)	2·4% (1·7 to 3·0)	2·5% (1·8 to 3·2)	−2·0% (−3·5 to −0·6)
Central Europe		41 646 (38 561 to 44 742)	20 989 (19 554 to 22 417)	2332 (2161 to 2545)	−1·9% (−2·1 to −1·6)	−1·8% (−2·0 to −1·6)	−3·6% (−4·0 to −3·3)	−2·8% (−3·2 to −2·4)	−2·7% (−3·1 to −2·3)	−6·5% (−7·2 to −5·6)
	Albania	447 (348 to 562)	318 (246 to 414)	12 (8 to 22)	−1·1% (−1·5 to −0·7)	−0·8% (−1·2 to −0·4)	−6·9% (−8·8 to −3·3)	2·4% (1·7 to 3·0)	2·7% (2·0 to 3·4)	−5·8% (−8·7 to −4·1)
	Bosnia and Herzegovina	1283 (1124 to 1466)	838 (720 to 978)	135 (76 to 164)	−1·6% (−2·1 to −1·1)	−0·9% (−1·4 to −0·4)	−6·9% (−8·6 to −5·9)	0·3% (−0·3 to 0·8)	0·7% (0·0 to 1·2)	−3·6% (−4·8 to −2·0)
	Bulgaria	2372 (2160 to 2581)	1115 (1011 to 1215)	115 (103 to 129)	0·7% (0·1 to 1·2)	0·7% (0·1 to 1·3)	−0·8% (−1·5 to −0·2)	−1·5% (−2·1 to −0·9)	−1·3% (−2·0 to −0·7)	−6·2% (−7·4 to −5·0)
	Croatia	1041 (929 to 1164)	485 (430 to 542)	68 (61 to 75)	−5·7% (−6·1 to −5·3)	−5·7% (−6·1 to −5·4)	−8·1% (−8·7 to −7·4)	−4·1% (−5·0 to −3·3)	−3·9% (−4·8 to −3·1)	−7·6% (−8·6 to −6·6)
	Czech Republic	1248 (1057 to 1471)	606 (508 to 714)	50 (45 to 55)	−3·8% (−4·3 to −3·3)	−3·8% (−4·3 to −3·3)	−6·6% (−7·4 to −5·7)	−1·2% (−1·9 to −0·5)	−1·0% (−1·7 to −0·3)	−6·1% (−7·2 to −5·1)
	Hungary	1720 (1515 to 1966)	819 (717 to 934)	81 (73 to 90)	−5·4% (−5·8 to −4·9)	−5·4% (−5·8 to −5·0)	−9·1% (−9·7 to −8·4)	−3·2% (−4·0 to −2·4)	−3·1% (−3·9 to −2·2)	−8·3% (−9·5 to −7·2)
	Macedonia	523 (455 to 613)	360 (310 to 423)	51 (34 to 113)	−3·3% (−3·7 to −2·9)	−3·3% (−3·8 to −2·9)	−4·1% (−6·0 to −2·1)	−2·7% (−3·6 to −1·7)	−2·4% (−3·3 to −1·4)	−5·9% (−8·0 to −3·8)
	Montenegro	102 (84 to 123)	72 (58 to 88)	4 (3 to 6)	−0·9% (−1·2 to −0·6)	−0·9% (−1·2 to −0·5)	−2·6% (−4·4 to −0·6)	1·3% (0·7 to 1·9)	1·6% (1·0 to 2·2)	−5·2% (−6·6 to −3·9)
	Poland	10 672 (9626 to 11 794)	5141 (4684 to 5633)	586 (528 to 653)	−5·1% (−5·5 to −4·7)	−5·0% (−5·4 to −4·6)	−7·2% (−7·7 to −6·6)	−2·0% (−2·5 to −1·3)	−2·0% (−2·6 to −1·4)	−6·6% (−7·7 to −5·5)
	Romania	19 211 (17 605 to 20 731)	9227 (8597 to 9916)	1020 (915 to 1138)	1·1% (0·6 to 1·5)	1·1% (0·7 to 1·5)	1·2% (0·6 to 1·8)	−3·2% (−3·9 to −2·5)	−3·3% (−3·9 to −2·7)	−6·4% (−7·6 to −5·1)
	Serbia	1862 (1639 to 2128)	1264 (1095 to 1458)	162 (134 to 217)	−1·6% (−2·0 to −1·2)	−1·6% (−1·9 to −1·2)	−2·0% (−3·2 to −0·3)	−0·4% (−0·9 to 0·1)	−0·1% (−0·6 to 0·5)	−5·8% (−7·5 to −3·3)
	Slovakia	826 (651 to 1026)	581 (449 to 733)	32 (26 to 49)	−2·3% (−2·7 to −2·0)	−2·2% (−2·6 to −1·8)	−5·4% (−6·7 to −3·3)	−0·3% (−1·4 to 0·4)	−0·1% (−1·1 to 0·7)	−6·7% (−8·6 to −3·8)
	Slovenia	339 (290 to 394)	163 (136 to 190)	17 (15 to 19)	−4·9% (−5·3 to −4·4)	−4·9% (−5·3 to −4·4)	−7·7% (−8·4 to −7·0)	−1·5% (−2·2 to −0·7)	−1·4% (−2·2 to −0·7)	−5·0% (−6·3 to −3·8)
Eastern Europe		286 284 (260 925 to 307 553)	157 006 (146 643 to 167 092)	16 027 (14 841 to 17 471)	2·5% (2·0 to 2·9)	3·4% (3·0 to 3·8)	5·3% (4·8 to 5·8)	−3·5% (−4·1 to −2·7)	−3·9% (−4·5 to −3·4)	−8·4% (−9·3 to −7·5)
	Belarus	5698 (5047 to 6457)	3979 (3543 to 4508)	461 (251 to 590)	1·3% (0·8 to 1·7)	1·3% (0·8 to 1·7)	2·1% (−4·0 to 4·1)	−1·1% (−1·8 to −0·3)	−0·9% (−1·6 to −0·1)	−5·0% (−6·6 to −3·0)
	Estonia	655 (583 to 722)	331 (299 to 363)	29 (26 to 33)	0·2% (−0·2 to 0·8)	0·9% (0·4 to 1·3)	−0·6% (−1·3 to 0·2)	−5·3% (−6·1 to −4·4)	−6·0% (−6·8 to −5·1)	−10·0% (−11·2 to −8·7)
	Latvia	1234 (1121 to 1362)	599 (545 to 658)	58 (51 to 66)	−0·6% (−1·0 to −0·2)	−0·5% (−0·8 to −0·1)	0·0% (−0·7 to 0·7)	−4·1% (−4·8 to −3·4)	−4·1% (−4·9 to −3·5)	−9·9% (−11·2 to −8·4)
	Lithuania	3133 (2847 to 3442)	1572 (1461 to 1689)	186 (167 to 206)	0·3% (−0·2 to 0·7)	0·5% (0·1 to 0·9)	1·5% (0·9 to 2·2)	−3·1% (−3·9 to −2·4)	−3·0% (−3·7 to −2·5)	−6·7% (−7·9 to −5·6)
	Moldova	3590 (3264 to 3901)	1635 (1489 to 1785)	215 (188 to 244)	2·4% (1·9 to 2·9)	2·1% (1·7 to 2·6)	6·1% (5·3 to 6·9)	−1·7% (−2·5 to −0·9)	−1·6% (−2·4 to −0·8)	−8·5% (−10·1 to −7·0)
	Russia	208 626 (188 822 to 226 139)	116 730 (108 396 to 125 347)	11 020 (10 010 to 12 238)	2·4% (1·8 to 3·0)	3·4% (2·9 to 3·9)	5·4% (4·8 to 6·1)	−3·8% (−4·6 to −2·9)	−4·2% (−4·8 to −3·6)	−8·8% (−9·8 to −7·7)
	Ukraine	63 348 (57 599 to 67 923)	32 160 (30 005 to 34 363)	4058 (3587 to 4606)	3·0% (2·5 to 3·6)	4·1% (3·6 to 4·6)	5·8% (5·1 to 6·5)	−2·7% (−3·6 to −2·0)	−3·6% (−4·3 to −2·9)	−7·9% (−9·1 to −6·5)
Western Europe		46 878 (40 077 to 54 290)	22 728 (19 038 to 26 348)	3617 (3384 to 3859)	−5·0% (−5·3 to −4·6)	−5·0% (−5·4 to −4·6)	−6·1% (−6·4 to −5·8)	−1·5% (−2·2 to −0·9)	−1·5% (−2·3 to −0·8)	−4·6% (−5·1 to −4·0)
	Andorra	14 (11 to 18)	10 (7 to 12)	0 (0 to 1)	−0·2% (−0·5 to 0·1)	−0·1% (−0·4 to 0·2)	−4·1% (−5·5 to −1·9)	1·0% (0·5 to 1·5)	1·1% (0·6 to 1·6)	−2·4% (−4·3 to −0·7)
	Austria	897 (762 to 1043)	430 (363 to 503)	54 (49 to 60)	−4·5% (−5·1 to −4·0)	−4·5% (−5·1 to −4·0)	−8·7% (−9·4 to −8·0)	−1·7% (−2·5 to −1·0)	−1·7% (−2·4 to −0·9)	−2·7% (−3·7 to −1·6)
	Belgium	1168 (1006 to 1348)	557 (472 to 643)	81 (71 to 92)	−2·6% (−3·1 to −2·2)	−2·6% (−3·1 to −2·1)	−4·2% (−4·9 to −3·5)	−1·2% (−1·7 to −0·7)	−1·2% (−1·7 to −0·6)	−4·5% (−5·7 to −3·4)
	Cyprus	76 (60 to 96)	54 (41 to 71)	3 (3 to 5)	−0·3% (−0·6 to 0·1)	−0·1% (−0·4 to 0·4)	−4·8% (−6·5 to −1·8)	0·3% (−0·3 to 0·7)	0·4% (−0·2 to 1·0)	−5·0% (−6·8 to −2·5)
	Denmark	472 (396 to 554)	229 (188 to 269)	31 (27 to 34)	−2·2% (−2·8 to −1·7)	−2·3% (−2·8 to −1·7)	−3·3% (−4·0 to −2·6)	−1·1% (−1·6 to −0·5)	−1·0% (−1·7 to −0·4)	−4·1% (−5·3 to −3·0)
	Finland	552 (468 to 638)	252 (209 to 294)	58 (50 to 66)	−4·2% (−4·7 to −3·7)	−4·2% (−4·7 to −3·7)	−5·9% (−6·8 to −5·1)	−2·0% (−2·5 to −1·5)	−1·8% (−2·4 to −1·3)	−6·0% (−7·4 to −4·6)
	France	7832 (6904 to 8837)	3651 (3151 to 4099)	1061 (901 to 1249)	−8·4% (−9·2 to −7·8)	−8·6% (−9·3 to −7·9)	−5·7% (−6·5 to −4·9)	−2·4% (−3·0 to −1·8)	−2·7% (−3·5 to −2·1)	−4·8% (−6·3 to −3·4)
	Germany	6525 (5417 to 7677)	3112 (2523 to 3710)	421 (372 to 472)	−5·9% (−6·5 to −5·4)	−5·9% (−6·5 to −5·3)	−9·7% (−10·4 to −9·0)	−0·8% (−1·5 to −0·1)	−0·7% (−1·5 to 0·1)	−4·7% (−5·8 to −3·5)
	Greece	1112 (966 to 1253)	494 (423 to 561)	173 (147 to 200)	−4·4% (−4·8 to −4·0)	−4·3% (−4·8 to −3·9)	−5·9% (−6·7 to −5·2)	−0·3% (−0·8 to 0·2)	−0·3% (−0·8 to 0·2)	0·3% (−1·2 to 1·7)
	Iceland	67 (53 to 83)	33 (26 to 41)	1 (1 to 2)	−1·6% (−2·0 to −1·2)	−1·5% (−1·9 to −1·1)	−6·0% (−7·1 to −5·0)	1·3% (0·7 to 1·9)	1·3% (0·7 to 2·0)	−5·0% (−6·5 to −3·5)
	Ireland	514 (436 to 600)	251 (211 to 296)	28 (25 to 31)	−3·0% (−3·5 to −2·5)	−3·2% (−3·8 to −2·7)	−5·1% (−5·9 to −4·4)	−2·3% (−3·0 to −1·6)	−2·3% (−3·0 to −1·5)	−4·6% (−5·9 to −3·4)
	Israel	769 (633 to 915)	374 (302 to 448)	29 (26 to 34)	−3·0% (−3·5 to −2·6)	−3·0% (−3·5 to −2·5)	−3·5% (−4·3 to −2·7)	0·7% (0·1 to 1·3)	1·0% (0·3 to 1·6)	−7·6% (−8·9 to −6·3)
	Italy	4825 (4078 to 5624)	2272 (1893 to 2671)	469 (410 to 537)	−4·3% (−4·8 to −3·9)	−4·4% (−4·9 to −3·9)	−5·6% (−6·3 to −4·9)	−2·0% (−2·7 to −1·4)	−1·9% (−2·7 to −1·2)	−3·8% (−4·9 to −2·6)
	Luxembourg	74 (57 to 94)	37 (28 to 47)	1 (1 to 2)	−1·4% (−1·8 to −1·0)	−1·4% (−1·9 to −1·0)	−6·8% (−7·5 to −6·0)	0·7% (−0·2 to 1·5)	0·7% (−0·2 to 1·6)	−5·7% (−6·9 to −4·6)
	Malta	61 (48 to 76)	30 (23 to 37)	1 (1 to 1)	0·2% (−0·2 to 0·5)	0·1% (−0·3 to 0·5)	−6·1% (−7·0 to −5·3)	2·3% (1·8 to 2·8)	2·3% (1·7 to 2·9)	−4·7% (−5·8 to −3·5)
	Netherlands	1260 (1066 to 1464)	609 (510 to 717)	75 (66 to 87)	−4·5% (−5·0 to −4·0)	−4·6% (−5·2 to −4·1)	−5·5% (−6·3 to −4·7)	−1·0% (−1·5 to −0·5)	−0·9% (−1·4 to −0·4)	−5·6% (−7·0 to −4·3)
	Norway	544 (456 to 633)	263 (218 to 309)	34 (30 to 40)	−2·2% (−2·7 to −1·7)	−2·2% (−2·7 to −1·7)	−3·8% (−4·7 to −3·0)	−0·5% (−0·9 to −0·0)	−0·3% (−0·9 to 0·2)	−5·0% (−6·4 to −3·5)
	Portugal	2893 (2631 to 3165)	1380 (1261 to 1517)	222 (200 to 246)	−3·2% (−3·8 to −2·7)	−3·0% (−3·4 to −2·5)	−4·9% (−5·5 to −4·2)	−4·5% (−5·3 to −3·6)	−5·3% (−6·1 to −4·5)	−5·0% (−6·0 to −4·0)
	Spain	6202 (5536 to 7055)	2943 (2588 to 3372)	408 (363 to 464)	−5·6% (−6·3 to −5·0)	−6·1% (−6·8 to −5·4)	−6·5% (−7·1 to −5·9)	−4·1% (−4·9 to −3·4)	−4·1% (−4·9 to −3·4)	−6·1% (−7·2 to −4·8)
	Sweden	965 (744 to 1207)	501 (374 to 627)	70 (61 to 82)	−2·8% (−3·4 to −2·3)	−2·4% (−3·1 to −1·9)	−5·9% (−6·7 to −5·1)	0·7% (0·2 to 1·2)	1·2% (0·6 to 1·9)	−4·2% (−5·6 to −2·8)
	Switzerland	727 (609 to 850)	355 (293 to 415)	34 (29 to 39)	−3·9% (−4·4 to −3·4)	−3·7% (−4·3 to −3·3)	−6·8% (−7·6 to −5·9)	0·2% (−0·4 to 0·7)	0·2% (−0·3 to 0·9)	−4·7% (−6·1 to −3·3)
	UK	9283 (7237 to 11 710)	4869 (3734 to 6046)	359 (338 to 381)	−0·7% (−1·6 to 0·3)	−0·6% (−1·5 to 0·5)	−3·9% (−4·2 to −3·7)	0·9% (−0·7 to 2·5)	1·1% (−0·5 to 2·6)	−4·2% (−4·8 to −3·6)
Andean Latin America		40 363 (35 713 to 46 150)	27 295 (24 074 to 31 246)	3708 (2955 to 6433)	−7·0% (−7·2 to −6·6)	−7·2% (−7·5 to −6·9)	−8·3% (−10·2 to −2·9)	−2·3% (−2·8 to −1·6)	−2·0% (−2·7 to −1·4)	−4·8% (−5·9 to −3·8)
	Bolivia	6760 (6002 to 7591)	4353 (3868 to 4921)	885 (625 to 1148)	−4·6% (−4·9 to −4·2)	−4·8% (−5·1 to −4·5)	−5·3% (−7·0 to −2·9)	−1·5% (−2·2 to −0·9)	−1·3% (−1·9 to −0·6)	−4·0% (−5·7 to −2·3)
	Ecuador	8272 (7660 to 9285)	5764 (5262 to 6405)	883 (680 to 1564)	−5·6% (−6·1 to −5·1)	−5·7% (−6·1 to −5·4)	−6·0% (−7·5 to −2·2)	−3·4% (−3·9 to −2·7)	−3·2% (−3·8 to −2·5)	−5·4% (−7·2 to −3·5)
	Peru	25 331 (21 789 to 29 649)	17 178 (14 802 to 20 201)	1941 (1376 to 3906)	−7·8% (−8·2 to −7·3)	−8·1% (−8·5 to −7·6)	−10·1% (−12·9 to −2·6)	−2·0% (−2·8 to −1·2)	−1·8% (−2·5 to −0·9)	−4·8% (−6·4 to −3·2)
Central Latin America		63 258 (58 480 to 68 188)	30 872 (28 304 to 33 419)	5399 (5082 to 5863)	−4·6% (−4·8 to −4·4)	−4·5% (−4·7 to −4·3)	−7·2% (−7·6 to −6·8)	−2·3% (−2·7 to −1·9)	−2·2% (−2·6 to −1·9)	−4·6% (−5·1 to −4·1)
	Colombia	13 086 (11 903 to 14 299)	5968 (5403 to 6550)	860 (789 to 941)	−3·6% (−3·9 to −3·3)	−3·6% (−3·9 to −3·4)	−4·3% (−4·8 to −3·8)	−2·2% (−2·7 to −1·8)	−2·1% (−2·7 to −1·6)	−5·8% (−6·9 to −4·7)
	Costa Rica	764 (671 to 865)	355 (309 to 401)	49 (44 to 55)	−4·4% (−4·7 to −4·1)	−4·1% (−4·5 to −3·8)	−6·8% (−7·5 to −6·0)	−1·6% (−2·2 to −1·0)	−1·4% (−2·0 to −0·8)	−6·4% (−7·6 to −5·2)
	El Salvador	1169 (990 to 1391)	757 (629 to 925)	100 (78 to 178)	−5·3% (−5·8 to −4·8)	−4·9% (−5·4 to −4·4)	−9·2% (−11·5 to −3·3)	0·1% (−0·5 to 0·8)	0·4% (−0·3 to 1·1)	−3·6% (−5·3 to −2·2)
	Guatemala	5022 (4366 to 5789)	2309 (1999 to 2653)	334 (285 to 388)	−9·1% (−9·5 to −8·7)	−9·1% (−9·5 to −8·7)	−10·8% (−11·3 to −10·3)	−3·9% (−4·7 to −3·1)	−3·9% (−4·7 to −3·1)	−5·9% (−7·5 to −4·3)
	Honduras	3029 (2730 to 3349)	1873 (1675 to 2079)	449 (267 to 706)	−1·9% (−2·2 to −1·6)	−1·9% (−2·2 to −1·7)	−2·5% (−5·5 to −0·3)	−1·6% (−2·1 to −1·0)	−1·5% (−2·0 to −0·9)	−3·6% (−6·0 to −1·8)
	Mexico	28 125 (25 747 to 30 826)	13 231 (11 828 to 14 711)	2597 (2474 to 2736)	−4·3% (−4·7 to −4·0)	−4·2% (−4·6 to −3·9)	−8·6% (−9·0 to −8·2)	−2·6% (−3·3 to −1·7)	−2·6% (−3·3 to −2·0)	−4·2% (−4·8 to −3·7)
	Nicaragua	1424 (1230 to 1658)	908 (777 to 1063)	184 (146 to 299)	−4·6% (−5·0 to −4·3)	−4·9% (−5·2 to −4·5)	−5·2% (−6·7 to −1·6)	−1·8% (−2·6 to −1·1)	−1·6% (−2·3 to −0·8)	−4·1% (−6·0 to −2·0)
	Panama	2101 (1930 to 2291)	1424 (1312 to 1547)	199 (158 to 299)	−2·1% (−2·4 to −1·7)	−1·9% (−2·2 to −1·6)	−3·6% (−4·5 to −2·6)	−0·7% (−1·3 to −0·2)	−0·4% (−0·9 to 0·1)	−4·0% (−6·2 to −2·4)
	Venezuela	8540 (7812 to 9332)	4047 (3687 to 4441)	627 (537 to 730)	−4·6% (−5·0 to −4·3)	−4·5% (−4·8 to −4·1)	−5·3% (−6·0 to −4·6)	−2·3% (−2·8 to −1·8)	−2·2% (−2·7 to −1·7)	−4·9% (−6·7 to −3·2)
Southern Latin America		15 552 (14 194 to 16 928)	8533 (7827 to 9256)	1168 (1084 to 1275)	−3·8% (−4·2 to −3·3)	−2·8% (−3·2 to −2·5)	−5·6% (−6·0 to −5·2)	−1·5% (−2·1 to −1·0)	−1·8% (−2·3 to −1·3)	−4·5% (−5·3 to −3·6)
	Argentina	10 091 (9166 to 11 101)	5603 (5129 to 6093)	604 (550 to 667)	−3·8% (−4·5 to −3·2)	−2·8% (−3·2 to −2·3)	−5·8% (−6·4 to −5·3)	−1·6% (−2·4 to −0·9)	−2·0% (−2·6 to −1·4)	−4·7% (−5·7 to −3·5)
	Chile	4763 (4315 to 5187)	2598 (2381 to 2834)	519 (464 to 583)	−4·2% (−4·6 to −3·8)	−3·3% (−3·6 to −3·0)	−5·5% (−6·1 to −4·9)	−1·6% (−2·2 to −0·9)	−1·6% (−2·1 to −1·1)	−4·9% (−6·0 to −3·6)
	Uruguay	697 (625 to 769)	332 (300 to 367)	46 (41 to 52)	−3·6% (−4·0 to −3·3)	−3·3% (−3·7 to −3·0)	−5·6% (−6·3 to −4·9)	−0·8% (−1·5 to −0·1)	−1·1% (−1·7 to −0·4)	−5·4% (−6·8 to −3·8)
Tropical Latin America		77 036 (67 778 to 85 333)	55 933 (48 521 to 63 010)	6016 (4167 to 7739)	−1·3% (−1·7 to −0·9)	−1·4% (−1·8 to −1·0)	−3·9% (−5·4 to −2·9)	−1·6% (−2·0 to −1·2)	−1·4% (−1·8 to −1·0)	−4·3% (−5·3 to −2·9)
	Brazil	74 539 (65 551 to 82 668)	54 219 (46 970 to 61 179)	5763 (3947 to 7379)	−1·3% (−1·7 to −0·9)	−1·4% (−1·8 to −1·0)	−4·0% (−5·5 to −3·0)	−1·6% (−2·0 to −1·2)	−1·5% (−1·9 to −1·0)	−4·3% (−5·4 to −2·9)
	Paraguay	2497 (2257 to 2748)	1714 (1549 to 1895)	254 (197 to 391)	−0·9% (−1·2 to −0·6)	−0·9% (−1·1 to −0·6)	−1·8% (−3·2 to −0·1)	−0·7% (−1·2 to −0·2)	−0·5% (−1·0 to 0·0)	−2·8% (−5·0 to −0·9)
North Africa and the Middle East		192 790 (169 116 to 220 039)	142 092 (124 640 to 161 664)	19 066 (15 259 to 24 487)	−2·9% (−3·1 to −2·8)	−3·1% (−3·2 to −3·0)	−3·4% (−4·4 to −2·0)	−1·0% (−1·3 to −0·6)	−0·9% (−1·3 to −0·5)	−3·5% (−4·6 to −2·4)
	Algeria	20 449 (17 949 to 22 992)	14 760 (13 011 to 16 780)	1824 (1384 to 2348)	−3·4% (−3·8 to −3·0)	−3·6% (−3·9 to −3·3)	−4·0% (−5·4 to −2·1)	−0·9% (−1·6 to −0·3)	−0·9% (−1·5 to −0·3)	−2·4% (−4·3 to −0·7)
	Bahrain	351 (289 to 427)	263 (216 to 325)	10 (8 to 14)	−3·1% (−3·5 to −2·7)	−3·2% (−3·6 to −2·8)	−4·1% (−5·4 to −2·4)	−3·1% (−3·9 to −2·2)	−3·0% (−3·9 to −2·1)	−5·7% (−8·5 to −2·6)
	Egypt	13 844 (11 588 to 16 646)	10 354 (8582 to 12 661)	884 (685 to 1649)	−3·7% (−4·1 to −3·3)	−3·9% (−4·4 to −3·5)	−5·6% (−6·8 to −3·0)	−1·5% (−2·2 to −0·7)	−1·3% (−2·1 to −0·5)	−4·3% (−6·5 to −1·3)
	Iran	19 347 (16 519 to 22 484)	14 455 (12 372 to 17 023)	1603 (964 to 2057)	−2·3% (−2·7 to −2·0)	−2·2% (−2·5 to −1·9)	−3·8% (−7·8 to −0·8)	0·2% (−0·3 to 0·7)	0·4% (−0·1 to 0·9)	−2·4% (−5·2 to 0·2)
	Iraq	17 193 (15 134 to 19 607)	13 016 (11 522 to 14 670)	1319 (963 to 1912)	−3·1% (−3·5 to −2·7)	−3·1% (−3·4 to −2·8)	−2·3% (−4·8 to −0·1)	−1·4% (−2·1 to −0·7)	−1·6% (−2·2 to −1·0)	−3·7% (−5·8 to −1·8)
	Jordan	678 (496 to 912)	526 (381 to 731)	20 (12 to 25)	−2·2% (−2·8 to −1·8)	−2·1% (−2·7 to −1·6)	−4·5% (−6·4 to −2·5)	0·7% (−0·5 to 1·6)	1·0% (−0·3 to 2·1)	−6·0% (−8·0 to −3·8)
	Kuwait	1059 (895 to 1255)	553 (466 to 661)	19 (16 to 22)	−3·7% (−4·1 to −3·3)	−3·6% (−3·9 to −3·3)	−4·4% (−5·6 to −3·1)	−4·3% (−4·9 to −3·7)	−4·5% (−5·0 to −3·9)	−7·2% (−9·2 to −5·4)
	Lebanon	1002 (830 to 1215)	754 (616 to 930)	56 (34 to 87)	−4·2% (−4·7 to −3·6)	−4·2% (−4·7 to −3·6)	−5·9% (−8·7 to −2·5)	0·8% (−0·1 to 1·7)	1·0% (0·0 to 1·9)	−4·2% (−6·8 to −1·8)
	Libya	1538 (1290 to 1835)	1137 (953 to 1353)	88 (69 to 116)	−2·6% (−2·9 to −2·2)	−2·7% (−3·0 to −2·3)	−3·4% (−6·1 to −0·3)	−0·6% (−1·1 to −0·1)	−0·5% (−1·0 to 0·0)	−1·7% (−3·5 to 0·4)
	Morocco	25 241 (22 485 to 28 011)	18 172 (16 500 to 19 897)	3186 (2261 to 4723)	−3·1% (−3·5 to −2·7)	−3·3% (−3·6 to −3·0)	−3·8% (−5·6 to −2·0)	−1·3% (−1·9 to −0·7)	−1·1% (−1·7 to −0·6)	−3·2% (−5·8 to −1·0)
	Oman	964 (725 to 1239)	725 (544 to 946)	29 (19 to 37)	−2·9% (−3·3 to −2·5)	−2·9% (−3·3 to −2·5)	−5·2% (−9·1 to −1·3)	−0·6% (−1·6 to 0·2)	−0·5% (−1·6 to 0·3)	−1·4% (−4·1 to 1·0)
	Palestine	1077 (794 to 1427)	832 (596 to 1118)	3 (2 to 4)	0·8% (0·3 to 1·2)	0·8% (0·3 to 1·3)	−3·6% (−6·0 to −0·8)	3·1% (2·5 to 3·6)	3·2% (2·6 to 3·8)	−1·1% (−4·0 to 2·2)
	Qatar	859 (687 to 1051)	635 (499 to 785)	6 (5 to 9)	−0·0% (−0·6 to 0·5)	−0·1% (−0·6 to 0·5)	−3·0% (−5·1 to −1·0)	−0·3% (−1·2 to 0·6)	−0·2% (−1·0 to 0·7)	−3·9% (−7·3 to −0·5)
	Saudi Arabia	8646 (6989 to 10 492)	7128 (5759 to 8728)	575 (377 to 678)	−4·1% (−4·5 to −3·8)	−4·0% (−4·4 to −3·7)	−5·0% (−7·0 to −2·3)	−2·0% (−2·5 to −1·4)	−1·8% (−2·4 to −1·1)	−3·5% (−4·9 to −2·0)
	Sudan	17 189 (15 233 to 19 400)	12 320 (10 946 to 13 912)	1875 (1169 to 2842)	−1·2% (−1·6 to −0·9)	−1·3% (−1·6 to −1·0)	−2·8% (−5·3 to −0·5)	−2·1% (−2·6 to −1·7)	−1·9% (−2·4 to −1·4)	−4·3% (−7·2 to −1·0)
	Syria	2972 (2363 to 3713)	2245 (1748 to 2854)	60 (38 to 148)	0·3% (−0·1 to 0·7)	0·4% (−0·0 to 0·9)	−4·6% (−6·8 to −2·3)	2·5% (1·9 to 3·2)	2·8% (2·1 to 3·5)	−3·0% (−5·1 to −1·4)
	Tunisia	3390 (3053 to 3817)	2552 (2270 to 2906)	306 (218 to 511)	−3·7% (−4·2 to −3·2)	−3·5% (−4·0 to −3·1)	−5·9% (−7·8 to −3·6)	−0·0% (−0·7 to 0·7)	−0·0% (−0·6 to 0·8)	−4·1% (−6·7 to −2·0)
	Turkey	17 389 (14 689 to 20 558)	12 812 (10 612 to 15 268)	877 (685 to 1686)	−4·8% (−5·2 to −4·4)	−4·9% (−5·4 to −4·5)	−8·2% (−11·1 to −4·0)	−1·0% (−1·8 to −0·4)	−1·1% (−1·9 to −0·3)	−6·3% (−8·0 to −4·6)
	United Arab Emirates	2197 (1612 to 2792)	1656 (1196 to 2153)	62 (24 to 110)	−4·3% (−4·8 to −3·8)	−4·3% (−4·9 to −3·8)	−4·9% (−7·9 to −1·3)	−1·3% (−2·1 to −0·6)	−1·2% (−2·1 to −0·5)	−2·1% (−5·3 to 2·9)
	Yemen	12 690 (11 269 to 14 335)	9382 (8313 to 10 563)	1710 (790 to 3320)	−2·5% (−2·8 to −2·1)	−2·6% (−2·9 to −2·3)	−3·1% (−7·2 to 1·6)	−1·9% (−2·4 to −1·3)	−2·1% (−2·7 to −1·6)	−2·8% (−6·3 to 0·9)
High-income North America		14 544 (12 271 to 17 008)	7422 (6067 to 8701)	978 (929 to 1025)	−5·6% (−6·3 to −4·8)	−5·5% (−6·2 to −4·8)	−7·1% (−7·4 to −6·8)	−2·0% (−2·5 to −1·6)	−1·9% (−2·5 to −1·5)	−3·3% (−3·8 to −2·8)
	Canada	1994 (1687 to 2360)	954 (793 to 1134)	123 (108 to 142)	−2·3% (−2·8 to −1·8)	−2·3% (−2·9 to −1·8)	−5·1% (−5·9 to −4·4)	−0·3% (−0·7 to 0·2)	−0·2% (−0·8 to 0·3)	−3·8% (−5·1 to −2·4)
	Greenland	29 (23 to 37)	16 (12 to 20)	2 (0 to 2)	−2·8% (−3·5 to −2·1)	−2·3% (−3·0 to −1·4)	−5·0% (−6·2 to −2·2)	8·0% (6·4 to 9·5)	8·5% (6·7 to 10·4)	−4·2% (−6·0 to −2·4)
	USA	12 516 (10 573 to 14 627)	6450 (5241 to 7583)	853 (807 to 896)	−5·9% (−6·6 to −5·0)	−5·8% (−6·5 to −5·0)	−7·3% (−7·7 to −7·0)	−2·3% (−2·9 to −1·9)	−2·1% (−2·8 to −1·7)	−3·2% (−3·8 to −2·8)
Oceania		6512 (5761 to 7443)	6372 (5700 to 7176)	683 (432 to 1033)	−1·4% (−1·6 to −1·1)	−1·5% (−1·7 to −1·3)	−2·7% (−4·4 to −0·9)	0·1% (−0·4 to 0·7)	0·4% (−0·1 to 0·9)	−3·2% (−5·3 to −1·0)
	American Samoa	46 (36 to 57)	38 (29 to 48)	1 (0 to 1)	−0·9% (−1·5 to −0·4)	−0·9% (−1·7 to −0·3)	−5·3% (−7·1 to −2·7)	1·8% (0·7 to 2·6)	2·1% (0·8 to 3·0)	−1·4% (−3·6 to 0·9)
	Fiji	339 (299 to 388)	363 (322 to 408)	36 (28 to 48)	−2·5% (−2·9 to −2·2)	−2·4% (−2·7 to −2·1)	−3·3% (−5·0 to −1·7)	−0·7% (−1·4 to −0·1)	−0·7% (−1·2 to −0·1)	−2·9% (−4·8 to −1·0)
	Guam	83 (70 to 102)	87 (74 to 105)	4 (3 to 6)	−2·3% (−2·7 to −2·0)	−2·4% (−2·8 to −2·0)	−4·8% (−6·4 to −2·4)	1·5% (0·7 to 2·1)	1·6% (1·0 to 2·2)	−0·7% (−2·6 to 1·3)
	Kiribati	178 (160 to 201)	178 (161 to 196)	45 (34 to 71)	−2·6% (−3·0 to −2·2)	−2·7% (−3·0 to −2·4)	−2·8% (−4·2 to −1·3)	−1·5% (−2·1 to −1·0)	−1·4% (−1·9 to −0·9)	−2·3% (−4·2 to −0·3)
	Marshall Islands	36 (31 to 42)	35 (30 to 41)	3 (2 to 5)	−2·8% (−3·1 to −2·5)	−2·9% (−3·1 to −2·6)	−4·3% (−6·0 to −2·1)	−0·0% (−0·8 to 0·7)	0·2% (−0·5 to 0·8)	−4·5% (−6·2 to −2·6)
	Federated States of Micronesia	52 (44 to 62)	51 (43 to 61)	4 (2 to 9)	−2·5% (−2·9 to −2·2)	−2·6% (−2·9 to −2·3)	−5·4% (−8·2 to −2·3)	0·8% (0·1 to 1·4)	1·1% (0·4 to 1·8)	−3·3% (−6·3 to −0·1)
	Northern Mariana Islands	79 (61 to 102)	68 (51 to 90)	1 (1 to 1)	−1·1% (−1·7 to −0·4)	−1·4% (−2·0 to −0·8)	−5·3% (−6·8 to −3·2)	0·5% (−0·4 to 1·4)	0·8% (−0·2 to 1·7)	−0·2% (−2·2 to 3·3)
	Papua New Guinea	4751 (4190 to 5455)	4601 (4100 to 5216)	431 (231 to 711)	−0·8% (−1·1 to −0·4)	−0·9% (−1·2 to −0·7)	−2·0% (−4·4 to 0·5)	0·3% (−0·4 to 1·0)	0·6% (0·1 to 1·3)	−3·3% (−6·3 to −0·0)
	Samoa	52 (44 to 63)	56 (47 to 67)	5 (3 to 10)	−3·2% (−3·5 to −2·9)	−3·2% (−3·5 to −2·9)	−5·5% (−7·5 to −3·0)	−0·4% (−1·1 to 0·4)	−0·1% (−0·8 to 0·6)	−3·9% (−6·2 to −1·6)
	Solomon Islands	302 (270 to 338)	301 (272 to 333)	57 (26 to 92)	−3·7% (−4·0 to −3·4)	−3·8% (−4·2 to −3·6)	−4·3% (−6·8 to −1·1)	−1·7% (−2·2 to −1·1)	−1·4% (−1·9 to −0·9)	−4·1% (−6·8 to −1·2)
	Tonga	33 (26 to 42)	34 (27 to 44)	2 (1 to 2)	−1·2% (−1·6 to −0·9)	−1·1% (−1·4 to −0·8)	−2·8% (−4·7 to −0·9)	2·6% (2·0 to 3·3)	2·9% (2·3 to 3·5)	−3·0% (−5·5 to −0·3)
	Vanuatu	116 (104 to 131)	120 (109 to 133)	21 (11 to 34)	−3·4% (−3·8 to −3·1)	−3·4% (−3·7 to −3·2)	−3·9% (−6·5 to −0·7)	−1·9% (−2·4 to −1·3)	−1·7% (−2·2 to −1·2)	−4·2% (−7·1 to −1·4)
Central sub-Saharan Africa		220 347 (195 416 to 248 336)	192 120 (170 331 to 214 480)	46 546 (23 827 to 94 213)	−0·7% (−0·9 to −0·4)	−1·3% (−1·6 to −1·0)	−0·3% (−3·6 to 3·1)	−1·1% (−1·5 to −0·7)	−0·9% (−1·3 to −0·5)	−2·6% (−6·6 to 0·7)
	Angola	55 064 (48 779 to 61 978)	50 316 (45 437 to 55 429)	6923 (2261 to 18 801)	−2·0% (−2·4 to −1·6)	−2·2% (−2·4 to −1·9)	−3·4% (−10·7 to 3·4)	−1·2% (−1·7 to −0·6)	−1·1% (−1·6 to −0·7)	−4·1% (−12·9 to 4·1)
	Central African Republic	12 519 (11 321 to 14 127)	11 235 (10 220 to 12 329)	4129 (1637 to 8068)	−0·3% (−0·6 to 0·1)	−0·4% (−0·7 to −0·2)	−0·3% (−4·9 to 3·0)	−1·6% (−2·2 to −1·0)	−1·9% (−2·4 to −1·3)	−0·8% (−6·5 to 4·4)
	Congo	7019 (6262 to 7910)	6321 (5755 to 6945)	943 (483 to 1631)	−3·0% (−3·4 to −2·5)	−3·1% (−3·4 to −2·8)	−3·7% (−6·2 to −0·9)	−1·5% (−2·3 to −0·9)	−1·8% (−2·3 to −1·3)	−2·7% (−7·7 to 2·4)
	Democratic Republic of the Congo	139 980 (122 762 to 158 224)	119 059 (102 942 to 136 249)	34 198 (16 261 to 81 194)	−0·0% (−0·4 to 0·3)	−0·9% (−1·3 to −0·5)	0·9% (−3·0 to 4·7)	−0·9% (−1·4 to −0·5)	−0·6% (−1·1 to −0·1)	−2·6% (−7·1 to 1·5)
	Equatorial Guinea	1970 (1735 to 2240)	1876 (1690 to 2086)	125 (37 to 359)	0·8% (0·3 to 1·3)	2·1% (1·8 to 2·5)	−9·0% (−17·7 to −0·9)	−1·6% (−2·3 to −0·9)	−2·1% (−2·6 to −1·6)	−4·4% (−13·2 to 4·0)
	Gabon	3796 (3356 to 4289)	3312 (3003 to 3663)	230 (99 to 438)	−1·2% (−1·6 to −0·8)	−1·3% (−1·6 to −1·0)	−3·9% (−6·8 to −1·5)	−2·2% (−2·8 to −1·6)	−2·4% (−2·9 to −1·9)	−3·6% (−8·5 to 1·5)
Eastern sub-Saharan Africa		599 195 (538 430 to 673 508)	533 816 (490 150 to 585 824)	113 498 (78 976 to 152 204)	−1·0% (−1·2 to −0·7)	−1·2% (−1·4 to −1·0)	−2·0% (−3·7 to −0·6)	−1·5% (−1·9 to −1·0)	−1·0% (−1·4 to −0·7)	−3·1% (−5·9 to −0·6)
	Burundi	14 732 (13 339 to 16 450)	13 594 (12 413 to 14 885)	4694 (2346 to 8125)	−1·8% (−2·2 to −1·3)	−2·1% (−2·6 to −1·7)	−1·0% (−5·5 to 4·3)	−2·5% (−3·1 to −1·9)	−2·6% (−3·2 to −2·1)	−1·7% (−7·6 to 3·0)
	Comoros	638 (563 to 735)	598 (533 to 673)	198 (103 to 363)	−2·3% (−2·7 to −2·0)	−2·5% (−2·8 to −2·2)	−1·6% (−5·8 to 3·2)	−1·3% (−1·9 to −0·7)	−1·5% (−2·1 to −0·9)	−0·5% (−5·7 to 4·2)
	Djibouti	1575 (1401 to 1775)	1355 (1240 to 1483)	261 (111 to 541)	0·5% (0·0 to 0·9)	0·2% (−0·2 to 0·6)	1·9% (−4·5 to 8·4)	−0·4% (−1·0 to 0·2)	−0·3% (−0·8 to 0·2)	−1·8% (−9·9 to 6·9)
	Eritrea	6879 (6127 to 7724)	6264 (5709 to 6946)	2040 (898 to 3920)	−0·9% (−1·4 to −0·6)	−0·6% (−0·9 to −0·2)	−1·4% (−6·6 to 2·9)	−0·8% (−1·3 to −0·2)	−1·2% (−1·7 to −0·7)	0·8% (−4·7 to 6·0)
	Ethiopia	177 354 (159 606 to 201 221)	169 386 (156 217 to 185 645)	43 753 (25 040 to 71 185)	−2·4% (−2·9 to −1·9)	−2·7% (−3·0 to −2·4)	−3·5% (−5·5 to −1·1)	−2·7% (−3·4 to −2·1)	−1·9% (−2·4 to −1·4)	−4·9% (−10·3 to 0·2)
	Kenya	47 552 (38 886 to 58 101)	42 304 (35 125 to 50 814)	7354 (5180 to 9749)	0·7% (0·3 to 1·2)	0·4% (0·0 to 0·8)	0·2% (−2·7 to 3·0)	−1·7% (−2·6 to −0·9)	−1·3% (−2·0 to −0·6)	−1·5% (−3·3 to 0·4)
	Madagascar	25 977 (22 715 to 30 006)	21 978 (19 273 to 25 014)	3948 (1946 to 7173)	−1·5% (−2·1 to −1·0)	−2·0% (−2·3 to −1·6)	−2·0% (−4·8 to 0·2)	−0·4% (−1·2 to 0·3)	−0·5% (−1·2 to 0·1)	−1·8% (−7·3 to 3·0)
	Malawi	25 886 (22 730 to 29 346)	24 411 (21 585 to 27 349)	4100 (2182 to 7729)	0·9% (0·3 to 1·5)	1·0% (0·4 to 1·6)	0·1% (−3·7 to 3·2)	−1·6% (−2·7 to −0·6)	−1·3% (−2·4 to −0·4)	−2·8% (−8·4 to 3·1)
	Mauritius	516 (437 to 593)	267 (233 to 303)	13 (11 to 14)	−3·1% (−3·6 to −2·5)	−3·2% (−3·7 to −2·8)	−6·8% (−7·5 to −6·1)	1·3% (0·7 to 1·8)	1·3% (0·8 to 1·9)	−3·0% (−4·4 to −1·8)
	Mozambique	57 075 (50 079 to 65 407)	51 803 (46 510 to 57 304)	9785 (4266 to 18 071)	0·4% (−0·2 to 1·0)	0·7% (0·1 to 1·4)	−1·3% (−5·8 to 3·2)	0·4% (−0·4 to 1·2)	1·1% (0·4 to 1·7)	−2·0% (−8·9 to 3·9)
	Rwanda	10 763 (9587 to 12 164)	10 329 (9342 to 11 419)	2621 (1325 to 4956)	−2·2% (−2·6 to −1·8)	−2·4% (−2·8 to −2·1)	−2·5% (−6·1 to 0·5)	−2·8% (−3·4 to −2·2)	−2·4% (−3·0 to −1·9)	−2·5% (−7·7 to 2·7)
	Seychelles	41 (33 to 50)	39 (32 to 47)	1 (1 to 2)	−2·4% (−2·8 to −1·8)	−2·8% (−3·2 to −2·3)	−6·6% (−8·4 to −2·8)	1·6% (0·9 to 2·3)	1·5% (0·8 to 2·3)	−3·9% (−5·7 to −1·9)
	Somalia	21 060 (19 111 to 23 536)	18 017 (16 459 to 19 720)	7178 (2373 to 17 821)	1·1% (0·7 to 1·5)	0·7% (0·3 to 1·0)	2·2% (−4·7 to 9·8)	−0·6% (−1·0 to −0·2)	−0·6% (−1·0 to −0·2)	−0·3% (−7·2 to 8·6)
	South Sudan	30 653 (27 245 to 34 496)	30 060 (27 323 to 33 198)	5595 (1760 to 15 286)	0·5% (−0·1 to 0·9)	0·5% (0·1 to 0·8)	0·5% (−7·1 to 8·6)	−1·4% (−2·1 to −0·7)	−1·3% (−1·7 to −0·8)	−0·1% (−8·7 to 9·6)
	Tanzania	90 627 (82 098 to 99 748)	61 366 (56 749 to 66 456)	10 413 (5319 to 19 682)	−0·7% (−1·2 to −0·3)	−0·8% (−1·2 to −0·4)	−1·0% (−4·6 to 2·2)	−0·0% (−0·6 to 0·6)	0·3% (−0·2 to 0·8)	−1·4% (−7·2 to 4·1)
	Uganda	54 587 (48 594 to 63 087)	51 407 (47 136 to 56 660)	7903 (3992 to 13 599)	0·7% (0·2 to 1·2)	0·9% (0·4 to 1·3)	0·2% (−3·3 to 4·8)	−1·0% (−1·8 to −0·3)	−0·7% (−1·4 to −0·1)	−3·1% (−9·3 to 2·0)
	Zambia	33 457 (29 071 to 38 709)	30 607 (27 611 to 34 076)	3573 (1559 to 6195)	2·3% (1·8 to 2·9)	2·1% (1·6 to 2·5)	3·3% (−1·4 to 7·2)	−1·1% (−2·2 to −0·0)	−1·2% (−2·1 to −0·3)	−3·3% (−8·7 to 0·9)
Southern sub-Saharan Africa		617 593 (518 343 to 755 364)	540 901 (454 626 to 633 264)	37 421 (26 317 to 45 960)	2·6% (1·8 to 3·3)	2·5% (2·0 to 3·1)	0·3% (−2·7 to 2·3)	−0·7% (−1·5 to 0·1)	−0·5% (−1·1 to 0·1)	−3·7% (−5·5 to −1·7)
	Botswana	24 784 (21 404 to 28 535)	23 923 (21 143 to 26 889)	1499 (318 to 4967)	1·8% (1·1 to 2·5)	2·1% (1·6 to 2·6)	−0·2% (−9·7 to 7·7)	−0·7% (−1·9 to 0·2)	−0·9% (−1·7 to −0·2)	−4·3% (−14·7 to 8·0)
	Lesotho	17 432 (14 903 to 20 341)	17 132 (14 991 to 19 793)	2329 (989 to 4330)	1·2% (0·4 to 2·0)	1·4% (0·7 to 2·2)	1·4% (−2·4 to 4·9)	0·6% (−0·4 to 1·8)	1·2% (0·3 to 2·0)	−2·5% (−8·8 to 2·7)
	Namibia	17 911 (15 677 to 20 427)	17 406 (15 683 to 19 334)	1157 (536 to 2079)	0·5% (−0·2 to 1·2)	0·5% (−0·0 to 1·0)	−0·9% (−5·2 to 2·0)	−2·1% (−3·1 to −1·0)	−2·0% (−2·7 to −1·3)	−6·1% (−11·7 to −0·8)
	South Africa	483 516 (394 984 to 614 305)	407 918 (327 854 to 493 337)	25 313 (17 771 to 30 925)	2·7% (1·7 to 3·6)	2·5% (1·9 to 3·2)	−0·1% (−3·1 to 1·8)	−0·5% (−1·5 to 0·5)	−0·2% (−1·0 to 0·5)	−3·6% (−5·3 to −1·6)
	Swaziland	12 945 (10 863 to 15 167)	12 348 (10 629 to 14 289)	797 (223 to 1609)	3·5% (2·6 to 4·3)	3·1% (2·4 to 3·8)	0·8% (−4·3 to 4·8)	−0·5% (−1·7 to 0·6)	0·1% (−0·8 to 1·0)	−3·6% (−11·3 to 2·0)
	Zimbabwe	61 005 (54 341 to 68 687)	62 175 (56 363 to 68 696)	6325 (3273 to 10 613)	2·2% (1·5 to 2·9)	3·0% (2·4 to 3·6)	2·3% (−1·7 to 5·6)	−1·1% (−2·2 to −0·2)	−1·6% (−2·5 to −0·7)	−3·6% (−9·8 to 2·6)
Western sub-Saharan Africa		433 268 (386 857 to 488 595)	411 199 (371 387 to 455 335)	68 861 (54 681 to 103 225)	−1·1% (−1·3 to −1·0)	−1·2% (−1·3 to −1·1)	−2·2% (−3·5 to −1·0)	−0·8% (−1·2 to −0·4)	−0·6% (−1·0 to −0·2)	−2·9% (−4·6 to −1·3)
	Benin	10 193 (9232 to 11 291)	10 081 (9266 to 10 955)	2676 (1316 to 4945)	−0·7% (−1·1 to −0·4)	−0·6% (−0·8 to −0·3)	−1·2% (−3·9 to 1·3)	−1·5% (−2·0 to −1·0)	−1·5% (−2·0 to −1·0)	−1·7% (−7·0 to 3·0)
	Burkina Faso	20 972 (19 126 to 23 464)	21 505 (19 941 to 23 232)	5727 (3311 to 9314)	−0·7% (−1·1 to −0·4)	−0·5% (−0·8 to −0·2)	−1·3% (−3·6 to 1·0)	−1·3% (−1·9 to −0·7)	−1·1% (−1·5 to −0·7)	−1·5% (−6·7 to 3·6)
	Cameroon	22 532 (19 863 to 25 670)	21 665 (19 382 to 24 146)	3294 (1561 to 6645)	−1·2% (−1·6 to −0·7)	−1·3% (−1·7 to −1·0)	−1·2% (−3·4 to 1·1)	−2·0% (−2·8 to −1·4)	−2·1% (−2·7 to −1·6)	−3·6% (−9·2 to 1·7)
	Cape Verde	260 (225 to 300)	238 (209 to 274)	24 (18 to 39)	−2·6% (−2·9 to −2·2)	−2·4% (−2·8 to −2·1)	−4·7% (−7·6 to −0·2)	−0·5% (−1·2 to 0·2)	−0·5% (−1·1 to 0·2)	−5·7% (−9·4 to −1·4)
	Chad	19 053 (17 139 to 21 300)	19 395 (17 659 to 21 360)	3903 (1819 to 7567)	−0·0% (−0·3 to 0·3)	0·3% (−0·0 to 0·6)	−0·1% (−3·0 to 2·7)	−1·3% (−1·9 to −0·7)	−1·0% (−1·5 to −0·5)	−2·6% (−9·1 to 2·5)
	Côte d'Ivoire	28 208 (25 291 to 31 648)	27 208 (24 833 to 29 747)	5161 (2599 to 9614)	−1·1% (−1·5 to −0·7)	−1·3% (−1·6 to −1·0)	−1·1% (−3·2 to 0·8)	−1·7% (−2·3 to −1·1)	−1·8% (−2·3 to −1·3)	−2·6% (−8·0 to 2·4)
	The Gambia	1567 (1392 to 1764)	1450 (1303 to 1610)	312 (197 to 534)	−1·2% (−1·5 to −0·9)	−1·3% (−1·6 to −1·1)	−1·4% (−4·9 to 2·2)	−1·2% (−1·7 to −0·6)	−1·2% (−1·8 to −0·7)	−2·7% (−6·8 to 1·6)
	Ghana	26 769 (24 279 to 29 930)	26 016 (24 037 to 28 113)	4106 (2192 to 7034)	−2·1% (−2·5 to −1·7)	−1·9% (−2·2 to −1·6)	−4·0% (−7·1 to −0·5)	−1·9% (−2·6 to −1·3)	−1·7% (−2·2 to −1·2)	−4·2% (−9·5 to 0·6)
	Guinea	14 101 (12 765 to 15 823)	13 494 (12 308 to 14 785)	3657 (2089 to 6162)	−0·2% (−0·6 to 0·2)	−0·3% (−0·6 to 0·0)	−0·4% (−2·3 to 1·7)	−1·7% (−2·3 to −1·1)	−1·8% (−2·3 to −1·2)	−1·3% (−6·1 to 3·1)
	Guinea-Bissau	2666 (2385 to 2989)	2597 (2375 to 2848)	679 (235 to 1985)	−0·2% (−0·6 to 0·1)	−0·2% (−0·5 to 0·1)	−0·9% (−7·9 to 6·3)	−1·0% (−1·6 to −0·4)	−0·9% (−1·4 to −0·4)	−1·8% (−11·2 to 6·1)
	Liberia	7857 (6879 to 8974)	7047 (6084 to 8156)	2100 (1185 to 4029)	−1·3% (−1·7 to −0·9)	−1·9% (−2·3 to −1·6)	−1·0% (−3·6 to 2·0)	−0·5% (−1·0 to 0·0)	−0·4% (−1·0 to 0·2)	−2·4% (−6·6 to 1·9)
	Mali	14 014 (12 630 to 15 700)	13 645 (12 426 to 14 977)	3629 (1733 to 5977)	−1·7% (−2·0 to −1·4)	−1·3% (−1·6 to −1·1)	−3·1% (−4·8 to −1·4)	−1·6% (−2·1 to −1·1)	−1·5% (−2·0 to −1·0)	−1·6% (−5·9 to 2·5)
	Mauritania	3393 (3016 to 3807)	3255 (2944 to 3599)	566 (304 to 1018)	−3·1% (−3·4 to −2·7)	−3·3% (−3·6 to −3·1)	−4·2% (−7·1 to −1·2)	−1·2% (−1·8 to −0·8)	−0·9% (−1·4 to −0·5)	−3·2% (−8·3 to 1·4)
	Niger	23 295 (21 091 to 25 887)	21 743 (19 668 to 24 062)	7067 (4005 to 13 390)	−0·8% (−1·1 to −0·6)	−1·0% (−1·3 to −0·8)	−1·1% (−3·0 to 0·9)	−1·1% (−1·6 to −0·6)	−1·0% (−1·5 to −0·5)	−0·9% (−5·3 to 3·3)
	Nigeria	200 044 (173 240 to 228 875)	184 815 (162 132 to 209 748)	16 425 (9013 to 37 039)	−1·2% (−1·5 to −0·9)	−1·3% (−1·6 to −1·1)	−3·5% (−6·5 to −0·6)	0·1% (−0·6 to 0·7)	0·3% (−0·4 to 0·9)	−5·3% (−9·5 to −0·9)
	São Tomé and Principe	110 (97 to 126)	101 (88 to 116)	21 (11 to 40)	−1·5% (−1·9 to −1·1)	−1·7% (−2·1 to −1·4)	−0·9% (−3·0 to 1·3)	0·0% (−0·6 to 0·6)	0·3% (−0·3 to 0·9)	−2·8% (−8·0 to 1·2)
	Senegal	24 556 (21 977 to 27 776)	24 032 (21 868 to 26 251)	6330 (3321 to 11 182)	−1·6% (−2·0 to −1·3)	−1·7% (−1·9 to −1·4)	−1·7% (−4·3 to 1·0)	−0·8% (−1·4 to −0·2)	−0·9% (−1·3 to −0·4)	−0·9% (−5·8 to 3·8)
	Sierra Leone	7989 (7194 to 9072)	7320 (6671 to 8083)	1662 (958 to 2812)	0·4% (0·1 to 0·8)	0·3% (0·0 to 0·6)	0·3% (−2·3 to 3·4)	−1·7% (−2·3 to −1·1)	−1·4% (−1·9 to −0·9)	−4·1% (−8·9 to 0·1)
	Togo	5682 (5124 to 6351)	5587 (5094 to 6148)	1518 (851 to 2533)	−0·8% (−1·1 to −0·5)	−0·8% (−1·1 to −0·6)	−0·7% (−3·2 to 1·7)	−1·9% (−2·4 to −1·3)	−2·0% (−2·5 to −1·5)	−3·0% (−8·1 to 1·9)

Data in parentheses are 95% uncertainty intervals.

**Figure 2:**
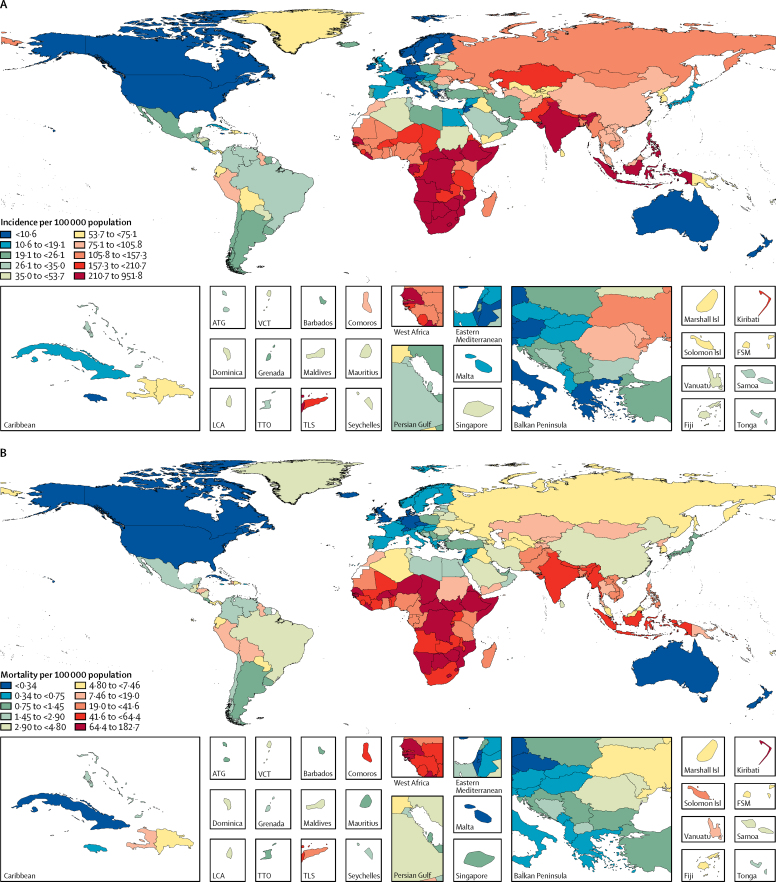
Age-standardised rates (per 100 000 population) of tuberculosis incidence (A) and mortality (B) in HIV-negative individuals in 2015 for both sexes ATG=Antigua and Barbuda. FSM=Federated States of Micronesia. LCA=Saint Lucia. Marshall Isl=Marshall Islands. Solomon Isl=Solomon Islands. TLS=Timor-Leste. TTO=Trinidad and Tobago. VCT=Saint Vincent and the Grenadines.

Figure 2 shows maps of age-standardised incidence and death rates for tuberculosis in HIV-negative individuals in 2015. The age-standardised incidence rate of tuberculosis in HIV-negative people was more than 210 per 100 000 population in 17 countries in sub-Saharan Africa as well as India, Indonesia, and the Philippines. Death rates in HIV-negative individuals were more than 50 per 100 000 population in 25 countries in sub-Saharan Africa as well as Indonesia, Kiribati, Myanmar, and Nepal. Death rates varied greatly in north Africa and the Middle East, ranging from 0·1 (95% UI 0·1–0·2) per 100 000 in Palestine in 2015 to 30·1 (18·2–44·5) per 100 000 in Afghanistan. Detailed results, broken down by age and sex, are available online.

### Observed versus expected tuberculosis incidence, prevalence, and mortality

**Figure 3:**
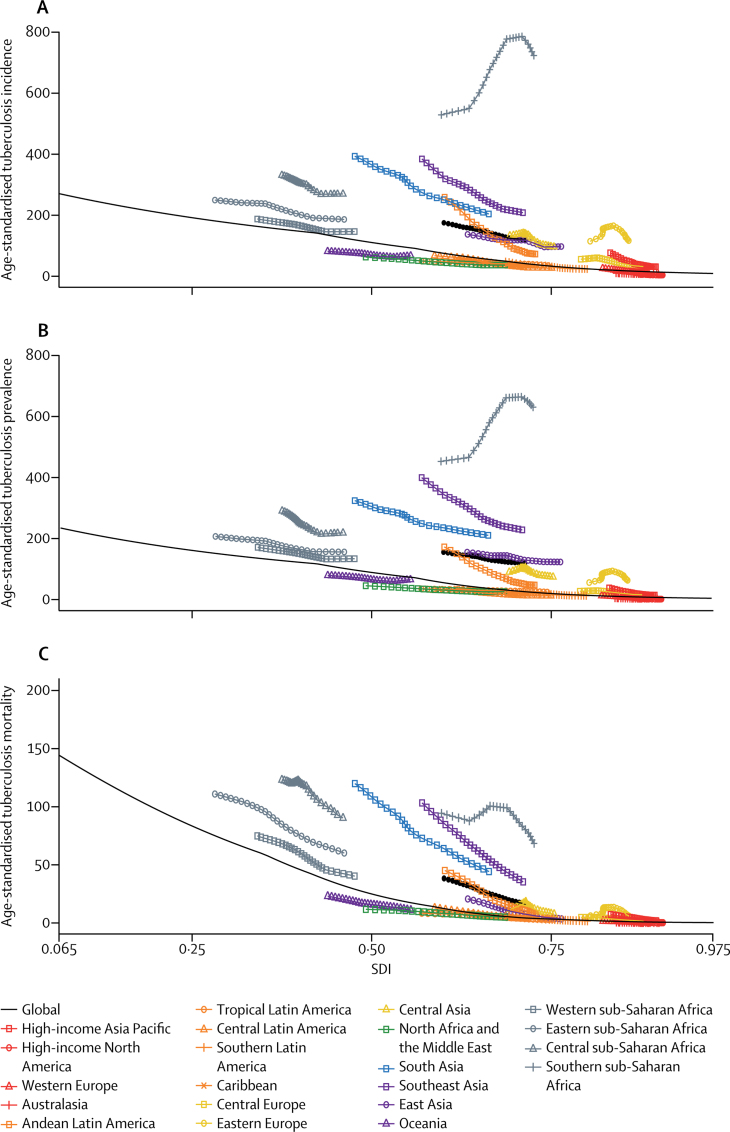
Estimated observed and expected age-standardised rates of tuberculosis incidence (A), prevalence (B), and mortality (C) per 100 000 population among HIV-negative individuals based on SDI, 1990–2015 Each point on a line represents 1 year, starting at 1990 and ending at 2015. In all regions, SDI has increased year on year, so progress in SDI is associated with later years for a given region. The black lines indicate trajectories for each geography expected based on SDI alone. SDI=Socio-demographic Index.

Globally and in most regions, age-standardised tuberculosis incidence, prevalence, and mortality rates showed a steady decline with rising SDI (figure 3). Many regions (eg, southeast Asia, south Asia, central Asia, eastern Europe, Andean Latin America, and sub-Saharan Africa) had higher than expected incidence, prevalence, and mortality rates, whereas a few others (eg, Oceania and north Africa and the Middle East) showed lower than expected levels over time (appendix). Of all regions in 2015, southern sub-Saharan Africa had the largest difference between observed and expected levels, although the observed mortality has begun to fall closer to expected levels since around 2007. The gaps between observed and expected incidence and mortality also gradually decreased over time in several other regions (eg, southeast Asia, south Asia, and Andean Latin America), but we observed little change in the gaps for central, eastern, and western sub-Saharan Africa. In east Asia, we observed little change in the gap between observed and expected levels of incidence and prevalence over time, although the observed mortality converged with expected levels during 2015. In eastern Europe, the observed incidence, prevalence, and mortality increased between 1990 and 2005 but has begun to fall closer to expected levels in the last decade.

### Tuberculosis mortality and DALYs attributable to individual risk factors

Table 3 shows the global and regional tuberculosis deaths attributable to smoking, alcohol use, and diabetes in 2015 and the corresponding ARCs for age-standardised rates of death in individuals who are HIV negative (the appendix contains DALYs attributable to the three risk factors and ARCs). Globally, in 2015, among HIV-negative individuals, alcohol use accounted for 126 459 (95% UI 94 124–168 699) tuberculosis deaths, followed by diabetes (118 298 [73 111–169 308] deaths) and smoking (86 849 [41 265–140 152] deaths). The corresponding PAF due to alcohol was 11·4% (9·3–13·0), due to diabetes was 10·6% (6·8–14·8), and due to smoking was 7·8% (3·8–12·0), and we observed no significant difference between the PAFs due to these three risk factors (appendix). Age-standardised tuberculosis deaths attributable to smoking changed at a faster rate per year than did those attributable to alcohol use and diabetes from 2005 to 2015 (table 3). Across regions, ARCs for age-standardised tuberculosis deaths attributable to smoking varied from −2·4% (−7·3 to 2·3) in central sub-Saharan Africa to −8·7% (−9·7 to −7·6) in eastern Europe and to alcohol use from −1·9% (−6·3 to 2·4) to −8·3% (−9·3 to −7·1). ARCs for age-standardised tuberculosis deaths attributable to diabetes varied from −1·3% (−5·3 to 2·3) in central sub-Saharan Africa to −8·6% (−10·0 to −6·8) in east Asia.

**Table 3:** Tuberculosis deaths attributable to smoking, alcohol use, and diabetes and annualised rates of change of age-standardised rates in HIV-negative individuals (2005–15)

	**Tuberculosis deaths**						**Annualised rate of change from 2005 to 2015 (%)**		
	Smoking		Alcohol		Diabetes		Smoking	Alcohol use	Diabetes
	2005	2015	2005	2015	2005	2015			
Global	114 069 (56 813 to 179 882)	86 849 (41 265 to 140 152)	146 817 (117 226 to 187 496)	126 459 (94 124 to 168 699)	133 100 (82 683 to 188 878)	118 298 (73 111 to 169 308)	−5·1% (−6·3 to −4·2)	−3·7% (−4·8 to −2·7)	−3·6% (−4·6 to −2·8)
High SDI	6886 (3520 to 10 155)	3346 (1707 to 5022)	12 534 (10 743 to 14 078)	6331 (5458 to 7308)	3137 (2019 to 4452)	2031 (1277 to 2937)	−8·8% (−9·8 to −7·8)	−8·3% (−9·4 to −7·3)	−6·5% (−7·6 to −5·4)
High-middle SDI	12 933 (6298 to 20 234)	8584 (4138 to 13 972)	21 941 (17 134 to 26 222)	17 222 (12 989 to 21 440)	15 356 (9485 to 21 754)	12 134 (7566 to 17 271)	−6·6% (−7·5 to −5·9)	−4·7% (−5·7 to −3·9)	−5·0% (−5·8 to −4·2)
Middle SDI	42 607 (20 870 to 70 447)	32 491 (15 648 to 55 436)	41 712 (31 506 to 54 309)	33 536 (25 163 to 45 974)	47 369 (29 389 to 68 228)	39 300 (23 747 to 57 970)	−5·5% (−7·2 to −4·0)	−4·6% (−5·9 to −3·3)	−4·6% (−5·9 to −3·4)
Low-middle SDI	44 057 (22 359 to 70 192)	34 828 (15 945 to 56 609)	54 079 (42 417 to 73 518)	51 738 (36 961 to 70 298)	55 409 (34 245 to 79 602)	51 128 (31 647 to 74 115)	−5·0% (−7·2 to −3·5)	−3·0% (−4·9 to −1·4)	−3·4% (−5·0 to −2·1)
Low SDI	7516 (3243 to 12 920)	7538 (3230 to 13322)	16 505 (11 834 to 23 052)	17586 (10776 to 25822)	11 756 (6530 to 18 829)	13 628 (7610 to 22 723)	−3·2% (−5·8 to −0·7)	−2·6% (−5·4 to 0·1)	−1·6% (−3·7 to 0·5)
High-income Asia Pacific	1042 (538 to 1577)	631 (316 to 965)	809 (578 to 1082)	605 (409 to 864)	647 (343 to 1016)	543 (274 to 879)	−7·9% (−8·9 to −7·1)	−5·6% (−7·1 to −4·2)	−4·8% (−5·9 to −3·8)
Central Asia	1125 (543 to 1734)	715 (336 to 1116)	1967 (1534 to 2283)	1245 (855 to 1533)	679 (408 to 970)	514 (309 to 732)	−6·7% (−7·8 to −5·3)	−6·7% (−8·4 to −5·3)	−5·3% (−6·2 to −4·3)
East Asia	12 793 (6135 to 20 759)	7625 (3464 to 14 249)	13 519 (10 206 to 19 589)	9476 (7278 to 15 322)	8491 (4978 to 12 832)	4739 (2657 to 8097)	−7·9% (−9·4 to −6·1)	−6·1% (−7·7 to −4·2)	−8·6% (−10·0 to −6·8)
South Asia	45 561 (22 154 to 72 939)	35 394 (15 912 to 57 471)	64 036 (47 953 to 84 547)	58 989 (40 831 to 80 302)	69 867 (43 938 to 99 563)	60 034 (37 096 to 87 139)	−5·3% (−7·3 to −3·9)	−3·4% (−5·2 to −2·0)	−4·2% (−5·7 to −3·1)
Southeast Asia	29 048 (14 917 to 47 933)	23 720 (11 854 to 41 019)	12 625 (10 301 to 15 958)	11 223 (8164 to 15 007)	25 226 (15 409 to 37 224)	24 188 (14 373 to 36 939)	−5·1% (−7·4 to −3·1)	−3·8% (−6·0 to −2·1)	−3·4% (−5·3 to −1·7)
Australasia	6 (4 to 9)	4 (3 to 7)	15 (13 to 17)	13 (11 to 16)	8 (4 to 12)	7 (4 to 12)	−6·0% (−7·0 to −5·0)	−3·8% (−5·4 to −2·2)	−3·1% (−4·3 to −1·9)
The Caribbean	118 (57 to 201)	81 (39 to 149)	263 (191 to 435)	261 (177 to 456)	168 (95 to 264)	173 (94 to 277)	−5·9% (−8·0 to −4·0)	−1·9% (−3·8 to 0·0)	−2·0% (−3·6 to −0·5)
Central Europe	595 (304 to 882)	327 (169 to 493)	1156 (1055 to 1264)	664 (588 to 753)	326 (200 to 483)	210 (130 to 305)	−6·8% (−7·8 to −5·7)	−6·3% (−7·5 to −5·0)	−5·4% (−6·4 to −4·4)
Eastern Europe	6755 (3379 to 9970)	2979 (1511 to 4399)	13 726 (12 005 to 15 247)	6296 (5532 to 7152)	2331 (1420 to 3277)	1153 (709 to 1632)	−8·7% (−9·7 to −7·6)	−8·3% (−9·3 to −7·1)	−7·9% (−9·2 to −6·6)
Western Europe	383 (215 to 568)	244 (139 to 362)	806 (685 to 935)	556 (425 to 685)	327 (165 to 518)	266 (130 to 434)	−6·2% (−6·9 to −5·5)	−5·5% (−6·9 to −4·6)	−4·3% (−5·1 to −3·4)
Andean Latin America	299 (148 to 573)	216 (102 to 420)	693 (529 to 1324)	573 (407 to 1119)	294 (166 to 497)	282 (156 to 480)	−6·2% (−7·9 to −4·6)	−4·5% (−6·3 to −2·9)	−3·4% (−4·7 to −2·3)
Central Latin America	422 (219 to 649)	302 (148 to 480)	1024 (915 to 1151)	869 (728 to 1012)	822 (531 to 1142)	771 (505 to 1077)	−6·7% (−7·6 to −5·8)	−4·9% (−5·8 to −4·0)	−4·0% (−4·7 to −3·4)
Southern Latin America	142 (79 to 212)	100 (55 to 152)	240 (185 to 292)	204 (147 to 256)	128 (73 to 194)	101 (57 to 153)	−5·4% (−6·6 to −4·2)	−3·5% (−5·3 to −1·8)	−4·6% (−5·6 to −3·4)
Tropical Latin America	614 (316 to 949)	409 (196 to 643)	1333 (831 to 1699)	1153 (699 to 1544)	724 (432 to 1029)	700 (401 to 1035)	−7·0% (−8·3 to −5·5)	−4·2% (−5·4 to −2·7)	−3·5% (−4·7 to −1·9)
North Africa and the Middle East	1324 (658 to 2142)	1197 (581 to 2049)	638 (483 to 853)	583 (415 to 797)	2647 (1661 to 3789)	2800 (1784 to 4050)	−4·3% (−5·8 to −3·0)	−4·0% (−5·5 to −2·7)	−2·6% (−3·8 to −1·4)
High-income North America	89 (51 to 130)	66 (38 to 97)	164 (144 to 184)	138 (112 to 166)	145 (85 to 212)	140 (82 to 207)	−5·1% (−5·6 to −4·6)	−3·6% (−4·7 to −2·7)	−2·5% (−3·1 to −2·0)
Oceania	92 (45 to 156)	88 (43 to 158)	49 (27 to 81)	47 (24 to 82)	137 (78 to 213)	142 (79 to 230)	−3·6% (−6·2 to −1·0)	−3·1% (−6·3 to 0·2)	−2·7% (−4·9 to −0·5)
Central sub-Saharan Africa	2021 (649 to 5237)	2222 (660 to 5970)	3173 (1151 to 7480)	3595 (1232 to 8767)	3941 (1581 to 8704)	4694 (1848 to 10 753)	−2·4% (−7·3 to 2·3)	−1·9% (−6·3 to 2·4)	−1·3% (−5·3 to 2·3)
Eastern sub-Saharan Africa	5006 (2290 to 8398)	4865 (2040 to 8756)	13 144 (9631 to 16 969)	14 099 (8548 to 20 130)	6351 (3710 to 9459)	7119 (3809 to 11 616)	−3·6% (−6·8 to −0·8)	−2·6% (−6·1 to 0·6)	−2·1% (−5·1 to 0·8)
Southern sub-Saharan Africa	4011 (1909 to 6310)	3287 (1552 to 5427)	8146 (5653 to 10 068)	7342 (4857 to 9429)	5494 (3309 to 7929)	5013 (2984 to 7222)	−4·2% (−6·3 to −1·8)	−3·1% (−5·0 to −0·9)	−3·0% (−4·7 to −1·0)
Western sub-Saharan Africa	2624 (1277 to 4670)	2376 (1121 to 4350)	9292 (6897 to 14 627)	8527 (5750 to 14 979)	4348 (2540 to 6778)	4711 (2666 to 7536)	−4·0% (−6·1 to −2·0)	−3·6% (−5·9 to −0·9)	−1·8% (−3·5 to −0·1)

Data in parentheses are 95% uncertainty intervals. SDI=Socio-demographic Index.

**Figure 4:**
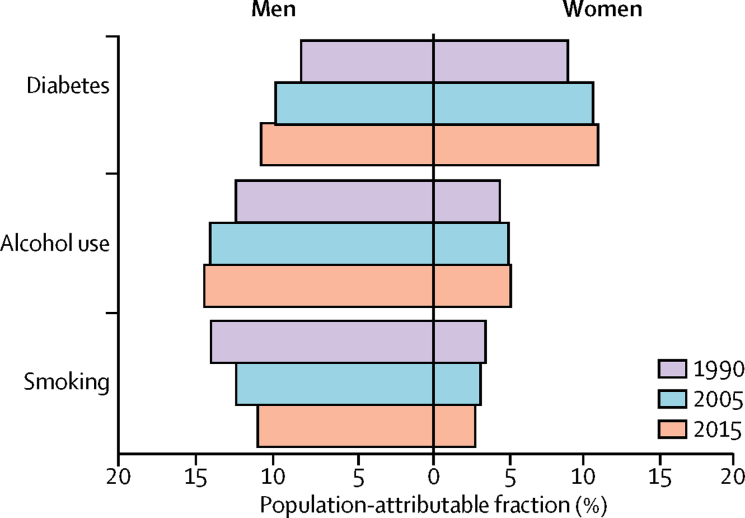
Age-standardised population-attributable fractions of tuberculosis deaths due to diabetes, alcohol use, and smoking among HIV-negative men and women in 1990, 2005, and 2015

Figure 4 shows the age-standardised PAFs for global tuberculosis deaths due to the three risk factors among HIV-negative male and female individuals in 1990, 2005, and 2015 (the appendix contains PAFs for DALYs). The age-standardised PAFs for tuberculosis deaths due to smoking and alcohol use were between four times and six times higher among men than among women across all three timepoints, whereas they were similar between sexes for diabetes. In both men and women, PAFs for smoking, alcohol use, and diabetes did not change substantially from 1990 to 2005 and 2005 to 2015.

## Discussion

Globally, substantial progress has been made in reducing mortality from tuberculosis. However, age-standardised tuberculosis incidence and prevalence are declining much more slowly than mortality in many countries. Despite a powerful interaction between tuberculosis and HIV, most tuberculosis cases and deaths occur among HIV-negative people in south and southeast Asia, where HIV prevalence is relatively low. Most of Asia, eastern Europe, and all of sub-Saharan Africa had higher tuberculosis burden than expected given their level of sociodemographic development.

Despite a decline in mortality from tuberculosis, an estimated 1·1 million deaths still occurred among HIV-negative individuals worldwide in 2015, along with 0·2 million deaths among HIV-positive individuals. Age-standardised mortality rates due to tuberculosis are declining at a slower pace than are those due to HIV and malaria.^3^ Whereas improved access to treatment probably reduced tuberculosis deaths, large funding gaps remain, with the largest gap being for multidrug-resistant (MDR) tuberculosis.^27^ WHO and the Global Fund to Fight AIDS, Tuberculosis and Malaria estimated that at least US$1·6 billion of international support was required annually to fill the funding gap for tuberculosis control between 2014 and 2016 in 118 low-income and middle-income countries.^27^ However, the growth rate of development assistance for tuberculosis has decelerated substantially since 2010,^28^ making it more challenging for health systems to reduce the burden of tuberculosis in low-income countries than in middle-income and high-income countries.

Tuberculosis incidence is either stagnant or declining more slowly than mortality in many tuberculosis-endemic countries, suggesting delays in diagnosis and treatment.^29^, ^30^, ^31^, ^32^, ^33^ One untreated patient with tuberculosis can infect many healthy contacts.^34^, ^35^ Although only a small proportion of infected people progress to active tuberculosis, it is difficult to predict who will progress from latent infection to active disease.^36^ Early diagnosis of active tuberculosis is challenging; substantial delays in diagnosis and treatment have been linked to multiple factors, including absence of awareness of symptoms, absence of access to health services, shortages of trained clinicians and laboratory personnel to make the diagnosis, and poor diagnostic tools.^30^, ^31^, ^37^, ^38^ High proportions of initial default (ie, never starting tuberculosis treatment) have been reported in settings relying on passive case finding.^39^, ^40^, ^41^, ^42^ Community-wide active case finding aims to reduce barriers to early detection, but few studies have evaluated the cost-effectiveness of screening for active tuberculosis.^43^ Evidence suggests that compared with conventional smear microscopy, use of sputum Xpert-MTB/RIF (Cepheid, USA) substantially increases case detection (by almost 50%) during intensified case finding in high-burden community settings.^44^ Studies evaluating the cost-effectiveness of screening for active tuberculosis using new diagnostic tools, such as Xpert-MTB/RIF, would therefore be very useful. Tuberculosis incidence is also declining more slowly than mortality in various low-tuberculosis-burden countries, with some showing either stagnant or increasing trends in incidence. Several low-tuberculosis-burden countries do not have a national tuberculosis programme or elimination plan to guide control efforts.^45^

Our results showed a notable difference in the global age distribution of tuberculosis cases and deaths: cases were highest among young adults, but deaths were highest among old adults. This finding might be explained by a greater risk of reactivation of latent tuberculosis in younger adults as reported by longitudinal birth cohort studies^46^, ^47^ and a higher risk of adverse reactions from anti-tuberculosis drugs^48^ and mortality in older people.^49^, ^50^ Our results also showed that age-standardised incidence and mortality from tuberculosis were about twice as high in men than in women. Various explanations have been suggested for the sex difference in tuberculosis risk, including differential access to health care, differential exposure to risk factors (eg, smoking), and genetic variation.^51^, ^52^, ^53^ An understanding of the age–sex distribution of tuberculosis cases and deaths has implications for tuberculosis control programmes in terms of targeting of interventions to high-risk groups.

Risk factors also play an important part in the control of tuberculosis. For example, alcohol abuse has been linked to poor tuberculosis treatment compliance and outcomes.^54^, ^55^, ^56^ Moreover, tuberculosis risk factors, including diabetes, alcohol use, and smoking, could increase the risk of tuberculosis through suppression of the immune system, especially cell-mediated immunity.^57^, ^58^, ^59^, ^60^ With an increase in diabetes prevalence as countries go through demographic and epidemiological transition,^8^ many low-income and middle-income countries will increasingly bear the double burden of tuberculosis and diabetes. Globally, in 2015, diabetes, alcohol use, and smoking together accounted for about a quarter of tuberculosis deaths and DALYs. Efforts to prevent these risk factors can therefore have a substantial collateral impact on the burden of tuberculosis.

Our method for computation of tuberculosis burden differs from that used by WHO and results in different estimates in some locations. At the global level, our tuberculosis (all forms) incidence estimate (10·2 million cases) is slightly lower than that of WHO (10·4 million cases) in 2015, but we estimate a higher proportion of HIV–tuberculosis (13%) than does WHO (11%).^5^ Our estimated number of all tuberculosis deaths (1·3 million) is lower than WHO's estimate (1·8 million) for 2015. The WHO global prevalence estimates for 2015 were unavailable for comparison. At the country level, our list of countries with a high burden of tuberculosis is consistent with that of WHO, with a few exceptions. The WHO top 20 high-burden countries as assessed by incident case numbers include Angola, Kenya, and North Korea, which in our list are replaced by Uganda, Ukraine, and Zimbabwe.

These discrepancies stem from differences in the methods used. WHO generated incidence estimates for 74 countries by adjusting notification data on the basis of expert opinion of the case detection rate. By contrast, our estimates of prevalence and incidence are driven by the statistical triangulation that enforces consistency between the data overall for different parameters and are ultimately based on the logical relationships between age-specific and sex-specific incidence, prevalence, remission, excess mortality, and cause-specific mortality with use of a Bayesian meta-regression method. Our statistical triangulation of all sources of data for a country revealed discrepancies between notifications, prevalence, and cause of death data in some countries (eg, the incidence model showed a pattern in under-reporting of notification data, which increased with age). In many high-tuberculosis-burden settings, tuberculosis cases treated in the private sector are not notified; barriers to notification include, but are not limited to, confidentiality concerns, ignorance of reporting procedure, and scarcity of time.^61^ Strengthening tuberculosis notification and vital registration systems is needed to improve the quality of data.^62^, ^63^ Until such systems are fully developed, variation in estimates is unavoidable and should be appreciated by users of these estimates. Various interim improvement options have been suggested, including use of inventory studies to assess under-reporting of notification data^62^, ^64^ and sample-based mortality surveillance to generate more robust cause-of-death data than so far possible.^65^ The availability of widely shared, high-quality data for low-income and middle-income countries and efforts to use a common set of data for estimation (which is being increasingly facilitated by WHO) would help reduce the discrepancy between GBD and WHO estimates.

Paediatric tuberculosis incidence has been estimated by different groups. We estimated that 690 262 (95% UI 551 275–859 100) incident cases of tuberculosis occurred among children aged younger than 15 years in 2015. Our estimate is lower than that from WHO (1 000 000 [900 000–1 100 000]) for both 2014^66^ and 2015^5^ and from Dodd and colleagues^67^ (847 000 [558 000–1 280 000]) for 2014. These differences are due to differences in the methods used. Dodd and colleagues^67^ used WHO tuberculosis prevalence data and demographic information to estimate childhood tuberculosis using a mathematical model. WHO combines the CDR adjustment approach (ie, incidence=notifications/estimated CDR^68^) and the method of Dodd and colleagues^67^ to produce their childhood tuberculosis incidence estimates.

This study has several limitations. First, our assessment of tuberculosis mortality in countries without vital registration data is driven by verbal autopsy studies, which have modest sensitivity in identifying tuberculosis deaths.^69^, ^70^, ^71^ Verbal autopsy studies have poor ability to distinguish HIV deaths from HIV–tuberculosis deaths; for this reason, we excluded verbal autopsy data in countries with high HIV prevalence. We applied various modelling methods by assuming that countries in the same region have a similar age–sex distribution of the tuberculosis burden as do other countries in that region and using many different combinations of covariates to help predict for locations and years with sparse or no data. Estimates for a location with sparse data are reflected by wide uncertainty intervals. Tuberculosis mortality estimates could be improved in the future by inclusion of additional covariates that have proximal relationships with tuberculosis mortality (eg, prevalence of latent tuberculosis infection).

Second, a major challenge in our statistical triangulation exercise has been the difficulty of finding consistent estimates between tuberculosis death rates and prevalence data from surveys, particularly in sub-Saharan Africa, where we have few prevalence surveys and often no usable cause of death data because of high HIV prevalence.

Third, although we used Bayesian meta-regression to generate a final incidence estimate that is consistent with prevalence data and cause-specific mortality estimates, use of CDRs as covariates is controversial since they are based on expert opinion. We plan to avoid using CDRs in the next iteration of GBD.

Fourth, our analysis of the relationship between SDI and tuberculosis incidence, prevalence, and mortality cannot be interpreted as being causal as it only reflects the average historical correlation between SDI and each of the measures. SDI use might also be low in countries with high income inequality. The applicability of SDI could be enhanced in the future by taking into account social heterogeneity within countries.

Fifth, despite the biological plausibility of a strong link between malnutrition and tuberculosis, we have not quantified the burden of tuberculosis attributable to malnutrition because of insufficient evidence of a causal relationship and a scarcity of information about the relative risk of tuberculosis associated with different levels of malnutrition.^21^, ^22^ We plan to assess the evidence for a causal relationship between low body-mass index in adults and risk of tuberculosis in a future iteration of GBD. We also have not quantified the burden of tuberculosis attributable to indoor air pollution since the evidence is based on cross-sectional (from which a causal relationship cannot be established) and case-control (none of which measured biofuel exposure objectively and were thus prone to recall bias) studies.^23^

Finally, in our modelling of tuberculosis, we did not separately examine the burden of MDR tuberculosis. Given the epidemiological and clinical importance of MDR tuberculosis, we plan to include MDR and extensively drug-resistant tuberculosis estimates in the next round of GBD estimation. Despite these limitations, we believe the methodological innovation with use of statistical triangulation of data sources has yielded more robust estimates than would be yielded from reliance on a single source of data. This approach could probably be further strengthened by incorporation of population-based surveys of latent tuberculosis infection and then modelling of the progression from latent tuberculosis infection to active tuberculosis disease. Estimation and mapping of tuberculosis incidence, prevalence, and deaths at a finer spatial resolution than current national and subnational estimates could also better inform surveillance and targeting of resources for interventions than at present.^72^

Strengthening of national surveillance systems to capture all tuberculosis cases is an important public health goal for all countries. Until this goal is achieved, statistical data triangulation methods will be needed to make use of the available data for tracking of the tuberculosis burden. Despite general progress in reduction of tuberculosis mortality, the disease is still an enormous burden globally. Strengthening of health systems for early case detection and improvement of the quality of tuberculosis care, including prompt and accurate diagnostics, early initiation of treatment, and routine follow-up, are priorities.^32^, ^73^ Countries where the tuberculosis burden is higher than expected based on sociodemographic development should investigate the reasons for lagging behind and address them as appropriate. Efforts to prevent smoking, alcohol use, diabetes, and HIV will also probably have a substantial collateral impact on reduction of the burden of tuberculosis.

Correspondence to: Prof Christopher J L Murray, Institute for Health Metrics and Evaluation, Seattle, WA 98121, USA **cjlm@uw.edu**
