# Supplementary material for: The global burden of tuberculosis: results from the Global Burden of Disease Study 2015
Source: Lancet Infect Dis. 2018 Mar;18(3):261–84. doi: 10.1016/S1473-3099(17)30703-X (PMC5831985; doi:10.1016/S1473-3099(17)30703-X)
Supplement: Supplementary appendix [file mmc1.pdf]

THE LANCET
Infectious Diseases

Supplementary appendix

This appendix formed part of the original submission and has been peer reviewed. We post it as supplied by the authors.

Supplement to: GBD Tuberculosis Collaborators. The global burden of tuberculosis: results from the Global Burden of Disease Study 2015. *Lancet Infect Dis* 2017; published online Dec 6. http://dx.doi.org/10.1016/S1473-3099(17)30703-X.

The global burden of tuberculosis: results from the Global Burden of Diseases, Injuries, and Risk Factors (GBD) 2015 Study

Supplementary Appendix

Contents

Appendix Figures 3

eFigure 1. Tuberculosis (TB) mortality: input data, analytical process, and output 3

eFigure 2. HIV-TB mortality: input data, analytical process, and output 4

eFigure 3. Non-fatal TB and HIV-TB: input data, analytical process, and output 5

eFigure 4. Bayesian meta-regression estimates for TB prevalence, incidence, remission, excess mortality, and cause-specific mortality for male individuals in Gujarat, rural, 2015 6

eFigure 5. Age-standardized population attributable fractions for TB DALYs due to diabetes, alcohol use, and smoking among HIV-negative male and female individuals in 1990, 2005, and 2015 7

Appendix Tables 8

eTable 1a. Beta coefficients and exponentiated values from the model using remission calculated based on incidence and prevalence data 8

eTable 1b. Beta coefficients and exponentiated values from the model applying the remission assumption for data-rich countries 9

eTable 2. Relative risks for the associations between smoking, diabetes, and alcohol use, and tuberculosis 10

eTable 3. Age-standardized tuberculosis (with and without HIV) incidence, prevalence, and mortality rates and annualized rates of change for both sexes for 21 GBD regions and five SDI categories 13

eTable 4. Tuberculosis DALYs (in HIV-negative individuals) attributable to smoking, alcohol use, and diabetes and annualized rate of change (2005–2015) for 21 Global Burden of Disease regions 15

eTable 5. Mean differences and 95% uncertainty intervals for population attributable fractions of risk factors for global tuberculosis deaths 18

eTable 6. List of data-rich locations 18

Comparative Risk Assessment 25

Alcohol 25
Smoking 33
High Fasting Plasma Glucose/Diabetes 37
Comparative risk assessment input data sources 42
Alcohol 42
Smoking 72
High Fasting Plasma Glucose/Diabetes 158
Supporting tables 213
Estimated observed age-standardized rates per 100,000 population of tuberculosis incidence, prevalence, and mortality by SDI values and GBD regions (1990-2015) 213
Expected age-standardized rates per 100,000 population of tuberculosis incidence, prevalence, and mortality among HIV-negative individuals based on SDI 228

Appendix Figures

eFigure 1. Tuberculosis (TB) mortality: input data, analytical process, and output

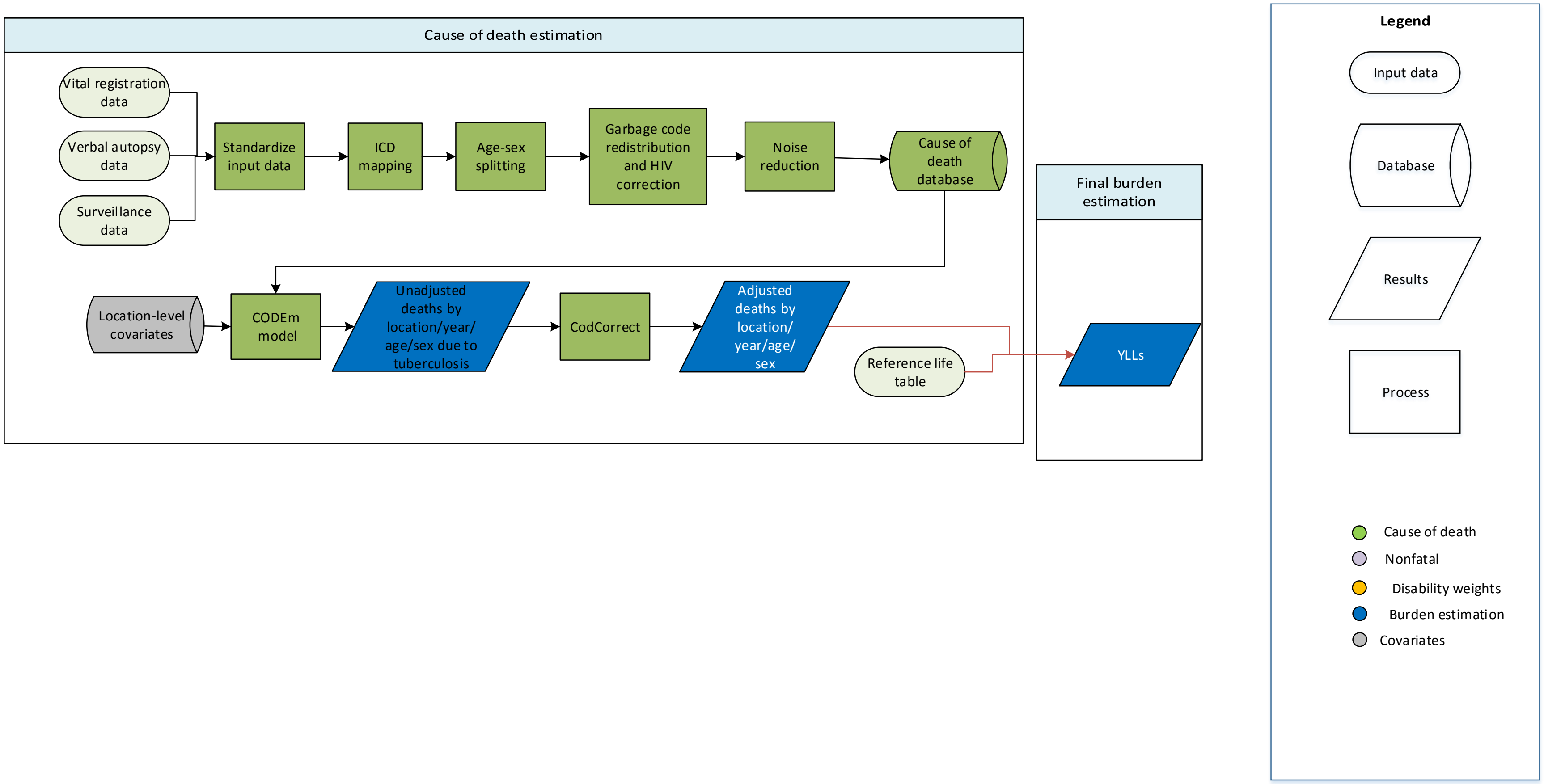

The GBD cause of death database is composed of vital registration data corrected for under-registration and garbage coding, national and subnational verbal autopsy studies corrected for garbage coding, and other sources including surveys and sureillance systems for specific causes such as maternal mortality.

eFigure 2. HIV-TB mortality: input data, analytical process, and output

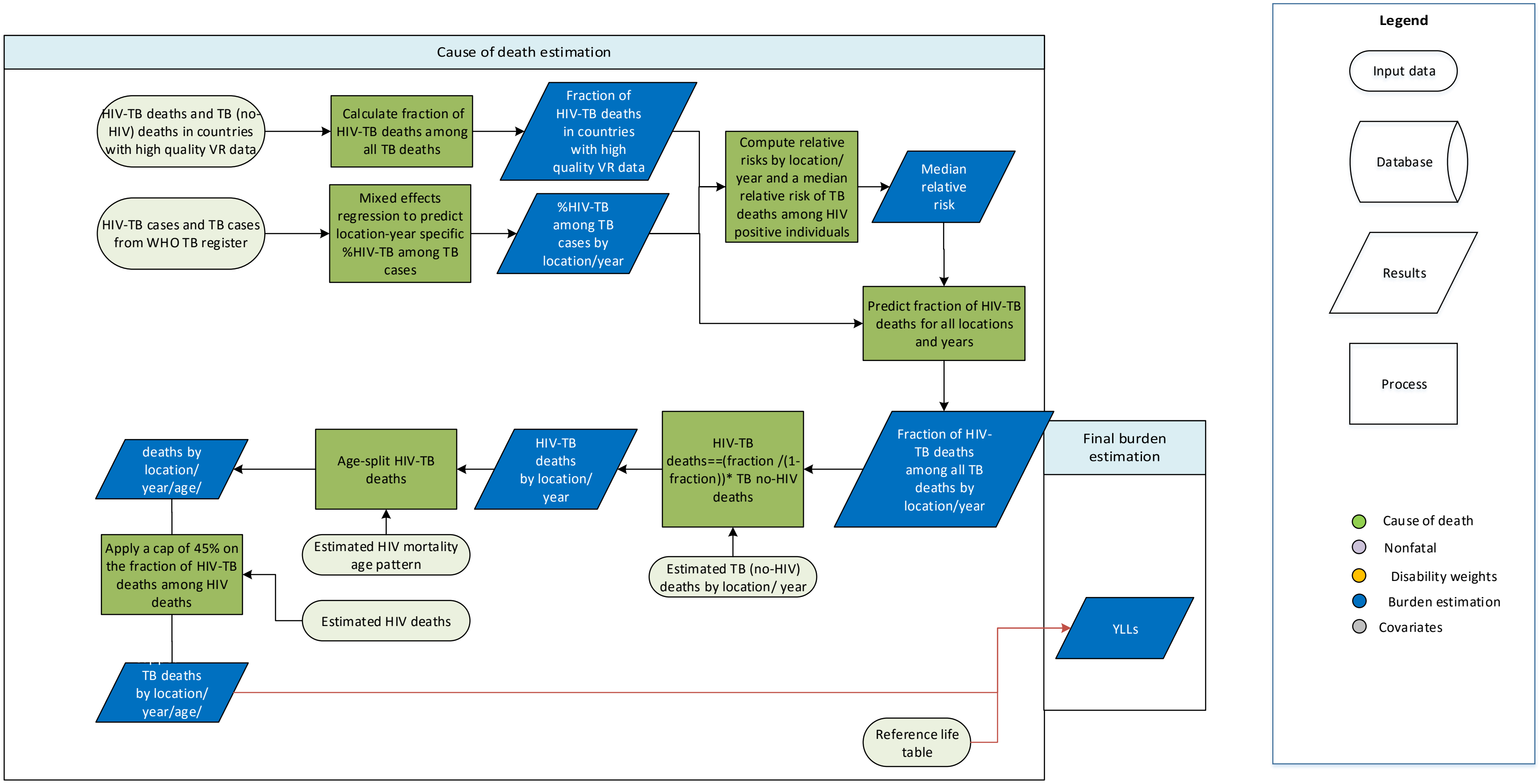

eFigure 3. Non-fatal TB and HIV-TB: input data, analytical process, and output

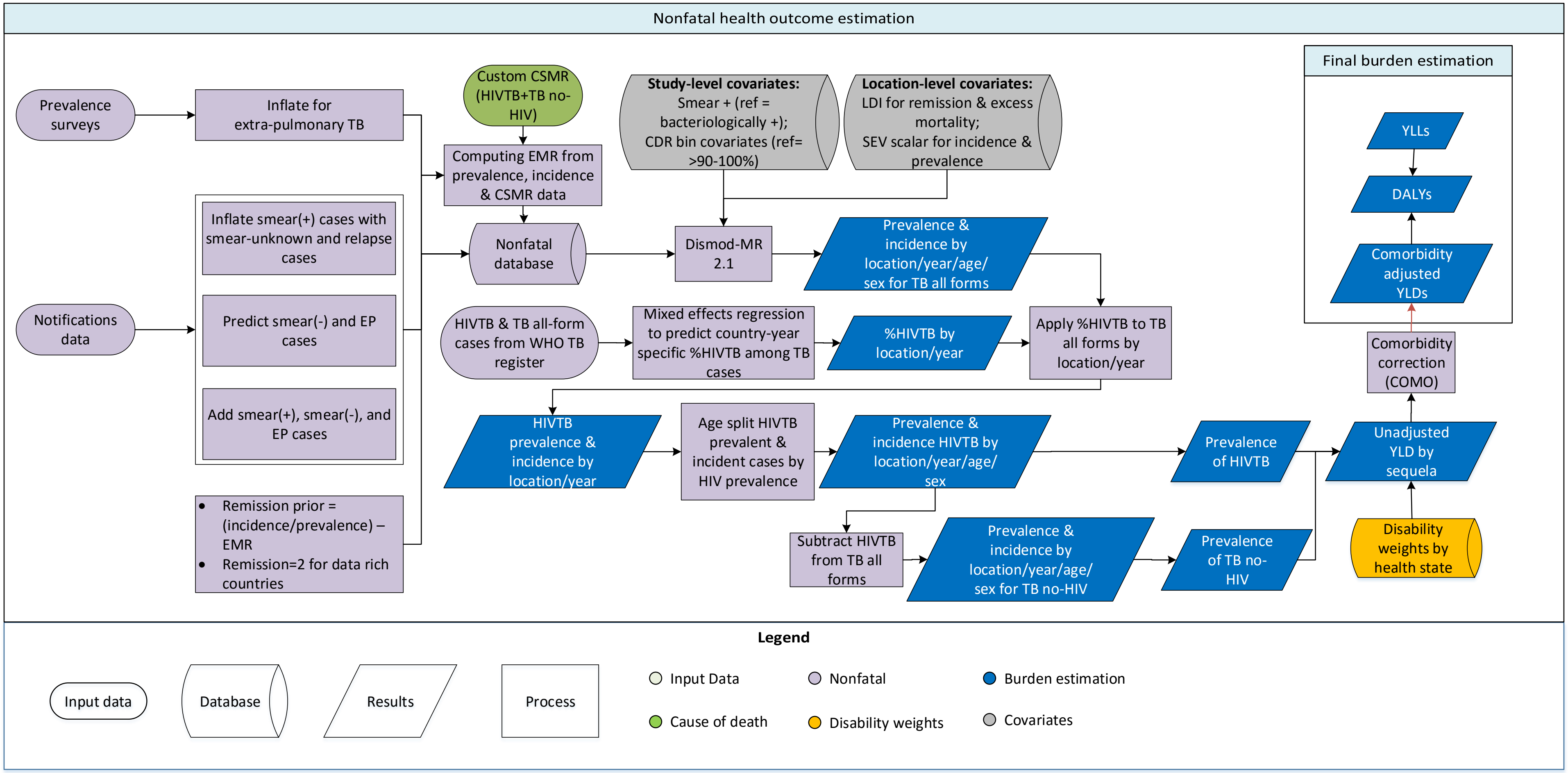

eFigure 4. Bayesian meta-regression estimates for TB prevalence, incidence, remission, excess mortality, and cause-specific mortality for male individuals in Gujarat, rural, 2015

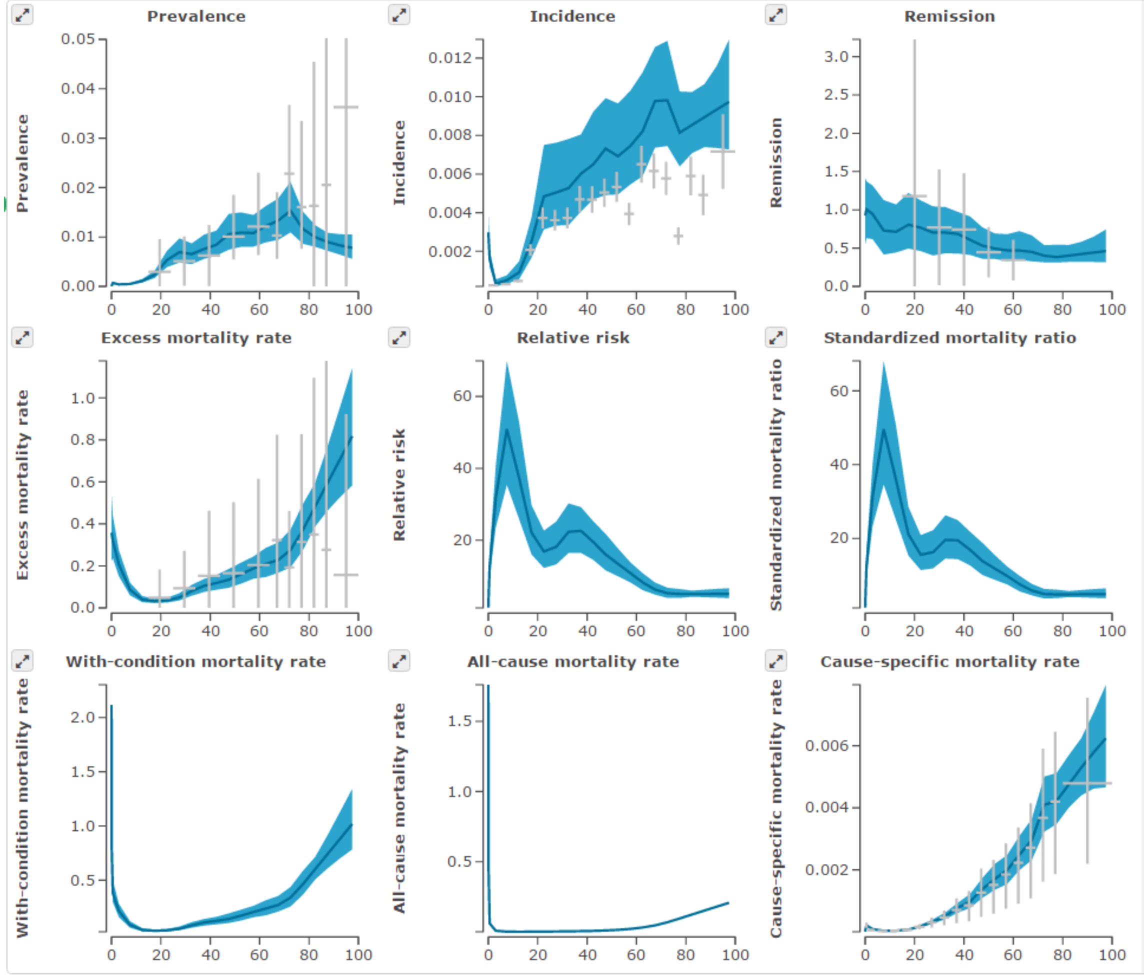

For each observation in grey, the length of the horizontal bar represents the age interval of the observation and the length of the vertical bar represents the uncertainty interval. The blue line represents the mean estimates and the shaded area represents uncertainty intervals.

eFigure 5. Age-standardized population attributable fractions for TB DALYs due to diabetes, alcohol use, and smoking among HIV-negative male and female individuals in 1990, 2005, and 2015

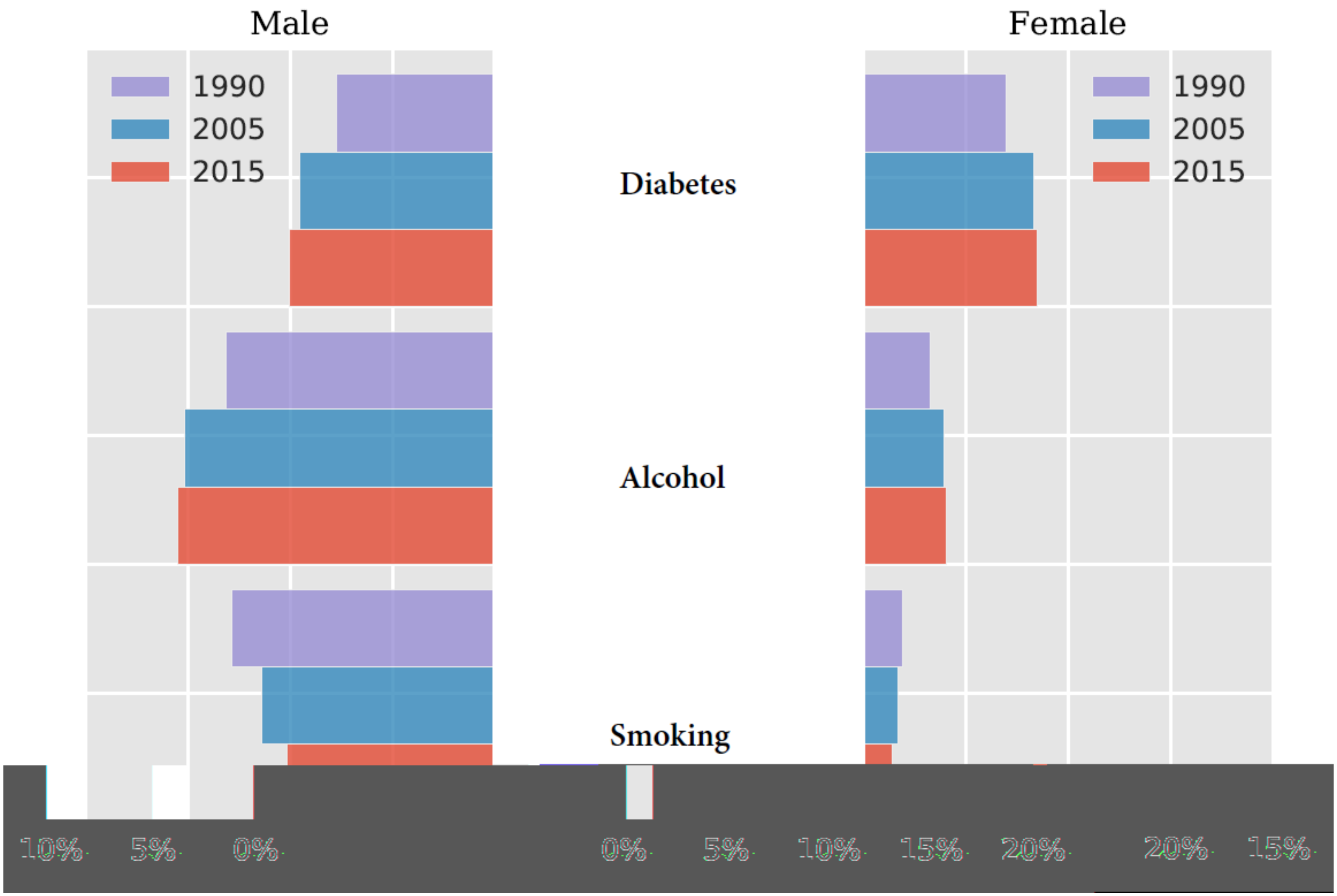

Appendix Tables

eTable 1a. Beta coefficients and exponentiated values from the model using remission calculated based on incidence and prevalence data

Covariate	Parameter	Beta (95% CI)	Exponentiated beta (95% CI)
Smear positive TB	Prevalence	-0.75	0.47 (0.47 – 0.47)
Sex (male)	Prevalence	0.57	1.77 (1.63 – 1.92)
Sex (male)	Incidence	0.40	1.49 (1.47 – 1.51)
CDR 0 to 10%	Incidence	-2.31	0.099 (0.097 – 0.10)
CDR >10 to 20%	Incidence	-1.62	0.20 (0.19 – 0.20)
CDR >20 to 30%	Incidence	-1.2	0.30 (0.30 – 0.30)
CDR >30 to 40%	Incidence	-0.9	0.41 (0.40 – 0.41)
CDR >40 to 50%	Incidence	-0.7	0.50 (0.49 – 0.50)
CDR >50 to 60%	Incidence	-0.5	0.60 (0.60 – 0.61)
CDR >60 to 70%	Incidence	-0.42	0.66 (0.64 – 0.67)
CDR >70 to 80%	Incidence	-0.28	0.76 (0.73 – 0.79)
CDR >80 to 90%	Incidence	-0.2	0.82 (0.82 – 0.82)
Age-standardized SEV scalar (log-transformed)	Prevalence	0.77	2.16 (2.12 – 2.27)
Age-standardized SEV scalar (log-transformed)	Incidence	0.76	2.13 (2.12 – 2.16)
LDI (log-transformed)	Remission	0.11	1.12 (1.06 – 1.22)
LDI (log-transformed)	Excess mortality	-0.50	0.61 (0.61 – 0.61)

eTable 1b. Beta coefficients and exponentiated values from the model applying the remission assumption for data-rich countries

**Covariate**	**Parameter**	**Beta (95% CI)**	**Exponentiated beta (95% CI)**
Smear positive TB	Prevalence	-0.75	0.47 (0.47 – 0.47)
Sex (male)	Prevalence	0.57	1.77 (1.62 – 1.96)
Sex (male)	Incidence	0.39	1.48 (1.47 – 1.49)
CDR 0 to 10%	Incidence	-2.30	0.10 (0.10 – 0.10)
CDR >10 to 20%	Incidence	-1.61	0.20 (0.20 – 0.20)
CDR >20 to 30%	Incidence	-1.20	0.30 (0.30 – 0.30)
CDR >30 to 40%	Incidence	-0.90	0.41 (0.41 – 0.41)
CDR >40 to 50%	Incidence	-0.70	0.50 (0.50 – 0.50)
CDR >50 to 60%	Incidence	-0.50	0.61 (0.60 – 0.61)
CDR >60 to 70%	Incidence	-0.43	0.65 (0.64 – 0.66)
CDR >70 to 80%	Incidence	-0.28	0.75 (0.74 – 0.76)
CDR >80 to 90%	Incidence	-0.20	0.82 (0.82 – 0.82)
Age-standardized SEV scalar (log-transformed)	Prevalence	0.77	2.17 (2.12 – 2.30)
Age-standardized SEV scalar (log-transformed)	Incidence	0.75	2.12 (2.12 – 2.12)
LDI (log-transformed)	Remission	0.11	1.12 (1.07 – 1.20)
LDI (log-transformed)	Excess mortality	-0.50	0.61 (0.61 – 0.61)

eTable 2. Relative risks for the associations between smoking, diabetes, and alcohol use, and tuberculosis

Risk – Outcome	Category	Morbidity/ Mortality	Sex	All Ages	25-29	30-34	35-39	40-44	45-49	50-54	55-59	60-64	65-69	70-74	75-79	80+
**Smoking**																
Tuberculosis	Smoker (5 year lag)	Both	Male			1·588 (1·242 to 2·039)	1·588 (1.242 to 2.039)	1.588 (1.242 to 2.039)	1.588 (1.242 to 2.039)	1.588 (1.242 to 2.039)	1.588 (1.242 to 2.039)	1.588 (1.242 to 2.039)	1.588 (1.242 to 2.039)	1.588 (1.242 to 2.039)	1.588 (1.242 to 2.039)	1.588 (1.242 to 2.039)
Tuberculosis	Nonsmoker (5 year lag)	Both	Male			1.000 (1.000 to 1.000)	1.000 (1.000 to 1.000)	1.000 (1.000 to 1.000)	1.000 (1.000 to 1.000)	1.000 (1.000 to 1.000)	1.000 (1.000 to 1.000)	1.000 (1.000 to 1.000)	1.000 (1.000 to 1.000)	1.000 (1.000 to 1.000)	1.000 (1.000 to 1.000)	1.000 (1.000 to 1.000)
Tuberculosis	Smoker (5 year lag)	Both	Female			1.599 (1.258 to 2.024)	1.599 (1.258 to 2.024)	1.599 (1.258 to 2.024)	1.599 (1.258 to 2.024)	1.599 (1.258 to 2.024)	1.599 (1.258 to 2.024)	1.599 (1.258 to 2.024)	1.599 (1.258 to 2.024)	1.599 (1.258 to 2.024)	1.599 (1.258 to 2.024)	1.599 (1.258 to 2.024)
Tuberculosis	Nonsmoker (5 year lag)	Both	Female			1.000 (1.000 to 1.000)	1.000 (1.000 to 1.000)	1.000 (1.000 to 1.000)	1.000 (1.000 to 1.000)	1.000 (1.000 to 1.000)	1.000 (1.000 to 1.000)	1.000 (1.000 to 1.000)	1.000 (1.000 to 1.000)	1.000 (1.000 to 1.000)	1.000 (1.000 to 1.000)	1.000 (1.000 to 1.000)
**Diabetes**																
Tuberculosis	Diabetic	Both	Both		2.730 (1.972 to 3.604)	2.801 (2.053 to 3.672)	2.871 (2.039 to 3.710)	2.798 (1.963 to 3.630)	2.581 (1.906 to 3.275)	2.364 (1.813 to 2.946)	2.147 (1.684 to 2.677)	1.930 (1.485 to 2.441)	1.713 (1.231 to 2.326)	1.598 (1.123 to 2.242)	1.587 (1.182 to 2.116)	1.559 (1.180 to 2.179)
Tuberculosis	Not diabetic	Both	Both		1.000 (1.000 to 1.000)	1.000 (1.000 to 1.000)	1.000 (1.000 to 1.000)	1.000 (1.000 to 1.000)	1.000 (1.000 to 1.000)	1.000 (1.000 to 1.000)	1.000 (1.000 to 1.000)	1.000 (1.000 to 1.000)	1.000 (1.000 to 1.000)	1.000 (1.000 to 1.000)	1.000 (1.000 to 1.000)	1.000 (1.000 to 1.000)
**Alcohol Use**																
Tuberculosis	Former	Both	Male	1.210 (1.095 to 1.313)												
Tuberculosis	85 g/day	Both	Male	2.960 (2.302 to 3.835)												

Risk – Outcome	Category	Morbidity/ Mortality	Sex	All Ages	25-29	30-34	35-39	40-44	45-49	50-54	55-59	60-64	65-69	70-74	75-79	80+
Tuberculosis	80 g/day	Both	Male	2.960 (2.302 to 3.835)												
Tuberculosis	70 g/day	Both	Male	2.960 (2.302 to 3.835)												
Tuberculosis	60 g/day	Both	Male	2.960 (2.302 to 3.835)												
Tuberculosis	50 g/day	Both	Male	2.960 (2.302 to 3.835)												
**Alcohol Use (continued)**																
Tuberculosis	40 g/day	Both	Male	2.960 (2.302 to 3.835)												
Tuberculosis	30 g/day	Both	Male	1.000 (1.000 to 1.000)												
Tuberculosis	20 g/day	Both	Male	1.000 (1.000 to 1.000)												
Tuberculosis	10 g/day	Both	Male	1.000 (1.000 to 1.000)												
Tuberculosis	0 g/day	Both	Male	1.000 (1.000 to 1.000)												
Tuberculosis	Former	Both	Female	1.440 (1.280 to 1.608)												

**Risk – Outcome**	**Category**	**Morbidity/ Mortality**	**Sex**	**All Ages**	**25-29**	**30-34**	**35-39**	**40-44**	**45-49**	**50-54**	**55-59**	**60-64**	**65-69**	**70-74**	**75-79**	**80+**
Tuberculosis	60 g/day	Both	Female	2.960 (2.253 to 3.851)												
Tuberculosis	50 g/day	Both	Female	2.960 (2.253 to 3.851)												
Tuberculosis	40 g/day	Both	Female	2.960 (2.253 to 3.851)												
Tuberculosis	30 g/day	Both	Female	1.000 (1.000 to 1.000)												
Tuberculosis	20 g/day	Both	Female	1.000 (1.000 to 1.000)												
Tuberculosis	10 g/day	Both	Female	1.000 (1.000 to 1.000)												
Tuberculosis	0 g/day	Both	Female	1.000 (1.000 to 1.000)												

eTable 3. Age-standardized tuberculosis (with and without HIV) incidence, prevalence, and mortality rates and annualized rates of change for both sexes for 21 GBD regions and five SDI categories

	Age-standardized rates in 2015 (per 100,000 population) with 95% uncertainty intervals			Annualized rate of change of age-standardized rate (%) with 95% uncertainty intervals					
				1990–2005			2005–2015		
	**Incidence**	**Prevalence**	**Mortality**	**Incidence**	**Prevalence**	**Mortality**	**Incidence**	**Prevalence**	**Mortality**
Global	137.5 (124.2-150.7)	137.0 (126.2-148.1)	18.9 (15.4-22.4)	-0.5 (-0.7 to -0.3)	-0.3 (-0.4 to -0.1)	-1.8 (-2.4 to -1.4)	-2.0 (-2.3 to -1.6)	-1.2 (-1.4 to -0.9)	-4.6 (-5.4 to -3.9)
High SDI	29.3 (27.0-31.5)	17.0 (15.8-18.1)	1.5 (1.4-1.5)	-1.1 (-1.5 to -0.7)	-0.5 (-0.8 to -0.2)	-0.9 (-1.2 to -0.5)	-3.1 (-3.6 to -2.6)	-3.1 (-3.5 to -2.7)	-7.3 (-8.0 to -6.6)
High-middle SDI	107.8 (96.8-118.9)	100.6 (91.9-109.4)	7.8 (6.6-9.0)	0.7 (0.4 to 1.0)	0.8 (0.5 to 1.0)	-1.3 (-2.1 to -0.7)	-11.7 (-12.3 to -11.1)	-10.5 (-11.0 to -10.1)	-6.0 (-6.6 to -5.2)
Middle SDI	175.9 (159.3-192.7)	196.6 (181.2-212.2)	19.4 (15.3-23.4)	-1.3 (-1.5 to -1.0)	-1.1 (-1.3 to -1.0)	-3.5 (-4.4 to -2.7)	-2.1 (-3.3 to -0.8)	-1.1 (-2.2 to -0.1)	-5.3 (-6.4 to -4.3)
Low-middle SDI	211.6 (191.2-231.6)	208.8 (189.9-228.2)	48.5 (38.4-58.4)	-1.8 (-2.7 to -0.9)	-1.2 (-2.1 to -0.3)	-2.6 (-3.5 to -2.0)	-2.2 (-3.5 to -0.9)	-1.5 (-2.8 to -0.4)	-4.4 (-5.7 to -3.4)
Low SDI	247.9 (229.6-265.9)	215.6 (200.9-230.4)	86.6 (63.0-109.9)	-4.0 (-4.2 to -3.8)	-4.1 (-4.3 to -4.0)	-0.1 (-1.3 to 1.2)	3.6 (3.3 to 3.9)	3.8 (3.6 to 4.1)	-4.2 (-5.9 to -2.5)
High-income Asia Pacific	31.7 (29.1-34.3)	15.4 (14.0-16.8)	1.8 (1.7-1.9)	-5.1 (-5.5 to -4.8)	-5.3 (-5.7 to -5.0)	-6.4 (-6.8 to -6.1)	-1.4 (-2.0 to -0.8)	-1.4 (-2.1 to -0.9)	-4.6 (-5.3 to -4.0)
Central Asia	98.5 (90.1-106.8)	76.2 (70.0-82.3)	7.7 (6.1-9.2)	0.2 (-0.1 to 0.5)	0.7 (0.5 to 0.9)	1.8 (-0.3 to 2.7)	-3.4 (-4.0 to -2.9)	-2.8 (-3.3 to -2.3)	-6.9 (-7.7 to -6.0)
East Asia	100.5 (89.2-111.9)	127.5 (116.1-138.9)	3.6 (2.5-4.8)	-0.9 (-1.4 to -0.4)	-1.1 (-1.4 to -0.7)	-6.6 (-7.8 to -4.4)	-1.8 (-2.2 to -1.3)	-0.4 (-0.7 to 0.0)	-7.5 (-8.7 to -5.8)
South Asia	212.3 (187.6-237.6)	218.9 (194.8-242.9)	45.9 (36.1-55.9)	-2.4 (-2.6 to -2.1)	-1.7 (-1.9 to -1.5)	-3.3 (-4.0 to -2.6)	-2.8 (-3.2 to -2.2)	-1.6 (-1.9 to -1.2)	-4.8 (-6.2 to -3.8)
Southeast Asia	227.7 (209.9-246.4)	250.1 (233.8-266.1)	37.5 (28.8-46.2)	-2.5 (-2.8 to -2.2)	-2.4 (-2.6 to -2.2)	-3.7 (-4.9 to -2.6)	-1.5 (-1.9 to -1.0)	-1.1 (-1.4 to -0.8)	-4.5 (-6.2 to -3.0)
Australasia	7.7 (6.2-9.3)	3.9 (3.1-4.7)	0.2 (0.2-0.2)	-2.2 (-3.0 to -1.6)	-2.2 (-3.0 to -1.6)	-5.6 (-6.2 to -5.0)	0.2 (-0.4 to 1.0)	0.4 (-0.3 to 1.1)	-4.0 (-5.0 to -3.0)

Caribbean	41.6 (38.5-44.7)	26.3 (24.3-28.3)	5.0 (3.4-6.7)	-2.0 (-2.3 to -1.8)	-2.0 (-2.2 to -1.8)	-2.8 (-4.2 to -0.9)	-1.6 (-2.0 to -1.2)	-1.6 (-2.1 to -1.2)	-3.6 (-4.9 to -2.3)
Central Europe	32.5 (29.9-35.1)	16.5 (15.2-17.9)	1.5 (1.3-1.6)	-1.8 (-2.0 to -1.5)	-1.7 (-1.9 to -1.5)	-3.4 (-3.8 to -3.0)	-2.9 (-3.3 to -2.4)	-2.7 (-3.1 to -2.3)	-6.5 (-7.2 to -5.6)
Eastern Europe	124.9 (115.5-134.3)	67.8 (63.5-72.0)	6.6 (6.1-7.0)	2.8 (2.3 to 3.2)	3.8 (3.3 to 4.2)	6.0 (5.5 to 6.5)	-3.5 (-4.1 to -2.7)	-4.0 (-4.5 to -3.4)	-8.3 (-9.2 to -7.4)
Western Europe	11.2 (9.2-13.1)	5.6 (4.6-6.6)	0.5 (0.5-0.5)	-5.3 (-5.7 to -4.9)	-5.4 (-5.8 to -5.0)	-6.5 (-6.8 to -6.1)	-1.8 (-2.4 to -1.3)	-1.5 (-2.1 to -0.9)	-4.9 (-5.4 to -4.3)
Andean Latin America	79.3 (70.6-87.8)	52.5 (46.8-58.2)	8.9 (5.3-12.6)	-6.7 (-7.0 to -6.3)	-6.9 (-7.2 to -6.6)	-7.8 (-9.7 to -2.5)	-2.4 (-2.9 to -1.7)	-2.1 (-2.8 to -1.5)	-4.7 (-5.8 to -3.8)
Central Latin America	29.9 (28.0-31.8)	14.3 (13.3-15.3)	3.1 (2.9-3.3)	-4.2 (-4.4 to -4.0)	-4.1 (-4.3 to -3.9)	-6.7 (-7.0 to -6.3)	-2.5 (-2.9 to -2.0)	-2.4 (-2.8 to -2.0)	-4.6 (-5.1 to -4.1)
Southern Latin America	37.9 (35.5-40.4)	20.9 (19.6-22.2)	3.0 (2.9-3.2)	-2.7 (-3.2 to -2.3)	-1.8 (-2.1 to -1.4)	-3.1 (-3.4 to -2.8)	-1.8 (-2.4 to -1.2)	-2.1 (-2.6 to -1.6)	-3.2 (-3.8 to -2.6)
Tropical Latin America	42.6 (38.7-46.6)	30.6 (27.5-33.7)	3.9 (3.0-4.9)	-0.9 (-1.4 to -0.5)	-1.1 (-1.5 to -0.7)	-3.4 (-4.7 to -2.5)	-1.3 (-1.7 to -1.0)	-1.2 (-1.6 to -0.8)	-3.8 (-4.8 to -2.5)
North Africa and Middle East	38.5 (34.1-42.8)	27.6 (24.5-30.5)	5.1 (3.8-6.5)	-2.8 (-2.9 to -2.7)	-3.0 (-3.1 to -2.8)	-3.3 (-4.3 to -2.0)	-0.9 (-1.3 to -0.6)	-0.8 (-1.2 to -0.4)	-3.5 (-4.5 to -2.4)
High-income North America	4.1 (3.5-4.8)	2.2 (1.8-2.5)	0.2 (0.2-0.2)	-6.1 (-6.8 to -5.3)	-6.0 (-6.8 to -5.3)	-7.9 (-8.2 to -7.6)	-2.4 (-2.9 to -2.0)	-2.3 (-2.9 to -1.8)	-3.8 (-4.3 to -3.3)
Oceania	74.4 (67.1-81.8)	72.8 (66.4-79.3)	12.1 (7.3-16.9)	-0.7 (-1.0 to -0.5)	-0.9 (-1.1 to -0.7)	-2.1 (-3.8 to -0.3)	-0.1 (-0.7 to 0.6)	0.2 (-0.3 to 0.8)	-3.4 (-5.4 to -1.3)
Central Sub-Saharan Africa	320.3 (290.7-350.4)	263.1 (238.1-287.7)	103.5 (30.0-176.1)	-0.1 (-0.4 to 0.1)	-0.6 (-0.9 to -0.4)	0.3 (-2.8 to 3.5)	-1.5 (-1.9 to -1.1)	-1.4 (-1.8 to -1.0)	-3.4 (-6.9 to -0.5)
Eastern Sub-Saharan Africa	278.5 (259.2-298.0)	237.6 (224.2-251.1)	79.7 (58.5-100.8)	0.8 (0.5 to 1.1)	0.8 (0.6 to 1.0)	0.2 (-1.3 to 1.3)	-3.1 (-3.5 to -2.6)	-2.9 (-3.2 to -2.5)	-5.3 (-7.5 to -3.2)
Southern Sub-Saharan Africa	1,506.3 (1,338.9-1,675.3)	1,330.5 (1,205.9-1,454.2)	161.1 (136.0-186.0)	7.7 (7.0 to 8.3)	7.9 (7.4 to 8.4)	7.3 (4.7 to 9.0)	-3.0 (-3.6 to -2.2)	-3.0 (-3.5 to -2.5)	-6.3 (-7.6 to -4.7)
Western Sub-Saharan Africa	178.7 (163.3-194.1)	164.7 (151.7-177.8)	46.2 (32.1-60.7)	-0.0 (-0.2 to 0.1)	-0.0 (-0.2 to 0.1)	-1.2 (-2.4 to -0.1)	-1.3 (-1.8 to -0.9)	-1.3 (-1.7 to -0.9)	-3.6 (-5.1 to -2.1)

eTable 4. Tuberculosis DALYs (in HIV-negative individuals) attributable to smoking, alcohol use, and diabetes and annualized rate of change (2005–2015) for 21 Global Burden of Disease regions

	Tuberculosis DALYs (all ages, both sexes) attributable to each risk factor (95% UI)						Annualized rate of change in DALYs attributable to each risk factor (2005–2015) (%) (95% UI)		
	Smoking		Alcohol use		Diabetes				
	2005	2015	2005	2015	2005	2015	Smoking	Alcohol use	Diabetes
Global	3,717,425 (1,859,497 to 5,830,354)	2,835,620 (1,356,976 to 4,514,191)	5,498,279 (4,496,910 to 6,911,424)	4,724,643 (3,591,134 to 6,197,917)	4,313,417 (2,737,185 to 5,959,512)	3,801,840 (2,430,311 to 5,303,540)	-4.9 (-5.9 to -4.1)	-3.5 (-4.5 to -2.6)	-3.5 (-4.5 to -2.8)
High SDI	235,229 (120,235 to 345,291)	107,221 (54,248 to 159,112)	478,473 (412,409 to 534,376)	229,078 (200,776 to 260,389)	92,868 (58,242 to 129,693)	53,090 (33,637 to 74,193)	-8.9 (-10.0 to -7.8)	-8.4 (-9.5 to -7.3)	-7.1 (-8.2 to -6.0)
High-middle SDI	446,252 (221,611 to 695,845)	295,173 (144,158 to 467,908)	855,809 (680,235 to 998,623)	674,642 (519,054 to 807,844)	518,674 (322,875 to 710,181)	408,829 (258,749 to 563,868)	-6.4 (-7.2 to -5.6)	-4.4 (-5.3 to -3.6)	-4.8 (-5.6 to -4.0)
Middle SDI	1,348,642 (655,849 to 2,182,381)	1,044,948 (501,434 to 1,718,701)	1,516,288 (1,173,992 to 1,916,166)	1,228,867 (939,850 to 1,596,519)	1,522,242 (976,723 to 2,118,654)	1,264,862 (793,465 to 1,812,499)	-4.9 (-6.4 to -3.7)	-4.2 (-5.2 to -3.2)	-4.3 (-5.6 to -3.3)
Low-middle SDI	1,425,324 (717,234 to 2,265,307)	1,122,405 (509,435 to 1,820,030)	2,014,442 (1,602,490 to 2,683,181)	1,910,622 (1,393,635 to 2,589,363)	1,802,185 (1,147,045 to 2,487,516)	1,637,194 (1,029,699 to 2,287,454)	-5.0 (-7.0 to -3.6)	-3.0 (-4.7 to -1.6)	-3.5 (-5.0 to -2.4)
Low SDI	259,913 (112,831 to 443,984)	264,117 (112,678 to 464,499)	631,630 (456,178 to 867,595)	679,886 (426,150 to 982,554)	375,281 (216,434 to 563,709)	435,670 (253,139 to 686,398)	-3.1 (-5.7 to -0.6)	-2.5 (-5.4 to 0.2)	-1.7 (-3.9 to 0.5)
High-income Asia Pacific	21,717 (11,354 to 32,417)	11,596 (5,777 to 17,571)	19,959 (15,951 to 23,951)	12,797 (9,852 to 15,892)	12,820 (7,710 to 18,687)	9,231 (5,503 to 13,802)	-7.9 (-8.8 to -7.0)	-5.7 (-7.1 to -4.4)	-5.2 (-6.3 to -4.2)
Central Asia	47,009 (22,891 to 72,448)	29,553 (13,927 to 46,041)	89,928 (70,518 to 102,825)	57,119 (40,268 to 69,705)	25,944 (15,820 to 36,884)	19,590 (11,967 to 27,857)	-6.6 (-7.7 to -5.3)	-6.6 (-8.2 to -5.3)	-5.2 (-6.2 to -4.3)

East Asia	398,336 (193,416 to 628,847)	246,761 (113,953 to 421,149)	461,623 (363,928 to 623,997)	346,527 (275,076 to 498,612)	280,358 (174,422 to 397,806)	173,934 (103,303 to 264,753)	-7.0 (-8.3 to -5.5)	-5.0 (-6.3 to -3.5)	-7.2 (-8.4 to -5.7)
South Asia	1,514,450 (715,187 to 2,450,924)	1,160,246 (516,409 to 1,891,349)	2,409,374 (1,905,273 to 3,124,929)	2,189,746 (1,590,518 to 2,930,183)	2,309,423 (1,472,900 to 3,173,825)	1,948,027 (1,227,955 to 2,740,549)	-5.3 (-7.2 to -4.1)	-3.4 (-5.1 to -2.2)	-4.3 (-5.7 to -3.3)
Australasia	154 (91 to 222)	106 (62 to 153)	365 (321 to 412)	316 (272 to 367)	150 (90 to 219)	134 (82 to 197)	-5.6 (-6.5 to -4.8)	-3.4 (-4.6 to -2.3)	-3.4 (-4.3 to -2.5)
Caribbean	3,778 (1,858 to 6,205)	2,589 (1,240 to 4,550)	10,167 (7,632 to 15,544)	9,944 (6,969 to 16,295)	5,214 (3,064 to 7,773)	5,265 (3,011 to 8,272)	-5.6 (-7.7 to -3.6)	-1.6 (-3.6 to 0.3)	-1.9 (-3.5 to -0.4)
Central Europe	20,423 (10,395 to 30,124)	10,956 (5,605 to 16,380)	38,962 (35,618 to 42,657)	21,834 (19,321 to 24,908)	9,429 (5,923 to 13,378)	5,903 (3,640 to 8,374)	-6.8 (-7.9 to -5.6)	-6.2 (-7.5 to -4.9)	-5.4 (-6.4 to -4.2)
Eastern Europe	259,083 (131,012 to 379,729)	111,854 (56,663 to 165,666)	560,269 (493,752 to 619,994)	25,3676 (223,963 to 286,759)	84,784 (52,955 to 117,650)	40,382 (24,716 to 56,545)	-8.6 (-9.6 to -7.5)	-8.1 (-9.2 to -7.1)	-7.9 (-9.2 to -6.6)
Andean Latin America	9,438 (4,683 to 18,106)	6,483 (3,140 to 12,526)	26,879 (20,967 to 49,033)	21,310 (15,576 to 40,461)	8,559 (4,979 to 14,188)	7,701 (4,324 to 13,684)	-6.5 (-8.2 to -4.8)	-4.6 (-6.3 to -3.0)	-3.8 (-5.0 to -2.7)
Central Latin America	12,844 (6,670 to 19,541)	8,964 (4,355 to 14,096)	34,873 (31,758 to 38,382)	29,103 (24,961 to 33,336)	24,046 (15,841 to 32,463)	21,923 (14,698 to 29,526)	-6.7 (-7.5 to -5.8)	-4.7 (-5.6 to -3.9)	-4.1 (-4.7 to -3.5)
North Africa and Middle East	44,620 (22,273 to 72,092)	40,525 (20,213 to 67,620)	23,825 (18,055 to 29,881)	21,740 (15,327 to 28,848)	83,790 (55,000 to 118,162)	88,073 (57,102 to 123,621)	-4.3 (-5.8 to -3.0)	-3.9 (-5.5 to -2.7)	-2.8 (-4.0 to -1.5)
High-income North America	2,397 (1,359 to 3,463)	1,702 (982 to 2,476)	5,096 (4,580 to 5,638)	4,175 (3,465 to 4,849)	3,288 (2,117 to 4,561)	3,080 (1,961 to 4,261)	-5.1 (-5.6 to -4.6)	-3.5 (-4.4 to -2.7)	-2.7 (-3.2 to -2.1)
Oceania	3,222 (1,589 to 5,487)	3,065 (1,474 to 5,551)	2,085 (1,173 to 3,383)	1,982 (1,097 to 3,554)	4,640 (2,690 to 7,087)	4,717 (2,734 to 7,538)	-3.4 (-6.2 to -0.5)	-2.9 (-6.2 to 0.4)	-2.7 (-5.0 to -0.3)

Central sub-Saharan Africa	67,182 (22,151 to 173,456)	75,994 (23,151 to 201,732)	119,952 (46,335 to 269,913)	136,784 (49,827 to 318,598)	121,631 (52,369 to 251,237)	145,264 (59,588 to 337,605)	-2.1 (-7.4 to 2.8)	-1.8 (-6.6 to 2.7)	-1.3 (-5.8 to 2.4)
Eastern sub-Saharan Africa	175,895 (78,937 to 296,036)	171,999 (70,694 to 308,271)	514,484 (381,743 to 663,083)	558,976 (354,320 to 796,402)	206,555 (124,064 to 301,461)	232,864 (129,553 to 371,948)	-3.6 (-6.9 to -0.7)	-2.5 (-5.9 to 0.7)	-2.1 (-5.3 to 0.8)

Note. Total HIV-negative tuberculosis DALYs in 2005 and 2015 were 49,769,565 (43,196,422 to 60,348,200) and 40,302,237 (34,065,779 to 49,653,867) respectively.

eTable 5. Mean differences and 95% uncertainty intervals for population attributable fractions of risk factors for global tuberculosis deaths

Risk factor pairs	Mean difference (95% uncertainty interval)
Alcohol and diabetes	0.88 (-3.38 to 5.31)
Alcohol and smoking	3.72 (-0.94 to 8.16)
Diabetes and smoking	2.84 (-2.81 to 8.45)

eTable 6. List of data-rich locations

Location name	Region name
Australia	Australasia
New Zealand	Australasia
Antigua and Barbuda	Caribbean
Barbados	Caribbean
Bermuda	Caribbean
Cuba	Caribbean
Puerto Rico	Caribbean
Saint Lucia	Caribbean
Saint Vincent and the Grenadines	Caribbean
Trinidad and Tobago	Caribbean
Kazakhstan	Central Asia
Bulgaria	Central Europe
Croatia	Central Europe
Czech Republic	Central Europe
Hungary	Central Europe
Poland	Central Europe
Romania	Central Europe
Slovenia	Central Europe
Aguascalientes	Central Latin America

**Location name**	**Region name**
Baja California	Central Latin America
Baja California Sur	Central Latin America
Campeche	Central Latin America
Chiapas	Central Latin America
Chihuahua	Central Latin America
Coahuila	Central Latin America
Colima	Central Latin America
Colombia	Central Latin America
Costa Rica	Central Latin America
Distrito Federal	Central Latin America
Durango	Central Latin America
Guanajuato	Central Latin America
Guatemala	Central Latin America
Guerrero	Central Latin America
Hidalgo	Central Latin America
Jalisco	Central Latin America
Michoacán de Ocampo	Central Latin America
Morelos	Central Latin America
México	Central Latin America
Nayarit	Central Latin America
Nuevo León	Central Latin America
Oaxaca	Central Latin America
Puebla	Central Latin America
Querétaro	Central Latin America
Quintana Roo	Central Latin America
San Luis Potosí	Central Latin America
Sinaloa	Central Latin America
Sonora	Central Latin America
Tabasco	Central Latin America
Tamaulipas	Central Latin America

Location name	Region name
Tlaxcala	Central Latin America
Venezuela	Central Latin America
Veracruz de Ignacio de la Llave	Central Latin America
Yucatán	Central Latin America
Zacatecas	Central Latin America
Estonia	Eastern Europe
Latvia	Eastern Europe
Lithuania	Eastern Europe
Moldova	Eastern Europe
Russia	Eastern Europe
Ukraine	Eastern Europe
Aichi	High-income Asia Pacific
Akita	High-income Asia Pacific
Aomori	High-income Asia Pacific
Chiba	High-income Asia Pacific
Ehime	High-income Asia Pacific
Fukui	High-income Asia Pacific
Fukuoka	High-income Asia Pacific
Fukushima	High-income Asia Pacific
Gifu	High-income Asia Pacific
Gunma	High-income Asia Pacific
Hiroshima	High-income Asia Pacific
Hokkaidō	High-income Asia Pacific
Hyōgo	High-income Asia Pacific
Ibaraki	High-income Asia Pacific
Ishikawa	High-income Asia Pacific
Iwate	High-income Asia Pacific
Kagawa	High-income Asia Pacific
Kagoshima	High-income Asia Pacific
Kanagawa	High-income Asia Pacific

**Location name**	**Region name**
Kumamoto	High-income Asia Pacific
Kyōto	High-income Asia Pacific
Kōchi	High-income Asia Pacific
Mie	High-income Asia Pacific
Miyagi	High-income Asia Pacific
Miyazaki	High-income Asia Pacific
Nagano	High-income Asia Pacific
Nagasaki	High-income Asia Pacific
Nara	High-income Asia Pacific
Niigata	High-income Asia Pacific
Okayama	High-income Asia Pacific
Okinawa	High-income Asia Pacific
Saga	High-income Asia Pacific
Saitama	High-income Asia Pacific
Shiga	High-income Asia Pacific
Shimane	High-income Asia Pacific
Shizuoka	High-income Asia Pacific
Singapore	High-income Asia Pacific
South Korea	High-income Asia Pacific
Tochigi	High-income Asia Pacific
Tokushima	High-income Asia Pacific
Tottori	High-income Asia Pacific
Toyama	High-income Asia Pacific
Tōkyō	High-income Asia Pacific
Wakayama	High-income Asia Pacific
Yamagata	High-income Asia Pacific
Yamaguchi	High-income Asia Pacific
Yamanashi	High-income Asia Pacific
Ōita	High-income Asia Pacific
Ōsaka	High-income Asia Pacific

**Location name**	**Region name**
Alabama	High-income North America
Alaska	High-income North America
Arizona	High-income North America
Arkansas	High-income North America
California	High-income North America
Canada	High-income North America
Colorado	High-income North America
Connecticut	High-income North America
Delaware	High-income North America
District of Columbia	High-income North America
Florida	High-income North America
Georgia	High-income North America
Hawaii	High-income North America
Idaho	High-income North America
Illinois	High-income North America
Indiana	High-income North America
Iowa	High-income North America
Kansas	High-income North America
Kentucky	High-income North America
Louisiana	High-income North America
Maine	High-income North America
Maryland	High-income North America
Massachusetts	High-income North America
Michigan	High-income North America
Minnesota	High-income North America
Mississippi	High-income North America
Missouri	High-income North America
Montana	High-income North America
Nebraska	High-income North America
Nevada	High-income North America

Location name	Region name
New Hampshire	High-income North America
New Jersey	High-income North America
New Mexico	High-income North America
New York	High-income North America
North Carolina	High-income North America
North Dakota	High-income North America
Ohio	High-income North America
Oklahoma	High-income North America
Oregon	High-income North America
Pennsylvania	High-income North America
Rhode Island	High-income North America
South Carolina	High-income North America
South Dakota	High-income North America
Tennessee	High-income North America
Texas	High-income North America
Utah	High-income North America
Vermont	High-income North America
Virginia	High-income North America
Washington	High-income North America
West Virginia	High-income North America
Wisconsin	High-income North America
Wyoming	High-income North America
Kuwait	North Africa and Middle East
Mauritius	Southeast Asia
Sri Lanka	Southeast Asia
Argentina	Southern Latin America
Chile	Southern Latin America
Uruguay	Southern Latin America
Austria	Western Europe
Belgium	Western Europe

**Location name**	**Region name**
Denmark	Western Europe
East Midlands	Western Europe
East of England	Western Europe
Finland	Western Europe
France	Western Europe
Germany	Western Europe
Greater London	Western Europe
Greece	Western Europe
Iceland	Western Europe
Ireland	Western Europe
Israel	Western Europe
Italy	Western Europe
Luxembourg	Western Europe
Malta	Western Europe
Netherlands	Western Europe
North East England	Western Europe
North West England	Western Europe
Northern Ireland	Western Europe
Norway	Western Europe
Portugal	Western Europe
Scotland	Western Europe
South East England	Western Europe
South West England	Western Europe
Spain	Western Europe
Stockholm	Western Europe
Sweden except Stockholm	Western Europe
Switzerland	Western Europe
Wales	Western Europe
West Midlands	Western Europe
Yorkshire and the Humber	Western Europe

Comparative Risk Assessment

Alcohol

Flowchart

Alcohol Use

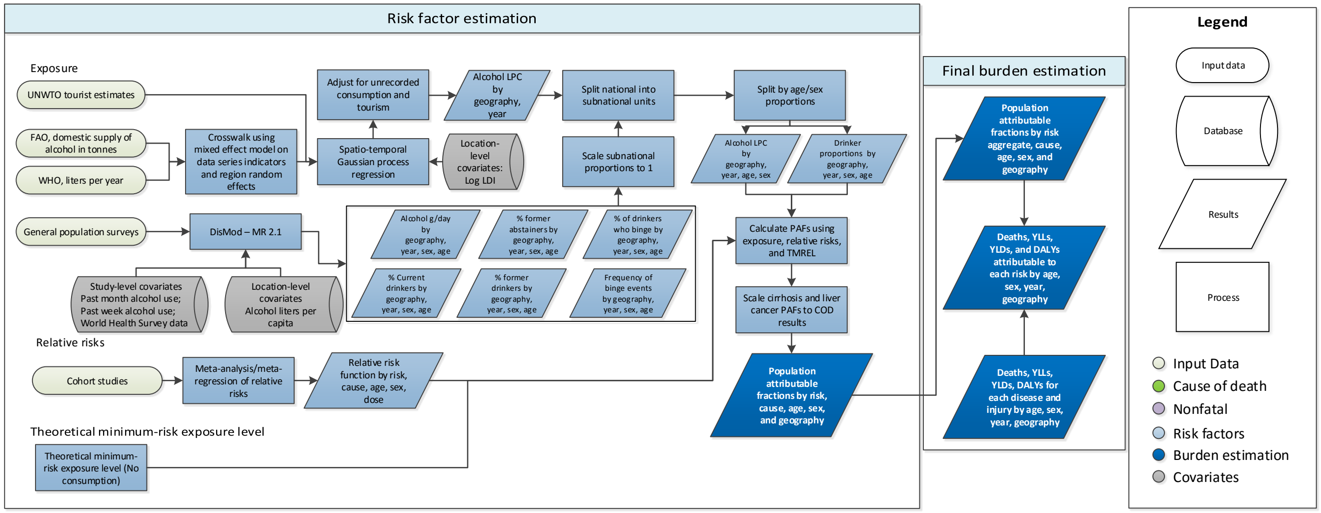

Input Data and Methodological Summary

Exposure

Case definition

The impact of alcohol consumption on morbidity and mortality can be largely described by two separate but related dimensions. The 1st dimension is the individual level drinking and consists of four indicators;

1. Current drinkers, defined as the proportion of individuals who have consumed at least one alcoholic beverage (or some approximation) in the last 12 months.

2. Former drinkers, defined as the proportion of individuals who have ever consumed an alcoholic beverage, but not in the last 12 months.
3. Lifetime abstainers, defined as the proportion of individuals who have never consumed an alcoholic beverage.
4. Alcohol consumption (in grams per day), defined as grams of alcohol consumed by current drinkers, per day, over a 12 month period.

The 2nd dimension of alcohol consumption relates to the pattern of drinking and consists of two indicators;

5. Binge drinkers, defined as the proportion of drinkers who have had a binge event in the past 12 months. A binge event was defined as consuming 60 grams of alcohol (approximately five drinks or more) in a single occasion for males and 48 grams of alcohol in a single occasion for females.
6. Binge times, defined as the proportion of drinking events that are binge amongst binge drinkers i.e. the proportion of days that a binger has a binge event.

Input data

For GBD 2013, a systematic review of the literature was conducted to capture population survey data on all six alcohol use indicators. In summary, the search was conducted in three stages involving electronic searches of the peer-reviewed literature via PubMed, the grey literature and, expert consultation. Updates to systematic reviews via PubMed are performed on an ongoing schedule across all GBD causes and risk factors, an update for alcohol use will be performed in the next 1-2 iterations. For GBD 2015, stages two and three of the literature review were conducted, prioritizing countries for which subnational estimates were generated. The Global Health Exchange (GHDx), IHME's online database of health-related data, was searched for population survey data containing participant-level information from which we could formulate the required alcohol use indicators. Data-sources were included if they captured a sample representative of the geographic location under study and contained variables that could be used to formulate any of the six alcohol use indicators. Relevant survey variables from each data-source were documented in a Microsoft Excel codebook and extracted using STATA 13.1. A total of 629 potential data-sources were available in GhDx across countries with subnational locations, out of which 127 data-sources (66,108 data-points) were included across all six indicators.

To generate estimates of alcohol consumption in grams per day, data from population surveys were used in combination with estimates of per capita consumption from the Food and Agriculture Organization (FAO) [1] and the Global Information System on Alcohol and Health (GISAH database [2]) Per capita consumption is an aggregate measure of recorded, unrecorded, and tourist per capita consumption of alcohol (UNWTO database [3]) derived from sales, production, and other economic statistics. While population-based surveys provide accurate estimates of the prevalence of lifetime abstainers, former drinkers and current drinkers, they typically underestimate real alcohol consumption levels. As a result, the all-age, both-sex per capita consumption figures from the FAO and GISAH are considered to be a better estimate of overall volume of consumption. Per capita consumption, however, does not provide age- and sex-specific consumption estimates needed to compute alcohol-attributable burden of disease. Therefore, we use the age-sex pattern of consumption among drinkers modeled from the population survey data and the overall volume of consumption from FAO and GISAH to determine the total amount of alcohol consumed by country.

To generate estimates of alcohol consumption in liter per capita, raw inputs were obtained from FAOSTAT [1] and WHO GISAH database [2]. To provide more stable time trends in the model, FAO sales data was transformed to a lagged 5-year average. FAO data was used when WHO data wasn't available. Otherwise, FAO and WHO data was adjusted (crosswalked) by running a mixed effect model on the log average of the data with indicators for the FAO and WHO data series, as well as random effects on super region, region, country, and time. Each data point was adjusted by the predicted betas on super-region and region.

$$Log\ Average\ Data = D + (Super\ Region\ |D, Region\ |\ D, Country\ |D, Year|\ D)$$

$$Transformed\ data = \ data\ *\ e^{\widehat{\beta_1} + \widehat{\beta_3}}$$

Where D = Indicator variable for data source

To generate uncertainty, a Lowess model was run on the adjusted data and the standard deviation between the difference of the Lowess smoothed model and the adjusted data points was used for data points missing uncertainty.

Unrecorded consumption was incorporated into the alcohol LPC data using estimates provided by the WHO [4]. WHO estimates were only reported for the years 1990, 2005, and 2010 so for missing years, estimates were interpolated. For years outside this range, unrecorded estimates were carried forward or backwards from the closest year. Unrecorded consumption estimates were reported in liters per capita so estimates were added to adjusted data points to account for unrecorded consumption.

Tourism data was obtained through the UNWTO [4]. A crosswalk was applied across different tourist categories, similar to the one used for FAO and WHO data, to estimate tourist proportions for a given country. Tourism consumption was incorporated after modeling unadjusted alcohol LPC as outlined below.

Modeling strategy

DisMod-MR 2.1 was used to estimate country-, year-, age- and sex-specific proportions of current drinkers, former drinkers, lifetime abstainers, binge drinkers, and binge times; and alcohol consumption as a continuous variable in grams per day. We have made no substantive changes in the modeling strategy from GBD 2013. We ran single-parameter models for each alcohol use indicator and included a combination of location- and study-level covariates in each model. An alcohol liters per capita location-level covariate was used for all six indicators to assist in the predictive power of the models. Additionally, study-level covariates were used to accommodate for known sources of variability in the raw data. In the current drinkers, former drinkers, binge drinkers and binge times models, we included two covariates which adjusted estimates derived in the past week and past month towards those derived in the past year respectively. Estimates derived in the past year were considered to be the gold standard given the previously outlined definition for each indicator.

In the alcohol consumption model, we included a separate study-level covariate flagging data points derived from The World Health Organization's World Health Surveys (WHS) conducted across multiple countries. There was considerable variability in estimates derived from the WHS which may have been influenced by methodological differences in how alcohol use was captured. This study-level covariate looked for unsystematic bias between data-points and added more

uncertainty onto those from the WHS. If other data-points causing higher or lower modelled output were identified during the modelling process for a given indicator, the plausibility of these data points was assessed and the study methodology reviewed. Data points with methodological limitations, for instance those derived from survey items not entirely representatively of the alcohol use indicators required, with small sample sizes, or derived from samples not entirely representative of the general population were excluded.

A spatial-temporal Gaussian process regression was used to model total alcohol in liters per capita (see appendix, section 2). Parameters and a random effect model for the prior were chosen using out-of-sample cross validation. This produced estimates of alcohol LPC for a complete time series for the years 1980-2015 by country.

Alcohol LPC was adjusted for each country hosting tourists using the following equations:

$$Alcohol\ LPC_H = Unadjusted\ Alcohol\ LPC_H + Alcohol\ LPC_{Consumption\ abroad} - Alcohol\ LPC_{Tourist\ consumption}$$

$$Alcohol\ LPC_{Consumption\ abroad} = \frac{\sum_V Proportion\ of\ tourists_{H,V} * Unadjusted\ Alcohol\ LPC_H * \frac{Average\ length\ of\ stay_{H,V}}{365} * Tourist\ Population_V}{Population_H}$$

$$Alcohol\ LPC_{Tourist\ consumption} = \frac{\sum_V Proportion\ of\ tourists_V * Unadjusted\ Alcohol\ LPC_V * \frac{Average\ length\ of\ stay_V}{365} * Tourist\ Population_H}{Population_H}$$

Where H = Host country, V = Visiting country

Or, in other words, alcohol LPC was adjusted by adding in the per capita rate of consumption abroad and subtracting the per capita rate of tourist consumption domestically.

After adjusting alcohol LPC by tourist consumption and unrecorded consumption for all location/years reported, sex-specific and age-specific estimates were generated by incorporating estimates modeled in Dismod for percentage of current drinkers within a location/year/sex/age, as well as consumption trends modeled in Dismod g/day by location/year/sex/age, using the following equations.

$$Proportion\ of\ total\ consumption_{l,y,s,a} = \frac{Alcohol\ g/day_{l,y,s,a} * Population_{l,y,s,a} * \%\ Current\ drinkers_{l,y,s,a}}{\sum_{s,a} Alcohol\ g/day_{l,y,s,a} * Population_{l,y,s,a} * \%\ Current\ drinkers_{l,y,s,a}}$$

$$Alcohol\ LPC_{l,y,s,a} = \frac{Alcohol\ LPC_{l,y} * Population_{l,y} * Proportion\ of\ total\ consumption_{l,y,s,a}}{Population_{l,y,s,a}}$$

Where L = location, Y = Year, S = Sex, A = Age

A similar scalar was applied so that total subnational consumption equaled national consumption.

Theoretical minimum-risk exposure level

For alcohol use, the theoretical minimum-risk exposure level (TMREL) was assumed to be no alcohol use, i.e. 0 g/day of alcohol consumption. This diverges from the definition of other theoretical minimum-risk exposure level of risks because, for some alcohol-use relative risks, there's a preventative effect for low levels of consumption. However, due to the modeling of alcohol relative risks outlined below, it was found that 0 g/day provided the most consistency between the definition of alcohol-use TMREL and other GBD risk's TMREL. This is an area of improvement for future GBD iterations. Current research suggests that the preventative effect noted in studies may be due to issues in estimating abstainer populations. [5-7] If this is the case, a TMREL of 0 would still be valid.

Relative Risks

Relative risks were derived for each GBD cause by mapping functions to the dose-response relationships found in meta-analysis. [11-22] Due to data availability, for high levels of consumption, uncertainty in the relative risk functions increases greatly. To minimize the uncertainty of these measures, relative risks were estimated up to the 90th percentile of exposures in men (85 g/day) and the 95th percentile of exposures in women (60 g/day). For exposures beyond this, the associated relative risk was carried forward from these chosen percentile exposure levels. Though a dose-response relationship is evident at higher levels of exposure, the shape of the relative risk function is highly uncertain for higher levels of exposure both due to a lack of observations at these exposure levels, as well as confounding variables affecting estimation of the relative risk of these populations. Thus, our relative risk estimates are likely an underestimate for the top 10% of male exposures and 5% of female exposures.

Population Attributable Fraction

For chronic conditions, PAF was defined as

$$PAF(x) = \frac{P_A + P_F * RR_F + \int_0^{150} P(x) * RR_C(x)\,dx - 1}{P_A + P_F * RR_F + \int_0^{150} P(x) * RR_C(x)\,dx} \qquad P(x) = P_C * \frac{\Gamma(k,\theta)}{\int_{0.1}^{150} \Gamma(k,\theta)}$$

where:

x = alcohol consumption in g/day

P_A = Prevalence of lifetime abstainers

P_F = Prevalence of former drinkers

P(x) = Prevalence of alcohol consumption

RR_F = Relative risk of former drinkers

RR_C (x) = Relative risk function for drinkers

$$k = \frac{\overline{x}^2}{\sigma(\overline{x})^2}$$

$$\theta = \frac{\sigma(\overline{x})^2}{\overline{x}^2}$$

A thousand draws were taken of PAFs to generate uncertainty. The gamma distribution was used to estimate individual level variation within drinking populations [8-9]. Binge drinkers were not taken into account for chronic causes since the pattern of drinking has not been found to be an indicator of most outcomes [10].

For non-chronic conditions, such as injuries, binge drinking was accounted for in the model since patterns of drinking is significant.

$$PAF(x) = \frac{P_A + P_F + P_C + P_{C+B} * RR_{C+B}(x) - 1}{P_A + P_F + P_C + P_{C+B} * RR_{C+B}(x)}$$

$$RR_{C+B}(x) = P_D * P_{D+B} * (RR_{crude}(x) - 1) + 1$$

where:

P_{C+B} = Prevalence of current drinkers who binge

RR_{C+B} = Relative risk of current drinkers who binge

RR_{crude} = Relative risk for a given mean level of consumption

P_D = Proportion of a day that is a binge event

P_{D+B} = Proportion of all days where a binge event occurs

References

1. Food and Agriculture Organization of the United Nations. FAOSTAT Statistics Database.
2. World Health Organization (WHO). WHO Global Health Observatory - Recorded adult per capita alcohol consumption, Total per country. Geneva, Switzerland: World Health Organization (WHO).
3. UN World Tourism Organization (UNWTO). UN World Tourism Organization Compendium of Tourism Statistics 2015 [Electronic]. Madrid, Spain: UN World Tourism Organization (UNWTO), 2016.
4. World Health Organization (WHO). WHO Global Health Observatory – Unrecorded consumption by country. Geneva, Switzerland: World Health Organization (WHO).
5. Rehm, J., et al. "Are lifetime abstainers the best control group in alcohol epidemiology? On the stability and validity of reported lifetime abstention."American journal of epidemiology 168.8 (2008): 866-871.
6. Chikritzhs, Tanya, Kaye Fillmore, and T. I. M. Stockwell. "A healthy dose of scepticism: four good reasons to think again about protective effects of alcohol on coronary heart disease." Drug and alcohol review 28, no. 4 (2009): 441-444.
7. Jackson, Rod, Joanna Broad, Jennie Connor, and Susan Wells. "Alcohol and ischaemic heart disease: probably no free lunch." The Lancet 366, no. 9501 (2005): 1911-1912.
8. Kehoe, Tara, Gerrit Gmel, Kevin D. Shield, Gerhard Gmel, and Jürgen Rehm. "Determining the best population-level alcohol consumption model and its impact on estimates of alcohol-attributable harms." Population health metrics 10, no. 1 (2012): 1.
9. Rehm, Jürgen, Tara Kehoe, Gerrit Gmel, Fred Stinson, Bridget Grant, and Gerhard Gmel. "Statistical modeling of volume of alcohol exposure for epidemiological studies of population health: the US example." Population Health Metrics 8, no. 1 (2010): 1.
10. Rehm, Jürgen, Robin Room, Kathryn Graham, Maristela Monteiro, Gerhard Gmel, and Christopher T. Sempos. "The relationship of average volume of alcohol consumption and patterns of drinking to burden of disease: an overview." Addiction 98, no. 9 (2003): 1209-1228.
11. Roerecke M, Rehm J. Alcohol consumption and the risk for morbidity and mortality of ischemic heart disease - A systemic review and meta-analysis. Toronto, Canada: Centre for Addiction and Mental Health; 2011
12. Bagnardi V, Blangiardo M, La Vecchia C, Corrao G. A meta-analysis of alcohol drinking and cancer risk. Br J Cancer. 2001; 85(11): 1700-5.

13. Corrao G, Bagnardi V, Zambon A, La Vecchia C. A meta-analysis of alcohol consumption and the risk of 15 diseases. Prev Med. 2004; 38(5): 613-9.

14. Samokhvalov AV, Irving HM, Rehm J. Alcohol consumption as a risk factor for pneumonia: a systematic review and meta-analysis. Epidemiol Infect. 2010; 138(12): 1789-95.

15. Samokhvalov AV, Irving H, Mohapatra S, Rehm J. Alcohol consumption, unprovoked seizures, and epilepsy: a systematic review and meta-analysis. Epilepsia. 2010; 51(7): 1177-84.

16. Samokhvalov AV, Irving HM, Rehm J. Alcohol consumption as a risk factor for atrial fibrillation: a systematic review and meta-analysis. Eur J Cardiovasc Prev Rehabil. 2010; 17(6): 706-12.

17. Rehm J, Samokhvalov AV, Neuman MG, Room R, Parry C, Lönnroth K, Patra J, Poznyak V, Popova S. The association between alcohol use, alcohol use disorders and tuberculosis (TB). A systematic review. BMC Public Health. 2009; 450.

18. Lönnroth K, Williams BG, Stadlin S, Jaramillo E, Dye C. Alcohol use as a risk factor for tuberculosis - a systematic review. BMC Public Health. 2008; 289.

19. Roerecke M, Rehm J. Alcohol consumption and the risk for morbidity and mortality of ischemic heart disease - A systemic review and meta-analysis. Toronto, Canada: Centre for Addiction and Mental Health; 2011.

20. Rehm J, Taylor B, Mohapatra S, Irving H, Baliunas D, Patra J, Roerecke M.. Alcohol as a risk factor for liver cirrhosis: a systematic review and meta-analysis. Drug Alcohol Rev. 2010; 29(4): 437-45.

21. Patra J, Taylor B, Irving H, Roerecke M, Baliunas D, Mohapatra S, Rehm J. Alcohol consumption and the risk of morbidity and mortality for different stroke types--a systematic review and meta-analysis. BMC Public Health. 2010; 258.

22. Taylor B, Irving HM, Kanteres F, Room R, Borges G, Cherpitel C, Greenfield T, Rehm J. The more you drink, the harder you fall: a systematic review and meta-analysis of how acute alcohol consumption and injury or collision risk increase together. Drug Alcohol Depend. 2010; 110(1-2): 108-16.

Smoking

Flowchart

Smoking

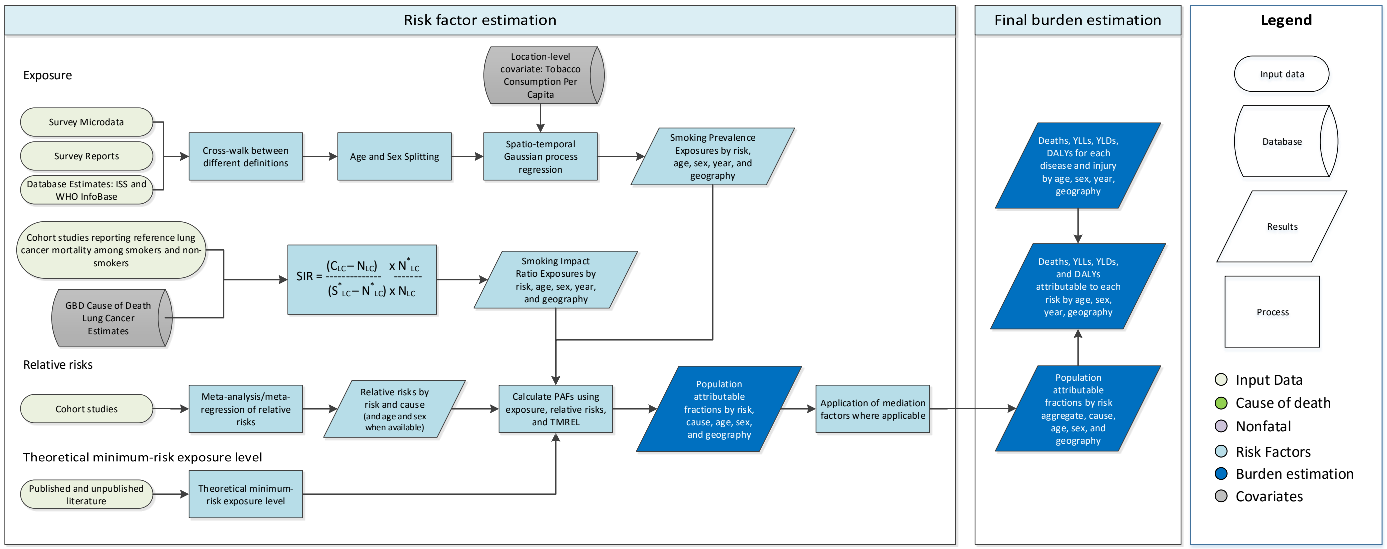

Input Data & Methodological Summary

Exposure

Case definition

We used 5-year lagged smoking prevalence, for modeling burden attributable to smoking for cardiovascular diseases, TB, diabetes, lower respiratory infections, asthma, cataracts, macular degeneration, fractures, rheumatoid arthritis, and peptic ulcer disease. Smoking is a dichotomous exposure defined as current daily use of smoked tobacco.

Input data

Consistent with GBD 2013, we used nationally representative survey data to estimate smoking prevalence. Survey and report data identified in the Global Health Data Exchange (GHDx), the WHO InfoBase, and the International Smoking Statistics (ISS) Database.

Inclusion Criteria

- Nationally representative
- Report current use of any of the following frequency-type combinations:
    - Daily use of smoked tobacco
    - Any use (both daily and occasional) of smoked tobacco
    - Daily use of cigarettes
    - Any use (both daily and occasional) of cigarettes
    - Daily use of any tobacco (both smoked and smokeless)
    - Any use (both daily and occasional) of any tobacco (both smoked and smokeless)
    - Daily use of any tobacco excluding cigarettes
- Report data within the time period of January 1, 1980 – December 31, 2015 for any geography estimated in the GBD framework
- Smoking prevalence reported among individuals ages 10+

Global Health Data Exchange (GHDx)

Sources were identified through a systematic search of the GHDx.

- Search Terms (Keywords): Tobacco Use
- Time Period: January 1, 1980 – December 31, 2015
- Data Type: Survey OR Report
- Search Date: February 16, 2016

Out of 3,912 sources identified in the GHDx, 2,818 sources were included.

WHO InfoBase and International Smoking Statistics (ISS) Database

An effort was made to replace database-derived estimates used in GBD 2013 with original extractions from primary data sources. In GBD 2013, [851] sources were derived from the WHO InfoBase or the ISS Database. In GBD 2015, we replaced [257] sources with extractions from primary data sources and continued to use [594] sources from the WHO InfoBase (n=[281]) and the ISS Databse (n=[313]).

Outliers

Throughout the modeling process, data were assessed for bias and outliers were flagged. A data point was flagged as a candidate outlier if it was not consistent with the majority of other data points in a country with respect to level, age-pattern, sex-pattern, or temporal trend. In data-scarce countries, data points were also compared to data from other countries in a region. Candidate outliers were scrutinized for potential sources of bias and were ultimately excluded if the point or source was deemed to not be representative.

Modeling strategy

Data Extraction

When possible, we extracted individual smoking status for all available frequency-type categories (listed above) from person-level microdata and collapsed these data to produce prevalence estimates in the standard GBD 5-year age-sex groups. If microdata were unavailable we extracted the most granular age-sex groups available from survey reports. Any available measures of uncertainty were extracted, including standard error, confidence or uncertainty intervals, and sample size.

Data Preparation: Crosswalking

Regressions to crosswalk other frequency-type categories to the gold-standard definition of daily use of smoked tobacco were estimated in the form:

$$p_{daily-smoked,k} = \beta_1 p_{i,k} + \epsilon_k$$

where $p_{daily\text{-}smoked,k}$ is the prevalence of daily smoking reported in survey *k*, and $p_{i,k}$ is the prevalence of an alternative frequency-type combination *i* also reported in survey *k*. Consistent with previous GBD smoking crosswalks, the intercept was omitted from the regression. The estimated regression coefficient β_1 was used to crosswalk alternative frequency-type categories to the gold-standard daily smoking definition in sources only providing the alternative category. Predication error at the data-point level was used to propagate uncertainty and was calculated using the following equation:

$$PE_k = \sigma_\epsilon^2 + X_k^2 var(\hat{\beta})$$

Compared to the separate frequency and type crosswalks used in GBD 2013, the combined frequency-type crosswalk used in GBD 2015 represents an improvement because patterns in frequency that may vary by type and patterns in type that may vary by frequency are captured.

Data Preparation: Age and Sex Splitting

Report data provided in age groups wider than the standard GBD 5-year age groups or as both sexes combined were split using the approach used in Ng et al. Briefly, age-sex patterns were identified using sources with data on multiple age-sex groups and these patterns were applied to split aggregated report data. Uncertainty in the age-sex split was propagated by multiplying the standard error of the data (including the predication error of the crosswalk) by the square root of the number of splits performed.

Modeling: Linear Model

After data preparation, the dataset consisted of prevalence estimates of daily smoked tobacco use in standard GBD country-year-age-sex groups. The mean function used in ST-GPR was estimated using the following hierarchical mixed-effects linear regression, run separately by sex:

$$\text{logit}(p_{c,a,t}) = \beta_0 + \beta_1 CPC_{c,t} + \sum_{k=2}^{16} \beta_k I_{A[a]} + \alpha_s + \alpha_r + \alpha_c + \epsilon_{c,a,t}$$

where $CPC_{c,t}$ is the annual tobacco consumption per capita covariate, $I_{A[a]}$ is a dummy variable indicating specific age group A that the prevalence point $p_{c,a,t}$ is capturing, and α_s, α_r, and α_c are super region, region, and country-specific random effects.

Modeling: Spatio-Temporal Gaussian Process Regression (ST-GPR)

The estimated mean function was then propagated through the ST-GPR framework to obtain 1,000 draws of smoking prevalence estimates for each location, year, age, and sex. Parameter selection for the ST-GPR hyper-parameters were selected through out-of-sample cross-validation using the strategy described elsewhere in this appendix.

Theoretical minimum-risk exposure level

The theoretical minimum-risk exposure level is that no one in the population smokes tobacco; that is, the smoking impact ratio is zero and smoking prevalence is zero.

Relative risk

We have made no substantive updates to relative risks for outcomes included in GBD 2013.

High Fasting Plasma Glucose/Diabetes

Flowchart

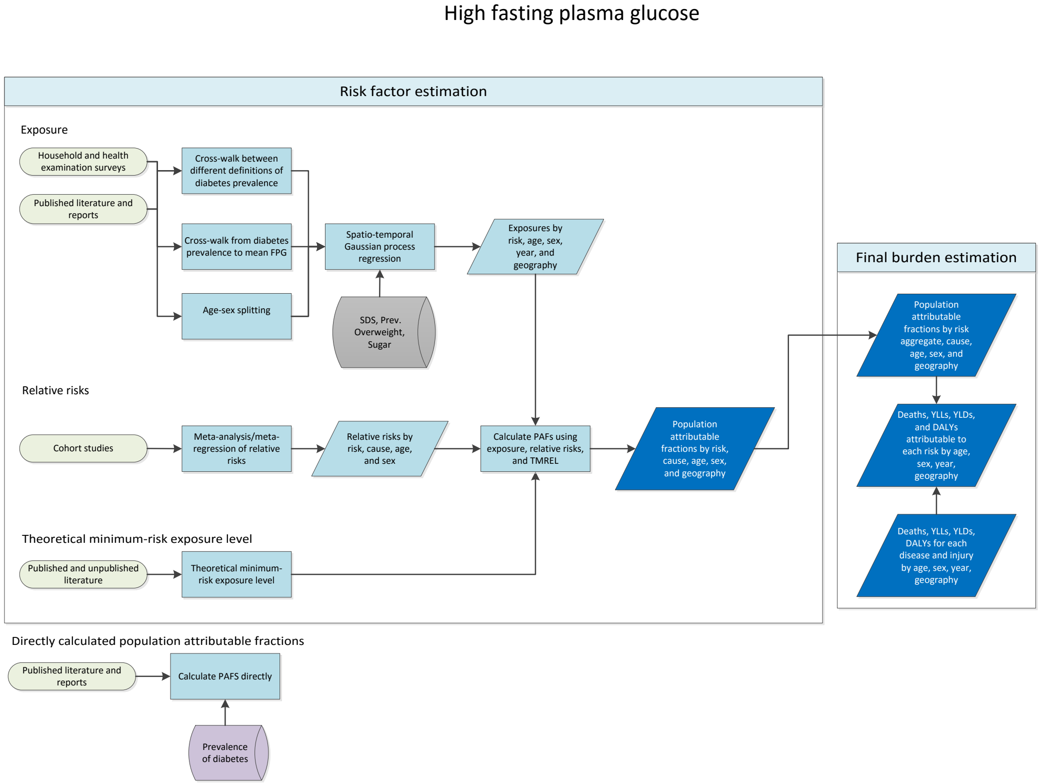

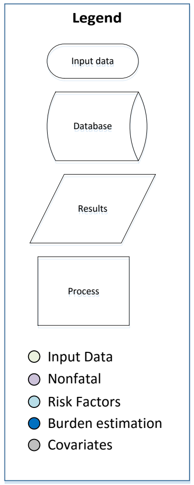

Input Data & Methodological Summary

Exposure

Case Definition

We measure fasting plasma glucose as a continuous exposure in units of mmol/L and define diabetes according to the American Diabetes Association (ADA) and World Health Organization (WHO) diagnostic guidelines as FPG $\geq$ 7.0 mmol/L and/or currently taking diabetes.[1,2]

Input Data

Consistent with GBD 2013, we utilized data on mean fasting plasma glucose from literature and from household survey microdata and reports (e.g. STEPS, NHANES). Please see appendix for a full list of included sources. In GBD 2013, a systematic review of the literature was completed to capture population survey data on mean fasting plasma glucose. For GBD 2015, we updated the systematic review using the same strategy, drawing from the GHDx and Medline via PubMed. In total, we have utilized 717 sources corresponding to 24,926 unique data points.

Global Health Data Exchange Database

We systematically searched the Global Health Data Exchange (GHDx) for multi-country survey programs, national surveys, and longitudinal studies which provide measured individual level data on fasting plasma glucose. The search was completed for systolic blood pressure, fasting plasma glucose, and blood cholesterol simultaneously, as many sources studying the other metabolic risks will often report mean fasting plasma glucose or diabetes prevalence.

Search Terms (Keywords): Blood pressure OR Blood glucose OR Glucose tests OR Cholesterol OR Cholesterol tests OR Hypercholesterolemia
Data Type: Survey OR Report
Search date: 2/6/2016

Literature Review

We systematically searched PubMed for articles published between 15 July 2009 and 31 December 2015 which provided national or subnational estimates of mean fasting plasma glucose. As above, the literature review was completed for systolic blood pressure, fasting plasma glucose, and blood cholesterol simultaneously for the reasons previously stated.

Search terms:

(("hypertension"[Mesh:NoExp] OR "blood pressure"[Mesh:NoExp] OR "Hyperlipidemias"[Mesh:NoExp] OR "Hypercholesterolemia"[Mesh] OR "Cholesterol"[Mesh] OR “diabetes mellitus”[Mesh:NoExp] OR “diabetes mellitus, type 2”[Mesh] OR “glucose”[Mesh] OR “hyperglycemia”[Mesh] OR

“prediabetic state”[Mesh]) AND "Geographic Locations"[Mesh] NOT "United States"[Mesh]) AND ("humans"[Mesh] AND "adult"[MeSH]) AND ("Data Collection"[Mesh] OR "Health Services Research"[Mesh] OR "Population Surveillance"[Mesh] OR "Vital statistics"[Mesh] OR "Population"[Mesh] OR "Epidemiology"[Mesh] OR "surve*"[TiAb]) NOT Comment[ptyp] NOT Case Reports[ptyp] AND ("2009/07/15"[PDAT] : "2015/12/31"[PDAT]) NOT "hospital"[TiAb]

Search date: 1/26/2016

Expert Groups

To capture any remaining sources not identified in the GHDx or in PubMed, we looked to other leaders in the field to ensure our datasets were as comprehensive as possible. These included the IDF Atlas Database and a recent publication on diabetes from the NCD Risk Factor Collaboration. [3,4]

Inclusion Criteria

Studies were included if they were population-based and measured glucose using a blood test (as FPG, HbA1c). We accepted data on diabetes prevalence only if the study performed an objective blood measurement and/or individuals reported self-report of taking anti-diabetic medication. Studies that included self-report of diabetes were excluded. We assumed the data is representative of the location if the geography was not related to the diseases (a mining area) and if it is not an outlier compared to other data in the country or region.

Outliers

Data was utilized in the modeling process unless an assessment of data showed that the data is biased. A data point was considered to be an outlier candidate if the level is not consistent with other (sources) country data, or - if there are no other data points - not consistent with other country in the region. A candidate outlier source was scrutinized and validated and the data point was excluded if the quality of study did not warrant a valid estimate because of selection (specific populations), different definitions, other biases, or if the study did not provide methodological details for evaluation.

Data Extraction

Where possible, individual level data on fasting plasma glucose was extracted from survey microdata and these were collapsed across demographic groupings to produce mean estimates in the standard GBD 5-year age-sex groups. If microdata were unavailable, information from survey reports or from literature were extracted along with any available measure of uncertainty including standard error, uncertainty intervals, and sample size.

Survey reports and literature often only report information on diabetes prevalence in the population studied. If the study was otherwise representative, we extracted data on the prevalence of diabetes and, using all available data with both estimates of mean fasting plasma glucose and prevalence of diabetes, crosswalked this to estimates of mean fasting plasma glucose.

Crosswalk from Prevalence of Diabetes and HbA1c

We used a mixed-effects regression to crosswalk estimates of diabetes prevalence to the mean fasting plasma glucose of a given population. A separate regression was run for a given diagnostic criteria using the form:

$$\log\left(\mathrm{FPG}_{c,a,s,t,k}\right) = \beta_0 + \beta_1 \mathrm{logit}(p_{c,a,s,t,k}) + \beta_2 \mathrm{male} + \sum_{k=10}^{21} \beta_h I_{A[a]} + \alpha_s + \epsilon_{c,a,s,t,k}$$

Where $\mathrm{FPG}_{c,a,s,t,k}$ is the outcome of interest—the mean fasting plasma glucose of a given country-, age-, sex-, time-, from survey k; $p_{c,a,s,t,k}$ is the prevalence of diabetes for a given definition or the mean HbA1c level; $I_{A[a]}$ is a dummy variable indicating a specific age group A; and α_s is a super-region specific random effect.

Age and Sex Splitting

Prior to modeling, data provided in age groups wider than the GBD 5-year age groups were split using the approach outlined in Ng et al.[5] Briefly, age-sex patterns were identified using sources of data with multiple age-sex groups and these patterns were applied to split aggregated report data. Uncertainty in the age-sex split was propagated by multiplying the standard error of the data performed by the square root of the number of splits performed.

Modeling

Exposure estimates were produced from 1980 to 2015 for each national and subnational location, sex, and for each 5-year age group starting from 25+. As in GBD 2013, we used a Spatio-Temporal Gaussian Process Regression (ST-GPR) framework to model the mean fasting plasma glucose at the location-, year-, age-, sex- level. Updates to the ST-GR modeling framework for GBD 2015 are detailed in the appendix.

The FPG mean function was estimated using a mixed-effects linear regression, run separately by sex:

$$\mathrm{logit}\left(\mathrm{FPG}_{c,a,t}\right) = \beta_0 + \beta_1 \mathrm{SDS}_{c,t} + \beta_2 p_{\mathrm{overweight}_{c,a,t}} + \beta_2 \log(\mathrm{sugar}_{c,,t}) + \sum_{k=2}^{16} \beta_k I_{A[a]} + \alpha_s + \alpha_r + \alpha_c + \epsilon_{c,a,t}$$

where $\mathrm{SDS}_{c,t}$ is socio-demographic status (SDS), an index metric that includes a measure of education and income, $p_{\mathrm{overweight}_{c,a,t}}$ is the prevalence of overweight, $\mathrm{sugar}_{c,,t}$ is the diet adjusted mean consumption of sugar in grams per capita per day, $I_{A[a]}$ is a dummy variable for a fixed effect on a given 5-year age group, and α_s α_r α_c are random effects at the super-region, region, and country level, respectively.

The estimates were then propagated through the ST-GPR framework to obtain 1000 draws for each location, year, age, and sex.

Theoretical minimum-risk exposure level

As in GBD 2013, the theoretical minimum risk exposure level for fasting plasma glucose is between 4.9 and 5.3 mmol/l (uniformly distributed) with a standard deviation 0.3mmol/l. This SD is the lowest reported in population data, after correction for the effects of one-time measurement. We used the same TMREL at all ages because FPG does not rise sharply with age in populations with low blood glucose.

Relative risks

We used Dismod-MR 2.1 to pool effect sizes from included studies and generate a dose-response curve for each of the outcomes associated with high fasting plasma glucose. The tool enabled us to incorporate random effects across studies and include data with different age ranges. RRs were used universally for all countries and the meta-regression only helped to pool the three major sources and produce RRs with uncertainty and covariance across ages taking into account the uncertainty of the data points

We have updated the relative risks for tuberculosis as an outcome of diabetes using evidence from recent health record linkage studies from the UK, Australia, and Taiwan, as well as other prospective cohort studies. A full list of studies utilized is available in the GBD Data Input Sources Tool (http://ghdx.healthdata.org/gbd-2015/data-input-sources).

References

1 Association AD. Classification and Diagnosis of Diabetes. *Diabetes Care* 2015; **38**: S8–16.

2 World Health Organization, International Diabetes Federation. Definition and diagnosis of diabetes mellitus and intermediate hyperglycaemia: report of a WHO/IDF consultation. 2006 http://www.who.int/diabetes/publications/diagnosis%5Fdiabetes2006/en/ (accessed July 24, 2016).

3 IDF diabetes atlas - Home. http://www.diabetesatlas.org/ (accessed July 24, 2016).

4 Worldwide trends in diabetes since 1980: a pooled analysis of 751 population-based studies with 4·4 million participants. *The Lancet* 2016; **387**: 1513–30.

5 Ng M, Fleming T, Robinson M, *et al.* Global, regional, and national prevalence of overweight and obesity in children and adults during 1980–2013: a systematic analysis for the Global Burden of Disease Study 2013. *The Lancet* 2014; **384**: 766–81.

6 Singh GM, Danaei G, Farzadfar F, *et al.* The age-specific quantitative effects of metabolic risk factors on cardiovascular diseases and diabetes: a pooled analysis. *PloS One* 2013; **8**: e65174.

Comparative risk assessment input data sources

Alcohol

Citations

Aarhus University, Addiction Switzerland Research Institute, Alcohol Research Group, Public Health Institute, Centre for Addiction and Mental Health (Canada), Centre for Alcohol Policy Research, Turning Point Alcohol and Drug Centre (Australia), Kettil Bruun Society for Social and Epidemiological Research on Alcohol, University of North Dakota. France Gender, Alcohol and Culture: An International Study (GENACIS) 1999

Aarhus University, Addiction Switzerland Research Institute, Alcohol Research Group, Public Health Institute, Centre for Addiction and Mental Health (Canada), Centre for Alcohol Policy Research, Turning Point Alcohol and Drug Centre (Australia), Kettil Bruun Society for Social and Epidemiological Research on Alcohol, University of North Dakota. Mexico Gender, Alcohol and Culture: An International Study (GENACIS) 1998

Aarhus University, Addiction Switzerland Research Institute, Alcohol Research Group, Public Health Institute, Centre for Addiction and Mental Health (Canada), Centre for Alcohol Policy Research, Turning Point Alcohol and Drug Centre (Australia), Kettil Bruun Society for Social and Epidemiological Research on Alcohol, University of North Dakota. Norway Gender, Alcohol and Culture: An International Study (GENACIS) 1999

Aarhus University, Addiction Switzerland Research Institute, Alcohol Research Group, Public Health Institute, Centre for Addiction and Mental Health (Canada), Centre for Alcohol Policy Research, Turning Point Alcohol and Drug Centre (Australia), Kettil Bruun Society for Social and Epidemiological Research on Alcohol, University of North Dakota. Uganda Gender, Alcohol and Culture: An International Study (GENACIS) 2003

Aarhus University, Addiction Switzerland Research Institute, Alcohol Research Group, Public Health Institute, Centre for Addiction and Mental Health (Canada), Centre for Alcohol Policy Research, Turning Point Alcohol and Drug Centre (Australia), Kettil Bruun Society for Social and Epidemiological Research on Alcohol, University of North Dakota. Australia Gender, Alcohol and Culture: An International Study (GENACIS) 1994

Aarhus University, Addiction Switzerland Research Institute, Alcohol Research Group, Public Health Institute, Centre for Addiction and Mental Health (Canada), Centre for Alcohol Policy Research, Turning Point Alcohol and Drug Centre (Australia), Kettil Bruun Society for Social and Epidemiological Research on Alcohol, University of North Dakota. Australia - Victoria Gender, Alcohol and Culture: An International Study (GENACIS) 2007

Aarhus University, Addiction Switzerland Research Institute, Alcohol Research Group, Public Health Institute, Centre for Addiction and Mental Health (Canada), Centre for Alcohol Policy Research, Turning Point Alcohol and Drug Centre (Australia), Kettil Bruun Society for Social and Epidemiological Research on Alcohol, University of North Dakota. Canada Gender, Alcohol and Culture: An International Study (GENACIS) 2004

Aarhus University, Addiction Switzerland Research Institute, Alcohol Research Group, Public Health Institute, Centre for Addiction and Mental Health (Canada), Centre for Alcohol Policy Research, Turning Point Alcohol and Drug Centre (Australia), Kettil Bruun Society for Social and Epidemiological Research on Alcohol, University of North Dakota. Germany Gender, Alcohol and Culture: An International Study (GENACIS) 2000

Aarhus University, Addiction Switzerland Research Institute, Alcohol Research Group, Public Health Institute, Centre for Addiction and Mental Health (Canada), Centre for Alcohol Policy Research, Turning Point Alcohol and Drug Centre (Australia), Kettil Bruun Society for Social and Epidemiological Research on Alcohol, University of North Dakota. Nicaragua Gender, Alcohol and Culture: An International Study (GENACIS) 2005

Aarhus University, Addiction Switzerland Research Institute, Alcohol Research Group, Public Health Institute, Centre for Addiction and Mental Health (Canada), Centre for Alcohol Policy Research, Turning Point Alcohol and Drug Centre (Australia), Kettil Bruun Society for Social and Epidemiological Research on Alcohol, University of North Dakota. New Zealand Gender, Alcohol and Culture: An International Study (GENACIS) 2007

Aarhus University, Addiction Switzerland Research Institute, Alcohol Research Group, Public Health Institute, Centre for Addiction and Mental Health (Canada), Centre for Alcohol Policy Research, Turning Point Alcohol and Drug Centre (Australia), Kettil Bruun Society for Social and Epidemiological Research on Alcohol, University of North Dakota. Spain Gender, Alcohol and Culture: An International Study (GENACIS) 2003

Aarhus University, Addiction Switzerland Research Institute, Alcohol Research Group, Public Health Institute, Centre for Addiction and Mental Health (Canada), Centre for Alcohol Policy Research, Turning Point Alcohol and Drug Centre (Australia), Kettil Bruun Society for Social and Epidemiological Research on Alcohol, University of North Dakota. United States Gender, Alcohol and Culture: An International Study (GENACIS) 2001

Aarhus University, Addiction Switzerland Research Institute, Alcohol Research Group, Public Health Institute, Centre for Addiction and Mental Health (Canada), Centre for Alcohol Policy Research, Turning Point Alcohol and Drug Centre (Australia), Kettil Bruun Society for Social and Epidemiological Research on Alcohol, University of North Dakota. United States Gender, Alcohol and Culture: An International Study (GENACIS) 1995-1996

Aarhus University, Addiction Switzerland Research Institute, Alcohol Research Group, Public Health Institute, Centre for Addiction and Mental Health (Canada), Centre for Alcohol Policy Research, Turning Point Alcohol and Drug Centre (Australia), Kettil Bruun Society for Social and Epidemiological Research on Alcohol, University of North Dakota. United States Gender, Alcohol and Culture: An International Study (GENACIS) 2000

Aarhus University, Addiction Switzerland Research Institute, Alcohol Research Group, Public Health Institute, Centre for Addiction and Mental Health (Canada), Centre for Alcohol Policy Research, Turning Point Alcohol and Drug Centre (Australia), Kettil Bruun Society for Social and Epidemiological Research on Alcohol, University of North Dakota. Iceland Gender, Alcohol and Culture: An International Study (GENACIS) 2001

Aarhus University, Addiction Switzerland Research Institute, Alcohol Research Group, Public Health Institute, Centre for Addiction and Mental Health (Canada), Centre for Alcohol Policy Research, Turning Point Alcohol and Drug Centre (Australia), Kettil Bruun Society for Social and Epidemiological Research on Alcohol, University of North Dakota. India - Karnataka Gender, Alcohol and Culture: An International Study (GENACIS) 2003

Aarhus University, Addiction Switzerland Research Institute, Alcohol Research Group, Public Health Institute, Centre for Addiction and Mental Health (Canada), Centre for Alcohol Policy Research, Turning Point Alcohol and Drug Centre (Australia), Kettil Bruun Society for Social and Epidemiological Research on Alcohol, University of North Dakota. Peru - Lima and Ayacucho Gender, Alcohol and Culture: An International Study (GENACIS) 2005

Aarhus University, Addiction Switzerland Research Institute, Alcohol Research Group, Public Health Institute, Centre for Addiction and Mental Health (Canada), Centre for Alcohol Policy Research, Turning Point Alcohol and Drug Centre (Australia), Kettil Bruun Society for Social and Epidemiological Research on Alcohol, University of North Dakota. Sweden Gender, Alcohol and Culture: An International Study (GENACIS) 2002

Aarhus University, Addiction Switzerland Research Institute, Alcohol Research Group, Public Health Institute, Centre for Addiction and Mental Health (Canada), Centre for Alcohol Policy Research, Turning Point Alcohol and Drug Centre (Australia), Kettil Bruun Society for Social and Epidemiological Research on Alcohol, University of North Dakota. Ireland Gender, Alcohol and Culture: An International Study (GENACIS) 2002

Aarhus University, Addiction Switzerland Research Institute, Alcohol Research Group, Public Health Institute, Centre for Addiction and Mental Health (Canada), Centre for Alcohol Policy Research, Turning Point Alcohol and Drug Centre (Australia), Kettil Bruun Society for Social and Epidemiological Research on Alcohol, University of North Dakota. Nigeria Gender, Alcohol and Culture: An International Study (GENACIS) 2003

Aarhus University, Addiction Switzerland Research Institute, Alcohol Research Group, Public Health Institute, Centre for Addiction and Mental Health (Canada), Centre for Alcohol Policy Research, Turning Point Alcohol and Drug Centre (Australia), Kettil Bruun Society for Social and Epidemiological Research on Alcohol, University of North Dakota. Austria Gender, Alcohol and Culture: An International Study (GENACIS) 1993

Aarhus University, Addiction Switzerland Research Institute, Alcohol Research Group, Public Health Institute, Centre for Addiction and Mental Health (Canada), Centre for Alcohol Policy Research, Turning Point Alcohol and Drug Centre (Australia), Kettil Bruun Society for Social and Epidemiological Research on Alcohol, University of North Dakota. Brazil - Botucatu Gender, Alcohol and Culture: An International Study (GENACIS) 2001-2002

Aarhus University, Addiction Switzerland Research Institute, Alcohol Research Group, Public Health Institute, Centre for Addiction and Mental Health (Canada), Centre for Alcohol Policy Research, Turning Point Alcohol and Drug Centre (Australia), Kettil Bruun Society for Social and Epidemiological Research on Alcohol, University of North Dakota. Israel Gender, Alcohol and Culture: An International Study (GENACIS) 2001

Aarhus University, Addiction Switzerland Research Institute, Alcohol Research Group, Public Health Institute, Centre for Addiction and Mental Health (Canada), Centre for Alcohol Policy Research, Turning Point Alcohol and Drug Centre (Australia), Kettil Bruun Society for Social and Epidemiological Research on Alcohol, University of North Dakota. United Kingdom Gender, Alcohol and Culture: An International Study (GENACIS) 2000

Aarhus University, Addiction Switzerland Research Institute, Alcohol Research Group, Public Health Institute, Centre for Addiction and Mental Health (Canada), Centre for Alcohol Policy Research, Turning Point Alcohol and Drug Centre (Australia), Kettil Bruun Society for Social and Epidemiological Research on Alcohol, University of North Dakota. Costa Rica Gender, Alcohol and Culture: An International Study (GENACIS) 2003

Aarhus University, Addiction Switzerland Research Institute, Alcohol Research Group, Public Health Institute, Centre for Addiction and Mental Health (Canada), Centre for Alcohol Policy Research, Turning Point Alcohol and Drug Centre (Australia), Kettil Bruun Society for Social and Epidemiological Research on Alcohol, University of North Dakota. Italy - Tuscany Gender, Alcohol and Culture: An International Study (GENACIS) 2001-2002

Aarhus University, Addiction Switzerland Research Institute, Alcohol Research Group, Public Health Institute, Centre for Addiction and Mental Health (Canada), Centre for Alcohol Policy Research, Turning Point Alcohol and Drug Centre (Australia), Kettil Bruun Society for Social and Epidemiological Research on Alcohol, University of North Dakota. Kazakhstan Gender, Alcohol and Culture: An International Study (GENACIS) 2002-2003

Aarhus University, Addiction Switzerland Research Institute, Alcohol Research Group, Public Health Institute, Centre for Addiction and Mental Health (Canada), Centre for Alcohol Policy Research, Turning Point Alcohol and Drug Centre (Australia), Kettil Bruun Society for Social and Epidemiological Research on Alcohol, University of North Dakota. Argentina - Buenos Aires Gender, Alcohol and Culture: An International Study (GENACIS) 2003

Aarhus University, Addiction Switzerland Research Institute, Alcohol Research Group, Public Health Institute, Centre for Addiction and Mental Health (Canada), Centre for Alcohol Policy Research, Turning Point Alcohol and Drug Centre (Australia), Kettil Bruun Society for Social and Epidemiological Research on Alcohol, University of North Dakota. Czech Republic Gender, Alcohol and Culture: An International Study (GENACIS) 2002

Aarhus University, Addiction Switzerland Research Institute, Alcohol Research Group, Public Health Institute, Centre for Addiction and Mental Health (Canada), Centre for Alcohol Policy Research, Turning Point Alcohol and Drug Centre (Australia), Kettil Bruun Society for Social and Epidemiological Research on Alcohol, University of North Dakota. Japan Gender, Alcohol and Culture: An International Study (GENACIS) 2001

Aarhus University, Addiction Switzerland Research Institute, Alcohol Research Group, Public Health Institute, Centre for Addiction and Mental Health (Canada), Centre for Alcohol Policy Research, Turning Point Alcohol and Drug Centre (Australia), Kettil Bruun Society for Social and Epidemiological Research on Alcohol, University of North Dakota. Sri Lanka Gender, Alcohol and Culture: An International Study (GENACIS) 2002

Aarhus University, Addiction Switzerland Research Institute, Alcohol Research Group, Public Health Institute, Centre for Addiction and Mental Health (Canada), Centre for Alcohol Policy Research, Turning Point Alcohol and Drug Centre (Australia), Kettil Bruun Society for Social and Epidemiological Research on Alcohol, University of North Dakota. Belize Gender, Alcohol and Culture: An International Study (GENACIS) 2005

Aarhus University, Addiction Switzerland Research Institute, Alcohol Research Group, Public Health Institute, Centre for Addiction and Mental Health (Canada), Centre for Alcohol Policy Research, Turning Point Alcohol and Drug Centre (Australia), Kettil Bruun Society for Social and Epidemiological Research on Alcohol, University of North Dakota. Denmark Gender, Alcohol and Culture: An International Study (GENACIS) 2003

Aarhus University, Addiction Switzerland Research Institute, Alcohol Research Group, Public Health Institute, Centre for Addiction and Mental Health (Canada), Centre for Alcohol Policy Research, Turning Point Alcohol and Drug Centre (Australia), Kettil Bruun Society for Social and Epidemiological Research on Alcohol, University of North Dakota. Hungary Gender, Alcohol and Culture: An International Study (GENACIS) 2001

Aarhus University, Addiction Switzerland Research Institute, Alcohol Research Group, Public Health Institute, Centre for Addiction and Mental Health (Canada), Centre for Alcohol Policy Research, Turning Point Alcohol and Drug Centre (Australia), Kettil Bruun Society for Social and Epidemiological Research on Alcohol, University of North Dakota. Netherlands - Limburg Gender, Alcohol and Culture: An International Study (GENACIS) 1999

Aarhus University, Addiction Switzerland Research Institute, Alcohol Research Group, Public Health Institute, Centre for Addiction and Mental Health (Canada), Centre for Alcohol Policy Research, Turning Point Alcohol and Drug Centre (Australia), Kettil Bruun Society for Social and Epidemiological Research on Alcohol, University of North Dakota. Uruguay Gender, Alcohol and Culture: An International Study (GENACIS) 2004

AMATEM (Turkey), Plaza Ltd. Research, World Health Organization (WHO). Turkey WHO Multi-country Survey Study on Health and Health System Responsiveness 2000-2001. Geneva, Switzerland: World Health Organization (WHO)

Australian Agency for International Development (AusAID), Fiji School of Medicine, Menzies Center for Population Health Research, University of Tasmania (Australia), Ministry of Health (Fiji), World Health Organization (WHO). Fiji STEPS Noncommunicable Disease Risk Factors Survey 2002

Australian Institute of Health and Welfare, National Heart Foundation (Australia). Australia Risk Factor Prevalence Survey 1989. Acton, Australia: Australian Data Archive

Börsch-Supan, A. (2013). Survey of Health, Ageing and Retirement in Europe (SHARE) Wave 2. Release version: 2.6.0. SHARE-ERIC. Data set. DOI: 10.6103/SHARE.w2.260

Börsch-Supan, A. (2013). Survey of Health, Ageing and Retirement in Europe (SHARE) Wave 4. Release version: 1.1.1. SHARE-ERIC. Data set. DOI: 10.6103/SHARE.w4.111

Börsch-Supan, A. (2015). Survey of Health, Ageing and Retirement in Europe (SHARE) Wave 5. Release version: 1.0.0. SHARE-ERIC. Data set. DOI: 10.6103/SHARE.w5.100

Carolina Population Center, University of North Carolina at Chapel Hill, Chinese Center for Disease Control and Prevention (CCDC). China Health and Nutrition Survey 1989-2011. Chapel Hill, United States: Carolina Population Center, University of North Carolina at Chapel Hill

Carolina Population Center, University of North Carolina at Chapel Hill, Chinese Center for Disease Control and Prevention (CCDC). China Health and Nutrition Survey 1991

Carolina Population Center, University of North Carolina at Chapel Hill, Institute of Sociology, Russian Academy of Sciences, National Research University Higher School of Economics (Russia), ZAO Demoscope. Russia Longitudinal Monitoring Survey of HSE, Round I 1992

Case, A. 2003. Agincourt Integrated Family Survey 2002. [dataset] Version 1. Cape Town: DataFirst [distributor]

CBG Health Research Ltd., Ministry of Health (New Zealand), University of Otago (New Zealand). New Zealand Adult Nutrition Survey 2008-2009

Center for Addiction Medicine, National Institute of Mental Health and Neurosciences (India), Indian Council of Medical Research (ICMR). India - Andaman and Nicobar Islands Alcohol Consumption Survey 2007

Center for Addiction Medicine, National Institute of Mental Health and Neurosciences (India), World Health Organization (WHO). India - Karnataka Unrecorded Alcohol Consumption Survey 2001-2002

Center for Addiction Medicine, National Institute of Mental Health and Neurosciences (India). India Alcohol Misuse Epidemiological Survey 2011-2012

Center for Scientific and Technological Information, Oswaldo Cruz Foundation and World Health Organization (WHO). Brazil World Health Survey 2003. Geneva, Switzerland: World Health Organization (WHO), 2005

Centers for Disease Control and Prevention (CDC), World Health Organization (WHO). Kenya Global School-Based Student Health Survey 2003

Centers for Disease Control and Prevention (CDC). United States Behavioral Risk Factor Surveillance System 1984. Atlanta, Georgia: CDC, US Department of Health and Human Services

Centers for Disease Control and Prevention (CDC). United States Behavioral Risk Factor Surveillance System 1985. Atlanta, Georgia: CDC, US Department of Health and Human Services

Centers for Disease Control and Prevention (CDC). United States Behavioral Risk Factor Surveillance System 1986. Atlanta, Georgia: CDC, US Department of Health and Human Services

Centers for Disease Control and Prevention (CDC). United States Behavioral Risk Factor Surveillance System 1987. Atlanta, Georgia: CDC, US Department of Health and Human Services

Centers for Disease Control and Prevention (CDC). United States Behavioral Risk Factor Surveillance System 1989. Atlanta, Georgia: CDC, US Department of Health and Human Services

Centers for Disease Control and Prevention (CDC). United States Behavioral Risk Factor Surveillance System 1990. Atlanta, Georgia: CDC, US Department of Health and Human Services

Centers for Disease Control and Prevention (CDC). United States Behavioral Risk Factor Surveillance System 1991. Atlanta, Georgia: CDC, US Department of Health and Human Services

Centers for Disease Control and Prevention (CDC). United States Behavioral Risk Factor Surveillance System 1992. Atlanta, Georgia: CDC, US Department of Health and Human Services

Centers for Disease Control and Prevention (CDC). United States Behavioral Risk Factor Surveillance System 1993. Atlanta, Georgia: CDC, US Department of Health and Human Services

Centers for Disease Control and Prevention (CDC). United States Behavioral Risk Factor Surveillance System 1994. Atlanta, Georgia: CDC, US Department of Health and Human Services

Centers for Disease Control and Prevention (CDC). United States Behavioral Risk Factor Surveillance System 1995. Atlanta, Georgia: CDC, US Department of Health and Human Services

Centers for Disease Control and Prevention (CDC). United States Behavioral Risk Factor Surveillance System 1996. Atlanta, Georgia: CDC, US Department of Health and Human Services

Centers for Disease Control and Prevention (CDC). United States Behavioral Risk Factor Surveillance System 1997. Atlanta, Georgia: CDC, US Department of Health and Human Services

Centers for Disease Control and Prevention (CDC). United States Behavioral Risk Factor Surveillance System 1998. Atlanta, Georgia: CDC, US Department of Health and Human Services

Centers for Disease Control and Prevention (CDC). United States Behavioral Risk Factor Surveillance System 1999. Atlanta, Georgia: CDC, US Department of Health and Human Services

Centers for Disease Control and Prevention (CDC). United States Behavioral Risk Factor Surveillance System 2000. Atlanta, Georgia: CDC, US Department of Health and Human Services

Centers for Disease Control and Prevention (CDC). United States Behavioral Risk Factor Surveillance System 2001. Atlanta, Georgia: CDC, US Department of Health and Human Services

Centers for Disease Control and Prevention (CDC). United States Behavioral Risk Factor Surveillance System 2002. Atlanta, Georgia: CDC, US Department of Health and Human Services

Centers for Disease Control and Prevention (CDC). United States Behavioral Risk Factor Surveillance System 2003. Atlanta, Georgia: CDC, US Department of Health and Human Services

Centers for Disease Control and Prevention (CDC). United States Behavioral Risk Factor Surveillance System 2004. Atlanta, Georgia: CDC, US Department of Health and Human Services

Centers for Disease Control and Prevention (CDC). United States Behavioral Risk Factor Surveillance System 2005. Atlanta, Georgia: CDC, US Department of Health and Human Services

Centers for Disease Control and Prevention (CDC). United States Behavioral Risk Factor Surveillance System 2006. Atlanta, Georgia: CDC, US Department of Health and Human Services

Centers for Disease Control and Prevention (CDC). United States Behavioral Risk Factor Surveillance System 2007. Atlanta, Georgia: CDC, US Department of Health and Human Services

Centers for Disease Control and Prevention (CDC). United States Behavioral Risk Factor Surveillance System 2008. Atlanta, Georgia: CDC, US Department of Health and Human Services

Centers for Disease Control and Prevention (CDC). United States Behavioral Risk Factor Surveillance System 2009. Atlanta, Georgia: CDC, US Department of Health and Human Services

Centers for Disease Control and Prevention (CDC). United States Behavioral Risk Factor Surveillance System 2010. Atlanta, United States: Centers for Disease Control and Prevention (CDC)

Centers for Disease Control and Prevention (CDC). United States Behavioral Risk Factor Surveillance System 2011. Atlanta, Georgia: CDC, US Department of Health and Human Services

Centers for Disease Control and Prevention (CDC). United States Behavioral Risk Factor Surveillance System 2012. Atlanta, Georgia: CDC, US Department of Health and Human Services, 2013

Centers for Disease Control and Prevention (CDC). United States Behavioral Risk Factor Surveillance System 2013. Atlanta, United States: Centers for Disease Control and Prevention (CDC), 2014

Centers for Disease Control and Prevention (CDC). United States Behavioral Risk Factor Surveillance System; 1988. Atlanta, Georgia: CDC, US Department of Health and Human Services

Centre for Physical Activity and Health, University of Sydney (Australia), Department of Health and Social Affairs (Micronesia), Fiji School of Medicine, Micronesia Human Resources Development Center, Pohnpei State Department of Health Services, World Health Organization (WHO). Micronesia - Pohnpei STEPS Noncommunicable Disease Risk Factors Survey 2002

China Center for Economic Research, Peking University. China Health and Retirement Longitudinal Study 2008. Beijing, China: China Center for Economic Research, Peking University

China Center for Economic Research, Peking University. China Health and Retirement Longitudinal Study 2011-2012. Beijing, China: China Center for Economic Research, Peking University

Chinese Center for Disease Control and Prevention (CCDC). China Chronic Disease and Risk Factor Surveillance 2004

Chinese Center for Disease Control and Prevention (CCDC). China Chronic Disease and Risk Factor Surveillance 2007

Chinese Center for Disease Control and Prevention (CCDC). China Chronic Disease and Risk Factor Surveillance 2010

Chinese Center for Disease Control and Prevention (CCDC). China Chronic Disease and Risk Factor Surveillance 2013

Currie, C. and Corbett, J., Scottish Schools Adolescent Lifestyle and Substance Use Survey, 2002 [computer file]. Colchester, Essex: UK Data Archive [distributor], June 2005. SN: 5195, http://dx.doi.org/10.5255/UKDA-SN-5195-1

Currie, C. and Corbett, J., Scottish Schools Adolescent Lifestyle and Substance Use Survey, 2004 [computer file]. Colchester, Essex: UK Data Archive [distributor], September 2005. SN: 5239, http://dx.doi.org/10.5255/UKDA-SN-5239-1

Department of Epidemiology and Public Health, University College London, National Centre for Social Research (NatCen). United Kingdom Health Survey for England 2012-2013 - HSCIC

Department of Epidemiology and Public Health, University College London, National Centre for Social Research (NatCen). United Kingdom Health Survey for England 2013-2014 - HSCIC

Department of Health (South Africa), Human Sciences Research Council, World Health Organization (WHO). South Africa WHO Study on Global AGEing and Adult Health 2007-2008. Geneva, Switzerland: World Health Organization (WHO)

Esser MB, Gururaj G, Rao GN, Jernigan DH, Murthy P, Jayarajan D, Lakshmanan S, Benegal V, Collaborators Group on Epidemiological Study of Patterns and Consequences of Alcohol Misuse in India. Harms to Adults from Others' Heavy Drinking in Five Indian States. Alcohol Alcohol. 2016; 51(2): 177–85

European Commission (2012): Eurobarometer 52.1 (Nov-Dec 1999). INRA, Brussels; NSD, Bergen. GESIS Data Archive, Cologne. ZA3205 Data file Version 2.0.0, doi:10.4232/1.11377

European Commission (2012): Eurobarometer 52.1 (Nov-Dec 1999). INRA, Brussels; NSD, Bergen. GESIS Data Archive, Cologne. ZA3205 Data file Version 2.0.0, doi:10.4232/1.11377

European Commission (2012): Eurobarometer 57.2OVR (Apr-Jun 2002). European Opinion Research Group (EORG), Brussels. GESIS Data Archive, Cologne. ZA3641 Data file Version 1.0.1, doi:10.4232/1.10951

European Commission (2012): Eurobarometer 59.0 (Jan-Feb 2003). European Opinion Research Group (EORG), Brussels. GESIS Data Archive, Cologne. ZA3903 Data file Version 1.0.1, doi:10.4232/1.11352

European Commission (2012): Eurobarometer 59.0 (Jan-Feb 2003). European Opinion Research Group (EORG), Brussels. GESIS Data Archive, Cologne. ZA3903 Data file Version 1.0.1, doi:10.4232/1.11352

European Commission (2012): Eurobarometer 59.0 (Jan-Feb 2003). European Opinion Research Group (EORG), Brussels. GESIS Data Archive, Cologne. ZA3903 Data file Version 1.0.1, doi:10.4232/1.11352

European Commission (2012): Eurobarometer 59.0 (Jan-Feb 2003). European Opinion Research Group (EORG), Brussels. GESIS Data Archive, Cologne. ZA3903 Data file Version 1.0.1, doi:10.4232/1.11352

European Commission (2012): Eurobarometer 59.0 (Jan-Feb 2003). European Opinion Research Group (EORG), Brussels. GESIS Data Archive, Cologne. ZA3903 Data file Version 1.0.1, doi:10.4232/1.11352

European Commission (2012): Eurobarometer 59.0 (Jan-Feb 2003). European Opinion Research Group (EORG), Brussels. GESIS Data Archive, Cologne. ZA3903 Data file Version 1.0.1, doi:10.4232/1.11352

European Commission (2012): Eurobarometer 59.0 (Jan-Feb 2003). European Opinion Research Group (EORG), Brussels. GESIS Data Archive, Cologne. ZA3903 Data file Version 1.0.1, doi:10.4232/1.11352

European Commission (2012): Eurobarometer 59.0 (Jan-Feb 2003). European Opinion Research Group (EORG), Brussels. GESIS Data Archive, Cologne. ZA3903 Data file Version 1.0.1, doi:10.4232/1.11352

European Commission (2012): Eurobarometer 59.0 (Jan-Feb 2003). European Opinion Research Group (EORG), Brussels. GESIS Data Archive, Cologne. ZA3903 Data file Version 1.0.1, doi:10.4232/1.11352

European Commission (2012): Eurobarometer 59.0 (Jan-Feb 2003). European Opinion Research Group (EORG), Brussels. GESIS Data Archive, Cologne. ZA3903 Data file Version 1.0.1, doi:10.4232/1.11352

European Commission (2012): Eurobarometer 59.0 (Jan-Feb 2003). European Opinion Research Group (EORG), Brussels. GESIS Data Archive, Cologne. ZA3903 Data file Version 1.0.1, doi:10.4232/1.11352

European Commission (2012): Eurobarometer 59.0 (Jan-Feb 2003). European Opinion Research Group (EORG), Brussels. GESIS Data Archive, Cologne. ZA3903 Data file Version 1.0.1, doi:10.4232/1.11352

European Commission (2012): Eurobarometer 59.0 (Jan-Feb 2003). European Opinion Research Group (EORG), Brussels. GESIS Data Archive, Cologne. ZA3903 Data file Version 1.0.1, doi:10.4232/1.11352

European Commission (2012): Eurobarometer 59.0 (Jan-Feb 2003). European Opinion Research Group (EORG), Brussels. GESIS Data Archive, Cologne. ZA3903 Data file Version 1.0.1, doi:10.4232/1.11352

European Commission (2012): Eurobarometer 66.2 (Oct-Nov 2006). TNS OPINION & SOCIAL, Brussels [Producer]. GESIS Data Archive, Cologne. ZA4527 Data file Version 1.0.1, doi:10.4232/1.10981

European Commission (2012): Eurobarometer 66.2 (Oct-Nov 2006). TNS OPINION & SOCIAL, Brussels [Producer]. GESIS Data Archive, Cologne. ZA4527 Data file Version 1.0.1, doi:10.4232/1.10981

European Commission (2012): Eurobarometer 66.2 (Oct-Nov 2006). TNS OPINION & SOCIAL, Brussels [Producer]. GESIS Data Archive, Cologne. ZA4527 Data file Version 1.0.1, doi:10.4232/1.10981

European Commission (2012): Eurobarometer 66.2 (Oct-Nov 2006). TNS OPINION & SOCIAL, Brussels [Producer]. GESIS Data Archive, Cologne. ZA4527 Data file Version 1.0.1, doi:10.4232/1.10981

European Commission (2012): Eurobarometer 66.2 (Oct-Nov 2006). TNS OPINION & SOCIAL, Brussels [Producer]. GESIS Data Archive, Cologne. ZA4527 Data file Version 1.0.1, doi:10.4232/1.10981

European Commission (2012): Eurobarometer 66.2 (Oct-Nov 2006). TNS OPINION & SOCIAL, Brussels [Producer]. GESIS Data Archive, Cologne. ZA4527 Data file Version 1.0.1, doi:10.4232/1.10981

European Commission (2012): Eurobarometer 66.2 (Oct-Nov 2006). TNS OPINION & SOCIAL, Brussels [Producer]. GESIS Data Archive, Cologne. ZA4527 Data file Version 1.0.1, doi:10.4232/1.10981

European Commission (2012): Eurobarometer 66.2 (Oct-Nov 2006). TNS OPINION & SOCIAL, Brussels [Producer]. GESIS Data Archive, Cologne. ZA4527 Data file Version 1.0.1, doi:10.4232/1.10981

European Commission (2012): Eurobarometer 66.2 (Oct-Nov 2006). TNS OPINION & SOCIAL, Brussels [Producer]. GESIS Data Archive, Cologne. ZA4527 Data file Version 1.0.1, doi:10.4232/1.10981

European Commission (2012): Eurobarometer 66.2 (Oct-Nov 2006). TNS OPINION & SOCIAL, Brussels [Producer]. GESIS Data Archive, Cologne. ZA4527 Data file Version 1.0.1, doi:10.4232/1.10981

European Commission (2012): Eurobarometer 66.2 (Oct-Nov 2006). TNS OPINION & SOCIAL, Brussels [Producer]. GESIS Data Archive, Cologne. ZA4527 Data file Version 1.0.1, doi:10.4232/1.10981

European Commission (2012): Eurobarometer 66.2 (Oct-Nov 2006). TNS OPINION & SOCIAL, Brussels [Producer]. GESIS Data Archive, Cologne. ZA4527 Data file Version 1.0.1, doi:10.4232/1.10981

European Commission (2012): Eurobarometer 66.2 (Oct-Nov 2006). TNS OPINION & SOCIAL, Brussels [Producer]. GESIS Data Archive, Cologne. ZA4527 Data file Version 1.0.1, doi:10.4232/1.10981

European Commission (2012): Eurobarometer 66.2 (Oct-Nov 2006). TNS OPINION & SOCIAL, Brussels [Producer]. GESIS Data Archive, Cologne. ZA4527 Data file Version 1.0.1, doi:10.4232/1.10981

European Commission (2012): Eurobarometer 66.2 (Oct-Nov 2006). TNS OPINION & SOCIAL, Brussels [Producer]. GESIS Data Archive, Cologne. ZA4527 Data file Version 1.0.1, doi:10.4232/1.10981

European Commission (2012): Eurobarometer 66.2 (Oct-Nov 2006). TNS OPINION & SOCIAL, Brussels [Producer]. GESIS Data Archive, Cologne. ZA4527 Data file Version 1.0.1, doi:10.4232/1.10981

European Commission (2012): Eurobarometer 66.2 (Oct-Nov 2006). TNS OPINION & SOCIAL, Brussels [Producer]. GESIS Data Archive, Cologne. ZA4527 Data file Version 1.0.1, doi:10.4232/1.10981

European Commission (2012): Eurobarometer 66.2 (Oct-Nov 2006). TNS OPINION & SOCIAL, Brussels [Producer]. GESIS Data Archive, Cologne. ZA4527 Data file Version 1.0.1, doi:10.4232/1.10981

European Commission (2012): Eurobarometer 66.2 (Oct-Nov 2006). TNS OPINION & SOCIAL, Brussels [Producer]. GESIS Data Archive, Cologne. ZA4527 Data file Version 1.0.1, doi:10.4232/1.10981

European Commission (2012): Eurobarometer 66.2 (Oct-Nov 2006). TNS OPINION & SOCIAL, Brussels [Producer]. GESIS Data Archive, Cologne. ZA4527 Data file Version 1.0.1, doi:10.4232/1.10981

European Commission (2012): Eurobarometer 66.2 (Oct-Nov 2006). TNS OPINION & SOCIAL, Brussels [Producer]. GESIS Data Archive, Cologne. ZA4527 Data file Version 1.0.1, doi:10.4232/1.10981

European Commission (2012): Eurobarometer 66.2 (Oct-Nov 2006). TNS OPINION & SOCIAL, Brussels [Producer]. GESIS Data Archive, Cologne. ZA4527 Data file Version 1.0.1, doi:10.4232/1.10981

European Commission (2012): Eurobarometer 66.2 (Oct-Nov 2006). TNS OPINION & SOCIAL, Brussels [Producer]. GESIS Data Archive, Cologne. ZA4527 Data file Version 1.0.1, doi:10.4232/1.10981

European Commission (2012): Eurobarometer 66.2 (Oct-Nov 2006). TNS OPINION & SOCIAL, Brussels [Producer]. GESIS Data Archive, Cologne. ZA4527 Data file Version 1.0.1, doi:10.4232/1.10981

European Commission (2012): Eurobarometer 66.2 (Oct-Nov 2006). TNS OPINION & SOCIAL, Brussels [Producer]. GESIS Data Archive, Cologne. ZA4527 Data file Version 1.0.1, doi:10.4232/1.10981

European Commission (2012): Eurobarometer 66.2 (Oct-Nov 2006). TNS OPINION & SOCIAL, Brussels [Producer]. GESIS Data Archive, Cologne. ZA4527 Data file Version 1.0.1, doi:10.4232/1.10981

European Commission (2012): Eurobarometer 66.2 (Oct-Nov 2006). TNS OPINION & SOCIAL, Brussels [Producer]. GESIS Data Archive, Cologne. ZA4527 Data file Version 1.0.1, doi:10.4232/1.10981

European Commission (2012): Eurobarometer 66.2 (Oct-Nov 2006). TNS OPINION & SOCIAL, Brussels [Producer]. GESIS Data Archive, Cologne. ZA4527 Data file Version 1.0.1, doi:10.4232/1.10981

European Commission (2012): Eurobarometer 72.3 (Oct 2009). TNS OPINION & SOCIAL, Brussels [Producer]. GESIS Data Archive, Cologne. ZA4977 Data file Version 2.0.0, doi:10.4232/1.11140

European Commission (2012): Eurobarometer 72.3 (Oct 2009). TNS OPINION & SOCIAL, Brussels [Producer]. GESIS Data Archive, Cologne. ZA4977 Data file Version 2.0.0, doi:10.4232/1.11140

European Commission (2012): Eurobarometer 72.3 (Oct 2009). TNS OPINION & SOCIAL, Brussels [Producer]. GESIS Data Archive, Cologne. ZA4977 Data file Version 2.0.0, doi:10.4232/1.11140

European Commission (2012): Eurobarometer 72.3 (Oct 2009). TNS OPINION & SOCIAL, Brussels [Producer]. GESIS Data Archive, Cologne. ZA4977 Data file Version 2.0.0, doi:10.4232/1.11140

European Commission (2012): Eurobarometer 72.3 (Oct 2009). TNS OPINION & SOCIAL, Brussels [Producer]. GESIS Data Archive, Cologne. ZA4977 Data file Version 2.0.0, doi:10.4232/1.11140

European Commission (2012): Eurobarometer 72.3 (Oct 2009). TNS OPINION & SOCIAL, Brussels [Producer]. GESIS Data Archive, Cologne. ZA4977 Data file Version 2.0.0, doi:10.4232/1.11140

European Commission (2012): Eurobarometer 72.3 (Oct 2009). TNS OPINION & SOCIAL, Brussels [Producer]. GESIS Data Archive, Cologne. ZA4977 Data file Version 2.0.0, doi:10.4232/1.11140

European Commission (2012): Eurobarometer 72.3 (Oct 2009). TNS OPINION & SOCIAL, Brussels [Producer]. GESIS Data Archive, Cologne. ZA4977 Data file Version 2.0.0, doi:10.4232/1.11140

European Commission (2012): Eurobarometer 72.3 (Oct 2009). TNS OPINION & SOCIAL, Brussels [Producer]. GESIS Data Archive, Cologne. ZA4977 Data file Version 2.0.0, doi:10.4232/1.11140

European Commission (2012): Eurobarometer 72.3 (Oct 2009). TNS OPINION & SOCIAL, Brussels [Producer]. GESIS Data Archive, Cologne. ZA4977 Data file Version 2.0.0, doi:10.4232/1.11140

European Commission (2012): Eurobarometer 72.3 (Oct 2009). TNS OPINION & SOCIAL, Brussels [Producer]. GESIS Data Archive, Cologne. ZA4977 Data file Version 2.0.0, doi:10.4232/1.11140

European Commission (2012): Eurobarometer 72.3 (Oct 2009). TNS OPINION & SOCIAL, Brussels [Producer]. GESIS Data Archive, Cologne. ZA4977 Data file Version 2.0.0, doi:10.4232/1.11140

European Commission (2012): Eurobarometer 72.3 (Oct 2009). TNS OPINION & SOCIAL, Brussels [Producer]. GESIS Data Archive, Cologne. ZA4977 Data file Version 2.0.0, doi:10.4232/1.11140

European Commission (2012): Eurobarometer 72.3 (Oct 2009). TNS OPINION & SOCIAL, Brussels [Producer]. GESIS Data Archive, Cologne. ZA4977 Data file Version 2.0.0, doi:10.4232/1.11140

European Commission (2012): Eurobarometer 72.3 (Oct 2009). TNS OPINION & SOCIAL, Brussels [Producer]. GESIS Data Archive, Cologne. ZA4977 Data file Version 2.0.0, doi:10.4232/1.11140

European Commission (2012): Eurobarometer 72.3 (Oct 2009). TNS OPINION & SOCIAL, Brussels [Producer]. GESIS Data Archive, Cologne. ZA4977 Data file Version 2.0.0, doi:10.4232/1.11140

European Commission (2012): Eurobarometer 72.3 (Oct 2009). TNS OPINION & SOCIAL, Brussels [Producer]. GESIS Data Archive, Cologne. ZA4977 Data file Version 2.0.0, doi:10.4232/1.11140

European Commission (2012): Eurobarometer 72.3 (Oct 2009). TNS OPINION & SOCIAL, Brussels [Producer]. GESIS Data Archive, Cologne. ZA4977 Data file Version 2.0.0, doi:10.4232/1.11140

European Commission (2012): Eurobarometer 72.3 (Oct 2009). TNS OPINION & SOCIAL, Brussels [Producer]. GESIS Data Archive, Cologne. ZA4977 Data file Version 2.0.0, doi:10.4232/1.11140

European Commission (2012): Eurobarometer 72.3 (Oct 2009). TNS OPINION & SOCIAL, Brussels [Producer]. GESIS Data Archive, Cologne. ZA4977 Data file Version 2.0.0, doi:10.4232/1.11140

European Commission (2012): Eurobarometer 72.3 (Oct 2009). TNS OPINION & SOCIAL, Brussels [Producer]. GESIS Data Archive, Cologne. ZA4977 Data file Version 2.0.0, doi:10.4232/1.11140

European Commission (2012): Eurobarometer 72.3 (Oct 2009). TNS OPINION & SOCIAL, Brussels [Producer]. GESIS Data Archive, Cologne. ZA4977 Data file Version 2.0.0, doi:10.4232/1.11140

European Commission (2012): Eurobarometer 72.3 (Oct 2009). TNS OPINION & SOCIAL, Brussels [Producer]. GESIS Data Archive, Cologne. ZA4977 Data file Version 2.0.0, doi:10.4232/1.11140

European Commission (2012): Eurobarometer 72.3 (Oct 2009). TNS OPINION & SOCIAL, Brussels [Producer]. GESIS Data Archive, Cologne. ZA4977 Data file Version 2.0.0, doi:10.4232/1.11140

European Commission (2012): Eurobarometer 72.3 (Oct 2009). TNS OPINION & SOCIAL, Brussels [Producer]. GESIS Data Archive, Cologne. ZA4977 Data file Version 2.0.0, doi:10.4232/1.11140

European Commission (2012): Eurobarometer 72.3 (Oct 2009). TNS OPINION & SOCIAL, Brussels [Producer]. GESIS Data Archive, Cologne. ZA4977 Data file Version 2.0.0, doi:10.4232/1.11140

European Commission (2012): Eurobarometer 72.3 (Oct 2009). TNS OPINION & SOCIAL, Brussels [Producer]. GESIS Data Archive, Cologne. ZA4977 Data file Version 2.0.0, doi:10.4232/1.11140

Field, J. et al. , National Survey of Sexual Attitudes and Lifestyles, 1990 [computer file]. Colchester, Essex: UK Data Archive [distributor], August 1995. SN: 3434, http://dx.doi.org/10.5255/UKDA-SN-3434-1

Food and Agriculture Organization of the United Nations (FAO). FAOSTAT Food Balance Sheets, October 2014. Rome, Italy: Food and Agriculture Organization of the United Nations (FAO)

Ghana Health Service, Ministry of Health (Ghana), University of Ghana, World Health Organization (WHO). Ghana WHO Study on Global AGEing and Adult Health 2007-2008

Gilbert, G.N. et al. , General Household Survey (Surrey Files), 1980 [computer file]. Office of Population Censuses and Surveys. Social Survey Division, [original data producer(s)]. Colchester, Essex: UK Data Archive [distributor], 1984. SN: 1897, http://dx.doi.org/10.5255/UKDA-SN-1897-1

Harvard School of Public Health, International Institute for Population Sciences (India), RAND Corporation. India Longitudinal Aging Study Pilot 2010

Health Care International, World Health Organization (WHO). Egypt WHO Multi-country Survey Study on Health and Health System Responsiveness 2000-2001. Geneva, Switzerland: World Health Organization (WHO)

Indian Council of Medical Research (ICMR), National Institute of Medical Statistics (India). India Noncommunicable Disease Risk Factors Survey 2007-2008

Indian Council of Medical Research (ICMR), World Health Organization (WHO). India STEPS Noncommunicable Disease Risk Factors Survey 2003-2005

Institute for Polling and Marketing (Georgia), World Health Organization (WHO). Georgia WHO Multi-country Survey Study on Health and Health System Responsiveness 2000-2001

Institute of Social Medicine and Health Policy, Shandong University, Shandong University School of Medicine, World Health Organization (WHO). China WHO Multi-country Survey Study on Health and Health System Responsiveness 2000-2001

International Diabetes Institute (IDI). Australia Diabetes, Obesity and Lifestyle Study 1999-2000. Melbourne, Australia: International Diabetes Institute (IDI)

International Institute for Population Sciences (India), Macro International, Inc. India Demographic and Health Survey 2005-2006. Calverton, United States: Macro International, Inc

International Institute for Population Sciences (India), World Health Organization (WHO). India WHO Study on Global Ageing and Adult Health 2007. Geneva, Switzerland: World Health Organization (WHO), 2007

International Institute for Population Sciences (India), World Health Organization (WHO). India World Health Survey 2003. Geneva, Switzerland: World Health Organization (WHO), 2005

ISSP Research Group (2009): International Social Survey Programme: Health and Health Care - ISSP 2011. GESIS Data Archive, Cologne. ZA5800 Data file version 3.0.0, doi:10.4232/1.12252

Joint Health Surveys Unit of Social and Community Planning Research and University College London, Health Survey for England, 1995 [computer file]. 3rd ed. Colchester, Essex: UK Data Archive [distributor], 26 March 2001. SN: 3796

Joint Health Surveys Unit of Social and Community Planning Research and University College London, Health Survey for England, 1996 [computer file]. 3rd ed. Colchester, Essex: UK Data Archive [distributor], March 2001. SN: 3886

Joint Health Surveys Unit of Social and Community Planning Research and University College London, Health Survey for England, 1997 [computer file]. 2nd ed. Colchester, Essex: UK Data Archive [distributor], 4 December 2000. SN: 3979

Kenya National Bureau of Statistics, Ministry of Devolution and Planning (Kenya), Ministry of Health (Kenya), National AIDS and STI Control Program (Kenya). Kenya AIDS Indicator Survey 2012-2013. Nairobi, Kenya: Kenya National Bureau of Statistics

Kenya National Bureau of Statistics, Ministry of Health (Kenya), World Health Organization (WHO). Kenya STEPS Noncommunicable Disease Risk Factors Survey 2015

Kessler, Ronald C. National Comorbidity Survey: Baseline (NCS-1), 1990-1992. ICPSR06693-v6. Ann Arbor, MI: Inter-university Consortium for Political and Social Research [distributor], 2008-09-12. http://doi.org/10.3886/ICPSR06693.v6

Ministry of Health (Brazil), Secretariat of Health Surveillance (Brazil), Secretariat of Health Care (Brazil). Brazil Risk Factor Morbidity Noncommunicable Disease Survey 2002-2005. Rio de Janeiro, Brazil: National Cancer Institute (Brazil)

Ministry of Health (Brazil), Secretariat of Health Surveillance, Ministry of Health (Brazil), University of São Paulo. Brazil Surveillance System of Risk Factors for Chronic Diseases by Telephone Interviews 2006

Ministry of Health (Brazil), Secretariat of Health Surveillance, Ministry of Health (Brazil). Brazil Surveillance System of Risk Factors for Chronic Diseases by Telephone Interviews 2007

Ministry of Health (Brazil), Secretariat of Health Surveillance, Ministry of Health (Brazil). Brazil Surveillance System of Risk Factors for Chronic Diseases by Telephone Interviews 2008

Ministry of Health (Brazil), Secretariat of Health Surveillance, Ministry of Health (Brazil). Brazil Surveillance System of Risk Factors for Chronic Diseases by Telephone Interviews 2009

Ministry of Health (Brazil), Secretariat of Health Surveillance, Ministry of Health (Brazil). Brazil Surveillance System of Risk Factors for Chronic Diseases by Telephone Interviews 2010

Ministry of Health (Brazil), Secretariat of Health Surveillance, Ministry of Health (Brazil). Brazil Surveillance System of Risk Factors for Chronic Diseases by Telephone Interviews 2011

Ministry of Health (Brazil), Secretariat of Health Surveillance, Ministry of Health (Brazil). Brazil Surveillance System of Risk Factors for Chronic Diseases by Telephone Interviews 2012

Ministry of Health (China), National Center for Chronic and Noncommunicable Disease Control and Prevention (China), World Health Organization (WHO). China WHO Study on Global AGEing and Adult Health 2007-2010

Ministry of Health (Mexico), National Council Against Addictions (Mexico), National Institute of Psychiatry Ramón de la Fuente Muñiz (Mexico), National Institute of Public Health (Mexico). Mexico National Addiction Survey 1998

Ministry of Health (Mexico), National Institute of Statistics, Geography, and Informatics (Mexico). Mexico National Addiction Survey 2002. Aguascalientes, Mexico: National Institute of Statistics, Geography, and Informatics (Mexico)

Ministry of Health (Mexico), Secretary of Public Education (Mexico). Mexico National School Health Survey 2008. Cuernavaca, Mexico: National Institute of Public Health (Mexico), 2010

Ministry of Health (Mexico). Mexico National Survey of Chronic Diseases 1993

Ministry of Health (Syria), World Health Organization (WHO). Syria WHO Multi-country Survey Study on Health and Health System Responsiveness 2000-2001

Ministry of Health (Vanuatu), World Health Organization (WHO). Vanuatu STEPS Noncommunicable Disease Risk Factors Survey 2011

Ministry of Health and Medical Services (Kiribati), World Health Organization (WHO). Kiribati STEPS Noncommunicable Disease Risk Factors Survey 2004-2006

Ministry of Health, Labour and Welfare (Japan). Japan National Health and Nutrition Survey 2003

Ministry of Health, Labour and Welfare (Japan). Japan National Health and Nutrition Survey 2004

Ministry of Health, Labour and Welfare (Japan). Japan National Health and Nutrition Survey 2005

Ministry of Health, Labour and Welfare (Japan). Japan National Health and Nutrition Survey 2006

Ministry of Health, Labour and Welfare (Japan). Japan National Health and Nutrition Survey 2007

Ministry of Health, Labour and Welfare (Japan). Japan National Health and Nutrition Survey 2008

Ministry of Health, Labour and Welfare (Japan). Japan National Health and Nutrition Survey 2009

Ministry of Health, Labour and Welfare (Japan). Japan National Health and Nutrition Survey 2010

Ministry of Health, Labour and Welfare (Japan). Japan National Health and Nutrition Survey 2011

Ministry of Public Health (Lebanon), World Health Organization (WHO). Lebanon WHO Multi-country Survey Study on Health and Health System Responsiveness 2000-2001

Nangia V, Jonas JB, Sinha A, Matin A, Kulkarni M, Panda-Jonas S. Ocular axial length and its associations in an adult population of Central Rural India. The Central India Eye and Medical Study [Unpublished data]. Ophthalmology 2010;117(7):1360-6.

NatCen Social Research and Royal Free and University College Medical School. Department of Epidemiology and Public Health, Health Survey for England, 2010 [computer file]. 2nd Edition. Colchester, Essex: UK Data Archive [distributor], July 2012. SN: 6986, http://dx.doi.org/10.5255/UKDA-SN-6986-2

NatCen Social Research and University College London. Department of Epidemiology and Public Health, Health Survey for England, 2011 [computer file]. Colchester, Essex: UK Data Archive [distributor], April 2013. SN: 7260, http://dx.doi.org/10.5255/UKDA-SN-7260-1

National Center for Health Statistics (NCHS), Centers for Disease Control and Prevention (CDC). United States National Health and Nutrition Examination Survey 1999-2000. Hyattsville, United States: National Center for Health Statistics (NCHS), Centers for Disease Control and Prevention (CDC)

National Center for Health Statistics (NCHS), Centers for Disease Control and Prevention (CDC). United States National Health and Nutrition Examination Survey 2001-2002. Hyattsville, United States: National Center for Health Statistics (NCHS), Centers for Disease Control and Prevention (CDC)

National Center for Health Statistics (NCHS), Centers for Disease Control and Prevention (CDC). United States National Health and Nutrition Examination Survey 2003-2004. Hyattsville, United States: National Center for Health Statistics (NCHS), Centers for Disease Control and Prevention (CDC)

National Center for Health Statistics (NCHS), Centers for Disease Control and Prevention (CDC). United States National Health and Nutrition Examination Survey 2005-2006. Hyattsville, United States: National Center for Health Statistics (NCHS), Centers for Disease Control and Prevention (CDC), 2007

National Center for Health Statistics (NCHS), Centers for Disease Control and Prevention (CDC). United States National Health and Nutrition Examination Survey 2007-2008. Hyattsville, United States: National Center for Health Statistics (NCHS), Centers for Disease Control and Prevention (CDC), 2009

National Center for Health Statistics (NCHS), Centers for Disease Control and Prevention (CDC). United States National Health and Nutrition Examination Survey 2009-2010. Hyattsville, United States: National Center for Health Statistics (NCHS), Centers for Disease Control and Prevention (CDC), 2011

National Centre for Social Research and National Foundation for Educational Research, Smoking, Drinking and Drug Use among Young People, 2000 [computer file]. Colchester, Essex: UK Data Archive [distributor], April 2002. SN: 4485, http://dx.doi.org/10.5255/UKDA-SN-4485-1

National Centre for Social Research and University College London. Department of Epidemiology and Public Health, Health Survey for England, 2001 [computer file]. 2nd Edition. Colchester, Essex: UK Data Archive [distributor], June 2004. SN: 4628

National Centre for Social Research and University College London. Department of Epidemiology and Public Health, Health Survey for England, 2002 [computer file]. Colchester, Essex: UK Data Archive [distributor], May 2004. SN: 4912

National Centre for Social Research and University College London. Department of Epidemiology and Public Health, Health Survey for England, 2003 [computer file]. Colchester, Essex: UK Data Archive [distributor], March 2005. SN: 5098

National Centre for Social Research and University College London. Department of Epidemiology and Public Health, Health Survey for England, 2004 [computer file]. Colchester, Essex: UK Data Archive [distributor], July 2006. SN: 5439

National Centre for Social Research and University College London. Department of Epidemiology and Public Health, Health Survey for England, 2006 [computer file]. 4th Edition. Colchester, Essex: UK Data Archive [distributor], July 2011. SN: 5809, http://dx.doi.org/10.5255/UKDA-SN-5809-1

National Centre for Social Research and University College London. Department of Epidemiology and Public Health, Health Survey for England, 2007 [computer file]. 2nd Edition. Colchester, Essex: UK Data Archive [distributor], April 2010. SN: 6112, http://dx.doi.org/10.5255/UKDA-SN-6112-1

National Centre for Social Research and University College London. Department of Epidemiology and Public Health, Health Survey for England, 2008 [computer file]. 3rd Edition. Colchester, Essex: UK Data Archive [distributor], July 2011. SN: 6397, http://dx.doi.org/10.5255/UKDA-SN-6397-1

National Centre for Social Research and University College London. Department of Epidemiology and Public Health, Health Survey for England, 2009 [computer file]. 2nd Edition. Colchester, Essex: UK Data Archive [distributor], July 2011. SN: 6732, http://dx.doi.org/10.5255/UKDA-SN-6732-1

National Centre for Social Research and University College London. Department of Epidemiology and Public Health, Health Survey for England, 1999 [computer file]. 3rd Edition. Colchester, Essex: UK Data Archive [distributor], February 2002. SN: 4365

National Centre for Social Research and University College London. Department of Epidemiology and Public Health, Health Survey for England, 2005 [computer file]. Colchester, Essex: UK Data Archive [distributor], July 2007. SN: 5675

National Centre for Social Research et al. , National Survey of Sexual Attitudes and Lifestyles II, 2000-2001 [computer file]. Colchester, Essex: UK Data Archive [distributor], August 2005. SN: 5223, http://dx.doi.org/10.5255/UKDA-SN-5223-1

National Centre for Social Research, University College London Department of Epidemiology and Public Health, Health Survey for England, 1998 [computer file]. 4th ed. Colchester, Essex: UK Data Archive [distributor], 30 November 2002. SN: 4150

National Centre for Social Research, University College London Department of Epidemiology and Public Health, Health Survey for England, 2000 [computer file]. Colchester, Essex: UK Data Archive [distributor], 23 April 2002. SN: 4487

National Council Against Addictions (Mexico), National Institute of Psychiatry Ramón de la Fuente Muñiz (Mexico), National Institute of Public Health (Mexico). Mexico National Addiction Survey 2008. Cuernavaca, Mexico: National Institute of Public Health (Mexico)

National Institute of Health Research and Development (NIHRD), Ministry of Health (Indonesia), World Health Organization (WHO). Indonesia WHO Multi-country Survey Study on Health and Health System Responsiveness 2001

National Institute of Public Health (Mexico), World Health Organization (WHO). Mexico WHO Multi-country Survey Study on Health and Health System Responsiveness 2000-2001. Geneva, Switzerland: World Health Organization (WHO)

National Institute of Public Health (Mexico), World Health Organization (WHO). Mexico WHO Study on Global AGEing and Adult Health 2009-2010. Geneva, Switzerland: World Health Organization (WHO), 2011

National Institute of Public Health (Mexico). Mexico National Survey of Health and Nutrition 2005-2006. Cuernavaca, Mexico: National Institute of Public Health (Mexico)

National Institute of Public Health (Mexico). Mexico National Survey of Health and Nutrition 2011-2012. Cuernavaca, Mexico: National Institute of Public Health (Mexico)

National Institute of Statistics, Geography, and Informatics (Mexico), Population Studies Center, University of Pennsylvania, University of Maryland, University of Wisconsin. Mexico Health and Aging Study 2001

National Institute of Statistics, Geography, and Informatics (Mexico), Population Studies Center, University of Pennsylvania, University of Maryland, University of Wisconsin. Mexico Health and Aging Study 2003

National Institute of Statistics, Geography, and Informatics (Mexico), Population Studies Center, University of Pennsylvania, University of Maryland, University of Wisconsin. Mexico Health and Aging Study 2012. Mexico City, México: National Institute of Statistics, Geography, and Informatics (Mexico)

National Institute on Alcohol Abuse and Alcoholism (NIAAA), National Institutes of Health (NIH), U.S. Department of Health and Human Services. United States National Epidemiologic Survey on Alcohol and Related Conditions 2001-2002

National Institute on Alcohol Abuse and Alcoholism (NIAAA), National Institutes of Health (NIH), U.S. Department of Health and Human Services. United States National Epidemiologic Survey on Alcohol and Related Conditions 2004-2005

National Opinion Research Center, University of Chicago (NORC), Renmin University, Beijing, Peking Union Medical College, Beijing, University of North Carolina. China Health and Family Life Survey 1999-2000. Chicago, USA: Population Research Center, University of Chicago

National Research and Development Center for Welfare and Health (STAKES) (Finland), Swedish Institute for Social Research, World Health Organization (WHO). Finland European Comparative Alcohol Study (ECAS) Survey 2000 - GENACIS

National Research and Development Center for Welfare and Health (STAKES) (Finland), Swedish Institute for Social Research, World Health Organization (WHO). France European Comparative Alcohol Study (ECAS) Survey 2000 - GENACIS

National Research and Development Center for Welfare and Health (STAKES) (Finland), Swedish Institute for Social Research, World Health Organization (WHO). Germany European Comparative Alcohol Study (ECAS) Survey 2000 - GENACIS

National Research and Development Center for Welfare and Health (STAKES) (Finland), Swedish Institute for Social Research, World Health Organization (WHO). Italy European Comparative Alcohol Study (ECAS) Survey 2000 - GENACIS

National Research and Development Center for Welfare and Health (STAKES) (Finland), Swedish Institute for Social Research, World Health Organization (WHO). Sweden European Comparative Alcohol Study (ECAS) Survey 2000 - GENACIS

National Research and Development Center for Welfare and Health (STAKES) (Finland), Swedish Institute for Social Research, World Health Organization (WHO). United Kingdom European Comparative Alcohol Study (ECAS) Survey 2000 - GENACIS

NHS National Services Scotland. Information Services Division, Scottish Schools Adolescent Lifestyle and Substance Use Survey, 2013 [computer file]. Colchester, Essex: UK Data Archive [distributor], February 2015. SN: 7660, http://dx.doi.org/10.5255/UKDA-SN-7660-1

Obot IS, Room R, eds, World Health Organization (WHO). Alcohol, Gender, and Drinking Problems: Perspectives From Low and Middle Income Countries 1999-2003. Geneva, Switzerland: World Health Organization (WHO), 2005

Office for National Statistics. Social and Vital Statistics Division and Food Standards Agency, National Diet and Nutrition Survey : Adults Aged 19 to 64 Years, 2000-2001 [computer file]. Colchester, Essex: UK Data Archive [distributor], May 2005. SN: 5140 , http://dx.doi.org/10.5255/UKDA-SN-5140-1

Office for National Statistics. Social and Vital Statistics Division, General Household Survey, 2006 [computer file]. 3rd Edition. Colchester, Essex: UK Data Archive [distributor], February 2009. SN: 5804, http://dx.doi.org/10.5255/UKDA-SN-5804-1

Office for National Statistics. Social Survey Division et al. , National Diet and Nutrition Survey : Young People Aged 4 to 18 Years, 1997 [computer file]. Colchester, Essex: UK Data Archive [distributor], January 2001. SN: 4243, http://dx.doi.org/10.5255/UKDA-SN-4243-1

Office of Population Censuses and Surveys. Social Survey Division, Health Survey for England, 1991-1992: Combined Data File. Colchester, Essex: UK Data Archive [distributor], October 1997. SN: 3238, http://dx.doi.org/10.5255/UKDA-SN-3238-1

Office of Population Censuses and Surveys. Social Survey Division, Health Survey for England, 1993 [Computer file]. Colchester, Essex: UK Data Archive [distributor], April 1995. SN: 3316, http://dx.doi.org/10.5255/UKDA-SN-3316-1

Pan American Health Organization (PAHO), Center for Demography and Ecology, University of Wisconsin-Madison, Inter-University Consortium for Political and Social Research (ICPSR), University of São Paulo. Brazil - São Paulo Survey on Health, Well-Being, and Aging in Latin America and the Caribbean 1999-2000. Ann Arbor, United States: Inter-University Consortium for Political and Social Research (ICPSR)

Panel Study of Income Dynamics, 1999 public use dataset. Produced and distributed by the University of Michigan with primary funding from the National Science Foundation, the National Institute of Aging, and the National Institute of Child Health and Human Development. Ann Arbor, MI, (2011)

Panel Study of Income Dynamics, 2001 public use dataset. Produced and distributed by the University of Michigan with primary funding from the National Science Foundation, the National Institute of Aging, and the National Institute of Child Health and Human Development. Ann Arbor, MI, (2011)

Panel Study of Income Dynamics, 2003 public use dataset. Produced and distributed by the University of Michigan with primary funding from the National Science Foundation, the National Institute of Aging, and the National Institute of Child Health and Human Development. Ann Arbor, MI, (2011)

Panel Study of Income Dynamics, 2005 public use dataset. Produced and distributed by the University of Michigan with primary funding from the National Science Foundation, the National Institute of Aging, and the National Institute of Child Health and Human Development. Ann Arbor, MI, (2011)

Panel Study of Income Dynamics, 2007 public use dataset. Produced and distributed by the University of Michigan with primary funding from the National Science Foundation, the National Institute of Aging, and the National Institute of Child Health and Human Development. Ann Arbor, MI, (2011)

Policy Planning and Research Unit (Northern Ireland). Central Survey Unit, Continuous Household Survey, 1990-1991 [computer file]. 2nd Edition. Colchester, Essex: UK Data Archive [distributor], November 2004. SN: 3281, http://dx.doi.org/10.5255/UKDA-SN-3281-1

Policy Planning and Research Unit (Northern Ireland). Central Survey Unit, Continuous Household Survey, 1992-1993 [computer file]. 2nd Edition. Colchester, Essex: UK Data Archive [distributor], November 2004. SN: 3773, http://dx.doi.org/10.5255/UKDA-SN-3773-1

Pontificia Universidad Javeriana (Colombia), World Health Organization (WHO). Colombia WHO Multi-country Survey Study on Health and Health System Responsiveness 2000-2001. Geneva, Switzerland: World Health Organization (WHO)

Public Health Authority of the Slovak Republic, World Health Organization (WHO). Slovakia WHO Multi-country Survey Study on Health and Health System Responsiveness 2000

Russian Academy of Medical Science, World Health Organization (WHO). Russia WHO Study on Global AGEing and Adult Health 2007-2010

Southern Africa Labour Development Research Unit (SALDRU), University of Cape Town. South Africa Integrated Family Survey 1999

United States Department of Health and Human Services. National Institutes of Health. National Institute on Drug Abuse. National Household Survey on Drug Abuse, 1985. ICPSR06844-v2. Ann Arbor, MI: Inter-university Consortium for Political and Social Research [distributor], 2008-07-25. doi:10.3886/ICPSR06844.v2

United States Department of Health and Human Services. National Institutes of Health. National Institute on Drug Abuse. National Household Survey on Drug Abuse, 1988. ICPSR09522-v3. Ann Arbor, MI: Inter-university Consortium for Political and Social Research [distributor], 2008-07-23. doi:10.3886/ICPSR09522.v3

United States Department of Health and Human Services. National Institutes of Health. National Institute on Drug Abuse. National Household Survey on Drug Abuse, 1990. ICPSR09833-v4. Ann Arbor, MI: Inter-university Consortium for Political and Social Research [distributor], 2008-07-25. doi:10.3886/ICPSR09833.v4

United States Department of Health and Human Services. Substance Abuse and Mental Health Services Administration. Center for Behavioral Health Statistics and Quality. National Survey on Drug Use and Health, 2010. ICPSR32722-v3. Ann Arbor, MI: Inter-university Consortium for Political and Social Research [distributor], 2012-10-19. doi:10.3886/ICPSR32722.v3

United States Department of Health and Human Services. Substance Abuse and Mental Health Services Administration. Center for Behavioral Health Statistics and Quality. National Survey on Drug Use and Health, 2011. ICPSR34481-v1. Ann Arbor, MI: Inter-university Consortium for Political and Social Research [distributor], 2012-11-28. doi:10.3886/ICPSR34481.v1

United States Department of Health and Human Services. Substance Abuse and Mental Health Services Administration. Center for Behavioral Health Statistics and Quality. National Survey on Drug Use and Health, 2012. ICPSR34933-v1. Ann Arbor, MI: Inter-university Consortium for Political and Social Research [distributor], 2013-11-26. doi:10.3886/ICPSR34933.v1

United States Department of Health and Human Services. Substance Abuse and Mental Health Services Administration. Office of Applied Studies. National Household Survey on Drug Abuse, 1992. ICPSR06887-v2. Ann Arbor, MI: Inter-university Consortium for Political and Social Research [distributor], 2008-08-18. doi:10.3886/ICPSR06887.v2

United States Department of Health and Human Services. Substance Abuse and Mental Health Services Administration. Office of Applied Studies. National Household Survey on Drug Abuse, 1993. ICPSR06852-v1. Ann Arbor, MI: Inter-university Consortium for Political and Social Research [distributor], 2008-10-28. doi:10.3886/ICPSR06852.v1

United States Department of Health and Human Services. Substance Abuse and Mental Health Services Administration. Office of Applied Studies. National Household Survey on Drug Abuse, 1994. ICPSR06949-v1. Ann Arbor, MI: Inter-university Consortium for Political and Social Research [distributor], 2008-10-28. doi:10.3886/ICPSR06949.v1

United States Department of Health and Human Services. Substance Abuse and Mental Health Services Administration. Office of Applied Studies. National Household Survey on Drug Abuse, 1995. ICPSR06950-v1. Ann Arbor, MI: Inter-university Consortium for Political and Social Research [distributor], 2008-10-23. doi:10.3886/ICPSR06950.v1

United States Department of Health and Human Services. Substance Abuse and Mental Health Services Administration. Office of Applied Studies. National Household Survey on Drug Abuse, 1996. ICPSR02391-v1. Ann Arbor, MI: Inter-university Consortium for Political and Social Research [distributor], 2008-10-23. doi:10.3886/ICPSR02391.v1

United States Department of Health and Human Services. Substance Abuse and Mental Health Services Administration. Office of Applied Studies. National Household Survey on Drug Abuse, 1997. ICPSR02755-v2. Ann Arbor, MI: Inter-university Consortium for Political and Social Research [distributor], 2008-10-23. doi:10.3886/ICPSR02755.v2

United States Department of Health and Human Services. Substance Abuse and Mental Health Services Administration. Office of Applied Studies. National Household Survey on Drug Abuse, 1998. ICPSR02934-v3. Ann Arbor, MI: Inter-university Consortium for Political and Social Research [distributor], 2008-04-25. doi:10.3886/ICPSR02934.v3

United States Department of Health and Human Services. Substance Abuse and Mental Health Services Administration. Office of Applied Studies. National Household Survey on Drug Abuse, 1999. ICPSR03239-v4. Ann Arbor, MI: Inter-university Consortium for Political and Social Research [distributor], 2006-12-07. doi:10.3886/ICPSR03239.v4

United States Department of Health and Human Services. Substance Abuse and Mental Health Services Administration. Office of Applied Studies. National Household Survey on Drug Abuse, 2000. ICPSR03262-v4. Ann Arbor, MI: Inter-university Consortium for Political and Social Research [distributor], 2006-12-07. doi:10.3886/ICPSR03262.v4

United States Department of Health and Human Services. Substance Abuse and Mental Health Services Administration. Office of Applied Studies. National Household Survey on Drug Abuse, 2001. ICPSR03580-v3. Ann Arbor, MI: Inter-university Consortium for Political and Social Research [distributor], 2006-12-07. doi:10.3886/ICPSR03580.v3

United States Department of Health and Human Services. Substance Abuse and Mental Health Services Administration. Office of Applied Studies. National Survey on Drug Use and Health, 2002. ICPSR03903-v3. Ann Arbor, MI: Inter-university Consortium for Political and Social Research [distributor], 2006-10-26. doi:10.3886/ICPSR03903.v3

United States Department of Health and Human Services. Substance Abuse and Mental Health Services Administration. Office of Applied Studies. National Survey on Drug Use and Health, 2003. ICPSR04138-v2. Ann Arbor, MI: Inter-university Consortium for Political and Social Research [distributor], 2006-10-17. doi:10.3886/ICPSR04138.v2

United States Department of Health and Human Services. Substance Abuse and Mental Health Services Administration. Office of Applied Studies. National Survey on Drug Use and Health, 2004. ICPSR04373-v1. Ann Arbor, MI: Inter-university Consortium for Political and Social Research [distributor], 2006-05-12. doi:10.3886/ICPSR04373.v1

United States Department of Health and Human Services. Substance Abuse and Mental Health Services Administration. Office of Applied Studies. National Survey on Drug Use and Health, 2005. ICPSR04596-v3. Ann Arbor, MI: Inter-university Consortium for Political and Social Research [distributor], 2013-01-22. doi:10.3886/ICPSR04596.v3

United States Department of Health and Human Services. Substance Abuse and Mental Health Services Administration. Office of Applied Studies. National Survey on Drug Use and Health, 2006. ICPSR21240-v5. Ann Arbor, MI: Inter-university Consortium for Political and Social Research [distributor], 2013-01-07. doi:10.3886/ICPSR21240.v5

United States Department of Health and Human Services. Substance Abuse and Mental Health Services Administration. Office of Applied Studies. National Survey on Drug Use and Health, 2007. ICPSR23782-v3. Ann Arbor, MI: Inter-university Consortium for Political and Social Research [distributor], 2013-01-04. doi:10.3886/ICPSR23782.v3

United States Department of Health and Human Services. Substance Abuse and Mental Health Services Administration. Office of Applied Studies. National Survey on Drug Use and Health, 2008. ICPSR26701-v3. Ann Arbor, MI: Inter-university Consortium for Political and Social Research [distributor], 2012-12-10. doi:10.3886/ICPSR26701.v3

United States Department of Health and Human Services. Substance Abuse and Mental Health Services Administration. Office of Applied Studies. National Survey on Drug Use and Health, 2009. ICPSR29621-v3. Ann Arbor, MI: Inter-university Consortium for Political and Social Research [distributor], 2012-11-16. doi:10.3886/ICPSR29621.v3

World Health Organization (WHO), Joint United Nations Program on HIV/AIDS (UNAIDS), United Nations Educational, Scientific and Cultural Organization (UNESCO), United Nations Children's Fund (UNICEF), and Centers for Disease Control and Prevention (CDC). China Global School-Based Student Health Survey 2003. Geneva, Switzerland: World Health Organization (WHO)

World Health Organization (WHO). Bangladesh World Health Survey 2003. Geneva, Switzerland: World Health Organization (WHO), 2005

World Health Organization (WHO). Bosnia and Herzegovina World Health Survey 2003. Geneva, Switzerland: World Health Organization (WHO), 2005

World Health Organization (WHO). Burkina Faso World Health Survey 2002-2003. Geneva, Switzerland: World Health Organization (WHO), 2005

World Health Organization (WHO). Chad World Health Survey 2003. Geneva, Switzerland: World Health Organization (WHO), 2005

World Health Organization (WHO). China World Health Survey 2002. Geneva, Switzerland: World Health Organization (WHO), 2005

World Health Organization (WHO). Comoros World Health Survey 2003. Geneva, Switzerland: World Health Organization (WHO), 2005

World Health Organization (WHO). Congo World Health Survey 2003. Geneva, Switzerland: World Health Organization (WHO), 2005

World Health Organization (WHO). Côte d'Ivoire World Health Survey 2003. Geneva, Switzerland: World Health Organization (WHO), 2005

World Health Organization (WHO). Croatia World Health Survey 2003. Geneva, Switzerland: World Health Organization (WHO), 2005

World Health Organization (WHO). Czech Republic World Health Survey 2002-2003. Geneva, Switzerland: World Health Organization (WHO), 2005

World Health Organization (WHO). Dominican Republic World Health Survey 2003. Geneva, Switzerland: World Health Organization (WHO), 2005

World Health Organization (WHO). Ecuador World Health Survey 2003. Geneva, Switzerland: World Health Organization (WHO), 2005

World Health Organization (WHO). Estonia World Health Survey 2003. Geneva, Switzerland: World Health Organization (WHO), 2005

World Health Organization (WHO). Ethiopia World Health Survey 2003. Geneva, Switzerland: World Health Organization (WHO), 2005

World Health Organization (WHO). Georgia World Health Survey 2003. Geneva, Switzerland: World Health Organization (WHO), 2005

World Health Organization (WHO). Ghana World Health Survey 2003. Geneva, Switzerland: World Health Organization (WHO), 2005

World Health Organization (WHO). Guatemala World Health Survey 2003. Geneva, Switzerland: World Health Organization (WHO), 2005

World Health Organization (WHO). Hungary World Health Survey 2003. Geneva, Switzerland: World Health Organization (WHO), 2005

World Health Organization (WHO). Kazakhstan World Health Survey 2002-2003. Geneva, Switzerland: World Health Organization (WHO), 2005

World Health Organization (WHO). Kenya World Health Survey 2004. Geneva, Switzerland: World Health Organization (WHO), 2005

World Health Organization (WHO). Laos World Health Survey 2003

World Health Organization (WHO). Latvia World Health Survey 2003. Geneva, Switzerland: World Health Organization (WHO), 2005

World Health Organization (WHO). Malawi World Health Survey 2003. Geneva, Switzerland: World Health Organization (WHO), 2005

World Health Organization (WHO). Malaysia World Health Survey 2003. Geneva, Switzerland: World Health Organization (WHO), 2005

World Health Organization (WHO). Mali World Health Survey 2003. Geneva, Switzerland: World Health Organization (WHO), 2005

World Health Organization (WHO). Mauritania World Health Survey 2003. Geneva, Switzerland: World Health Organization (WHO), 2005

World Health Organization (WHO). Mauritius World Health Survey 2003. Geneva, Switzerland: World Health Organization (WHO), 2005

World Health Organization (WHO). Mexico World Health Survey 2002-2003. Geneva, Switzerland: World Health Organization (WHO), 2005

World Health Organization (WHO). Morocco World Health Survey 2003. Geneva, Switzerland: World Health Organization (WHO), 2005

World Health Organization (WHO). Myanmar World Health Survey 2003. Geneva, Switzerland: World Health Organization (WHO), 2005

World Health Organization (WHO). Namibia World Health Survey 2003. Geneva, Switzerland: World Health Organization (WHO), 2005

World Health Organization (WHO). Nepal World Health Survey 2003. Geneva, Switzerland: World Health Organization (WHO), 2005

World Health Organization (WHO). Pakistan World Health Survey 2003-2004. Geneva, Switzerland: World Health Organization (WHO), 2005

World Health Organization (WHO). Paraguay World Health Survey 2002-2003. Geneva, Switzerland: World Health Organization (WHO), 2005

World Health Organization (WHO). Philippines World Health Survey 2003. Geneva, Switzerland: World Health Organization (WHO), 2005

World Health Organization (WHO). Russia World Health Survey 2003. Geneva, Switzerland: World Health Organization (WHO), 2005

World Health Organization (WHO). Senegal World Health Survey 2003. Geneva, Switzerland: World Health Organization (WHO), 2005

World Health Organization (WHO). Slovakia World Health Survey 2003. Geneva, Switzerland: World Health Organization (WHO), 2005

World Health Organization (WHO). Slovenia World Health Survey 2003. Geneva, Switzerland: World Health Organization (WHO), 2005

World Health Organization (WHO). South Africa World Health Survey 2002-2003. Geneva, Switzerland: World Health Organization (WHO), 2005

World Health Organization (WHO). Spain World Health Survey 2002-2003. Geneva, Switzerland: World Health Organization (WHO), 2005

World Health Organization (WHO). Sri Lanka World Health Survey 2003. Geneva, Switzerland: World Health Organization (WHO), 2005

World Health Organization (WHO). Swaziland World Health Survey 2003. Geneva, Switzerland: World Health Organization (WHO), 2005

World Health Organization (WHO). Tunisia World Health Survey 2003. Geneva, Switzerland: World Health Organization (WHO), 2005

World Health Organization (WHO). Turkey World Health Survey 2003. Geneva, Switzerland: World Health Organization (WHO), 2005

World Health Organization (WHO). Ukraine World Health Survey 2002-2003. Geneva, Switzerland: World Health Organization (WHO), 2005

World Health Organization (WHO). United Arab Emirates World Health Survey 2003. Geneva, Switzerland: World Health Organization (WHO), 2005

World Health Organization (WHO). Uruguay World Health Survey 2002-2003. Geneva, Switzerland: World Health Organization (WHO), 2005

World Health Organization (WHO). Vietnam World Health Survey 2002-2003. Geneva, Switzerland: World Health Organization (WHO), 2005

World Health Organization (WHO). WHO Global Health Observatory - Recorded Alcohol Per Capita Consumption 1960-1979. Geneva, Switzerland: World Health Organization (WHO)

World Health Organization (WHO). WHO Global Health Observatory - Recorded Alcohol Per Capita Consumption 1980-1999. Geneva, Switzerland: World Health Organization (WHO)

World Health Organization (WHO). WHO Global Health Observatory - Recorded Alcohol Per Capita Consumption from 2000. Geneva, Switzerland: World Health Organization (WHO)

World Health Organization (WHO). Zambia World Health Survey 2003. Geneva, Switzerland: World Health Organization (WHO), 2005

World Health Organization (WHO). Zimbabwe World Health Survey 2003. Geneva, Switzerland: World Health Organization (WHO), 2005

World Health Organization Regional Office for Europe (EURO-WHO). Health Behaviour in School-aged Children: WHO Collaborative Cross-National survey/study (HBSC) 1997

World Health Organization Regional Office for Europe (EURO-WHO). Health Behaviour in School-aged Children: WHO Collaborative Cross-National survey/study (HBSC) 1997

World Health Organization Regional Office for Europe (EURO-WHO). Health Behaviour in School-aged Children: WHO Collaborative Cross-National survey/study (HBSC) 1997-1998

World Health Organization Regional Office for Europe (EURO-WHO). Health Behaviour in School-aged Children: WHO Collaborative Cross-National survey/study (HBSC) 1998

World Health Organization Regional Office for Europe (EURO-WHO). Health Behaviour in School-aged Children: WHO Collaborative Cross-National survey/study (HBSC) 1998

World Health Organization Regional Office for Europe (EURO-WHO). Health Behaviour in School-aged Children: WHO Collaborative Cross-National survey/study (HBSC) 1998

World Health Organization Regional Office for Europe (EURO-WHO). Health Behaviour in School-aged Children: WHO Collaborative Cross-National survey/study (HBSC) 1998

World Health Organization Regional Office for Europe (EURO-WHO). Health Behaviour in School-aged Children: WHO Collaborative Cross-National survey/study (HBSC) 1998

World Health Organization Regional Office for Europe (EURO-WHO). Health Behaviour in School-aged Children: WHO Collaborative Cross-National survey/study (HBSC) 1998

World Health Organization Regional Office for Europe (EURO-WHO). Health Behaviour in School-aged Children: WHO Collaborative Cross-National survey/study (HBSC) 2005

World Health Organization Regional Office for Europe (EURO-WHO). Health Behaviour in School-aged Children: WHO Collaborative Cross-National survey/study (HBSC) 2005-2006

World Health Organization Regional Office for Europe (EURO-WHO). Health Behaviour in School-aged Children: WHO Collaborative Cross-National survey/study (HBSC) 2006

World Health Organization Regional Office for Europe (EURO-WHO). Health Behaviour in School-aged Children: WHO Collaborative Cross-National survey/study (HBSC) 2006

World Health Organization Regional Office for Europe (EURO-WHO). Health Behaviour in School-aged Children: WHO Collaborative Cross-National survey/study (HBSC) 2006

World Health Organization Regional Office for Europe (EURO-WHO). Health Behaviour in School-aged Children: WHO Collaborative Cross-National survey/study (HBSC) 2009

World Health Organization Regional Office for Europe (EURO-WHO). Health Behaviour in School-aged Children: WHO Collaborative Cross-National survey/study (HBSC) 2009-2010

World Health Organization Regional Office for Europe (EURO-WHO). Health Behaviour in School-aged Children: WHO Collaborative Cross-National survey/study (HBSC) 2010

World Health Organization Regional Office for Europe (EURO-WHO). Health Behaviour in School-aged Children: WHO Collaborative Cross-National survey/study (HBSC) 2010

World Health Organization Regional Office for Europe (EURO-WHO). Health Behaviour in School-aged Children: WHO Collaborative Cross-National survey/study (HBSC) 2010

Yi, Zeng, James W. Vaupel, Xiao Zhenyu, Liu Yuzhi, and Zhang Chunyuan. Chinese Longitudinal Healthy Longevity Survey (CLHLS), 1998-2005. ICPSR24901-v2. Ann Arbor, MI: Inter-university Consortium for Political and Social Research [distributor], 2009-06-04. doi:10.3886/ICPSR24901.v2

Smoking

Citations

Adolescent Health Research Group, University of Auckland, Faculty of Medical and Health Sciences, University of Auckland. New Zealand Youth2000 Health and Wellbeing of Secondary School Students 2001

Aoki M, Hisamichi S, Tominaga S, eds. Smoking and Health 1987. Proceedings of the 6th World Conference on Smoking and Health. World Conference on Smoking and Health; 1987 Nov 9-12; Tokyo, Japan. Amsterdam, The Netherlands: Excerpta Medica, 1988

Argentina Observatory on Drugs, Secretariat for Programming Drug Abuse Prevention and the Fight against Drug Trafficking (SEDRONAR). Argentina National Survey of High School Students 2005

Argentina Observatory on Drugs, Secretariat for Programming Drug Abuse Prevention and the Fight against Drug Trafficking (SEDRONAR). Argentina National Survey of High School Students 2007

Argentina Observatory on Drugs, Secretariat for Programming Drug Abuse Prevention and the Fight against Drug Trafficking (SEDRONAR). Argentina National Survey of High School Students 2009

Argentina Observatory on Drugs, Secretariat for Programming Drug Abuse Prevention and the Fight against Drug Trafficking (SEDRONAR). Argentina National Survey of High School Students 2011

Ashkenazi I, Shemer J. [Smoking habits of young Israeli soldiers]. Harefuah. 1997; 132(7): 502-526 as it appears in P.N. Lee Statistics and Computing Ltd. International Mortality and Smoking Statistics Version 4.04. Sutton, United Kingdom: P.N. Lee Statistics and Computing Ltd, 2009

Australia National Campaign Against Drug Abuse Social Issues Survey 1988 as it appears in P.N. Lee Statistics and Computing Ltd. International Mortality and Smoking Statistics Version 4.04. Sutton, United Kingdom: P.N. Lee Statistics and Computing Ltd, 2009

Australia National Campaign Against Drug Abuse Social Issues Survey 1993 as it appears in P.N. Lee Statistics and Computing Ltd. International Mortality and Smoking Statistics Version 4.04. Sutton, United Kingdom: P.N. Lee Statistics and Computing Ltd, 2009

Australia National Drug Strategy Household Survey 1998 as it appears in P.N. Lee Statistics and Computing Ltd. International Mortality and Smoking Statistics Version 4.04. Sutton, United Kingdom: P.N. Lee Statistics and Computing Ltd, 2009

Australia National Health Survey 1989-1990 as it appears in P.N. Lee Statistics and Computing Ltd. International Mortality and Smoking Statistics Version 4.04. Sutton, United Kingdom: P.N. Lee Statistics and Computing Ltd, 2009

Australia National Survey of Mental Health and Wellbeing 1997 as it appears in P.N. Lee Statistics and Computing Ltd. International Mortality and Smoking Statistics Version 4.04. Sutton, United Kingdom: P.N. Lee Statistics and Computing Ltd, 2009

Australia National Tobacco Campaign Evaluation Survey 1997 as it appears in P.N. Lee Statistics and Computing Ltd. International Mortality and Smoking Statistics Version 4.04. Sutton, United Kingdom: P.N. Lee Statistics and Computing Ltd, 2009

Australia National Tobacco Campaign Evaluation Survey 1998 as it appears in P.N. Lee Statistics and Computing Ltd. International Mortality and Smoking Statistics Version 4.04. Sutton, United Kingdom: P.N. Lee Statistics and Computing Ltd, 2009

Australia National Tobacco Campaign Evaluation Survey 1999 as it appears in P.N. Lee Statistics and Computing Ltd. International Mortality and Smoking Statistics Version 4.04. Sutton, United Kingdom: P.N. Lee Statistics and Computing Ltd, 2009

Australia National Tobacco Campaign Evaluation Survey 2000 as it appears in P.N. Lee Statistics and Computing Ltd. International Mortality and Smoking Statistics Version 4.04. Sutton, United Kingdom: P.N. Lee Statistics and Computing Ltd, 2009

Australia Schools Health And Fitness Survey 1985 as it appears in P.N. Lee Statistics and Computing Ltd. International Mortality and Smoking Statistics Version 4.04. Sutton, United Kingdom: P.N. Lee Statistics and Computing Ltd, 2009

Australian Bureau of Statistics, Australian Health Survey Reference Group, Roy Morgan Research, University of Western Australia. Australia Child and Adolescent Survey of Mental Health and Wellbeing 2013-2014

Australian Bureau of Statistics. Australia National Health Survey 1995. Canberra, Australia: Australian Bureau of Statistics

Australian Bureau of Statistics. Australia National Health Survey 2001. Canberra, Australia: Australian Bureau of Statistics

Australian Bureau of Statistics. Australia National Health Survey 2004-2005

Australian Bureau of Statistics. Australia National Health Survey 2007-2008. Canberra, Australia: Australian Bureau of Statistics

Australian Bureau of Statistics. Australia National Health Survey 2011-2013

Australian Institute of Health and Welfare, Department of Health and Ageing (Australia), Roy Morgan Research, Social Research Centre (Australia). Australia National Drug Strategy Household Survey 2007

Australian Institute of Health and Welfare, National Heart Foundation (Australia). Australia Risk Factor Prevalence Survey 1989. Acton, Australia: Australian Data Archive

Australian Institute of Health and Welfare, National Heart Foundation (Australia). Australia Risk Factor Prevalence Survey 1994

Australian Institute of Health and Welfare, Roy Morgan Research. Australia National Drug Strategy Household Survey 2010

Australian Institute of Health and Welfare. Australia National Drug Strategy Household Survey 2001

Australian Institute of Health and Welfare. Australia National Drug Strategy Household Survey 2004

Austria Microcensus 1991 as it appears in P.N. Lee Statistics and Computing Ltd. International Mortality and Smoking Statistics Version 4.04. Sutton, United Kingdom: P.N. Lee Statistics and Computing Ltd, 2009

Austria Smoking Habits: Results of the Microcensus 1997 as it appears in P.N. Lee Statistics and Computing Ltd. International Mortality and Smoking Statistics Version 4.04. Sutton, United Kingdom: P.N. Lee Statistics and Computing Ltd, 2009

Bain C, Feskanich D, Speizer FE, Thun M, Hertzmark E, Rosner BA, Colditz GA. Lung cancer rates in men and women with comparable histories of smoking. J Natl Cancer Inst. 2004; 96(11): 826-34 as it appears in Thun MJ, Hannan LM, Adams-Campbell LL, Boffetta P, Buring JE, Feskanich D, Flanders WD, Jee SH, Katanoda K, Kolonel LN, Lee IM, Marugame T, Palmer JR, Riboli E, Sobue T, Avila-Tang E, Wilkens LR, Samet JM. Lung cancer occurrence in never-smokers: an analysis of 13 cohorts and 22 cancer registry studies. PLoS Med. 2008; 5(9): e185

Bamia C, Trichopoulou A, Lenas D, Trichopoulos D. Tobacco smoking in relation to body fat mass and distribution in a general population sample. Int J Obes Relat Metab Disord. 2004; 28(8): 1091–6

Beck F, Guignard R, Richard JB, Wilquin JL, Peretti-Watel P. Recent Increase in smoking in France: main results of the Health Barometer. Bull Epidemiol Hebd (Paris). 2011; 230-3

Börsch-Supan, A. (2013). Survey of Health, Ageing and Retirement in Europe (SHARE) Wave 1. Release version: 2.6.0. SHARE-ERIC. Data set. DOI: 10.6103/SHARE.w1.260

Börsch-Supan, A. (2013). Survey of Health, Ageing and Retirement in Europe (SHARE) Wave 1. Release version: 2.6.0. SHARE-ERIC. Data set. DOI: 10.6103/SHARE.w1.260

Börsch-Supan, A. (2013). Survey of Health, Ageing and Retirement in Europe (SHARE) Wave 1. Release version: 2.6.0. SHARE-ERIC. Data set. DOI: 10.6103/SHARE.w1.260

Börsch-Supan, A. (2013). Survey of Health, Ageing and Retirement in Europe (SHARE) Wave 1. Release version: 2.6.0. SHARE-ERIC. Data set. DOI: 10.6103/SHARE.w1.260

Börsch-Supan, A. (2013). Survey of Health, Ageing and Retirement in Europe (SHARE) Wave 1. Release version: 2.6.0. SHARE-ERIC. Data set. DOI: 10.6103/SHARE.w1.260

Börsch-Supan, A. (2013). Survey of Health, Ageing and Retirement in Europe (SHARE) Wave 1. Release version: 2.6.0. SHARE-ERIC. Data set. DOI: 10.6103/SHARE.w1.260

Börsch-Supan, A. (2013). Survey of Health, Ageing and Retirement in Europe (SHARE) Wave 1. Release version: 2.6.0. SHARE-ERIC. Data set. DOI: 10.6103/SHARE.w1.260

Börsch-Supan, A. (2013). Survey of Health, Ageing and Retirement in Europe (SHARE) Wave 1. Release version: 2.6.0. SHARE-ERIC. Data set. DOI: 10.6103/SHARE.w1.260

Börsch-Supan, A. (2013). Survey of Health, Ageing and Retirement in Europe (SHARE) Wave 1. Release version: 2.6.0. SHARE-ERIC. Data set. DOI: 10.6103/SHARE.w1.260

Börsch-Supan, A. (2013). Survey of Health, Ageing and Retirement in Europe (SHARE) Wave 1. Release version: 2.6.0. SHARE-ERIC. Data set. DOI: 10.6103/SHARE.w1.260

Börsch-Supan, A. (2013). Survey of Health, Ageing and Retirement in Europe (SHARE) Wave 1. Release version: 2.6.0. SHARE-ERIC. Data set. DOI: 10.6103/SHARE.w1.260

Börsch-Supan, A. (2013). Survey of Health, Ageing and Retirement in Europe (SHARE) Wave 1. Release version: 2.6.0. SHARE-ERIC. Data set. DOI: 10.6103/SHARE.w1.260

Börsch-Supan, A. (2013). Survey of Health, Ageing and Retirement in Europe (SHARE) Wave 2. Release version: 2.6.0. SHARE-ERIC. Data set. DOI: 10.6103/SHARE.w2.260

Börsch-Supan, A. (2013). Survey of Health, Ageing and Retirement in Europe (SHARE) Wave 2. Release version: 2.6.0. SHARE-ERIC. Data set. DOI: 10.6103/SHARE.w2.260

Börsch-Supan, A. (2013). Survey of Health, Ageing and Retirement in Europe (SHARE) Wave 2. Release version: 2.6.0. SHARE-ERIC. Data set. DOI: 10.6103/SHARE.w2.260

Börsch-Supan, A. (2013). Survey of Health, Ageing and Retirement in Europe (SHARE) Wave 2. Release version: 2.6.0. SHARE-ERIC. Data set. DOI: 10.6103/SHARE.w2.260

Börsch-Supan, A. (2013). Survey of Health, Ageing and Retirement in Europe (SHARE) Wave 2. Release version: 2.6.0. SHARE-ERIC. Data set. DOI: 10.6103/SHARE.w2.260

Börsch-Supan, A. (2013). Survey of Health, Ageing and Retirement in Europe (SHARE) Wave 2. Release version: 2.6.0. SHARE-ERIC. Data set. DOI: 10.6103/SHARE.w2.260

Börsch-Supan, A. (2013). Survey of Health, Ageing and Retirement in Europe (SHARE) Wave 2. Release version: 2.6.0. SHARE-ERIC. Data set. DOI: 10.6103/SHARE.w2.260

Börsch-Supan, A. (2013). Survey of Health, Ageing and Retirement in Europe (SHARE) Wave 2. Release version: 2.6.0. SHARE-ERIC. Data set. DOI: 10.6103/SHARE.w2.260

Börsch-Supan, A. (2013). Survey of Health, Ageing and Retirement in Europe (SHARE) Wave 2. Release version: 2.6.0. SHARE-ERIC. Data set. DOI: 10.6103/SHARE.w2.260

Börsch-Supan, A. (2013). Survey of Health, Ageing and Retirement in Europe (SHARE) Wave 2. Release version: 2.6.0. SHARE-ERIC. Data set. DOI: 10.6103/SHARE.w2.260

Börsch-Supan, A. (2013). Survey of Health, Ageing and Retirement in Europe (SHARE) Wave 2. Release version: 2.6.0. SHARE-ERIC. Data set. DOI: 10.6103/SHARE.w2.260

Börsch-Supan, A. (2013). Survey of Health, Ageing and Retirement in Europe (SHARE) Wave 2. Release version: 2.6.0. SHARE-ERIC. Data set. DOI: 10.6103/SHARE.w2.260

Börsch-Supan, A. (2013). Survey of Health, Ageing and Retirement in Europe (SHARE) Wave 4. Release version: 1.1.1. SHARE-ERIC. Data set. DOI: 10.6103/SHARE.w4.111

Börsch-Supan, A. (2013). Survey of Health, Ageing and Retirement in Europe (SHARE) Wave 4. Release version: 1.1.1. SHARE-ERIC. Data set. DOI: 10.6103/SHARE.w4.111

Börsch-Supan, A. (2013). Survey of Health, Ageing and Retirement in Europe (SHARE) Wave 4. Release version: 1.1.1. SHARE-ERIC. Data set. DOI: 10.6103/SHARE.w4.111

Börsch-Supan, A. (2013). Survey of Health, Ageing and Retirement in Europe (SHARE) Wave 4. Release version: 1.1.1. SHARE-ERIC. Data set. DOI: 10.6103/SHARE.w4.111

Börsch-Supan, A. (2013). Survey of Health, Ageing and Retirement in Europe (SHARE) Wave 4. Release version: 1.1.1. SHARE-ERIC. Data set. DOI: 10.6103/SHARE.w4.111

Börsch-Supan, A. (2013). Survey of Health, Ageing and Retirement in Europe (SHARE) Wave 4. Release version: 1.1.1. SHARE-ERIC. Data set. DOI: 10.6103/SHARE.w4.111

Börsch-Supan, A. (2013). Survey of Health, Ageing and Retirement in Europe (SHARE) Wave 4. Release version: 1.1.1. SHARE-ERIC. Data set. DOI: 10.6103/SHARE.w4.111

Börsch-Supan, A. (2013). Survey of Health, Ageing and Retirement in Europe (SHARE) Wave 4. Release version: 1.1.1. SHARE-ERIC. Data set. DOI: 10.6103/SHARE.w4.111

Börsch-Supan, A. (2013). Survey of Health, Ageing and Retirement in Europe (SHARE) Wave 4. Release version: 1.1.1. SHARE-ERIC. Data set. DOI: 10.6103/SHARE.w4.111

Börsch-Supan, A. (2013). Survey of Health, Ageing and Retirement in Europe (SHARE) Wave 4. Release version: 1.1.1. SHARE-ERIC. Data set. DOI: 10.6103/SHARE.w4.111

Börsch-Supan, A. (2013). Survey of Health, Ageing and Retirement in Europe (SHARE) Wave 4. Release version: 1.1.1. SHARE-ERIC. Data set. DOI: 10.6103/SHARE.w4.111

Brule Ville Associés (BVA), French Committee for Health Education (CFES). France Youth Health Barometer 1997

Canada Fitness Survey 1981 as it appears in P.N. Lee Statistics and Computing Ltd. International Mortality and Smoking Statistics Version 4.04. Sutton, United Kingdom: P.N. Lee Statistics and Computing Ltd, 2009

Canada General Social Survey 1985 as it appears in P.N. Lee Statistics and Computing Ltd. International Mortality and Smoking Statistics Version 4.04. Sutton, United Kingdom: P.N. Lee Statistics and Computing Ltd, 2009

Canada General Social Survey 1991 as it appears in P.N. Lee Statistics and Computing Ltd. International Mortality and Smoking Statistics Version 4.04. Sutton, United Kingdom: P.N. Lee Statistics and Computing Ltd, 2009

Canada General Social Survey 1996 as it appears in P.N. Lee Statistics and Computing Ltd. International Mortality and Smoking Statistics Version 4.04. Sutton, United Kingdom: P.N. Lee Statistics and Computing Ltd, 2009

Canada Health Promotion Survey 1985 as it appears in P.N. Lee Statistics and Computing Ltd. International Mortality and Smoking Statistics Version 4.04. Sutton, United Kingdom: P.N. Lee Statistics and Computing Ltd, 2009

Canada Health Promotion Survey 1990 as it appears in P.N. Lee Statistics and Computing Ltd. International Mortality and Smoking Statistics Version 4.04. Sutton, United Kingdom: P.N. Lee Statistics and Computing Ltd, 2009

Canada Labor Force Survey Supplement 1981 as it appears in P.N. Lee Statistics and Computing Ltd. International Mortality and Smoking Statistics Version 4.04. Sutton, United Kingdom: P.N. Lee Statistics and Computing Ltd, 2009

Canada Labor Force Survey Supplement 1983 as it appears in P.N. Lee Statistics and Computing Ltd. International Mortality and Smoking Statistics Version 4.04. Sutton, United Kingdom: P.N. Lee Statistics and Computing Ltd, 2009

Canada Labor Force Survey Supplement 1986 as it appears in P.N. Lee Statistics and Computing Ltd. International Mortality and Smoking Statistics Version 4.04. Sutton, United Kingdom: P.N. Lee Statistics and Computing Ltd, 2009

Canada National Alcohol and Other Drugs Survey 1989 as it appears in P.N. Lee Statistics and Computing Ltd. International Mortality and Smoking Statistics Version 4.04. Sutton, United Kingdom: P.N. Lee Statistics and Computing Ltd, 2009

Canada Smoking Survey 1994 as it appears in P.N. Lee Statistics and Computing Ltd. International Mortality and Smoking Statistics Version 4.04. Sutton, United Kingdom: P.N. Lee Statistics and Computing Ltd, 2009

Capacent Gallup. Iceland Prevalence of Smoking 2008

CBG Health Research Ltd., Ministry of Health (New Zealand), University of Wollongong. New Zealand Health Survey 2011-2012. Wellington, New Zealand: Ministry of Health (New Zealand)

CBG Health Research Ltd., Ministry of Health (New Zealand). New Zealand Health Survey 2012-2013. Wellington, New Zealand: Ministry of Health (New Zealand)

CBG Health Research Ltd., Ministry of Health (New Zealand). New Zealand Health Survey 2013-2014. Wellington, New Zealand: Ministry of Health (New Zealand)

CDC Foundation, Centers for Disease Control and Prevention (CDC), Ministry of Health (Argentina), National Institute of Statistics and Censuses (Argentina), Pan American Health Organization (PAHO). Argentina Global Adult Tobacco Survey 2012. Atlanta, United States: Centers for Disease Control and Prevention (CDC), 2013

Center for Information and Research of Uruguay (CIESU), Inter-American Drug Abuse Control Commission (CICAD), Organization of American States (OAS), United Nations Office on Drugs and Crime (UNODC), Uruguay Observatory on Drugs (OUD). Uruguay National Survey on Drug Use in Secondary Students 2009

Center for Information and Research of Uruguay (CIESU), Inter-American Drug Abuse Control Commission (CICAD), Organization of American States (OAS), United Nations Office on Drugs and Crime (UNODC), Uruguay Observatory on Drugs (OUD). Uruguay National Survey on Drug Use in Secondary Students 2011

Center for Statistics, University of Hasselt (CenStat) (Belgium), Scientific Institute of Public Health (WIV-ISP) (Belgium), Statistics Belgium. Belgium Health Interview Survey 2013

Center for the Study of State and Society (CEDES), Ministry of Health and Environment (Argentina), National Institute of Statistics and Censuses (Argentina). Argentina National Survey of Risk Factors 2005

CentERdata. Netherlands Dutch National Bank Household Survey 1995. Tillburg, Netherlands: CentERdata

CentERdata. Netherlands Dutch National Bank Household Survey 1996. Tillburg, Netherlands: CentERdata

CentERdata. Netherlands Dutch National Bank Household Survey 1997. Tillburg, Netherlands: CentERdata

CentERdata. Netherlands Dutch National Bank Household Survey 1998. Tillburg, Netherlands: CentERdata

CentERdata. Netherlands Dutch National Bank Household Survey 1999. Tillburg, Netherlands: CentERdata

CentERdata. Netherlands Dutch National Bank Household Survey 2000. Tillburg, Netherlands: CentERdata

CentERdata. Netherlands Dutch National Bank Household Survey 2001. Tillburg, Netherlands: CentERdata

CentERdata. Netherlands Dutch National Bank Household Survey 2002. Tillburg, Netherlands: CentERdata

CentERdata. Netherlands Dutch National Bank Household Survey 2003. Tillburg, Netherlands: CentERdata

CentERdata. Netherlands Dutch National Bank Household Survey 2004. Tillburg, Netherlands: CentERdata

CentERdata. Netherlands Dutch National Bank Household Survey 2005. Tillburg, Netherlands: CentERdata

CentERdata. Netherlands Dutch National Bank Household Survey 2006. Tillburg, Netherlands: CentERdata

CentERdata. Netherlands Dutch National Bank Household Survey 2007. Tillburg, Netherlands: CentERdata

CentERdata. Netherlands Dutch National Bank Household Survey 2008. Tillburg, Netherlands: CentERdata

CentERdata. Netherlands Dutch National Bank Household Survey 2009. Tillburg, Netherlands: CentERdata

CentERdata. Netherlands Dutch National Bank Household Survey 2010. Tilburg, Netherlands: CentERdata

CentERdata. Netherlands Dutch National Bank Household Survey 2011. Tilburg, Netherlands: CentERdata

CentERdata. Netherlands Dutch National Bank Household Survey 2012. Tilburg, Netherlands: CentERdata

CentERdata. Netherlands Dutch National Bank Household Survey 2013. Tilburg, Netherlands: CentERdata

CentERdata. Netherlands Dutch National Bank Household Survey 2014

Centers for Disease Control and Prevention (CDC) and World Health Organization (WHO). Argentina Global Youth Tobacco Survey 2000. Atlanta, United States: Centers for Disease Control and Prevention (CDC)

Centers for Disease Control and Prevention (CDC) and World Health Organization (WHO). Argentina Global Youth Tobacco Survey 2007. United States: Centers for Disease Control and Prevention (CDC), 2007

Centers for Disease Control and Prevention (CDC) and World Health Organization (WHO). Cyprus Global Youth Tobacco Survey 2005. United States: Centers for Disease Control and Prevention (CDC), 2005

Centers for Disease Control and Prevention (CDC) and World Health Organization (WHO). Greece Global Youth Tobacco Survey 2005. United States: Centers for Disease Control and Prevention (CDC), 2005

Centers for Disease Control and Prevention (CDC) and World Health Organization (WHO). Korea, South Global Youth Tobacco Survey 2005. United States: Centers for Disease Control and Prevention (CDC), 2005

Centers for Disease Control and Prevention (CDC) and World Health Organization (WHO). New Zealand Global Youth Tobacco Survey 2007. United States: Centers for Disease Control and Prevention (CDC), 2007

Centers for Disease Control and Prevention (CDC) and World Health Organization (WHO). Singapore Global Youth Tobacco Survey 2000. United States: Centers for Disease Control and Prevention (CDC), 2000

Centers for Disease Control and Prevention (CDC) and World Health Organization (WHO). Uruguay Global Youth Tobacco Survey 2007. Atlanta, United States: Centers for Disease Control and Prevention (CDC)

Centers for Disease Control and Prevention (CDC), Department of Hygiene and Epidemiology, University of Thessaly (Greece), MRB Hellas, National School of Public Health (Greece), World Health Organization (WHO). Greece Global Adult Tobacco Survey 2013

Centers for Disease Control and Prevention (CDC), Johns Hopkins Bloomberg School of Public Health, Latin American Center for Human Economy, Ministry of Public Health (Uruguay), National Institute of Statistics (Uruguay), Pan American Health Organization (PAHO), Research Triangle Institute, Inc. (RTI), World Health Organization (WHO). Uruguay Global Adult Tobacco Survey 2009

Centers for Disease Control and Prevention (CDC), Joint United Nations Program on HIV/AIDS (UNAIDS), Ministry of Health (Cook Islands), United Nations Children's Fund (UNICEF), United Nations Educational, Scientific and Cultural Organization (UNESCO), World Health Organization (WHO). Cook Islands Global School-Based Student Health Survey 2011. Geneva, Switzerland: World Health Organization (WHO)

Centers for Disease Control and Prevention (CDC), Ministry of Education (Argentina), Ministry of Health (Argentina), Pan American Health Organization (PAHO), Public Opinion, Services, and Markets (OPSM), World Health Organization (WHO). Argentina Global School-Based Student Health Survey 2007

Centers for Disease Control and Prevention (CDC), Ministry of Public Health (Uruguay), World Health Organization (WHO). Uruguay Global School-Based Student Health Survey 2006. Geneva, Switzerland: World Health Organization (WHO)

Centers for Disease Control and Prevention (CDC), Ministry of Public Health (Uruguay), World Health Organization (WHO). Uruguay Global School-Based Student Health Survey 2012

Centers for Disease Control and Prevention (CDC), Ministry of Social Development (Anguilla), World Health Organization (WHO). Anguilla Global School-Based Student Health Survey 2009

Centers for Disease Control and Prevention (CDC), World Health Organization (WHO). Guam Global Youth Tobacco Survey 2011. Atlanta, United States: Centers for Disease Control and Prevention (CDC), 2013

Centers for Disease Control and Prevention (CDC), World Health Organization (WHO). Italy Global Youth Tobacco Survey 2010. Atlanta, United States: Centers for Disease Control and Prevention (CDC), 2013

Centers for Disease Control and Prevention (CDC), World Health Organization (WHO). New Caledonia Global Youth Tobacco Survey 2010. Atlanta, United States: Centers for Disease Control and Prevention (CDC), 2013

Centers for Disease Control and Prevention (CDC), World Health Organization (WHO). New Zealand Global Youth Tobacco Survey 2008. Atlanta, United States: Centers for Disease Control and Prevention (CDC)

Centers for Disease Control and Prevention (CDC), World Health Organization (WHO). New Zealand Global Youth Tobacco Survey 2010. Atlanta, United States: Centers for Disease Control and Prevention (CDC), 2013

Centers for Disease Control and Prevention (CDC), World Health Organization (WHO). South Korea Global Youth Tobacco Survey 2008. Atlanta, United States: Centers for Disease Control and Prevention (CDC)

Centers for Disease Control and Prevention (CDC), World Health Organization (WHO). United States National Youth Tobacco Survey 2000. Atlanta, United States: Centers for Disease Control and Prevention (CDC)

Centers for Disease Control and Prevention (CDC). Tobacco use among adults -- United States, 2005. MMWR Morb Mortal Wkly Rep. 2006; 55(42): 1145-8

Centers for Disease Control and Prevention (CDC). United States Behavioral Risk Factor Surveillance System 1984. Atlanta, Georgia: CDC, US Department of Health and Human Services

Centers for Disease Control and Prevention (CDC). United States Behavioral Risk Factor Surveillance System 1985. Atlanta, Georgia: CDC, US Department of Health and Human Services

Centers for Disease Control and Prevention (CDC). United States Behavioral Risk Factor Surveillance System 1986. Atlanta, Georgia: CDC, US Department of Health and Human Services

Centers for Disease Control and Prevention (CDC). United States Behavioral Risk Factor Surveillance System 1987. Atlanta, Georgia: CDC, US Department of Health and Human Services

Centers for Disease Control and Prevention (CDC). United States Behavioral Risk Factor Surveillance System 1989. Atlanta, Georgia: CDC, US Department of Health and Human Services

Centers for Disease Control and Prevention (CDC). United States Behavioral Risk Factor Surveillance System 1990. Atlanta, Georgia: CDC, US Department of Health and Human Services

Centers for Disease Control and Prevention (CDC). United States Behavioral Risk Factor Surveillance System 1991. Atlanta, Georgia: CDC, US Department of Health and Human Services

Centers for Disease Control and Prevention (CDC). United States Behavioral Risk Factor Surveillance System 1992. Atlanta, Georgia: CDC, US Department of Health and Human Services

Centers for Disease Control and Prevention (CDC). United States Behavioral Risk Factor Surveillance System 1993. Atlanta, Georgia: CDC, US Department of Health and Human Services

Centers for Disease Control and Prevention (CDC). United States Behavioral Risk Factor Surveillance System 1994. Atlanta, Georgia: CDC, US Department of Health and Human Services

Centers for Disease Control and Prevention (CDC). United States Behavioral Risk Factor Surveillance System 1995. Atlanta, Georgia: CDC, US Department of Health and Human Services

Centers for Disease Control and Prevention (CDC). United States Behavioral Risk Factor Surveillance System 1996. Atlanta, Georgia: CDC, US Department of Health and Human Services

Centers for Disease Control and Prevention (CDC). United States Behavioral Risk Factor Surveillance System 1997. Atlanta, Georgia: CDC, US Department of Health and Human Services

Centers for Disease Control and Prevention (CDC). United States Behavioral Risk Factor Surveillance System 1998. Atlanta, Georgia: CDC, US Department of Health and Human Services

Centers for Disease Control and Prevention (CDC). United States Behavioral Risk Factor Surveillance System 1999. Atlanta, Georgia: CDC, US Department of Health and Human Services

Centers for Disease Control and Prevention (CDC). United States Behavioral Risk Factor Surveillance System 2000. Atlanta, Georgia: CDC, US Department of Health and Human Services

Centers for Disease Control and Prevention (CDC). United States Behavioral Risk Factor Surveillance System 2001. Atlanta, Georgia: CDC, US Department of Health and Human Services

Centers for Disease Control and Prevention (CDC). United States Behavioral Risk Factor Surveillance System 2002. Atlanta, Georgia: CDC, US Department of Health and Human Services

Centers for Disease Control and Prevention (CDC). United States Behavioral Risk Factor Surveillance System 2003. Atlanta, Georgia: CDC, US Department of Health and Human Services

Centers for Disease Control and Prevention (CDC). United States Behavioral Risk Factor Surveillance System 2004. Atlanta, Georgia: CDC, US Department of Health and Human Services

Centers for Disease Control and Prevention (CDC). United States Behavioral Risk Factor Surveillance System 2005. Atlanta, Georgia: CDC, US Department of Health and Human Services

Centers for Disease Control and Prevention (CDC). United States Behavioral Risk Factor Surveillance System 2006. Atlanta, Georgia: CDC, US Department of Health and Human Services

Centers for Disease Control and Prevention (CDC). United States Behavioral Risk Factor Surveillance System 2007. Atlanta, Georgia: CDC, US Department of Health and Human Services

Centers for Disease Control and Prevention (CDC). United States Behavioral Risk Factor Surveillance System 2008. Atlanta, Georgia: CDC, US Department of Health and Human Services

Centers for Disease Control and Prevention (CDC). United States Behavioral Risk Factor Surveillance System 2009. Atlanta, Georgia: CDC, US Department of Health and Human Services

Centers for Disease Control and Prevention (CDC). United States Behavioral Risk Factor Surveillance System 2010. Atlanta, United States: Centers for Disease Control and Prevention (CDC)

Centers for Disease Control and Prevention (CDC). United States Behavioral Risk Factor Surveillance System 2011. Atlanta, Georgia: CDC, US Department of Health and Human Services

Centers for Disease Control and Prevention (CDC). United States Behavioral Risk Factor Surveillance System 2012. Atlanta, Georgia: CDC, US Department of Health and Human Services, 2013

Centers for Disease Control and Prevention (CDC). United States Behavioral Risk Factor Surveillance System 2013. Atlanta, United States: Centers for Disease Control and Prevention (CDC), 2014

Centers for Disease Control and Prevention (CDC). United States Behavioral Risk Factor Surveillance System 2014. Atlanta, United States: Centers for Disease Control and Prevention (CDC), 2015

Centers for Disease Control and Prevention (CDC). United States Behavioral Risk Factor Surveillance System; 1988. Atlanta, Georgia: CDC, US Department of Health and Human Services

Centers for Disease Control and Prevention (CDC). United States National Youth Risk Behavior Survey 1991. Atlanta, United States: Centers for Disease Control and Prevention (CDC)

Centers for Disease Control and Prevention (CDC). United States National Youth Risk Behavior Survey 1993. Atlanta, United States: Centers for Disease Control and Prevention (CDC)

Centers for Disease Control and Prevention (CDC). United States National Youth Risk Behavior Survey 1995. Atlanta, United States: Centers for Disease Control and Prevention (CDC), 1996

Centers for Disease Control and Prevention (CDC). United States National Youth Risk Behavior Survey 1997. Atlanta, United States: Centers for Disease Control and Prevention (CDC), 1998

Centers for Disease Control and Prevention (CDC). United States National Youth Risk Behavior Survey 1999. Atlanta, United States: Centers for Disease Control and Prevention (CDC), 2000

Centers for Disease Control and Prevention (CDC). United States National Youth Risk Behavior Survey 2001. Atlanta, United States: Centers for Disease Control and Prevention (CDC), 2002

Centers for Disease Control and Prevention (CDC). United States National Youth Risk Behavior Survey 2003. Atlanta, United States: Centers for Disease Control and Prevention (CDC), 2004

Centers for Disease Control and Prevention (CDC). United States National Youth Risk Behavior Survey 2005. Atlanta, United States: Centers for Disease Control and Prevention (CDC), 2006

Centers for Disease Control and Prevention (CDC). United States National Youth Risk Behavior Survey 2007. Atlanta, United States: Centers for Disease Control and Prevention (CDC), 2008

Centers for Disease Control and Prevention (CDC). United States National Youth Risk Behavior Survey 2009. Atlanta, United States: Centers for Disease Control and Prevention (CDC), 2010

Centers for Disease Control and Prevention (CDC). United States National Youth Risk Behavior Survey 2011. Atlanta, United States: Centers for Disease Control and Prevention (CDC), 2012

Centers for Disease Control and Prevention (CDC). United States National Youth Tobacco Survey 1999. Atlanta, United States: Centers for Disease Control and Prevention (CDC)

Centers for Disease Control and Prevention (CDC). United States National Youth Tobacco Survey 2002. Atlanta, United States: Centers for Disease Control and Prevention (CDC)

Centers for Disease Control and Prevention (CDC). United States National Youth Tobacco Survey 2004. Atlanta, United States: Centers for Disease Control and Prevention (CDC)

Centers for Disease Control and Prevention (CDC). United States National Youth Tobacco Survey 2006. Atlanta, United States: Centers for Disease Control and Prevention (CDC)

Centers for Disease Control and Prevention (CDC). United States National Youth Tobacco Survey 2009. Atlanta, United States: Centers for Disease Control and Prevention (CDC)

Centers for Disease Control and Prevention (CDC). United States National Youth Tobacco Survey 2011. Atlanta, United States: Centers for Disease Control and Prevention (CDC)

Centers for Disease Control and Prevention (CDC). United States National Youth Tobacco Survey 2012. Atlanta, United States: Centers for Disease Control and Prevention (CDC)

Centers for Disease Control and Prevention (CDC). USA National Health Interview Survey 1997

Central Bureau of Statistics (Israel), Ministry of Health (Israel). Israel Health Survey 2009

Centre for Drug Research, University of Amsterdam. Netherlands Licit and Illicit Drug Use Survey 1997

Centre for Health Promotion Studies, National University of Ireland, Galway, Department of Public Health Medicine and Epidemiology, University College Dublin, Health Promotion Unit, Department of Health and Children (Ireland). Ireland Survey of Lifestyle Attitudes and Nutrition 2002. Dublin, Ireland: Health Promotion Unit, Department of Health and Children (Ireland)

Centre for Health Promotion Studies, National University of Ireland, Galway, Health Promotion Unit, Department of Health and Children (Ireland). Ireland Survey of Lifestyle Attitudes and Nutrition 1998. Dublin, Ireland: Health Promotion Unit, Department of Health and Children (Ireland)

Centre of Health Economics (Latvia), Lithuanian University of Health Sciences, National Institute for Health Development (Estonia), National Institute for Health and Welfare (Finland). Social Determinants of Health Behaviors Finbalt Health Monitor 1998-2008. Helsinki, Finland: National Institute for Health and Welfare (Finland), 2011

Coll M, Borrell C, Villabi J, Goicoechea J. Prévalence du tabagisme en Andorre: Données de référence pour l'évaluation des interventions. Rev Epidemiol Sante Publique. 2000; 305–8

Commission of the European Communities (2012): Eurobarometer 27 (Mar-May 1987). Faits et Opinions, Paris. GESIS Data Archive, Cologne. ZA1712 Data file Version 1.0.1, doi:10.4232/1.10884

Commission of the European Communities (2012): Eurobarometer 27 (Mar-May 1987). Faits et Opinions, Paris. GESIS Data Archive, Cologne. ZA1712 Data file Version 1.0.1, doi:10.4232/1.10884

Commission of the European Communities (2012): Eurobarometer 27 (Mar-May 1987). Faits et Opinions, Paris. GESIS Data Archive, Cologne. ZA1712 Data file Version 1.0.1, doi:10.4232/1.10884

Commission of the European Communities (2012): Eurobarometer 27 (Mar-May 1987). Faits et Opinions, Paris. GESIS Data Archive, Cologne. ZA1712 Data file Version 1.0.1, doi:10.4232/1.10884

Commission of the European Communities (2012): Eurobarometer 27 (Mar-May 1987). Faits et Opinions, Paris. GESIS Data Archive, Cologne. ZA1712 Data file Version 1.0.1, doi:10.4232/1.10884

Commission of the European Communities (2012): Eurobarometer 27 (Mar-May 1987). Faits et Opinions, Paris. GESIS Data Archive, Cologne. ZA1712 Data file Version 1.0.1, doi:10.4232/1.10884

Commission of the European Communities (2012): Eurobarometer 27 (Mar-May 1987). Faits et Opinions, Paris. GESIS Data Archive, Cologne. ZA1712 Data file Version 1.0.1, doi:10.4232/1.10884

Commission of the European Communities (2012): Eurobarometer 27 (Mar-May 1987). Faits et Opinions, Paris. GESIS Data Archive, Cologne. ZA1712 Data file Version 1.0.1, doi:10.4232/1.10884

Commission of the European Communities (2012): Eurobarometer 27 (Mar-May 1987). Faits et Opinions, Paris. GESIS Data Archive, Cologne. ZA1712 Data file Version 1.0.1, doi:10.4232/1.10884

Commission of the European Communities (2012): Eurobarometer 27 (Mar-May 1987). Faits et Opinions, Paris. GESIS Data Archive, Cologne. ZA1712 Data file Version 1.0.1, doi:10.4232/1.10884

Commission of the European Communities (2012): Eurobarometer 27 (Mar-May 1987). Faits et Opinions, Paris. GESIS Data Archive, Cologne. ZA1712 Data file Version 1.0.1, doi:10.4232/1.10884

Commission of the European Communities (2012): Eurobarometer 29 (Mar-Apr 1988). Faits et Opinions, Paris. GESIS Data Archive, Cologne. ZA1714 Data file Version 1.0.1, doi:10.4232/1.10886

Commission of the European Communities (2012): Eurobarometer 29 (Mar-Apr 1988). Faits et Opinions, Paris. GESIS Data Archive, Cologne. ZA1714 Data file Version 1.0.1, doi:10.4232/1.10886

Commission of the European Communities (2012): Eurobarometer 29 (Mar-Apr 1988). Faits et Opinions, Paris. GESIS Data Archive, Cologne. ZA1714 Data file Version 1.0.1, doi:10.4232/1.10886

Commission of the European Communities (2012): Eurobarometer 29 (Mar-Apr 1988). Faits et Opinions, Paris. GESIS Data Archive, Cologne. ZA1714 Data file Version 1.0.1, doi:10.4232/1.10886

Commission of the European Communities (2012): Eurobarometer 29 (Mar-Apr 1988). Faits et Opinions, Paris. GESIS Data Archive, Cologne. ZA1714 Data file Version 1.0.1, doi:10.4232/1.10886

Commission of the European Communities (2012): Eurobarometer 29 (Mar-Apr 1988). Faits et Opinions, Paris. GESIS Data Archive, Cologne. ZA1714 Data file Version 1.0.1, doi:10.4232/1.10886

Commission of the European Communities (2012): Eurobarometer 29 (Mar-Apr 1988). Faits et Opinions, Paris. GESIS Data Archive, Cologne. ZA1714 Data file Version 1.0.1, doi:10.4232/1.10886

Commission of the European Communities (2012): Eurobarometer 29 (Mar-Apr 1988). Faits et Opinions, Paris. GESIS Data Archive, Cologne. ZA1714 Data file Version 1.0.1, doi:10.4232/1.10886

Commission of the European Communities (2012): Eurobarometer 29 (Mar-Apr 1988). Faits et Opinions, Paris. GESIS Data Archive, Cologne. ZA1714 Data file Version 1.0.1, doi:10.4232/1.10886

Commission of the European Communities (2012): Eurobarometer 29 (Mar-Apr 1988). Faits et Opinions, Paris. GESIS Data Archive, Cologne. ZA1714 Data file Version 1.0.1, doi:10.4232/1.10886

Commission of the European Communities (2012): Eurobarometer 29 (Mar-Apr 1988). Faits et Opinions, Paris. GESIS Data Archive, Cologne. ZA1714 Data file Version 1.0.1, doi:10.4232/1.10886

Commission of the European Communities (2012): Eurobarometer 29 (Mar-Apr 1988). Faits et Opinions, Paris. GESIS Data Archive, Cologne. ZA1714 Data file Version 1.0.1, doi:10.4232/1.10886

Commission of the European Communities (2012): Eurobarometer 31A (Jun-Jul 1989). Faits et Opinions, Paris. GESIS Data Archive, Cologne. ZA1751 Data file Version 1.0.1, doi:10.4232/1.10889

Commission of the European Communities (2012): Eurobarometer 31A (Jun-Jul 1989). Faits et Opinions, Paris. GESIS Data Archive, Cologne. ZA1751 Data file Version 1.0.1, doi:10.4232/1.10889

Commission of the European Communities (2012): Eurobarometer 31A (Jun-Jul 1989). Faits et Opinions, Paris. GESIS Data Archive, Cologne. ZA1751 Data file Version 1.0.1, doi:10.4232/1.10889

Commission of the European Communities (2012): Eurobarometer 31A (Jun-Jul 1989). Faits et Opinions, Paris. GESIS Data Archive, Cologne. ZA1751 Data file Version 1.0.1, doi:10.4232/1.10889

Commission of the European Communities (2012): Eurobarometer 31A (Jun-Jul 1989). Faits et Opinions, Paris. GESIS Data Archive, Cologne. ZA1751 Data file Version 1.0.1, doi:10.4232/1.10889

Commission of the European Communities (2012): Eurobarometer 31A (Jun-Jul 1989). Faits et Opinions, Paris. GESIS Data Archive, Cologne. ZA1751 Data file Version 1.0.1, doi:10.4232/1.10889

Commission of the European Communities (2012): Eurobarometer 31A (Jun-Jul 1989). Faits et Opinions, Paris. GESIS Data Archive, Cologne. ZA1751 Data file Version 1.0.1, doi:10.4232/1.10889

Commission of the European Communities (2012): Eurobarometer 31A (Jun-Jul 1989). Faits et Opinions, Paris. GESIS Data Archive, Cologne. ZA1751 Data file Version 1.0.1, doi:10.4232/1.10889

Commission of the European Communities (2012): Eurobarometer 31A (Jun-Jul 1989). Faits et Opinions, Paris. GESIS Data Archive, Cologne. ZA1751 Data file Version 1.0.1, doi:10.4232/1.10889

Commission of the European Communities (2012): Eurobarometer 31A (Jun-Jul 1989). Faits et Opinions, Paris. GESIS Data Archive, Cologne. ZA1751 Data file Version 1.0.1, doi:10.4232/1.10889

Commission of the European Communities (2012): Eurobarometer 32 (Oct-Nov 1989). INRA, Brussels. GESIS Data Archive, Cologne. ZA1752 Data file Version 1.1.0, doi:10.4232/1.10890

Commission of the European Communities (2012): Eurobarometer 32 (Oct-Nov 1989). INRA, Brussels. GESIS Data Archive, Cologne. ZA1752 Data file Version 1.1.0, doi:10.4232/1.10890

Commission of the European Communities (2012): Eurobarometer 32 (Oct-Nov 1989). INRA, Brussels. GESIS Data Archive, Cologne. ZA1752 Data file Version 1.1.0, doi:10.4232/1.10890

Commission of the European Communities (2012): Eurobarometer 32 (Oct-Nov 1989). INRA, Brussels. GESIS Data Archive, Cologne. ZA1752 Data file Version 1.1.0, doi:10.4232/1.10890

Commission of the European Communities (2012): Eurobarometer 34.1 (Nov 1990). INRA, Brussels. GESIS Data Archive, Cologne. ZA1961 Data file Version 1.0.1, doi:10.4232/1.10893

Commission of the European Communities (2012): Eurobarometer 34.1 (Nov 1990). INRA, Brussels. GESIS Data Archive, Cologne. ZA1961 Data file Version 1.0.1, doi:10.4232/1.10893

Commission of the European Communities (2012): Eurobarometer 34.1 (Nov 1990). INRA, Brussels. GESIS Data Archive, Cologne. ZA1961 Data file Version 1.0.1, doi:10.4232/1.10893

Commission of the European Communities (2012): Eurobarometer 34.1 (Nov 1990). INRA, Brussels. GESIS Data Archive, Cologne. ZA1961 Data file Version 1.0.1, doi:10.4232/1.10893

Commission of the European Communities (2012): Eurobarometer 34.1 (Nov 1990). INRA, Brussels. GESIS Data Archive, Cologne. ZA1961 Data file Version 1.0.1, doi:10.4232/1.10893

Commission of the European Communities (2012): Eurobarometer 34.1 (Nov 1990). INRA, Brussels. GESIS Data Archive, Cologne. ZA1961 Data file Version 1.0.1, doi:10.4232/1.10893

Commission of the European Communities (2012): Eurobarometer 34.1 (Nov 1990). INRA, Brussels. GESIS Data Archive, Cologne. ZA1961 Data file Version 1.0.1, doi:10.4232/1.10893

Commission of the European Communities (2012): Eurobarometer 34.1 (Nov 1990). INRA, Brussels. GESIS Data Archive, Cologne. ZA1961 Data file Version 1.0.1, doi:10.4232/1.10893

Commission of the European Communities (2012): Eurobarometer 34.1 (Nov 1990). INRA, Brussels. GESIS Data Archive, Cologne. ZA1961 Data file Version 1.0.1, doi:10.4232/1.10893

Commission of the European Communities (2012): Eurobarometer 34.1 (Nov 1990). INRA, Brussels. GESIS Data Archive, Cologne. ZA1961 Data file Version 1.0.1, doi:10.4232/1.10893

Commission of the European Communities (2012): Eurobarometer 34.1 (Nov 1990). INRA, Brussels. GESIS Data Archive, Cologne. ZA1961 Data file Version 1.0.1, doi:10.4232/1.10893

Commission of the European Communities (2012): Eurobarometer 36 (Oct-Nov 1991). INRA, Brussels. GESIS Data Archive, Cologne. ZA2081 Data file Version 1.1.0, doi:10.4232/1.10848

Commission of the European Communities (2012): Eurobarometer 36 (Oct-Nov 1991). INRA, Brussels. GESIS Data Archive, Cologne. ZA2081 Data file Version 1.1.0, doi:10.4232/1.10848

Commission of the European Communities (2012): Eurobarometer 36 (Oct-Nov 1991). INRA, Brussels. GESIS Data Archive, Cologne. ZA2081 Data file Version 1.1.0, doi:10.4232/1.10848

Commission of the European Communities (2012): Eurobarometer 36 (Oct-Nov 1991). INRA, Brussels. GESIS Data Archive, Cologne. ZA2081 Data file Version 1.1.0, doi:10.4232/1.10848

Commission of the European Communities (2012): Eurobarometer 36 (Oct-Nov 1991). INRA, Brussels. GESIS Data Archive, Cologne. ZA2081 Data file Version 1.1.0, doi:10.4232/1.10848

Commission of the European Communities (2012): Eurobarometer 36 (Oct-Nov 1991). INRA, Brussels. GESIS Data Archive, Cologne. ZA2081 Data file Version 1.1.0, doi:10.4232/1.10848

Commission of the European Communities (2012): Eurobarometer 36 (Oct-Nov 1991). INRA, Brussels. GESIS Data Archive, Cologne. ZA2081 Data file Version 1.1.0, doi:10.4232/1.10848

Commission of the European Communities (2012): Eurobarometer 36 (Oct-Nov 1991). INRA, Brussels. GESIS Data Archive, Cologne. ZA2081 Data file Version 1.1.0, doi:10.4232/1.10848

Commission of the European Communities (2012): Eurobarometer 36 (Oct-Nov 1991). INRA, Brussels. GESIS Data Archive, Cologne. ZA2081 Data file Version 1.1.0, doi:10.4232/1.10848

Commission of the European Communities (2012): Eurobarometer 36 (Oct-Nov 1991). INRA, Brussels. GESIS Data Archive, Cologne. ZA2081 Data file Version 1.1.0, doi:10.4232/1.10848

Commission of the European Communities (2012): Eurobarometer 36 (Oct-Nov 1991). INRA, Brussels. GESIS Data Archive, Cologne. ZA2081 Data file Version 1.1.0, doi:10.4232/1.10848

Commission of the European Communities (2012): Eurobarometer 36 (Oct-Nov 1991). INRA, Brussels. GESIS Data Archive, Cologne. ZA2081 Data file Version 1.1.0, doi:10.4232/1.10848

Commission of the European Communities (2012): Eurobarometer 36 (Oct-Nov 1991). INRA, Brussels. GESIS Data Archive, Cologne. ZA2081 Data file Version 1.1.0, doi:10.4232/1.10848

Commission of the European Communities (2012): Eurobarometer 38.0 (Sep-Oct 1992). INRA, Brussels. GESIS Data Archive, Cologne. ZA2294 Data file Version 1.1.0, doi:10.4232/1.10903

Commission of the European Communities (2012): Eurobarometer 38.0 (Sep-Oct 1992). INRA, Brussels. GESIS Data Archive, Cologne. ZA2294 Data file Version 1.1.0, doi:10.4232/1.10903

Commission of the European Communities (2012): Eurobarometer 38.0 (Sep-Oct 1992). INRA, Brussels. GESIS Data Archive, Cologne. ZA2294 Data file Version 1.1.0, doi:10.4232/1.10903

Commission of the European Communities (2012): Eurobarometer 38.0 (Sep-Oct 1992). INRA, Brussels. GESIS Data Archive, Cologne. ZA2294 Data file Version 1.1.0, doi:10.4232/1.10903

Commission of the European Communities (2012): Eurobarometer 38.0 (Sep-Oct 1992). INRA, Brussels. GESIS Data Archive, Cologne. ZA2294 Data file Version 1.1.0, doi:10.4232/1.10903

Commission of the European Communities (2012): Eurobarometer 38.0 (Sep-Oct 1992). INRA, Brussels. GESIS Data Archive, Cologne. ZA2294 Data file Version 1.1.0, doi:10.4232/1.10903

Cox, B.D., Health and Lifestyle Survey, 1984-1985 [computer file]. Colchester, Essex: UK Data Archive [distributor], October 1988. SN: 2218, http://dx.doi.org/10.5255/UKDA-SN-2218-1

Cox, B.D., Health and Lifestyle Survey: Seven-Year Follow-Up, 1991-1992 [computer file]. Colchester, Essex: UK Data Archive [distributor], January 1995. SN: 3279 , http://dx.doi.org/10.5255/UKDA-SN-3279-1

Danish Cancer Society, Danish Health and Medicines Authority, Danish Heart Foundation, Danish Lung Association, Ramboll. Denmark Monitoring Smoking Habits in the Danish Population 2003

Danish Cancer Society, Danish Health and Medicines Authority, Danish Heart Foundation, Danish Lung Association, Ramboll. Denmark Monitoring Smoking Habits in the Danish Population 2004

Danish Cancer Society, Danish Health and Medicines Authority, Danish Heart Foundation, Danish Lung Association, Ramboll. Denmark Monitoring Smoking Habits in the Danish Population 2005

Danish Cancer Society, Danish Health and Medicines Authority, Danish Heart Foundation, Danish Lung Association, Ramboll. Denmark Monitoring Smoking Habits in the Danish Population 2006

Danish Cancer Society, Danish Health and Medicines Authority, Danish Heart Foundation, Danish Lung Association, TNS Gallup. Denmark Monitoring Smoking Habits in the Danish Population 2007

Danish Cancer Society, Danish Health and Medicines Authority, Danish Heart Foundation, Danish Lung Association, TNS Gallup. Denmark Monitoring Smoking Habits in the Danish Population 2008

Danish Cancer Society, Danish Health and Medicines Authority, Danish Heart Foundation, Danish Lung Association, TNS Gallup. Denmark Monitoring Smoking Habits in the Danish Population 2009

Danish Cancer Society, Danish Health and Medicines Authority, Danish Heart Foundation, Danish Lung Association, TNS Gallup. Denmark Monitoring Smoking Habits in the Danish Population 2010

Danish Cancer Society, Danish Health and Medicines Authority, Danish Heart Foundation, Danish Lung Association, TNS Gallup. Denmark Monitoring Smoking Habits in the Danish Population 2011

Danish Cancer Society, Danish Health and Medicines Authority, Danish Heart Foundation, Danish Lung Association, TNS Gallup. Denmark Monitoring Smoking Habits in the Danish Population 2012

Danish Cancer Society, Danish Health and Medicines Authority, Danish Heart Foundation, Danish Lung Association, TNS Gallup. Denmark Monitoring Smoking Habits in the Danish Population 2013

Danish Health and Medicines Authority. Denmark Alcohol, Drugs and Tobacco Statistics 2003. Copenhagen, Denmark: Danish Health and Medicines Authority, 2004

Denmark Eurobarometer 32: The Single European Market, Drugs, Alcohol, and Cancer 1989 as it appears in P.N. Lee Statistics and Computing Ltd. International Mortality and Smoking Statistics Version 4.04. Sutton, United Kingdom: P.N. Lee Statistics and Computing Ltd, 2009

Denmark Health Interview Survey 1991 as it appears in P.N. Lee Statistics and Computing Ltd. International Mortality and Smoking Statistics Version 4.04. Sutton, United Kingdom: P.N. Lee Statistics and Computing Ltd, 2009

Denmark Health Interview Survey 1998 as it appears in P.N. Lee Statistics and Computing Ltd. International Mortality and Smoking Statistics Version 4.04. Sutton, United Kingdom: P.N. Lee Statistics and Computing Ltd, 2009

Denmark Health Interview Survey 1999 as it appears in P.N. Lee Statistics and Computing Ltd. International Mortality and Smoking Statistics Version 4.04. Sutton, United Kingdom: P.N. Lee Statistics and Computing Ltd, 2009

Denmark Health Interview Survey 2001 as it appears in P.N. Lee Statistics and Computing Ltd. International Mortality and Smoking Statistics Version 4.04. Sutton, United Kingdom: P.N. Lee Statistics and Computing Ltd, 2009

Denmark Longitudinal Health Behavior Survey 1990 as it appears in P.N. Lee Statistics and Computing Ltd. International Mortality and Smoking Statistics Version 4.04. Sutton, United Kingdom: P.N. Lee Statistics and Computing Ltd, 2009

Department of Economic Planning and Development (Brunei Darussalam). Brunei Population and Housing Census 2001

Department of Economics, University of Chile, Ministry of Planning (Chile). Chile National Socioeconomic Characterization Survey 1990

Department of Economics, University of Chile, Ministry of Planning (Chile). Chile National Socioeconomic Characterization Survey 1992

Department of Economics, University of Chile, Ministry of Planning (Chile). Chile National Socioeconomic Characterization Survey 1998

Department of Health (Ireland), Economic and Social Research Institute (ESRI) (Ireland). Ireland Smoking and Drinking Among Young People 1993

Department of Health (United Kingdom), Office for National Statistics (United Kingdom). United Kingdom General Household Survey 1988-1989 - UK Data Service

Department of Health (United Kingdom), Office for National Statistics (United Kingdom). United Kingdom General Household Survey 2000-2001 - ONS

Department of Health Information, Ministry for Health (Malta). Malta National Health Interview Survey 2002

Department of Public Health, Pontifical Catholic University of Chile, Institute of Nutrition and Food Technology (INTA), University of Chile, Ministry of Health (Chile), Pan American Health Organization (PAHO). Chile National Health Survey 2003

Dias CM, Graca MJ. National Health Survey in Portugal: History, Methods and Some Results. Lisbon, Portugal: National Health Institute Doutor Ricardo Jorge (INSA), 2000

Directorate for Health and Social Affairs (Norway), Norwegian Institute for Alcohol and Drug Research (SIRUS). Norwegian Tobacco Statistics 1973-2006. Oslo, Norway: Norwegian Institute for Alcohol and Drug Research (SIRUS), 2007

DOXA, Italian League for the Fight Against Cancer (LILT), Mario Negri Institute for Pharmacological Research, National Institute of Health (Italy). Italy Tobacco Use Survey 2007

DOXA, Italian League for the Fight Against Cancer (LILT), Mario Negri Institute for Pharmacological Research, National Institute of Health (Italy). Italy Tobacco Use Survey 2008

DOXA, Italian League for the Fight Against Cancer (LILT), Mario Negri Institute for Pharmacological Research, National Institute of Health (Italy). Italy Tobacco Use Survey 2009

DOXA, Italian League for the Fight Against Cancer (LILT), Mario Negri Institute for Pharmacological Research, National Institute of Health (Italy). Italy Tobacco Use Survey 2010

DOXA, Italian League for the Fight Against Cancer (LILT), National Institute of Health (Italy). Italy Tobacco Use Survey 2006

Economic and Social Research Institute (ESRI) (Ireland), Health Promotion Unit, Department of Health and Children (Ireland), National University of Ireland, Galway, Royal College of Surgeons in Ireland (RCSI), University College Cork. Ireland Survey of Lifestyle Attitudes and Nutrition 2007. Dublin, Ireland: Health Promotion Unit, Department of Health and Children (Ireland)

ESPAD Report 1995: Alcohol and Other Drug Use Among Students in 26 European Countries as it appears in P.N. Lee Statistics and Computing Ltd. International Mortality and Smoking Statistics Version 4.04. Sutton, United Kingdom: P.N. Lee Statistics and Computing Ltd, 2009

ESPAD Report 1999: Alcohol and Other Drug Use Among Students in 30 European Countries as it appears in P.N. Lee Statistics and Computing Ltd. International Mortality and Smoking Statistics Version 4.04. Sutton, United Kingdom: P.N. Lee Statistics and Computing Ltd, 2009

European Commission (2012): Eurobarometer 41.0 (Mar-May 1994). INRA, Brussels. GESIS Data Archive, Cologne. ZA2490 Data file Version 1.1.0, doi:10.4232/1.10909

European Commission (2012): Eurobarometer 41.0 (Mar-May 1994). INRA, Brussels. GESIS Data Archive, Cologne. ZA2490 Data file Version 1.1.0, doi:10.4232/1.10909

European Commission (2012): Eurobarometer 41.0 (Mar-May 1994). INRA, Brussels. GESIS Data Archive, Cologne. ZA2490 Data file Version 1.1.0, doi:10.4232/1.10909

European Commission (2012): Eurobarometer 41.0 (Mar-May 1994). INRA, Brussels. GESIS Data Archive, Cologne. ZA2490 Data file Version 1.1.0, doi:10.4232/1.10909

European Commission (2012): Eurobarometer 41.0 (Mar-May 1994). INRA, Brussels. GESIS Data Archive, Cologne. ZA2490 Data file Version 1.1.0, doi:10.4232/1.10909

European Commission (2012): Eurobarometer 41.0 (Mar-May 1994). INRA, Brussels. GESIS Data Archive, Cologne. ZA2490 Data file Version 1.1.0, doi:10.4232/1.10909

European Commission (2012): Eurobarometer 41.0 (Mar-May 1994). INRA, Brussels. GESIS Data Archive, Cologne. ZA2490 Data file Version 1.1.0, doi:10.4232/1.10909

European Commission (2012): Eurobarometer 41.0 (Mar-May 1994). INRA, Brussels. GESIS Data Archive, Cologne. ZA2490 Data file Version 1.1.0, doi:10.4232/1.10909

European Commission (2012): Eurobarometer 41.0 (Mar-May 1994). INRA, Brussels. GESIS Data Archive, Cologne. ZA2490 Data file Version 1.1.0, doi:10.4232/1.10909

European Commission (2012): Eurobarometer 41.0 (Mar-May 1994). INRA, Brussels. GESIS Data Archive, Cologne. ZA2490 Data file Version 1.1.0, doi:10.4232/1.10909

European Commission (2012): Eurobarometer 41.0 (Mar-May 1994). INRA, Brussels. GESIS Data Archive, Cologne. ZA2490 Data file Version 1.1.0, doi:10.4232/1.10909

European Commission (2012): Eurobarometer 41.0 (Mar-May 1994). INRA, Brussels. GESIS Data Archive, Cologne. ZA2490 Data file Version 1.1.0, doi:10.4232/1.10909

European Commission (2012): Eurobarometer 43.0 (Mar-Apr 1995). INRA, Brussels. GESIS Data Archive, Cologne. ZA2636 Data file Version 1.0.1, doi:10.4232/1.10912

European Commission (2012): Eurobarometer 43.0 (Mar-Apr 1995). INRA, Brussels. GESIS Data Archive, Cologne. ZA2636 Data file Version 1.0.1, doi:10.4232/1.10912

European Commission (2012): Eurobarometer 43.0 (Mar-Apr 1995). INRA, Brussels. GESIS Data Archive, Cologne. ZA2636 Data file Version 1.0.1, doi:10.4232/1.10912

European Commission (2012): Eurobarometer 43.0 (Mar-Apr 1995). INRA, Brussels. GESIS Data Archive, Cologne. ZA2636 Data file Version 1.0.1, doi:10.4232/1.10912

European Commission (2012): Eurobarometer 43.0 (Mar-Apr 1995). INRA, Brussels. GESIS Data Archive, Cologne. ZA2636 Data file Version 1.0.1, doi:10.4232/1.10912

European Commission (2012): Eurobarometer 43.0 (Mar-Apr 1995). INRA, Brussels. GESIS Data Archive, Cologne. ZA2636 Data file Version 1.0.1, doi:10.4232/1.10912

European Commission (2012): Eurobarometer 43.0 (Mar-Apr 1995). INRA, Brussels. GESIS Data Archive, Cologne. ZA2636 Data file Version 1.0.1, doi:10.4232/1.10912

European Commission (2012): Eurobarometer 43.0 (Mar-Apr 1995). INRA, Brussels. GESIS Data Archive, Cologne. ZA2636 Data file Version 1.0.1, doi:10.4232/1.10912

European Commission (2012): Eurobarometer 43.0 (Mar-Apr 1995). INRA, Brussels. GESIS Data Archive, Cologne. ZA2636 Data file Version 1.0.1, doi:10.4232/1.10912

European Commission (2012): Eurobarometer 43.0 (Mar-Apr 1995). INRA, Brussels. GESIS Data Archive, Cologne. ZA2636 Data file Version 1.0.1, doi:10.4232/1.10912

European Commission (2012): Eurobarometer 43.0 (Mar-Apr 1995). INRA, Brussels. GESIS Data Archive, Cologne. ZA2636 Data file Version 1.0.1, doi:10.4232/1.10912

European Commission (2012): Eurobarometer 43.0 (Mar-Apr 1995). INRA, Brussels. GESIS Data Archive, Cologne. ZA2636 Data file Version 1.0.1, doi:10.4232/1.10912

European Commission (2012): Eurobarometer 43.0 (Mar-Apr 1995). INRA, Brussels. GESIS Data Archive, Cologne. ZA2636 Data file Version 1.0.1, doi:10.4232/1.10912

European Commission (2012): Eurobarometer 43.0 (Mar-Apr 1995). INRA, Brussels. GESIS Data Archive, Cologne. ZA2636 Data file Version 1.0.1, doi:10.4232/1.10912

European Commission (2012): Eurobarometer 43.0 (Mar-Apr 1995). INRA, Brussels. GESIS Data Archive, Cologne. ZA2636 Data file Version 1.0.1, doi:10.4232/1.10912

European Commission (2012): Eurobarometer 58.2 (Oct-Dec 2002). European Opinion Research Group (EORG), Brussels. GESIS Data Archive, Cologne. ZA3886 Data file Version 1.0.1, doi:10.4232/1.10954

European Commission (2012): Eurobarometer 58.2 (Oct-Dec 2002). European Opinion Research Group (EORG), Brussels. GESIS Data Archive, Cologne. ZA3886 Data file Version 1.0.1, doi:10.4232/1.10954

European Commission (2012): Eurobarometer 58.2 (Oct-Dec 2002). European Opinion Research Group (EORG), Brussels. GESIS Data Archive, Cologne. ZA3886 Data file Version 1.0.1, doi:10.4232/1.10954

European Commission (2012): Eurobarometer 58.2 (Oct-Dec 2002). European Opinion Research Group (EORG), Brussels. GESIS Data Archive, Cologne. ZA3886 Data file Version 1.0.1, doi:10.4232/1.10954

European Commission (2012): Eurobarometer 58.2 (Oct-Dec 2002). European Opinion Research Group (EORG), Brussels. GESIS Data Archive, Cologne. ZA3886 Data file Version 1.0.1, doi:10.4232/1.10954

European Commission (2012): Eurobarometer 58.2 (Oct-Dec 2002). European Opinion Research Group (EORG), Brussels. GESIS Data Archive, Cologne. ZA3886 Data file Version 1.0.1, doi:10.4232/1.10954

European Commission (2012): Eurobarometer 58.2 (Oct-Dec 2002). European Opinion Research Group (EORG), Brussels. GESIS Data Archive, Cologne. ZA3886 Data file Version 1.0.1, doi:10.4232/1.10954

European Commission (2012): Eurobarometer 58.2 (Oct-Dec 2002). European Opinion Research Group (EORG), Brussels. GESIS Data Archive, Cologne. ZA3886 Data file Version 1.0.1, doi:10.4232/1.10954

European Commission (2012): Eurobarometer 58.2 (Oct-Dec 2002). European Opinion Research Group (EORG), Brussels. GESIS Data Archive, Cologne. ZA3886 Data file Version 1.0.1, doi:10.4232/1.10954

European Commission (2012): Eurobarometer 58.2 (Oct-Dec 2002). European Opinion Research Group (EORG), Brussels. GESIS Data Archive, Cologne. ZA3886 Data file Version 1.0.1, doi:10.4232/1.10954

European Commission (2012): Eurobarometer 58.2 (Oct-Dec 2002). European Opinion Research Group (EORG), Brussels. GESIS Data Archive, Cologne. ZA3886 Data file Version 1.0.1, doi:10.4232/1.10954

European Commission (2012): Eurobarometer 58.2 (Oct-Dec 2002). European Opinion Research Group (EORG), Brussels. GESIS Data Archive, Cologne. ZA3886 Data file Version 1.0.1, doi:10.4232/1.10954

European Commission (2012): Eurobarometer 58.2 (Oct-Dec 2002). European Opinion Research Group (EORG), Brussels. GESIS Data Archive, Cologne. ZA3886 Data file Version 1.0.1, doi:10.4232/1.10954

European Commission (2012): Eurobarometer 58.2 (Oct-Dec 2002). European Opinion Research Group (EORG), Brussels. GESIS Data Archive, Cologne. ZA3886 Data file Version 1.0.1, doi:10.4232/1.10954

European Commission (2012): Eurobarometer 58.2 (Oct-Dec 2002). European Opinion Research Group (EORG), Brussels. GESIS Data Archive, Cologne. ZA3886 Data file Version 1.0.1, doi:10.4232/1.10954

European Commission (2012): Eurobarometer 64.1 (Sep-Oct 2005). TNS OPINION & SOCIAL, Brussels [Producer]. GESIS Data Archive, Cologne. ZA4413 Data file Version 1.1.0, doi:10.4232/1.10969

European Commission (2012): Eurobarometer 64.3 (Nov-Dec 2005). TNS OPINION & SOCIAL, Brussels [Producer]. GESIS Data Archive, Cologne. ZA4415 Data file Version 1.0.1, doi:10.4232/1.10971

European Commission (2012): Eurobarometer 64.3 (Nov-Dec 2005). TNS OPINION & SOCIAL, Brussels [Producer]. GESIS Data Archive, Cologne. ZA4415 Data file Version 1.0.1, doi:10.4232/1.10971

European Commission (2012): Eurobarometer 64.3 (Nov-Dec 2005). TNS OPINION & SOCIAL, Brussels [Producer]. GESIS Data Archive, Cologne. ZA4415 Data file Version 1.0.1, doi:10.4232/1.10971

European Commission (2012): Eurobarometer 64.3 (Nov-Dec 2005). TNS OPINION & SOCIAL, Brussels [Producer]. GESIS Data Archive, Cologne. ZA4415 Data file Version 1.0.1, doi:10.4232/1.10971

European Commission (2012): Eurobarometer 64.3 (Nov-Dec 2005). TNS OPINION & SOCIAL, Brussels [Producer]. GESIS Data Archive, Cologne. ZA4415 Data file Version 1.0.1, doi:10.4232/1.10971

European Commission (2012): Eurobarometer 64.3 (Nov-Dec 2005). TNS OPINION & SOCIAL, Brussels [Producer]. GESIS Data Archive, Cologne. ZA4415 Data file Version 1.0.1, doi:10.4232/1.10971

European Commission (2012): Eurobarometer 64.3 (Nov-Dec 2005). TNS OPINION & SOCIAL, Brussels [Producer]. GESIS Data Archive, Cologne. ZA4415 Data file Version 1.0.1, doi:10.4232/1.10971

European Commission (2012): Eurobarometer 64.3 (Nov-Dec 2005). TNS OPINION & SOCIAL, Brussels [Producer]. GESIS Data Archive, Cologne. ZA4415 Data file Version 1.0.1, doi:10.4232/1.10971

European Commission (2012): Eurobarometer 64.3 (Nov-Dec 2005). TNS OPINION & SOCIAL, Brussels [Producer]. GESIS Data Archive, Cologne. ZA4415 Data file Version 1.0.1, doi:10.4232/1.10971

European Commission (2012): Eurobarometer 64.3 (Nov-Dec 2005). TNS OPINION & SOCIAL, Brussels [Producer]. GESIS Data Archive, Cologne. ZA4415 Data file Version 1.0.1, doi:10.4232/1.10971

European Commission (2012): Eurobarometer 64.3 (Nov-Dec 2005). TNS OPINION & SOCIAL, Brussels [Producer]. GESIS Data Archive, Cologne. ZA4415 Data file Version 1.0.1, doi:10.4232/1.10971

European Commission (2012): Eurobarometer 64.3 (Nov-Dec 2005). TNS OPINION & SOCIAL, Brussels [Producer]. GESIS Data Archive, Cologne. ZA4415 Data file Version 1.0.1, doi:10.4232/1.10971

European Commission (2012): Eurobarometer 64.3 (Nov-Dec 2005). TNS OPINION & SOCIAL, Brussels [Producer]. GESIS Data Archive, Cologne. ZA4415 Data file Version 1.0.1, doi:10.4232/1.10971

European Commission (2012): Eurobarometer 64.3 (Nov-Dec 2005). TNS OPINION & SOCIAL, Brussels [Producer]. GESIS Data Archive, Cologne. ZA4415 Data file Version 1.0.1, doi:10.4232/1.10971

European Commission (2012): Eurobarometer 64.3 (Nov-Dec 2005). TNS OPINION & SOCIAL, Brussels [Producer]. GESIS Data Archive, Cologne. ZA4415 Data file Version 1.0.1, doi:10.4232/1.10971

European Commission (2012): Eurobarometer 64.3 (Nov-Dec 2005). TNS OPINION & SOCIAL, Brussels [Producer]. GESIS Data Archive, Cologne. ZA4415 Data file Version 1.0.1, doi:10.4232/1.10971

European Commission (2012): Eurobarometer 64.3 (Nov-Dec 2005). TNS OPINION & SOCIAL, Brussels [Producer]. GESIS Data Archive, Cologne. ZA4415 Data file Version 1.0.1, doi:10.4232/1.10971

European Commission (2012): Eurobarometer 66.2 (Oct-Nov 2006). TNS OPINION & SOCIAL, Brussels [Producer]. GESIS Data Archive, Cologne. ZA4527 Data file Version 1.0.1, doi:10.4232/1.10981

European Commission (2012): Eurobarometer 66.2 (Oct-Nov 2006). TNS OPINION & SOCIAL, Brussels [Producer]. GESIS Data Archive, Cologne. ZA4527 Data file Version 1.0.1, doi:10.4232/1.10981

European Commission (2012): Eurobarometer 66.2 (Oct-Nov 2006). TNS OPINION & SOCIAL, Brussels [Producer]. GESIS Data Archive, Cologne. ZA4527 Data file Version 1.0.1, doi:10.4232/1.10981

European Commission (2012): Eurobarometer 66.2 (Oct-Nov 2006). TNS OPINION & SOCIAL, Brussels [Producer]. GESIS Data Archive, Cologne. ZA4527 Data file Version 1.0.1, doi:10.4232/1.10981

European Commission (2012): Eurobarometer 66.2 (Oct-Nov 2006). TNS OPINION & SOCIAL, Brussels [Producer]. GESIS Data Archive, Cologne. ZA4527 Data file Version 1.0.1, doi:10.4232/1.10981

European Commission (2012): Eurobarometer 66.2 (Oct-Nov 2006). TNS OPINION & SOCIAL, Brussels [Producer]. GESIS Data Archive, Cologne. ZA4527 Data file Version 1.0.1, doi:10.4232/1.10981

European Commission (2012): Eurobarometer 66.2 (Oct-Nov 2006). TNS OPINION & SOCIAL, Brussels [Producer]. GESIS Data Archive, Cologne. ZA4527 Data file Version 1.0.1, doi:10.4232/1.10981

European Commission (2012): Eurobarometer 66.2 (Oct-Nov 2006). TNS OPINION & SOCIAL, Brussels [Producer]. GESIS Data Archive, Cologne. ZA4527 Data file Version 1.0.1, doi:10.4232/1.10981

European Commission (2012): Eurobarometer 66.2 (Oct-Nov 2006). TNS OPINION & SOCIAL, Brussels [Producer]. GESIS Data Archive, Cologne. ZA4527 Data file Version 1.0.1, doi:10.4232/1.10981

European Commission (2012): Eurobarometer 66.2 (Oct-Nov 2006). TNS OPINION & SOCIAL, Brussels [Producer]. GESIS Data Archive, Cologne. ZA4527 Data file Version 1.0.1, doi:10.4232/1.10981

European Commission (2012): Eurobarometer 66.2 (Oct-Nov 2006). TNS OPINION & SOCIAL, Brussels [Producer]. GESIS Data Archive, Cologne. ZA4527 Data file Version 1.0.1, doi:10.4232/1.10981

European Commission (2012): Eurobarometer 66.2 (Oct-Nov 2006). TNS OPINION & SOCIAL, Brussels [Producer]. GESIS Data Archive, Cologne. ZA4527 Data file Version 1.0.1, doi:10.4232/1.10981

European Commission (2012): Eurobarometer 66.2 (Oct-Nov 2006). TNS OPINION & SOCIAL, Brussels [Producer]. GESIS Data Archive, Cologne. ZA4527 Data file Version 1.0.1, doi:10.4232/1.10981

European Commission (2012): Eurobarometer 66.2 (Oct-Nov 2006). TNS OPINION & SOCIAL, Brussels [Producer]. GESIS Data Archive, Cologne. ZA4527 Data file Version 1.0.1, doi:10.4232/1.10981

European Commission (2012): Eurobarometer 66.2 (Oct-Nov 2006). TNS OPINION & SOCIAL, Brussels [Producer]. GESIS Data Archive, Cologne. ZA4527 Data file Version 1.0.1, doi:10.4232/1.10981

European Commission (2012): Eurobarometer 66.2 (Oct-Nov 2006). TNS OPINION & SOCIAL, Brussels [Producer]. GESIS Data Archive, Cologne. ZA4527 Data file Version 1.0.1, doi:10.4232/1.10981

European Commission (2012): Eurobarometer 66.2 (Oct-Nov 2006). TNS OPINION & SOCIAL, Brussels [Producer]. GESIS Data Archive, Cologne. ZA4527 Data file Version 1.0.1, doi:10.4232/1.10981

European Commission (2012): Eurobarometer 72.3 (Oct 2009). TNS OPINION & SOCIAL, Brussels [Producer]. GESIS Data Archive, Cologne. ZA4977 Data file Version 2.0.0, doi:10.4232/1.11140

European Commission (2012): Eurobarometer 72.3 (Oct 2009). TNS OPINION & SOCIAL, Brussels [Producer]. GESIS Data Archive, Cologne. ZA4977 Data file Version 2.0.0, doi:10.4232/1.11140

European Commission (2012): Eurobarometer 72.3 (Oct 2009). TNS OPINION & SOCIAL, Brussels [Producer]. GESIS Data Archive, Cologne. ZA4977 Data file Version 2.0.0, doi:10.4232/1.11140

European Commission (2012): Eurobarometer 72.3 (Oct 2009). TNS OPINION & SOCIAL, Brussels [Producer]. GESIS Data Archive, Cologne. ZA4977 Data file Version 2.0.0, doi:10.4232/1.11140

European Commission (2012): Eurobarometer 72.3 (Oct 2009). TNS OPINION & SOCIAL, Brussels [Producer]. GESIS Data Archive, Cologne. ZA4977 Data file Version 2.0.0, doi:10.4232/1.11140

European Commission (2012): Eurobarometer 72.3 (Oct 2009). TNS OPINION & SOCIAL, Brussels [Producer]. GESIS Data Archive, Cologne. ZA4977 Data file Version 2.0.0, doi:10.4232/1.11140

European Commission (2012): Eurobarometer 72.3 (Oct 2009). TNS OPINION & SOCIAL, Brussels [Producer]. GESIS Data Archive, Cologne. ZA4977 Data file Version 2.0.0, doi:10.4232/1.11140

European Commission (2012): Eurobarometer 72.3 (Oct 2009). TNS OPINION & SOCIAL, Brussels [Producer]. GESIS Data Archive, Cologne. ZA4977 Data file Version 2.0.0, doi:10.4232/1.11140

European Commission (2012): Eurobarometer 72.3 (Oct 2009). TNS OPINION & SOCIAL, Brussels [Producer]. GESIS Data Archive, Cologne. ZA4977 Data file Version 2.0.0, doi:10.4232/1.11140

European Commission (2012): Eurobarometer 72.3 (Oct 2009). TNS OPINION & SOCIAL, Brussels [Producer]. GESIS Data Archive, Cologne. ZA4977 Data file Version 2.0.0, doi:10.4232/1.11140

European Commission (2012): Eurobarometer 72.3 (Oct 2009). TNS OPINION & SOCIAL, Brussels [Producer]. GESIS Data Archive, Cologne. ZA4977 Data file Version 2.0.0, doi:10.4232/1.11140

European Commission (2012): Eurobarometer 72.3 (Oct 2009). TNS OPINION & SOCIAL, Brussels [Producer]. GESIS Data Archive, Cologne. ZA4977 Data file Version 2.0.0, doi:10.4232/1.11140

European Commission (2012): Eurobarometer 72.3 (Oct 2009). TNS OPINION & SOCIAL, Brussels [Producer]. GESIS Data Archive, Cologne. ZA4977 Data file Version 2.0.0, doi:10.4232/1.11140

European Commission (2012): Eurobarometer 72.3 (Oct 2009). TNS OPINION & SOCIAL, Brussels [Producer]. GESIS Data Archive, Cologne. ZA4977 Data file Version 2.0.0, doi:10.4232/1.11140

European Commission (2012): Eurobarometer 72.3 (Oct 2009). TNS OPINION & SOCIAL, Brussels [Producer]. GESIS Data Archive, Cologne. ZA4977 Data file Version 2.0.0, doi:10.4232/1.11140

European Commission (2012): Eurobarometer 72.3 (Oct 2009). TNS OPINION & SOCIAL, Brussels [Producer]. GESIS Data Archive, Cologne. ZA4977 Data file Version 2.0.0, doi:10.4232/1.11140

European Commission (2012): Eurobarometer 72.3 (Oct 2009). TNS OPINION & SOCIAL, Brussels [Producer]. GESIS Data Archive, Cologne. ZA4977 Data file Version 2.0.0, doi:10.4232/1.11140

European Commission (2012): Eurobarometer 77.1 (2012). TNS OPINION & SOCIAL, Brussels [Producer]. GESIS Data Archive, Cologne. ZA5597 Data file Version 2.0.0, doi:10.4232/1.11481

European Commission (2012): Eurobarometer 77.1 (2012). TNS OPINION & SOCIAL, Brussels [Producer]. GESIS Data Archive, Cologne. ZA5597 Data file Version 2.0.0, doi:10.4232/1.11481

European Commission (2012): Eurobarometer 77.1 (2012). TNS OPINION & SOCIAL, Brussels [Producer]. GESIS Data Archive, Cologne. ZA5597 Data file Version 2.0.0, doi:10.4232/1.11481

European Commission (2012): Eurobarometer 77.1 (2012). TNS OPINION & SOCIAL, Brussels [Producer]. GESIS Data Archive, Cologne. ZA5597 Data file Version 2.0.0, doi:10.4232/1.11481

European Commission (2012): Eurobarometer 77.1 (2012). TNS OPINION & SOCIAL, Brussels [Producer]. GESIS Data Archive, Cologne. ZA5597 Data file Version 2.0.0, doi:10.4232/1.11481

European Commission (2012): Eurobarometer 77.1 (2012). TNS OPINION & SOCIAL, Brussels [Producer]. GESIS Data Archive, Cologne. ZA5597 Data file Version 2.0.0, doi:10.4232/1.11481

European Commission (2012): Eurobarometer 77.1 (2012). TNS OPINION & SOCIAL, Brussels [Producer]. GESIS Data Archive, Cologne. ZA5597 Data file Version 2.0.0, doi:10.4232/1.11481

European Commission (2012): Eurobarometer 77.1 (2012). TNS OPINION & SOCIAL, Brussels [Producer]. GESIS Data Archive, Cologne. ZA5597 Data file Version 2.0.0, doi:10.4232/1.11481

European Commission (2012): Eurobarometer 77.1 (2012). TNS OPINION & SOCIAL, Brussels [Producer]. GESIS Data Archive, Cologne. ZA5597 Data file Version 2.0.0, doi:10.4232/1.11481

European Commission (2012): Eurobarometer 77.1 (2012). TNS OPINION & SOCIAL, Brussels [Producer]. GESIS Data Archive, Cologne. ZA5597 Data file Version 2.0.0, doi:10.4232/1.11481

European Commission (2012): Eurobarometer 77.1 (2012). TNS OPINION & SOCIAL, Brussels [Producer]. GESIS Data Archive, Cologne. ZA5597 Data file Version 2.0.0, doi:10.4232/1.11481

European Commission (2012): Eurobarometer 77.1 (2012). TNS OPINION & SOCIAL, Brussels [Producer]. GESIS Data Archive, Cologne. ZA5597 Data file Version 2.0.0, doi:10.4232/1.11481

European Commission (2012): Eurobarometer 77.1 (2012). TNS OPINION & SOCIAL, Brussels [Producer]. GESIS Data Archive, Cologne. ZA5597 Data file Version 2.0.0, doi:10.4232/1.11481

European Commission (2012): Eurobarometer 77.1 (2012). TNS OPINION & SOCIAL, Brussels [Producer]. GESIS Data Archive, Cologne. ZA5597 Data file Version 2.0.0, doi:10.4232/1.11481

European Commission (2012): Eurobarometer 77.1 (2012). TNS OPINION & SOCIAL, Brussels [Producer]. GESIS Data Archive, Cologne. ZA5597 Data file Version 2.0.0, doi:10.4232/1.11481

European Commission (2012): Eurobarometer 77.1 (2012). TNS OPINION & SOCIAL, Brussels [Producer]. GESIS Data Archive, Cologne. ZA5597 Data file Version 2.0.0, doi:10.4232/1.11481

European Monitoring Centre for Drugs and Drug Addiction (EMCDDA), European School Survey Project on Alcohol and Other Drugs (ESPAD), Pompidou Group, Council of Europe, Swedish Council for Information on Alcohol and Other Drugs (CAN). ESPAD Report 2007: Substance Use Among Students in 35 European Countries. Stockholm, Sweden: Swedish Council for Information on Alcohol and Other Drugs (CAN), 2009

European Monitoring Centre for Drugs and Drug Addiction (EMCDDA), European School Survey Project on Alcohol and Other Drugs (ESPAD), Pompidou Group, Council of Europe, Swedish Council for Information on Alcohol and Other Drugs (CAN). ESPAD Report 2011: Substance Use Among Students in 36 European Countries. Stockholm, Sweden: Swedish Council for Information on Alcohol and Other Drugs (CAN), 2012

European School Survey Project on Alcohol and Other Drugs (ESPAD), Pompidou Group, Council of Europe, Swedish Council for Information on Alcohol and Other Drugs (CAN). Alcohol and Drug Use Among European 17-18 Year Old Students: Data from the ESPAD Project. Stockholm, Sweden: Swedish Council for Information on Alcohol and Other Drugs (CAN), 2007

European School Survey Project on Alcohol and Other Drugs (ESPAD), Pompidou Group, Council of Europe, Swedish Council for Information on Alcohol and Other Drugs (CAN). ESPAD Report 2003: Alcohol and Other Drug Use Among Students in 35 European Countries. Stockholm, Sweden: Swedish Council for Information on Alcohol and Other Drugs (CAN), 2004

EURO-PERISTAT, EuroNeoStat, European Surveillance of Congenital Anomalies (EUROCAT), Netherlands Organisation for Applied Scientific Research (TNO), Surveillance of Cerebral Palsy in Europe (SCPE). European Perinatal Health Report 2004. EURO-PERISTAT, 2008

Eurostat, Ministry for Health, the Elderly, and Community Care (Malta), Ministry for Social Policy (Malta). Malta European Health Interview Survey 2008

Eurostat. Eurostat Tobacco Use Prevalence 1999

Federal Centre for Health Education (BZGA) (Germany). Germany Youth Drug Use in Germany 1997

Federal Centre for Health Education (BZGA) (Germany). Germany Youth Drug Use in Germany 2001

Federal Centre for Health Education (BZGA) (Germany). Germany Youth Drug Use in Germany 2004

Federal Environment Agency (Germany), Federal Institute for Drugs and Medical Devices (Germany), Max Planck Institute of Psychiatry, Robert Koch Institute. Germany National Health Interview and Examination Survey 1997-1999. Berlin, Germany: Robert Koch Institute, 2000

Federal Ministry of Food, Agriculture, and Consumer Protection (Germany), Max Rubner Institute. Germany National Nutrition Survey II 2005-2007. Max Rubner Institute

Federal Ministry of Health (Austria), Statistics Austria. Austria Health Survey 2006-2007

Federal Ministry of Health (Germany), Institute for Therapy and Health Research (IFT). Germany Population Survey on the Consumption of Psychoative Substances in the German Adult Population 2000

Federal Ministry of Health and Social Security (Germany), Institute for Therapy and Health Research (IFT). Germany Epidemiological Survey of Substance Abuse Among Adults 2003

Federal Office of Public Health (Switzerland). Switzerland PERMA Study 1974-1989

Federal Statistical Office (Germany). Germany Microcensus 1995

Federal Statistical Office (Germany). Germany Microcensus 2005

Federal Statistical Office (Germany). Germany Statistical Yearbook 2006. Wiesbaden, Germany: Federal Statistical Office (Germany), 2006

Federal Statistical Office (Germany). Germany Statistical Yearbook 2007. Wiesbaden, Germany: Federal Statistical Office (Germany), 2007

Federal Statistical Office (Germany). Germany Statistical Yearbook 2010. Wiesbaden, Germany: Federal Statistical Office (Germany), 2010

Federal Statistical Office (Switzerland), MIS Trend SA. Switzerland Health Survey 2007

Federal Statistical Office (Switzerland). Switzerland Health Survey 1993

Federal Statistical Office (Switzerland). Switzerland Health Survey 1997

Federal Statistical Office (Switzerland). Switzerland Health Survey 2002

Federal Statistical Office (Switzerland). Switzerland Health Survey 2012

Ferrante E, Muzzolon R, Fuso L, Pistelli R, M G, Ciappi G. The relationship between sporting activity and smoking habits in young adults. Mil Med. 1993; 158(11): 696-8 as it appears in P.N. Lee Statistics and Computing Ltd. International Mortality and Smoking Statistics Version 4.04. Sutton, United Kingdom: P.N. Lee Statistics and Computing Ltd, 2009

Field, J. et al. , National Survey of Sexual Attitudes and Lifestyles, 1990 [computer file]. Colchester, Essex: UK Data Archive [distributor], August 1995. SN: 3434, http://dx.doi.org/10.5255/UKDA-SN-3434-1

Filippidis FT, Vardavas CI, Loukopoulou A, Behrakis P, Connolly GN, Tountas Y. Prevalence and determinants of tobacco use among adults in Greece: 4 year trends. Eur J Public Health. 2013; 23(5): 772-6

Finland Adolescent Health and Lifestyle Survey 1981 as it appears in P.N. Lee Statistics and Computing Ltd. International Mortality and Smoking Statistics Version 4.04. Sutton, United Kingdom: P.N. Lee Statistics and Computing Ltd, 2009

Finland Adolescent Health and Lifestyle Survey 1983 as it appears in P.N. Lee Statistics and Computing Ltd. International Mortality and Smoking Statistics Version 4.04. Sutton, United Kingdom: P.N. Lee Statistics and Computing Ltd, 2009

Finland Adolescent Health and Lifestyle Survey 1985 as it appears in P.N. Lee Statistics and Computing Ltd. International Mortality and Smoking Statistics Version 4.04. Sutton, United Kingdom: P.N. Lee Statistics and Computing Ltd, 2009

Finland Adolescent Health and Lifestyle Survey 1987 as it appears in P.N. Lee Statistics and Computing Ltd. International Mortality and Smoking Statistics Version 4.04. Sutton, United Kingdom: P.N. Lee Statistics and Computing Ltd, 2009

Finland Adolescent Health and Lifestyle Survey 1989 as it appears in P.N. Lee Statistics and Computing Ltd. International Mortality and Smoking Statistics Version 4.04. Sutton, United Kingdom: P.N. Lee Statistics and Computing Ltd, 2009

Finland Adolescent Health and Lifestyle Survey 1991 as it appears in P.N. Lee Statistics and Computing Ltd. International Mortality and Smoking Statistics Version 4.04. Sutton, United Kingdom: P.N. Lee Statistics and Computing Ltd, 2009

Finland Adolescent Health and Lifestyle Survey 1993 as it appears in P.N. Lee Statistics and Computing Ltd. International Mortality and Smoking Statistics Version 4.04. Sutton, United Kingdom: P.N. Lee Statistics and Computing Ltd, 2009

Finland Adolescent Health and Lifestyle Survey 1995 as it appears in P.N. Lee Statistics and Computing Ltd. International Mortality and Smoking Statistics Version 4.04. Sutton, United Kingdom: P.N. Lee Statistics and Computing Ltd, 2009

Finland Adolescent Health and Lifestyle Survey 1997 as it appears in P.N. Lee Statistics and Computing Ltd. International Mortality and Smoking Statistics Version 4.04. Sutton, United Kingdom: P.N. Lee Statistics and Computing Ltd, 2009

Finland Adolescent Health and Lifestyle Survey 1999 as it appears in P.N. Lee Statistics and Computing Ltd. International Mortality and Smoking Statistics Version 4.04. Sutton, United Kingdom: P.N. Lee Statistics and Computing Ltd, 2009

Finland Health Behavior and Health Among the Adult Population 1980 as it appears in P.N. Lee Statistics and Computing Ltd. International Mortality and Smoking Statistics Version 4.04. Sutton, United Kingdom: P.N. Lee Statistics and Computing Ltd, 2009

Finland Health Behavior and Health Among the Adult Population 1981 as it appears in P.N. Lee Statistics and Computing Ltd. International Mortality and Smoking Statistics Version 4.04. Sutton, United Kingdom: P.N. Lee Statistics and Computing Ltd, 2009

Finland Health Behavior and Health Among the Adult Population 1983 as it appears in P.N. Lee Statistics and Computing Ltd. International Mortality and Smoking Statistics Version 4.04. Sutton, United Kingdom: P.N. Lee Statistics and Computing Ltd, 2009

Finland Health Behavior and Health Among the Adult Population 1984 as it appears in P.N. Lee Statistics and Computing Ltd. International Mortality and Smoking Statistics Version 4.04. Sutton, United Kingdom: P.N. Lee Statistics and Computing Ltd, 2009

Finland Health Behavior and Health Among the Adult Population 1985 as it appears in P.N. Lee Statistics and Computing Ltd. International Mortality and Smoking Statistics Version 4.04. Sutton, United Kingdom: P.N. Lee Statistics and Computing Ltd, 2009

Finland Health Behavior and Health Among the Adult Population 1987 as it appears in P.N. Lee Statistics and Computing Ltd. International Mortality and Smoking Statistics Version 4.04. Sutton, United Kingdom: P.N. Lee Statistics and Computing Ltd, 2009

Finland Health Behavior and Health Among the Adult Population 1988 as it appears in P.N. Lee Statistics and Computing Ltd. International Mortality and Smoking Statistics Version 4.04. Sutton, United Kingdom: P.N. Lee Statistics and Computing Ltd, 2009

Finland Health Behavior and Health Among the Adult Population 1989 as it appears in P.N. Lee Statistics and Computing Ltd. International Mortality and Smoking Statistics Version 4.04. Sutton, United Kingdom: P.N. Lee Statistics and Computing Ltd, 2009

Finland Health Behavior and Health Among the Adult Population 1991 as it appears in P.N. Lee Statistics and Computing Ltd. International Mortality and Smoking Statistics Version 4.04. Sutton, United Kingdom: P.N. Lee Statistics and Computing Ltd, 2009

Finland Health Behavior and Health Among the Adult Population 1992 as it appears in P.N. Lee Statistics and Computing Ltd. International Mortality and Smoking Statistics Version 4.04. Sutton, United Kingdom: P.N. Lee Statistics and Computing Ltd, 2009

Finland Health Behavior and Health Among the Adult Population 1994 as it appears in P.N. Lee Statistics and Computing Ltd. International Mortality and Smoking Statistics Version 4.04. Sutton, United Kingdom: P.N. Lee Statistics and Computing Ltd, 2009

Finland Health Behavior and Health Among the Adult Population 1995 as it appears in P.N. Lee Statistics and Computing Ltd. International Mortality and Smoking Statistics Version 4.04. Sutton, United Kingdom: P.N. Lee Statistics and Computing Ltd, 2009

Finland Health Behavior and Health Among the Adult Population 1997 as it appears in P.N. Lee Statistics and Computing Ltd. International Mortality and Smoking Statistics Version 4.04. Sutton, United Kingdom: P.N. Lee Statistics and Computing Ltd, 2009

Finland Health Behavior and Health Among the Finnish Elderly Population 1985 as it appears in P.N. Lee Statistics and Computing Ltd. International Mortality and Smoking Statistics Version 4.04. Sutton, United Kingdom: P.N. Lee Statistics and Computing Ltd, 2009

Finland Health Behavior and Health Among the Finnish Elderly Population 1987 as it appears in P.N. Lee Statistics and Computing Ltd. International Mortality and Smoking Statistics Version 4.04. Sutton, United Kingdom: P.N. Lee Statistics and Computing Ltd, 2009

Finland Health Behavior and Health Among the Finnish Elderly Population 1989 as it appears in P.N. Lee Statistics and Computing Ltd. International Mortality and Smoking Statistics Version 4.04. Sutton, United Kingdom: P.N. Lee Statistics and Computing Ltd, 2009

Finland Health Behavior and Health Among the Finnish Elderly Population 1993 as it appears in P.N. Lee Statistics and Computing Ltd. International Mortality and Smoking Statistics Version 4.04. Sutton, United Kingdom: P.N. Lee Statistics and Computing Ltd, 2009

Finland Health Behavior and Health Among the Finnish Elderly Population 1995 as it appears in P.N. Lee Statistics and Computing Ltd. International Mortality and Smoking Statistics Version 4.04. Sutton, United Kingdom: P.N. Lee Statistics and Computing Ltd, 2009

Finland Health Behavior and Health Among the Finnish Elderly Population 1997 as it appears in P.N. Lee Statistics and Computing Ltd. International Mortality and Smoking Statistics Version 4.04. Sutton, United Kingdom: P.N. Lee Statistics and Computing Ltd, 2009

Finland Health Behavior and Health Among the Finnish Elderly Population 1999 as it appears in P.N. Lee Statistics and Computing Ltd. International Mortality and Smoking Statistics Version 4.04. Sutton, United Kingdom: P.N. Lee Statistics and Computing Ltd, 2009

Food and Nutrition Services, Ministry of Health (Israel), Israel Center for Disease Control (ICDC). Israel National Health and Nutrition Survey 1999-2001

France Adult Health Barometer 1993 as it appears in P.N. Lee Statistics and Computing Ltd. International Mortality and Smoking Statistics Version 4.04. Sutton, United Kingdom: P.N. Lee Statistics and Computing Ltd, 2009

France CFES Adolescent Health Survey 1981 as it appears in P.N. Lee Statistics and Computing Ltd. International Mortality and Smoking Statistics Version 4.04. Sutton, United Kingdom: P.N. Lee Statistics and Computing Ltd, 2009

France CFES Adolescent Health Survey 1983 as it appears in P.N. Lee Statistics and Computing Ltd. International Mortality and Smoking Statistics Version 4.04. Sutton, United Kingdom: P.N. Lee Statistics and Computing Ltd, 2009

France CFES Adolescent Health Survey 1984 as it appears in P.N. Lee Statistics and Computing Ltd. International Mortality and Smoking Statistics Version 4.04. Sutton, United Kingdom: P.N. Lee Statistics and Computing Ltd, 2009

France CFES Adolescent Health Survey 1994 as it appears in P.N. Lee Statistics and Computing Ltd. International Mortality and Smoking Statistics Version 4.04. Sutton, United Kingdom: P.N. Lee Statistics and Computing Ltd, 2009

France CFES Adult Health Survey 1986 as it appears in P.N. Lee Statistics and Computing Ltd. International Mortality and Smoking Statistics Version 4.04. Sutton, United Kingdom: P.N. Lee Statistics and Computing Ltd, 2009

France CFES Adult Health Survey 1989 as it appears in P.N. Lee Statistics and Computing Ltd. International Mortality and Smoking Statistics Version 4.04. Sutton, United Kingdom: P.N. Lee Statistics and Computing Ltd, 2009

France CFES Adult Health Survey 1992 as it appears in P.N. Lee Statistics and Computing Ltd. International Mortality and Smoking Statistics Version 4.04. Sutton, United Kingdom: P.N. Lee Statistics and Computing Ltd, 2009

France CFES Adult Health Survey 1995 as it appears in P.N. Lee Statistics and Computing Ltd. International Mortality and Smoking Statistics Version 4.04. Sutton, United Kingdom: P.N. Lee Statistics and Computing Ltd, 2009

France CFES Adult Health Survey Data 1999 as it appears in P.N. Lee Statistics and Computing Ltd. International Mortality and Smoking Statistics Version 4.04. Sutton, United Kingdom: P.N. Lee Statistics and Computing Ltd, 2009

France CFES Campaign Evaluation Survey 1988 as it appears in P.N. Lee Statistics and Computing Ltd. International Mortality and Smoking Statistics Version 4.04. Sutton, United Kingdom: P.N. Lee Statistics and Computing Ltd, 2009

France CFES Youth and Tobacco Survey 1980 as it appears in P.N. Lee Statistics and Computing Ltd. International Mortality and Smoking Statistics Version 4.04. Sutton, United Kingdom: P.N. Lee Statistics and Computing Ltd, 2009

France CFES Youth and Tobacco Survey 1990 as it appears in P.N. Lee Statistics and Computing Ltd. International Mortality and Smoking Statistics Version 4.04. Sutton, United Kingdom: P.N. Lee Statistics and Computing Ltd, 2009

France Decennial Survey on Health and Medical Care 1980-1981 as it appears in P.N. Lee Statistics and Computing Ltd. International Mortality and Smoking Statistics Version 4.04. Sutton, United Kingdom: P.N. Lee Statistics and Computing Ltd, 2009

France Decennial Survey on Health and Medical Care 1991-1992 as it appears in P.N. Lee Statistics and Computing Ltd. International Mortality and Smoking Statistics Version 4.04. Sutton, United Kingdom: P.N. Lee Statistics and Computing Ltd, 2009

France Eurobarometer 32: The Single European Market, Drugs, Alcohol, and Cancer 1989 as it appears in P.N. Lee Statistics and Computing Ltd. International Mortality and Smoking Statistics Version 4.04. Sutton, United Kingdom: P.N. Lee Statistics and Computing Ltd, 2009

France Eurobarometer 38: European Court of Justice, Passive Smoking, And Consumer Issues 1992 as it appears in P.N. Lee Statistics and Computing Ltd. International Mortality and Smoking Statistics Version 4.04. Sutton, United Kingdom: P.N. Lee Statistics and Computing Ltd, 2009

France Survey of Living Conditions of People in Disadvantaged Situations 1986-1987 as it appears in P.N. Lee Statistics and Computing Ltd. International Mortality and Smoking Statistics Version 4.04. Sutton, United Kingdom: P.N. Lee Statistics and Computing Ltd, 2009

French Monitoring Centre for Drugs and Drug Addiction, National Service Directorate (France). France Survey on Health and Consumption During the Day of Defense Preparation 2008

French Monitoring Centre for Drugs and Drug Addiction. France Survey on Health and Consumption During the Day of Defense Preparation 2000

French Monitoring Centre for Drugs and Drug Addiction. France Survey on Health and Consumption During the Day of Defense Preparation 2001

French Monitoring Centre for Drugs and Drug Addiction. France Survey on Health and Consumption During the Day of Defense Preparation 2002

French Monitoring Centre for Drugs and Drug Addiction. France Survey on Health and Consumption During the Day of Defense Preparation 2003

French Monitoring Centre for Drugs and Drug Addiction. France Survey on Health and Consumption During the Day of Defense Preparation 2005

French Monitoring Centre for Drugs and Drug Addiction. France Survey on Health and Consumption During the Day of Defense Preparation 2014

Gallup Poll. Iceland Prevalence of Smoking 2005

Gallus S, Colombo P, Scarpino V, Zuccaro P, Apolone G, La Vecchia C. Smoking in Italy, 2002. Tumori. 2002; 88(6): 453-6

Gartner CE, Barendregt JJ, Hall WD. Predicting the future prevalence of cigarette smoking in Australia: how low can we go and by when?. Tob Control. 2009; 18(3): 183-9

Germany Eurobarometer 38: European Court of Justice, Passive Smoking, And Consumer Issues 1992 as it appears in P.N. Lee Statistics and Computing Ltd. International Mortality and Smoking Statistics Version 4.04. Sutton, United Kingdom: P.N. Lee Statistics and Computing Ltd, 2009

Germany Microcensus 1992 as it appears in P.N. Lee Statistics and Computing Ltd. International Mortality and Smoking Statistics Version 4.04. Sutton, United Kingdom: P.N. Lee Statistics and Computing Ltd, 2009

Germany Microcensus 1999 as it appears in P.N. Lee Statistics and Computing Ltd. International Mortality and Smoking Statistics Version 4.04. Sutton, United Kingdom: P.N. Lee Statistics and Computing Ltd, 2009

Germany Microcensus 2003 as it appears in P.N. Lee Statistics and Computing Ltd. International Mortality and Smoking Statistics Version 4.04. Sutton, United Kingdom: P.N. Lee Statistics and Computing Ltd, 2009

Germany Telephone Health Survey 2002-2003 as it appears in P.N. Lee Statistics and Computing Ltd. International Mortality and Smoking Statistics Version 4.04. Sutton, United Kingdom: P.N. Lee Statistics and Computing Ltd, 2009

Gilbert, G.N. et al. , General Household Survey (Surrey Files), 1980 [computer file]. Office of Population Censuses and Surveys. Social Survey Division, [original data producer(s)]. Colchester, Essex: UK Data Archive [distributor], 1984. SN: 1897, http://dx.doi.org/10.5255/UKDA-SN-1897-1

Gilbert, G.N. et al., General Household Survey (Surrey Files), 1982 [computer file]. Office of Population Censuses and Surveys. Social Survey Division, [original data producer(s)]. Colchester, Essex: UK Data Archive [distributor], June 1986. SN: 2127, http://dx.doi.org/10.5255/UKDA-SN-2127-1

Gliksman MD, Dwyer T, Wlodarczyk J, Pierce JP. Cigarette smoking in Australian schoolchildren. Med J Aust. 1989; 150(2): 81–4

Government Delegation for the National Plan on Drugs (Spain), Ministry of Health, Social Services and Equality (Spain). Spain Household Survey on Alcohol and Drugs 2013

Government Delegation for the National Plan on Drugs (Spain), Ministry of Health, Social Services and Equality (Spain). Spain Survey on Drug Use in Secondary Schools 2002

Government Delegation for the National Plan on Drugs (Spain). Spain Household Survey on Alcohol and Drugs 1997

Government Delegation for the National Plan on Drugs (Spain). Spain Household Survey on Alcohol and Drugs 1999

Government Delegation for the National Plan on Drugs (Spain). Spain Household Survey on Alcohol and Drugs 2001

Government Delegation for the National Plan on Drugs (Spain). Spain Household Survey on Alcohol and Drugs 2003-2004

Government Delegation for the National Plan on Drugs (Spain). Spain Survey on Drug Use in Secondary Schools 1998

Government Delegation for the National Plan on Drugs (Spain). Spain Survey on Drug Use in Secondary Schools 2000

Gredler B, Kunze M. Impact of a national campaign on smoking attitudes and patterns in Austria. Int J Health Educ. 1981; 24(4): 271-9 as it appears in P.N. Lee Statistics and Computing Ltd. International Mortality and Smoking Statistics Version 4.04. Sutton, United Kingdom: P.N. Lee Statistics and Computing Ltd, 2009

Haidinger G, Waldhoer T, Vutuc C. The prevalence of smoking in Austria. Prev Med. 1998; 27(1): 50-5 as it appears in P.N. Lee Statistics and Computing Ltd. International Mortality and Smoking Statistics Version 4.04. Sutton, United Kingdom: P.N. Lee Statistics and Computing Ltd, 2009

Harris, Kathleen Mullan, and J. Richard Udry. National Longitudinal Study of Adolescent to Adult Health (Add Health), 1994-2008. ICPSR21600-v12. Ann Arbor, MI: Inter-university Consortium for Political and Social Research [distributor], 2013-03-08. doi:10.3886/ICPSR21600.v12

Harris, Kathleen Mullan, and J. Richard Udry. National Longitudinal Study of Adolescent to Adult Health (Add Health), 1994-2008. ICPSR21600-v12. Ann Arbor, MI: Inter-university Consortium for Political and Social Research [distributor], 2013-03-08. doi:10.3886/ICPSR21600.v12

Harris, Kathleen Mullan, and J. Richard Udry. National Longitudinal Study of Adolescent to Adult Health (Add Health), 1994-2008. ICPSR21600-v12. Ann Arbor, MI: Inter-university Consortium for Political and Social Research [distributor], 2013-03-08. doi:10.3886/ICPSR21600.v12

Harris, Kathleen Mullan, and J. Richard Udry. National Longitudinal Study of Adolescent to Adult Health (Add Health), 1994-2008. ICPSR21600-v12. Ann Arbor, MI: Inter-university Consortium for Political and Social Research [distributor], 2013-03-08. doi:10.3886/ICPSR21600.v12

Health Canada, Heart and Stroke Foundation of Canada. Canada Heart Health Database 1986-1992

Health Canada, Statistics Canada. Canada Tobacco Use Monitoring Survey 1999

Health Canada, Statistics Canada. Canada Tobacco Use Monitoring Survey 2000

Health Canada, Statistics Canada. Canada Tobacco Use Monitoring Survey 2001

Health Canada, Statistics Canada. Canada Tobacco Use Monitoring Survey 2002

Health Canada, Statistics Canada. Canada Tobacco Use Monitoring Survey 2003. Ottawa, Canada: Statistics Canada

Health Canada, Statistics Canada. Canada Tobacco Use Monitoring Survey 2004

Health Canada, Statistics Canada. Canada Tobacco Use Monitoring Survey 2005

Health Canada, Statistics Canada. Canada Tobacco Use Monitoring Survey 2006

Health Canada, Statistics Canada. Canada Tobacco Use Monitoring Survey 2007

Health Canada, Statistics Canada. Canada Tobacco Use Monitoring Survey 2008

Health Canada, Statistics Canada. Canada Tobacco Use Monitoring Survey 2011

Health Canada, Statistics Canada. Canada Tobacco Use Monitoring Survey 2012. Ottawa, Canada: Statistics Canada, 2013

Health Canada, Statistics Canada. Canada Youth Smoking Survey 1994

Health Promotion Board (Singapore). Singapore Student Health Survey 2006

Health Promotion Board (Singapore). Singapore Student Health Survey 2009

Health Sponsorship Council (New Zealand). New Zealand Year In-depth Survey 2006

Hercberg S, Preziosi P, Brian�on S, Galan P, Triol I, Malvy D, Roussel AM, Favier A. A primary prevention trial using nutritional doses of antioxidant vitamins and minerals in cardiovascular diseases and cancers in a general population: the SU.VI.MAX Study-design, methods, and participant characteristics. Control Clin Trials. 1998; 19(4): 336–51

Hill D, White V, Letcher T. Tobacco use among Australian secondary students in 1996. Aust N Z J Public Health. 1999; 23(3): 252–9

Hill D, Willcox S, Gardner G, Houston J. Tobacco and alcohol use among Australian secondary schoolchildren. Med J Aust. 1987; 146(3): 125–30

Hill DJ, White VM, Pain MD, Gardner GJ. Tobacco and alcohol use among Australian secondary schoolchildren in 1987. Med J Aust. 1990; 152(3): 124–30

Hill DJ, White VM, Scollo MM. Smoking behaviours of Australian adults in 1995: trends and concerns. Med J Aust. 1998; 168(5): 209-13

Hill DJ, White VM, Williams RM, Gardner GJ. Tobacco and alcohol use among Australian secondary school students in 1990. Med J Aust. 1993; 158(4): 228–34

Honorary Commission for Cardiovascular Health (Uruguay). Uruguay Cardiovascular Disease, Epidemiology, and Statistics 1990-1992. Montevideo, Uruguay: Honorary Commission for Cardiovascular Health (Uruguay), 1996

IARC Monographs On The Evaluation Of The Carcinogenic Risk Of Chemicals To Humans: Tobacco Smoking as it appears in P.N. Lee Statistics and Computing Ltd. International Mortality and Smoking Statistics Version 4.04. Sutton, United Kingdom: P.N. Lee Statistics and Computing Ltd, 2009

Ibsen KK. Smoking Habits in 9000 Danish Schoolchildren. Acta Paediatr. 1982; 71(1): 131-4 as it appears in P.N. Lee Statistics and Computing Ltd. International Mortality and Smoking Statistics Version 4.04. Sutton, United Kingdom: P.N. Lee Statistics and Computing Ltd, 2009

Icelandic Centre for Social Research and Analysis (ICSRA). Iceland Youth Survey 1992

Icelandic Centre for Social Research and Analysis (ICSRA). Iceland Youth Survey 1997

Icelandic Centre for Social Research and Analysis (ICSRA). Iceland Youth Survey 2000. Reykjavik, Iceland: Icelandic Centre for Social Research and Analysis (ICSRA)

Icelandic Centre for Social Research and Analysis (ICSRA). Iceland Youth Survey 2001

Icelandic Centre for Social Research and Analysis (ICSRA). Iceland Youth Survey 2003

Icelandic Centre for Social Research and Analysis (ICSRA). Iceland Youth Survey 2004

Icelandic Centre for Social Research and Analysis (ICSRA). Iceland Youth Survey 2005

Icelandic Centre for Social Research and Analysis (ICSRA). Iceland Youth Survey 2006

Icelandic Centre for Social Research and Analysis (ICSRA). Iceland Youth Survey 2007

Icelandic Centre for Social Research and Analysis (ICSRA). Iceland Youth Survey 2008

Icelandic Centre for Social Research and Analysis (ICSRA). Iceland Youth Survey 2009

Icelandic Centre for Social Research and Analysis (ICSRA). Iceland Youth Survey 2010

Icelandic Centre for Social Research and Analysis (ICSRA). Iceland Youth Survey 2011

Icelandic Centre for Social Research and Analysis (ICSRA). Iceland Youth Survey 2012

Icelandic Centre for Social Research and Analysis (ICSRA). Iceland Youth Survey 2013

Icelandic Centre for Social Research and Analysis (ICSRA). Iceland Youth Survey 2014

ICF Macro, Office on Smoking and Health, Centers for Disease Control and Prevention (CDC). United States National Adult Tobacco Survey 2009-2010. Atlanta, United States: Office on Smoking and Health, Centers for Disease Control and Prevention (CDC)

Information Center for Health and Social Care, National Health Service (United Kingdom). United Kingdom Statistics on Smoking 2006-2007. Leeds, United Kingdom, Information Center for Health and Social Care, National Health Service (United Kingdom), licensed under the Open Government License v.1.0.Copyright © 2012, Re-used with the permission of The Health and Social Care Information Centre. All rights reserved

Institute for Social and Economic Research, University of Essex, Gfk NOP, Office for National Statistics (ONS) (United Kingdom), Economic and Social Research Council (ESRC), Health Development Agency (United Kingdom). United Kingdom British Household Panel Survey 2000-2001. Essex, United Kingdom: Institute for Social and Economic Research, University of Essex

Institute for Social and Economic Research, University of Essex, Gfk NOP, Office for National Statistics (ONS) (United Kingdom), Economic and Social Research Council (ESRC), Health Education Authority (HEA) (United Kingdom). United Kingdom British Household Panel Survey 1994-1995. Essex, United Kingdom: Institute for Social and Economic Research, University of Essex

Institute for Social and Economic Research, University of Essex, Gfk NOP, Office for National Statistics (ONS) (United Kingdom), Economic and Social Research Council (ESRC), Health Education Authority (HEA) (United Kingdom). United Kingdom British Household Panel Survey 1995-1996. Essex, United Kingdom: Institute for Social and Economic Research, University of Essex

Institute for Social and Economic Research, University of Essex, Gfk NOP, Office for National Statistics (ONS) (United Kingdom), Economic and Social Research Council (ESRC), Health Education Authority (HEA) (United Kingdom). United Kingdom British Household Panel Survey 1996-1997. Essex, United Kingdom: Institute for Social and Economic Research, University of Essex

Institute for Social and Economic Research, University of Essex, Gfk NOP, Office for National Statistics (ONS) (United Kingdom), Economic and Social Research Council (ESRC), Health Education Authority (HEA) (United Kingdom). United Kingdom British Household Panel Survey 1997-1998. Essex, United Kingdom: Institute for Social and Economic Research, University of Essex

Institute for Social and Economic Research, University of Essex, Gfk NOP, Office for National Statistics (ONS) (United Kingdom), Economic and Social Research Council (ESRC), Health Education Authority (HEA) (United Kingdom). United Kingdom British Household Panel Survey 1998-1999. Essex, United Kingdom: Institute for Social and Economic Research, University of Essex

Institute for Social and Economic Research, University of Essex, Gfk NOP, Office for National Statistics (ONS) (United Kingdom), Economic and Social Research Council (ESRC), Health Education Authority (HEA) (United Kingdom). United Kingdom British Household Panel Survey 1999-2000. Essex, United Kingdom: Institute for Social and Economic Research, University of Essex

Institute for Social and Economic Research, University of Essex, Gfk NOP, Office for National Statistics (ONS) (United Kingdom), Economic and Social Research Council (ESRC). United Kingdom British Household Panel Survey 1991-1992. Essex, United Kingdom: Institute for Social and Economic Research, University of Essex

Institute for Social and Economic Research, University of Essex, Gfk NOP, Office for National Statistics (ONS) (United Kingdom), Economic and Social Research Council (ESRC). United Kingdom British Household Panel Survey 1992-1993. Essex, United Kingdom: Institute for Social and Economic Research, University of Essex

Institute for Social and Economic Research, University of Essex, Gfk NOP, Office for National Statistics (ONS) (United Kingdom), Economic and Social Research Council (ESRC). United Kingdom British Household Panel Survey 1993-1994. Essex, United Kingdom: Institute for Social and Economic Research, University of Essex

Institute for Social and Economic Research, University of Essex, Gfk NOP, Office for National Statistics (ONS) (United Kingdom), Northern Ireland Statistics and Research Agency (NISRA), Economic and Social Research Council (ESRC), Health Development Agency (United Kingdom). United Kingdom British Household Panel Survey 2001-2002. Essex, United Kingdom: Institute for Social and Economic Research, University of Essex

Institute for Social and Economic Research, University of Essex, Gfk NOP, Office for National Statistics (ONS) (United Kingdom), Northern Ireland Statistics and Research Agency (NISRA), Economic and Social Research Council (ESRC), Health Development Agency (United Kingdom). United Kingdom British Household Panel Survey 2002-2003. Essex, United Kingdom: Institute for Social and Economic Research, University of Essex

Institute for Social and Economic Research, University of Essex, Gfk NOP, Office for National Statistics (ONS) (United Kingdom), Northern Ireland Statistics and Research Agency (NISRA), Economic and Social Research Council (ESRC), Health Development Agency (United Kingdom). United Kingdom British Household Panel Survey 2003-2004. Essex, United Kingdom: Institute for Social and Economic Research, University of Essex

Institute for Social and Economic Research, University of Essex, Gfk NOP, Office for National Statistics (ONS) (United Kingdom), Northern Ireland Statistics and Research Agency (NISRA), Economic and Social Research Council (ESRC), Health Development Agency (United Kingdom). United Kingdom British Household Panel Survey 2004-2005. Essex, United Kingdom: Institute for Social and Economic Research, University of Essex

Institute for Social and Economic Research, University of Essex, Gfk NOP, Office for National Statistics (ONS) (United Kingdom), Northern Ireland Statistics and Research Agency (NISRA), Economic and Social Research Council (ESRC), National Institute for Health and Clinical Excellence (NICE). United Kingdom British Household Panel Survey 2005-2006. Essex, United Kingdom: Institute for Social and Economic Research, University of Essex

Institute for Social and Economic Research, University of Essex, Gfk NOP, Office for National Statistics (ONS) (United Kingdom), Northern Ireland Statistics and Research Agency (NISRA), Economic and Social Research Council (ESRC), National Institute for Health and Clinical Excellence (NICE). United Kingdom British Household Panel Survey 2006-2007. Essex, United Kingdom: Institute for Social and Economic Research, University of Essex

Institute for Social and Economic Research, University of Essex, Gfk NOP, Office for National Statistics (ONS) (United Kingdom), Northern Ireland Statistics and Research Agency (NISRA), Economic and Social Research Council (ESRC), National Institute for Health and Clinical Excellence (NICE). United Kingdom British Household Panel Survey 2007-2008. Essex, United Kingdom: Institute for Social and Economic Research, University of Essex

Institute for Social and Economic Research, University of Essex, Gfk NOP, Office for National Statistics (ONS) (United Kingdom), Northern Ireland Statistics and Research Agency (NISRA), Economic and Social Research Council (ESRC), National Institute for Health and Clinical Excellence (NICE). United Kingdom British Household Panel Survey 2008-2009. Essex, United Kingdom: Institute for Social and Economic Research, University of Essex

Institute of Basic Medical Sciences, University of Oslo, Norwegian Directorate of Health, Norwegian Food Safety Authority. Norway NORKOST National Food Consumption Survey 2010-2011

Inter-American Drug Abuse Control Commission (CICAD), Organization of American States (OAS), National Commission for Development and Life without Drugs (DEVIDA) (Peru), National Council for Narcotics Control (CONACE) (Chile), National Council for the Control of Narcotic and Psychotropic Substances (CONSEP), National Drug Board (JND), Secretariat for Programming Drug Abuse Prevention and the Fight against Drug Trafficking (SEDRONAR), The National Council for the Fight against Illicit Drug Trafficking (CONALTID), United Nations Office on Drugs and Crime (UNODC). Youth and Drugs in South American Countries: A Public Policy Challenge. Washington, D.C., United States: Inter-American Drug Abuse Control Commission (CICAD), Organization of American States (OAS), 2006

Inter-American Drug Abuse Control Commission (CICAD), Organization of American States (OAS). CICAD Comparative Report on Nationwide School Surveys in Seven Countries: El Salvador, Guatemala, Nicaragua, Panama, Paraguay, Dominican Republic, and Uruguay 2003. Washington, D.C., United States: Inter-American Drug Abuse Control Commission (CICAD), Organization of American States (OAS), 2004

International Diabetes Institute (IDI). Australia Diabetes, Obesity and Lifestyle Study 1999-2000. Melbourne, Australia: International Diabetes Institute (IDI)

Ireland Joint National Listenership Research Survey 1990 as it appears in P.N. Lee Statistics and Computing Ltd. International Mortality and Smoking Statistics Version 4.04. Sutton, United Kingdom: P.N. Lee Statistics and Computing Ltd, 2009

Ireland Joint National Listenership Research Survey 1991 as it appears in P.N. Lee Statistics and Computing Ltd. International Mortality and Smoking Statistics Version 4.04. Sutton, United Kingdom: P.N. Lee Statistics and Computing Ltd, 2009

Ireland Joint National Listenership Research Survey 1992 as it appears in P.N. Lee Statistics and Computing Ltd. International Mortality and Smoking Statistics Version 4.04. Sutton, United Kingdom: P.N. Lee Statistics and Computing Ltd, 2009

Ireland Joint National Listenership Research Survey 1993 as it appears in P.N. Lee Statistics and Computing Ltd. International Mortality and Smoking Statistics Version 4.04. Sutton, United Kingdom: P.N. Lee Statistics and Computing Ltd, 2009

Ireland Joint National Media Research Survey 1980 as it appears in P.N. Lee Statistics and Computing Ltd. International Mortality and Smoking Statistics Version 4.04. Sutton, United Kingdom: P.N. Lee Statistics and Computing Ltd, 2009

Ireland Joint National Media Research Survey 1981 as it appears in P.N. Lee Statistics and Computing Ltd. International Mortality and Smoking Statistics Version 4.04. Sutton, United Kingdom: P.N. Lee Statistics and Computing Ltd, 2009

Ireland Joint National Media Research Survey 1982 as it appears in P.N. Lee Statistics and Computing Ltd. International Mortality and Smoking Statistics Version 4.04. Sutton, United Kingdom: P.N. Lee Statistics and Computing Ltd, 2009

Ireland Joint National Media Research Survey 1983 as it appears in P.N. Lee Statistics and Computing Ltd. International Mortality and Smoking Statistics Version 4.04. Sutton, United Kingdom: P.N. Lee Statistics and Computing Ltd, 2009

Ireland Joint National Media Research Survey 1984 as it appears in P.N. Lee Statistics and Computing Ltd. International Mortality and Smoking Statistics Version 4.04. Sutton, United Kingdom: P.N. Lee Statistics and Computing Ltd, 2009

Ireland Joint National Media Research Survey 1985 as it appears in P.N. Lee Statistics and Computing Ltd. International Mortality and Smoking Statistics Version 4.04. Sutton, United Kingdom: P.N. Lee Statistics and Computing Ltd, 2009

Ireland Joint National Media Research Survey 1986 as it appears in P.N. Lee Statistics and Computing Ltd. International Mortality and Smoking Statistics Version 4.04. Sutton, United Kingdom: P.N. Lee Statistics and Computing Ltd, 2009

Ireland Joint National Media Research Survey 1987 as it appears in P.N. Lee Statistics and Computing Ltd. International Mortality and Smoking Statistics Version 4.04. Sutton, United Kingdom: P.N. Lee Statistics and Computing Ltd, 2009

Ireland Joint National Media Research Survey 1988 as it appears in P.N. Lee Statistics and Computing Ltd. International Mortality and Smoking Statistics Version 4.04. Sutton, United Kingdom: P.N. Lee Statistics and Computing Ltd, 2009

Ireland Joint National Media Research Survey 1989 as it appears in P.N. Lee Statistics and Computing Ltd. International Mortality and Smoking Statistics Version 4.04. Sutton, United Kingdom: P.N. Lee Statistics and Computing Ltd, 2009

Israel Center for Disease Control (ICDC), Ministry of Health (Israel), World Health Organization Regional Office for Europe (WHO/Europe). Israel National Health Interview Survey 2003-2004

ISSP Research Group (2009): International Social Survey Programme: Health and Health Care - ISSP 2011. GESIS Data Archive, Cologne. ZA5800 Data file version 3.0.0, doi:10.4232/1.12252

ISSP Research Group (2009): International Social Survey Programme: Health and Health Care - ISSP 2011. GESIS Data Archive, Cologne. ZA5800 Data file version 3.0.0, doi:10.4232/1.12252

ISSP Research Group (2009): International Social Survey Programme: Health and Health Care - ISSP 2011. GESIS Data Archive, Cologne. ZA5800 Data file version 3.0.0, doi:10.4232/1.12252

ISSP Research Group (2009): International Social Survey Programme: Health and Health Care - ISSP 2011. GESIS Data Archive, Cologne. ZA5800 Data file version 3.0.0, doi:10.4232/1.12252

ISSP Research Group (2009): International Social Survey Programme: Health and Health Care - ISSP 2011. GESIS Data Archive, Cologne. ZA5800 Data file version 3.0.0, doi:10.4232/1.12252

ISSP Research Group (2009): International Social Survey Programme: Health and Health Care - ISSP 2011. GESIS Data Archive, Cologne. ZA5800 Data file version 3.0.0, doi:10.4232/1.12252

ISSP Research Group (2009): International Social Survey Programme: Health and Health Care - ISSP 2011. GESIS Data Archive, Cologne. ZA5800 Data file version 3.0.0, doi:10.4232/1.12252

ISSP Research Group (2009): International Social Survey Programme: Health and Health Care - ISSP 2011. GESIS Data Archive, Cologne. ZA5800 Data file version 3.0.0, doi:10.4232/1.12252

ISSP Research Group (2009): International Social Survey Programme: Health and Health Care - ISSP 2011. GESIS Data Archive, Cologne. ZA5800 Data file version 3.0.0, doi:10.4232/1.12252

ISSP Research Group (2009): International Social Survey Programme: Health and Health Care - ISSP 2011. GESIS Data Archive, Cologne. ZA5800 Data file version 3.0.0, doi:10.4232/1.12252

ISSP Research Group (2009): International Social Survey Programme: Health and Health Care - ISSP 2011. GESIS Data Archive, Cologne. ZA5800 Data file version 3.0.0, doi:10.4232/1.12252

ISSP Research Group (2009): International Social Survey Programme: Health and Health Care - ISSP 2011. GESIS Data Archive, Cologne. ZA5800 Data file version 3.0.0, doi:10.4232/1.12252

ISSP Research Group (2009): International Social Survey Programme: Health and Health Care - ISSP 2011. GESIS Data Archive, Cologne. ZA5800 Data file version 3.0.0, doi:10.4232/1.12252

ISSP Research Group (2009): International Social Survey Programme: Health and Health Care - ISSP 2011. GESIS Data Archive, Cologne. ZA5800 Data file version 3.0.0, doi:10.4232/1.12252

ISSP Research Group (2009): International Social Survey Programme: Health and Health Care - ISSP 2011. GESIS Data Archive, Cologne. ZA5800 Data file version 3.0.0, doi:10.4232/1.12252

ISSP Research Group (2009): International Social Survey Programme: Health and Health Care - ISSP 2011. GESIS Data Archive, Cologne. ZA5800 Data file version 3.0.0, doi:10.4232/1.12252

ISSP Research Group (2009): International Social Survey Programme: Health and Health Care - ISSP 2011. GESIS Data Archive, Cologne. ZA5800 Data file version 3.0.0, doi:10.4232/1.12252

Italy Aspects of Daily Life 1998 as it appears in P.N. Lee Statistics and Computing Ltd. International Mortality and Smoking Statistics Version 4.04. Sutton, United Kingdom: P.N. Lee Statistics and Computing Ltd, 2009

Italy Aspects of Daily Life 1999 as it appears in P.N. Lee Statistics and Computing Ltd. International Mortality and Smoking Statistics Version 4.04. Sutton, United Kingdom: P.N. Lee Statistics and Computing Ltd, 2009

Italy Aspects of Daily Life 2000 as it appears in P.N. Lee Statistics and Computing Ltd. International Mortality and Smoking Statistics Version 4.04. Sutton, United Kingdom: P.N. Lee Statistics and Computing Ltd, 2009

Italy Aspects of Daily Life 2001 as it appears in P.N. Lee Statistics and Computing Ltd. International Mortality and Smoking Statistics Version 4.04. Sutton, United Kingdom: P.N. Lee Statistics and Computing Ltd, 2009

Italy Aspects of Daily Life 2002 as it appears in P.N. Lee Statistics and Computing Ltd. International Mortality and Smoking Statistics Version 4.04. Sutton, United Kingdom: P.N. Lee Statistics and Computing Ltd, 2009

Italy Aspects of Daily Life 2005 as it appears in P.N. Lee Statistics and Computing Ltd. International Mortality and Smoking Statistics Version 4.04. Sutton, United Kingdom: P.N. Lee Statistics and Computing Ltd, 2009

Italy Aspects of Daily Life 2006 as it appears in P.N. Lee Statistics and Computing Ltd. International Mortality and Smoking Statistics Version 4.04. Sutton, United Kingdom: P.N. Lee Statistics and Computing Ltd, 2009

Italy Aspects of Daily Life 2007 as it appears in P.N. Lee Statistics and Computing Ltd. International Mortality and Smoking Statistics Version 4.04. Sutton, United Kingdom: P.N. Lee Statistics and Computing Ltd, 2009

Italy Aspects of Daily Life 2008 as it appears in P.N. Lee Statistics and Computing Ltd. International Mortality and Smoking Statistics Version 4.04. Sutton, United Kingdom: P.N. Lee Statistics and Computing Ltd, 2009

Italy Behavioral Risk Factor Surveillance System 2009 as it appears in P.N. Lee Statistics and Computing Ltd. International Mortality and Smoking Statistics Version 4.04. Sutton, United Kingdom: P.N. Lee Statistics and Computing Ltd, 2009

Italy Eurobarometer 32: The Single European Market, Drugs, Alcohol, and Cancer 1989 as it appears in P.N. Lee Statistics and Computing Ltd. International Mortality and Smoking Statistics Version 4.04. Sutton, United Kingdom: P.N. Lee Statistics and Computing Ltd, 2009

Italy Eurobarometer 38: European Court of Justice, Passive Smoking, And Consumer Issues 1992 as it appears in P.N. Lee Statistics and Computing Ltd. International Mortality and Smoking Statistics Version 4.04. Sutton, United Kingdom: P.N. Lee Statistics and Computing Ltd, 2009

Italy National Health Survey 1980 as it appears in P.N. Lee Statistics and Computing Ltd. International Mortality and Smoking Statistics Version 4.04. Sutton, United Kingdom: P.N. Lee Statistics and Computing Ltd, 2009

Italy National Health Survey 1987 as it appears in P.N. Lee Statistics and Computing Ltd. International Mortality and Smoking Statistics Version 4.04. Sutton, United Kingdom: P.N. Lee Statistics and Computing Ltd, 2009

Italy National Health Survey 1991 as it appears in P.N. Lee Statistics and Computing Ltd. International Mortality and Smoking Statistics Version 4.04. Sutton, United Kingdom: P.N. Lee Statistics and Computing Ltd, 2009

Italy National Health Survey 1994 as it appears in P.N. Lee Statistics and Computing Ltd. International Mortality and Smoking Statistics Version 4.04. Sutton, United Kingdom: P.N. Lee Statistics and Computing Ltd, 2009

Italy Tobacco Use Survey 1987 as it appears in P.N. Lee Statistics and Computing Ltd. International Mortality and Smoking Statistics Version 4.04. Sutton, United Kingdom: P.N. Lee Statistics and Computing Ltd, 2009

Italy Tobacco Use Survey 1990 as it appears in P.N. Lee Statistics and Computing Ltd. International Mortality and Smoking Statistics Version 4.04. Sutton, United Kingdom: P.N. Lee Statistics and Computing Ltd, 2009

Italy Tobacco Use Survey 2001 as it appears in P.N. Lee Statistics and Computing Ltd. International Mortality and Smoking Statistics Version 4.04. Sutton, United Kingdom: P.N. Lee Statistics and Computing Ltd, 2009

Italy Tobacco Use Survey 2002 as it appears in P.N. Lee Statistics and Computing Ltd. International Mortality and Smoking Statistics Version 4.04. Sutton, United Kingdom: P.N. Lee Statistics and Computing Ltd, 2009

Italy Tobacco Use Survey 2003 as it appears in P.N. Lee Statistics and Computing Ltd. International Mortality and Smoking Statistics Version 4.04. Sutton, United Kingdom: P.N. Lee Statistics and Computing Ltd, 2009

Italy Tobacco Use Survey 2004 as it appears in P.N. Lee Statistics and Computing Ltd. International Mortality and Smoking Statistics Version 4.04. Sutton, United Kingdom: P.N. Lee Statistics and Computing Ltd, 2009

Italy Tobacco Use Survey 2005 as it appears in P.N. Lee Statistics and Computing Ltd. International Mortality and Smoking Statistics Version 4.04. Sutton, United Kingdom: P.N. Lee Statistics and Computing Ltd, 2009

Japan Smoking Rate Survey 1980 as it appears in P.N. Lee Statistics and Computing Ltd. International Mortality and Smoking Statistics Version 4.04. Sutton, United Kingdom: P.N. Lee Statistics and Computing Ltd, 2009

Japan Smoking Rate Survey 1981 as it appears in P.N. Lee Statistics and Computing Ltd. International Mortality and Smoking Statistics Version 4.04. Sutton, United Kingdom: P.N. Lee Statistics and Computing Ltd, 2009

Japan Smoking Rate Survey 1982 as it appears in P.N. Lee Statistics and Computing Ltd. International Mortality and Smoking Statistics Version 4.04. Sutton, United Kingdom: P.N. Lee Statistics and Computing Ltd, 2009

Japan Smoking Rate Survey 1983 as it appears in P.N. Lee Statistics and Computing Ltd. International Mortality and Smoking Statistics Version 4.04. Sutton, United Kingdom: P.N. Lee Statistics and Computing Ltd, 2009

Japan Smoking Rate Survey 1984 as it appears in P.N. Lee Statistics and Computing Ltd. International Mortality and Smoking Statistics Version 4.04. Sutton, United Kingdom: P.N. Lee Statistics and Computing Ltd, 2009

Japan Smoking Rate Survey 1985 as it appears in P.N. Lee Statistics and Computing Ltd. International Mortality and Smoking Statistics Version 4.04. Sutton, United Kingdom: P.N. Lee Statistics and Computing Ltd, 2009

Japan Smoking Rate Survey 1986 as it appears in P.N. Lee Statistics and Computing Ltd. International Mortality and Smoking Statistics Version 4.04. Sutton, United Kingdom: P.N. Lee Statistics and Computing Ltd, 2009

Japan Smoking Rate Survey 1987 as it appears in P.N. Lee Statistics and Computing Ltd. International Mortality and Smoking Statistics Version 4.04. Sutton, United Kingdom: P.N. Lee Statistics and Computing Ltd, 2009

Japan Smoking Rate Survey 1988 as it appears in P.N. Lee Statistics and Computing Ltd. International Mortality and Smoking Statistics Version 4.04. Sutton, United Kingdom: P.N. Lee Statistics and Computing Ltd, 2009

Japan Smoking Rate Survey 1989 as it appears in P.N. Lee Statistics and Computing Ltd. International Mortality and Smoking Statistics Version 4.04. Sutton, United Kingdom: P.N. Lee Statistics and Computing Ltd, 2009

Japan Smoking Rate Survey 1990 as it appears in P.N. Lee Statistics and Computing Ltd. International Mortality and Smoking Statistics Version 4.04. Sutton, United Kingdom: P.N. Lee Statistics and Computing Ltd, 2009

Japan Smoking Rate Survey 1991 as it appears in P.N. Lee Statistics and Computing Ltd. International Mortality and Smoking Statistics Version 4.04. Sutton, United Kingdom: P.N. Lee Statistics and Computing Ltd, 2009

Japan Smoking Rate Survey 1992 as it appears in P.N. Lee Statistics and Computing Ltd. International Mortality and Smoking Statistics Version 4.04. Sutton, United Kingdom: P.N. Lee Statistics and Computing Ltd, 2009

Japan Smoking Rate Survey 1993 as it appears in P.N. Lee Statistics and Computing Ltd. International Mortality and Smoking Statistics Version 4.04. Sutton, United Kingdom: P.N. Lee Statistics and Computing Ltd, 2009

Japan Smoking Rate Survey 1994 as it appears in P.N. Lee Statistics and Computing Ltd. International Mortality and Smoking Statistics Version 4.04. Sutton, United Kingdom: P.N. Lee Statistics and Computing Ltd, 2009

Japan Smoking Rate Survey 1995 as it appears in P.N. Lee Statistics and Computing Ltd. International Mortality and Smoking Statistics Version 4.04. Sutton, United Kingdom: P.N. Lee Statistics and Computing Ltd, 2009

Japan Smoking Rate Survey 1996 as it appears in P.N. Lee Statistics and Computing Ltd. International Mortality and Smoking Statistics Version 4.04. Sutton, United Kingdom: P.N. Lee Statistics and Computing Ltd, 2009

Japan Smoking Rate Survey 1997 as it appears in P.N. Lee Statistics and Computing Ltd. International Mortality and Smoking Statistics Version 4.04. Sutton, United Kingdom: P.N. Lee Statistics and Computing Ltd, 2009

Japan Smoking Rate Survey 1998 as it appears in P.N. Lee Statistics and Computing Ltd. International Mortality and Smoking Statistics Version 4.04. Sutton, United Kingdom: P.N. Lee Statistics and Computing Ltd, 2009

Japan Smoking Rate Survey 1999 as it appears in P.N. Lee Statistics and Computing Ltd. International Mortality and Smoking Statistics Version 4.04. Sutton, United Kingdom: P.N. Lee Statistics and Computing Ltd, 2009

Japan Smoking Rate Survey 2000 as it appears in P.N. Lee Statistics and Computing Ltd. International Mortality and Smoking Statistics Version 4.04. Sutton, United Kingdom: P.N. Lee Statistics and Computing Ltd, 2009

Japan Smoking Rate Survey 2001 as it appears in P.N. Lee Statistics and Computing Ltd. International Mortality and Smoking Statistics Version 4.04. Sutton, United Kingdom: P.N. Lee Statistics and Computing Ltd, 2009

Japan Smoking Rate Survey 2002 as it appears in P.N. Lee Statistics and Computing Ltd. International Mortality and Smoking Statistics Version 4.04. Sutton, United Kingdom: P.N. Lee Statistics and Computing Ltd, 2009

Japan Smoking Rate Survey 2003 as it appears in P.N. Lee Statistics and Computing Ltd. International Mortality and Smoking Statistics Version 4.04. Sutton, United Kingdom: P.N. Lee Statistics and Computing Ltd, 2009

Japan Smoking Rate Survey 2004 as it appears in P.N. Lee Statistics and Computing Ltd. International Mortality and Smoking Statistics Version 4.04. Sutton, United Kingdom: P.N. Lee Statistics and Computing Ltd, 2009

Japan Smoking Rate Survey 2005 as it appears in P.N. Lee Statistics and Computing Ltd. International Mortality and Smoking Statistics Version 4.04. Sutton, United Kingdom: P.N. Lee Statistics and Computing Ltd, 2009

Japan Smoking Rate Survey 2006 as it appears in P.N. Lee Statistics and Computing Ltd. International Mortality and Smoking Statistics Version 4.04. Sutton, United Kingdom: P.N. Lee Statistics and Computing Ltd, 2009

Jee SH, Samet JM, Ohrr H, Kim JH, Kim IS. Smoking and cancer risk in Korean men and women. Cancer Causes Control. 2004; 15(4): 341–8

Jee SH, Sull JW, Park J, Lee SY, Ohrr H, Guallar E, Samet JM. Body-mass index and mortality in Korean men and women. N Engl J Med. 2006; 355(8): 779-87 as it appears in Thun MJ, Hannan LM, Adams-Campbell LL, Boffetta P, Buring JE, Feskanich D, Flanders WD, Jee SH, Katanoda K, Kolonel LN, Lee IM, Marugame T, Palmer JR, Riboli E, Sobue T, Avila-Tang E, Wilkens LR, Samet JM. Lung cancer occurrence in never-smokers: an analysis of 13 cohorts and 22 cancer registry studies. PLoS Med. 2008; 5(9): e185

Joint Health Surveys Unit of Social and Community Planning Research and University College London, Health Survey for England, 1994 [computer file]. 4th ed. Colchester, Essex: UK Data Archive [distributor], 26 March 2001. SN: 3640

Joint Health Surveys Unit of Social and Community Planning Research and University College London, Health Survey for England, 1995 [computer file]. 3rd ed. Colchester, Essex: UK Data Archive [distributor], 26 March 2001. SN: 3796

Joint Health Surveys Unit of Social and Community Planning Research and University College London, Health Survey for England, 1996 [computer file]. 3rd ed. Colchester, Essex: UK Data Archive [distributor], March 2001. SN: 3886

Joint Health Surveys Unit of Social and Community Planning Research and University College London, Health Survey for England, 1997 [computer file]. 2nd ed. Colchester, Essex: UK Data Archive [distributor], 4 December 2000. SN: 3979

Kark JD, Laor A. Cigarette smoking and educational level among young Israelis upon release from military service in 1988--a public health challenge. Isr J Med Sci. 1992; 28(1): 33-7 as it appears in P.N. Lee Statistics and Computing Ltd. International Mortality and Smoking Statistics Version 4.04. Sutton, United Kingdom: P.N. Lee Statistics and Computing Ltd, 2009

Karolinska Institute, Statistics Sweden, Stockholm County Council. Sweden - Stockholm Public Health Survey 2002-2003

Karolinska Institute, Statistics Sweden, Stockholm County Council. Sweden - Stockholm Public Health Survey 2006-2007

Karolinska Institute, Statistics Sweden, Stockholm County Council. Sweden - Stockholm Public Health Survey 2010

Karolinska Institute. Sweden - Stockholm Proportion of Daily Smokers in Stockholm County by Sex, Age, and Year 1990-2010

Kessler, Ronald C. National Comorbidity Survey: Baseline (NCS-1), 1990-1992. ICPSR06693-v6. Ann Arbor, MI: Inter-university Consortium for Political and Social Research [distributor], 2008-09-12. http://doi.org/10.3886/ICPSR06693.v6

Kokkevi A, Loukadakis M, Plagianakou S, Politikou K, Stefanis C. Sharp increase in illicit drug use in Greece: trends from a general population survey on licit and illicit drug use. Eur Addict Res. 2000; 6(1): 42–9

Kolonel LN, Henderson BE, Hankin JH, Nomura AM, Wilkens LR, Pike MC, Stram DO, Monroe KR, Earle ME, Nagamine FS. A multiethnic cohort in Hawaii and Los Angeles: baseline characteristics. Am J Epidemiol. 2000; 151(4): 346-57 as it appears in Thun MJ, Hannan LM, Adams-Campbell LL, Boffetta P, Buring JE,

Feskanich D, Flanders WD, Jee SH, Katanoda K, Kolonel LN, Lee IM, Marugame T, Palmer JR, Riboli E, Sobue T, Avila-Tang E, Wilkens LR, Samet JM. Lung cancer occurrence in never-smokers: an analysis of 13 cohorts and 22 cancer registry studies. PLoS Med. 2008; 5(9): e185

Korea Centers for Disease Control and Prevention. South Korea National Health and Nutrition Examination Survey 1998

Korea Centers for Disease Control and Prevention. South Korea National Health and Nutrition Examination Survey 2001

Korea Centers for Disease Control and Prevention. South Korea National Health and Nutrition Examination Survey 2005

Korea Centers for Disease Control and Prevention. South Korea National Health and Nutrition Examination Survey 2011

Korea Centers for Disease Control and Prevention. South Korea National Health and Nutrition Examination Survey Tobacco Use Prevalence 2005-2014

Korea Centers for Disease Control and Prevention. South Korea Youth Risk Behavior Web-Based Survey 2012

Kurihama Alcoholism Center (Japan), National Hospital Organization (Japan). Japan Survey on Adult Drinking Patterns and Prevention for Related Problems 2003

La Vecchia C, Levi F, Decarli A, Wietlisbach V, Negri E, Gutzwiller F. Trends in smoking and lung cancer mortality in Switzerland. Prev Med. 1988; 17(6): 712–24

LINK Institute for Market and Social Research (Switzerland), University of Zurich. Switzerland Survey on Smoking 2001-2002

LINK Institute for Market and Social Research (Switzerland), University of Zurich. Tobacco Consumption in the Swiss Population in the Years 2001-2007. 2008

Ludwig Boltzmann Institute for Addiction Research. Austria Sample Survey on Substance Use 2004

Macdonald SM, Reeder BA, Chen Y, Després JP. Obesity in Canada: a descriptive analysis. CMAJ. 1997; S3–9

Marugame T, Sobue T, Satoh H, Komatsu S, Nishino Y, Nakatsuka H, Nakayama T, Suzuki T, Takezaki T, Tajima K, Tominaga S. Lung cancer death rates by smoking status: comparison of the Three-Prefecture Cohort study in Japan to the Cancer Prevention Study II in the USA. Cancer Sci. 2005; 96(2): 120-6 as it appears in Thun MJ, Hannan LM, Adams-Campbell LL, Boffetta P, Buring JE, Feskanich D, Flanders WD, Jee SH, Katanoda K, Kolonel LN, Lee IM, Marugame T, Palmer JR, Riboli E, Sobue T, Avila-Tang E, Wilkens LR, Samet JM. Lung cancer occurrence in never-smokers: an analysis of 13 cohorts and 22 cancer registry studies. PLoS Med. 2008; 5(9): e185

Medicine: Statistics of Smoking in the Member States of the European Community as it appears in P.N. Lee Statistics and Computing Ltd. International Mortality and Smoking Statistics Version 4.04. Sutton, United Kingdom: P.N. Lee Statistics and Computing Ltd, 2009

Milias GA, Panagiotakos DB, Pitsavos C, Xenaki D, Panagopoulos G, Stefanadis C. Prevalence of self-reported hypercholesterolaemia and its relation to dietary habits, in Greek adults; a national nutrition & health survey. Lipids Health Dis. 2006; 5(1): 1–7

Ministry of Education and Research (Norway). Baltic Sea Regional Study on Adolescents' Sexuality 2003-2004. Oslo, Norway: Ministry of Education and Research (Norway), 2007

Ministry of Health (Argentina), National Institute of Statistics and Censuses (Argentina). Argentina National Survey of Risk Factors 2009

Ministry of Health (Chile), National Institute of Statistics (Chile). Chile National Quality of Life and Health Survey 2000. Santiago, Chile: Ministry of Health (Chile)

Ministry of Health (Chile), National Institute of Statistics (Chile). Chile National Quality of Life and Health Survey 2006. Santiago, Chile: Ministry of Health (Chile)

Ministry of Health (Chile). Chile National Health Survey 2009-2010

Ministry of Health (Italy), National Association of Hospital Cardiologists (Italy), National Institute of Health (Italy). Italy Cardiovascular Epidemiologic Observatory 1998-2002

Ministry of Health (New Zealand), National Research Bureau Ltd (New Zealand), Statistics New Zealand. New Zealand Health Survey 2002-2004

Ministry of Health (New Zealand), National Research Bureau Ltd (New Zealand). New Zealand Health Survey 2006-2007

Ministry of Health (New Zealand). New Zealand Tobacco Facts 1981-2001

Ministry of Health (New Zealand). New Zealand Tobacco Use Survey 2006

Ministry of Health (New Zealand). New Zealand Tobacco Use Survey 2008

Ministry of Health (New Zealand). New Zealand Tobacco Use Survey 2009

Ministry of Health (Singapore). Singapore National Health Surveillance Survey 2001

Ministry of Health (Singapore). Singapore National Health Surveillance Survey 2007

Ministry of Health (Singapore). Singapore National Health Survey 1998

Ministry of Health (Singapore). Singapore National Health Survey 2004

Ministry of Health (Singapore). Singapore National Health Survey 2010

Ministry of Health (Singapore). Singapore State of Health 2000. Singapore, Singapore: Ministry of Health (Singapore), 2001

Ministry of Health and Welfare (Andorra). Andorra National Health Survey 2002

Ministry of Health and Welfare (Andorra). Andorra National Health Survey 2011

Ministry of Health and Welfare (Japan), National Institute of Health and Nutrition (Japan). Japan National Nutrition Survey 1999

Ministry of Health and Welfare (Japan). Japan National Nutrition Survey 1986

Ministry of Health and Welfare (Japan). Japan National Nutrition Survey 1989

Ministry of Health and Welfare (Japan). Japan National Nutrition Survey 1990

Ministry of Health and Welfare (Japan). Japan National Nutrition Survey 1991

Ministry of Health and Welfare (Japan). Japan National Nutrition Survey 1993

Ministry of Health and Welfare (Japan). Japan National Nutrition Survey 1994

Ministry of Health and Welfare (Japan). Japan National Nutrition Survey 1995

Ministry of Health and Welfare (Japan). Japan National Nutrition Survey 1996

Ministry of Health and Welfare (Japan). Japan National Nutrition Survey 1997

Ministry of Health and Welfare (Japan). Japan National Nutrition Survey 1998

Ministry of Health and Welfare (Japan). Japan National Nutrition Survey 2000

Ministry of Health and Welfare (Japan). Japan National Nutrition Survey 2001

Ministry of Health and Welfare (Japan). Japan National Survey on Smoking and Health 1999

Ministry of Health and Welfare (South Korea). South Korea National Health Survey 1989

Ministry of Health and Welfare (South Korea). South Korea National Health Survey 1995

Ministry of Health, Labour and Welfare (Japan), National Institute of Health and Nutrition (Japan). Japan National Health and Nutrition Survey 2012

Ministry of Health, Labour and Welfare (Japan). Japan National Health and Nutrition Survey 2003

Ministry of Health, Labour and Welfare (Japan). Japan National Health and Nutrition Survey 2004

Ministry of Health, Labour and Welfare (Japan). Japan National Health and Nutrition Survey 2005

Ministry of Health, Labour and Welfare (Japan). Japan National Health and Nutrition Survey 2006

Ministry of Health, Labour and Welfare (Japan). Japan National Health and Nutrition Survey 2007

Ministry of Health, Labour and Welfare (Japan). Japan National Health and Nutrition Survey 2008

Ministry of Health, Labour and Welfare (Japan). Japan National Health and Nutrition Survey 2009

Ministry of Health, Labour and Welfare (Japan). Japan National Health and Nutrition Survey 2013

Ministry of Health, Labour and Welfare (Japan). Japan National Nutrition Survey 2002

Ministry of Health, Labour and Welfare (Japan). Japan National Survey of Cardiovascular Diseases 2000

Ministry of Health, Social Services and Equality (Spain), National Statistics Institute (Spain). Spain National Health Survey 2011-2012

Ministry of Health, Social Services and Equality (Spain). Spain Annual Report on the National Health System 2006. Madrid, Spain: Ministry of Health, Social Services and Equality (Spain)

Ministry of Health, Social Services and Equality (Spain). Spain National Health Survey 1987

Ministry of Health, Social Services and Equality (Spain). Spain National Health Survey 1993

Ministry of Health, Social Services and Equality (Spain). Spain National Health Survey 1995

Ministry of Health, Social Services and Equality (Spain). Spain National Health Survey 1997

Ministry of Health, Social Services and Equality (Spain). Spain National Health Survey 2001

Ministry of Health, Welfare, and Sport (Netherlands), Netherlands Institute for Social Research (SCP), Statistics Netherlands. Netherlands Permanent Quality of Life Survey 1998

Ministry of Public Health (Uruguay), World Health Organization (WHO). Uruguay STEPS Noncommunicable Disease Risk Factors Survey 2006

NatCen Social Research and Royal Free and University College Medical School. Department of Epidemiology and Public Health, Health Survey for England, 2010 [computer file]. 2nd Edition. Colchester, Essex: UK Data Archive [distributor], July 2012. SN: 6986, http://dx.doi.org/10.5255/UKDA-SN-6986-2

NatCen Social Research and University College London. Department of Epidemiology and Public Health, Health Survey for England, 2011 [computer file]. Colchester, Essex: UK Data Archive [distributor], April 2013. SN: 7260, http://dx.doi.org/10.5255/UKDA-SN-7260-1

NatCen Social Research and University College London. Department of Epidemiology and Public Health, Health Survey for England, 2012 [computer file]. Colchester, Essex: UK Data Archive [distributor], April 2014. SN: 7480, http://dx.doi.org/10.5255/UKDA-SN-7480-1

National Board of Health and Welfare (Sweden). Health in Sweden - The National Public Health Report 2001. Scand J Public Health Suppl. 2001; 29(Suppl 58)

National Board of Health and Welfare (Sweden). Sweden Young People's Tobacco Use, Knowledge, and Attitudes Survey 1987

National Board of Health and Welfare (Sweden). Sweden Young People's Tobacco Use, Knowledge, and Attitudes Survey 1994

National Center for Health Statistics (NCHS), Centers for Disease Control and Prevention (CDC), Statistics Canada. Canada and United States Joint Survey of Health 2002-2003. Ottawa, Canada: Statistics Canada

National Center for Health Statistics (NCHS), Centers for Disease Control and Prevention (CDC), United States Census Bureau. United States National Health Interview Survey 2012. Hyattsville, United States: National Center for Health Statistics (NCHS), Centers for Disease Control and Prevention (CDC), 2013

National Center for Health Statistics (NCHS), Centers for Disease Control and Prevention (CDC), United States Census Bureau. United States National Health Interview Survey 2013. Hyattsville, United States: National Center for Health Statistics (NCHS), Centers for Disease Control and Prevention (CDC), 2014

National Center for Health Statistics (NCHS), Centers for Disease Control and Prevention (CDC), US Census Bureau. United States National Health Interview Survey 1983. Hyattville, United States: National Center for Health Statistics (NCHS), Centers for Disease Control and Prevention (CDC)

National Center for Health Statistics (NCHS), Centers for Disease Control and Prevention (CDC), US Census Bureau. United States National Health Interview Survey 1985. Hyattsville, United States: National Center for Health Statistics (NCHS), Centers for Disease Control and Prevention (CDC)

National Center for Health Statistics (NCHS), Centers for Disease Control and Prevention (CDC), US Census Bureau. United States National Health Interview Survey 1990. Hyattsville, United States: National Center for Health Statistics (NCHS), Centers for Disease Control and Prevention (CDC)

National Center for Health Statistics (NCHS), Centers for Disease Control and Prevention (CDC), US Census Bureau. United States National Health Interview Survey 1991. Hyattsville, United States: National Center for Health Statistics (NCHS), Centers for Disease Control and Prevention (CDC)

National Center for Health Statistics (NCHS), Centers for Disease Control and Prevention (CDC), US Census Bureau. United States National Health Interview Survey 1992. Hyattsville, United States: National Center for Health Statistics (NCHS), Centers for Disease Control and Prevention (CDC)

National Center for Health Statistics (NCHS), Centers for Disease Control and Prevention (CDC), US Census Bureau. United States National Health Interview Survey 1993. Hyattville, United States: National Center for Health Statistics (NCHS), Centers for Disease Control and Prevention (CDC)

National Center for Health Statistics (NCHS), Centers for Disease Control and Prevention (CDC), US Census Bureau. United States National Health Interview Survey 1994. Hyattsville, United States: National Center for Health Statistics (NCHS), Centers for Disease Control and Prevention (CDC)

National Center for Health Statistics (NCHS), Centers for Disease Control and Prevention (CDC), US Census Bureau. United States National Health Interview Survey 1995. Hyattsville, United States: National Center for Health Statistics (NCHS), Centers for Disease Control and Prevention (CDC)

National Center for Health Statistics (NCHS), Centers for Disease Control and Prevention (CDC), US Census Bureau. United States National Health Interview Survey 1997. Hyattsville, United States: National Center for Health Statistics (NCHS), Centers for Disease Control and Prevention (CDC)

National Center for Health Statistics (NCHS), Centers for Disease Control and Prevention (CDC), US Census Bureau. United States National Health Interview Survey 1998. Hyattsville, United States: National Center for Health Statistics (NCHS), Centers for Disease Control and Prevention (CDC)

National Center for Health Statistics (NCHS), Centers for Disease Control and Prevention (CDC), US Census Bureau. United States National Health Interview Survey 1999. Hyattsville, United States: National Center for Health Statistics (NCHS), Centers for Disease Control and Prevention (CDC)

National Center for Health Statistics (NCHS), Centers for Disease Control and Prevention (CDC), US Census Bureau. United States National Health Interview Survey 2000. Hyattsville, United States: National Center for Health Statistics (NCHS), Centers for Disease Control and Prevention (CDC)

National Center for Health Statistics (NCHS), Centers for Disease Control and Prevention (CDC), US Census Bureau. United States National Health Interview Survey 2001. Hyattsville, United States: National Center for Health Statistics (NCHS), Centers for Disease Control and Prevention (CDC)

National Center for Health Statistics (NCHS), Centers for Disease Control and Prevention (CDC), US Census Bureau. United States National Health Interview Survey 2002. Hyattsville, United States: National Center for Health Statistics (NCHS), Centers for Disease Control and Prevention (CDC)

National Center for Health Statistics (NCHS), Centers for Disease Control and Prevention (CDC), US Census Bureau. United States National Health Interview Survey 2003. Hyattsville, United States: National Center for Health Statistics (NCHS), Centers for Disease Control and Prevention (CDC)

National Center for Health Statistics (NCHS), Centers for Disease Control and Prevention (CDC), US Census Bureau. United States National Health Interview Survey 2005. Hyattsville, United States: National Center for Health Statistics (NCHS), Centers for Disease Control and Prevention (CDC)

National Center for Health Statistics (NCHS), Centers for Disease Control and Prevention (CDC), US Census Bureau. United States National Health Interview Survey 2006. Hyattsville, United States: National Center for Health Statistics (NCHS), Centers for Disease Control and Prevention (CDC)

National Center for Health Statistics (NCHS), Centers for Disease Control and Prevention (CDC), US Census Bureau. United States National Health Interview Survey 2007. Hyattsville, United States: National Center for Health Statistics (NCHS), Centers for Disease Control and Prevention (CDC)

National Center for Health Statistics (NCHS), Centers for Disease Control and Prevention (CDC), US Census Bureau. United States National Health Interview Survey 2008. Hyattsville, United States: National Center for Health Statistics (NCHS), Centers for Disease Control and Prevention (CDC)

National Center for Health Statistics (NCHS), Centers for Disease Control and Prevention (CDC), US Census Bureau. United States National Health Interview Survey 2009. Hyattsville, United States: National Center for Health Statistics (NCHS), Centers for Disease Control and Prevention (CDC)

National Center for Health Statistics (NCHS), Centers for Disease Control and Prevention (CDC), US Census Bureau. United States National Health Interview Survey 2010. Hyattsville, United States: National Center for Health Statistics (NCHS), Centers for Disease Control and Prevention (CDC)

National Center for Health Statistics (NCHS), Centers for Disease Control and Prevention (CDC), US Census Bureau. United States National Health Interview Survey 2011. Hyattsville, United States: National Center for Health Statistics (NCHS), Centers for Disease Control and Prevention (CDC)

National Center for Health Statistics (NCHS), Centers for Disease Control and Prevention (CDC). United States National Health and Nutrition Examination Survey 1988-1994. Hyattsville, United States: National Center for Health Statistics (NCHS), Centers for Disease Control and Prevention (CDC)

National Center for Health Statistics (NCHS), Centers for Disease Control and Prevention (CDC). United States National Health and Nutrition Examination Survey 1999-2000. Hyattsville, United States: National Center for Health Statistics (NCHS), Centers for Disease Control and Prevention (CDC)

National Center for Health Statistics (NCHS), Centers for Disease Control and Prevention (CDC). United States National Health and Nutrition Examination Survey 2001-2002. Hyattsville, United States: National Center for Health Statistics (NCHS), Centers for Disease Control and Prevention (CDC)

National Center for Health Statistics (NCHS), Centers for Disease Control and Prevention (CDC). United States National Health and Nutrition Examination Survey 2003-2004. Hyattsville, United States: National Center for Health Statistics (NCHS), Centers for Disease Control and Prevention (CDC)

National Center for Health Statistics (NCHS), Centers for Disease Control and Prevention (CDC). United States National Health and Nutrition Examination Survey 2005-2006. Hyattsville, United States: National Center for Health Statistics (NCHS), Centers for Disease Control and Prevention (CDC), 2007

National Center for Health Statistics (NCHS), Centers for Disease Control and Prevention (CDC). United States National Health and Nutrition Examination Survey 2007-2008. Hyattsville, United States: National Center for Health Statistics (NCHS), Centers for Disease Control and Prevention (CDC), 2009

National Center for Health Statistics (NCHS), Centers for Disease Control and Prevention (CDC). United States National Health and Nutrition Examination Survey 2009-2010. Hyattsville, United States: National Center for Health Statistics (NCHS), Centers for Disease Control and Prevention (CDC), 2011

National Center for Health Statistics (NCHS), Centers for Disease Control and Prevention (CDC). United States National Health and Nutrition Examination Survey 2011-2012. Hyattsville, United States: National Center for Health Statistics (NCHS), Centers for Disease Control and Prevention (CDC), 2013

National Centre for Social Research (NatCen). United Kingdom Health Survey for England Custom Covariates Tabulations 2001-2011 - Public Health England

National Centre for Social Research and University College London. Department of Epidemiology and Public Health, Health Survey for England, 2001 [computer file]. 2nd Edition. Colchester, Essex: UK Data Archive [distributor], June 2004. SN: 4628

National Centre for Social Research and University College London. Department of Epidemiology and Public Health, Health Survey for England, 2002 [computer file]. Colchester, Essex: UK Data Archive [distributor], May 2004. SN: 4912

National Centre for Social Research and University College London. Department of Epidemiology and Public Health, Health Survey for England, 2003 [computer file]. Colchester, Essex: UK Data Archive [distributor], March 2005. SN: 5098

National Centre for Social Research and University College London. Department of Epidemiology and Public Health, Health Survey for England, 2004 [computer file]. Colchester, Essex: UK Data Archive [distributor], July 2006. SN: 5439

National Centre for Social Research and University College London. Department of Epidemiology and Public Health, Health Survey for England, 2006 [computer file]. 4th Edition. Colchester, Essex: UK Data Archive [distributor], July 2011. SN: 5809, http://dx.doi.org/10.5255/UKDA-SN-5809-1

National Centre for Social Research and University College London. Department of Epidemiology and Public Health, Health Survey for England, 2007 [computer file]. 2nd Edition. Colchester, Essex: UK Data Archive [distributor], April 2010. SN: 6112, http://dx.doi.org/10.5255/UKDA-SN-6112-1

National Centre for Social Research and University College London. Department of Epidemiology and Public Health, Health Survey for England, 2008 [computer file]. 3rd Edition. Colchester, Essex: UK Data Archive [distributor], July 2011. SN: 6397, http://dx.doi.org/10.5255/UKDA-SN-6397-1

National Centre for Social Research and University College London. Department of Epidemiology and Public Health, Health Survey for England, 2009 [computer file]. 2nd Edition. Colchester, Essex: UK Data Archive [distributor], July 2011. SN: 6732, http://dx.doi.org/10.5255/UKDA-SN-6732-1

National Centre for Social Research and University College London. Department of Epidemiology and Public Health, Health Survey for England, 1999 [computer file]. 3rd Edition. Colchester, Essex: UK Data Archive [distributor], February 2002. SN: 4365

National Centre for Social Research and University College London. Department of Epidemiology and Public Health, Health Survey for England, 2005 [computer file]. Colchester, Essex: UK Data Archive [distributor], July 2007. SN: 5675

National Centre for Social Research and University of Leicester, Adult Psychiatric Morbidity Survey, 2007 [computer file]. 3rd Edition. Colchester, Essex: UK Data Archive [distributor], January 2011. SN: 6379, http://dx.doi.org/10.5255/UKDA-SN-6379-1

National Centre for Social Research et al. , National Survey of Sexual Attitudes and Lifestyles II, 2000-2001 [computer file]. Colchester, Essex: UK Data Archive [distributor], August 2005. SN: 5223, http://dx.doi.org/10.5255/UKDA-SN-5223-1

National Centre for Social Research, University College London Department of Epidemiology and Public Health, Health Survey for England, 1998 [computer file]. 4th ed. Colchester, Essex: UK Data Archive [distributor], 30 November 2002. SN: 4150

National Centre for Social Research, University College London Department of Epidemiology and Public Health, Health Survey for England, 2000 [computer file]. Colchester, Essex: UK Data Archive [distributor], 23 April 2002. SN: 4487

National Council for Narcotics Control (CONACE) (Chile). Chile National Drug Study 1996

National Council for Narcotics Control (CONACE) (Chile). Chile National Drug Study 1998-1999

National Council for Narcotics Control (CONACE) (Chile). Chile National Drug Study 2000

National Council for Narcotics Control (CONACE) (Chile). Chile National Drug Study 2006

National Hospital Organization (Japan). Japan National Survey on Underage Smoking and Drinking 1996

National Hospital Organization (Japan). Japan National Survey on Underage Smoking and Drinking 2001

National Hospital Organization (Japan). Japan National Survey on Underage Smoking and Drinking 2004

National Institute for Health and Welfare (Finland). Finland Health Behavior and Health Among the Adult Population 2008

National Institute for Health and Welfare (Finland). Finland Health Behavior and Health Among the Adult Population 2009

National Institute for Health and Welfare (Finland). Finland Health Behavior and Health Among the Finnish Elderly Population 2009

National Institute for Health and Welfare (Finland). Finland Health Behavior and Health Among the Finnish Elderly Population 2011

National Institute for Health and Welfare (Finland). Finland Tobacco Statistics 2011. Helsinki, Finland: National Institute for Health and Welfare (Finland), 2012

National Institute for Prevention and Health Education (France). France Health Barometer 2005. Paris, France: National Institute for Prevention and Health Education (France)

National Institute for Public Health and the Environment (Netherlands). Netherlands Measurement Survey 2009-2010

National Institute of Health (Italy). Italy Behavioral Risk Factor Surveillance System 2007

National Institute of Health (Italy). Italy Behavioral Risk Factor Surveillance System 2008

National Institute of Health (Italy). Italy Behavioral Risk Factor Surveillance System 2010

National Institute of Health (Italy). Italy Behavioral Risk Factor Surveillance System 2011

National Institute of Health (Italy). Italy Behavioral Risk Factor Surveillance System 2012

National Institute of Health (Italy). Italy Behavioral Risk Factor Surveillance System 2013

National Institute of Health (Portugal), Statistics Portugal. Portugal National Health Survey 2005-2006

National Institute of Public Health (Denmark). Denmark Health Interview Survey 1986-1987

National Institute of Public Health (Denmark). Denmark Health Interview Survey 1994

National Institute of Public Health (Denmark). Denmark Health Interview Survey 2000

National Institute of Public Health (Denmark). Denmark Health Interview Survey 2010

National Institute of Public Health (Denmark). Denmark National Health Examination Survey 2007-2008. Copenhagen, Denmark: National Institute of Public Health (Denmark)

National Institute of Public Health (Denmark). Greenland Population Health Survey 1993-1994

National Institute of Public Health (Denmark). Greenland Population Health Survey 1999-2001

National Institute of Public Health (Denmark). Greenland Population Health Survey 2005-2010

National Institute of Public Health (Denmark). Greenland Population Health Survey 2014

National Institute of Statistics (Italy). Italy Aspects of Daily Life 2003

National Institute of Statistics (Italy). Italy Smoking 2000

National Institute of Statistics (Italy). Italy Smoking 2001

National Institute of Statistics (Italy). Italy Smoking 2002

National Institute of Statistics (Italy). Italy Smoking 2003

National Institute of Statistics (Italy). Italy Smoking 2005

National Institute of Statistics (Italy). Italy Statistical Yearbook 2008. Rome, Italy: National Institute of Statistics (Italy), 2008

National Institute on Alcohol Abuse and Alcoholism (NIAAA), National Institutes of Health (NIH), U.S. Department of Health and Human Services. United States National Epidemiologic Survey on Alcohol and Related Conditions 2001-2002

National Institute on Alcohol Abuse and Alcoholism (NIAAA), National Institutes of Health (NIH), U.S. Department of Health and Human Services. United States National Epidemiologic Survey on Alcohol and Related Conditions 2004-2005

National Public Health Institute (Finland), Statistics Finland. Finland Health Examination Survey 2000-2001

National Public Health Institute (Finland). Finland Health Behavior and Health Among the Adult Population 1998

National Public Health Institute (Finland). Finland Health Behavior and Health Among the Adult Population 1999

National Public Health Institute (Finland). Finland Health Behavior and Health Among the Adult Population 2000

National Public Health Institute (Finland). Finland Health Behavior and Health Among the Adult Population 2001

National Public Health Institute (Finland). Finland Health Behavior and Health Among the Adult Population 2002

National Public Health Institute (Finland). Finland Health Behavior and Health Among the Adult Population 2003

National Public Health Institute (Finland). Finland Health Behavior and Health Among the Adult Population 2004

National Public Health Institute (Finland). Finland Health Behavior and Health Among the Adult Population 2005

National Public Health Institute (Finland). Finland Health Behavior and Health Among the Adult Population 2007

National Public Health Institute (Finland). Finland Health Behavior and Health Among the Elderly 2003

National Public Health Institute (Finland). Finland Health Behavior and Health Among the Finnish Elderly Population 2001

National Public Health Institute (Finland). Finland Health Behavior and Health Among the Finnish Elderly Population 2003

National Public Health Institute (Finland). Finland Health Behavior and Health Among the Finnish Elderly Population 2005

National Public Health Institute (Finland). Finland Health Behavior and Health Among the Finnish Elderly Population 2007

National Public Health Institute (Finland). Finland National FINRISK Health Survey 2007

National Research Bureau Ltd (New Zealand). New Zealand Environmental Tobacco Smoke Survey 1996

National Research Bureau Ltd (New Zealand). New Zealand Heart Health Behavior Survey 1989

National Research Bureau Ltd (New Zealand). New Zealand Heart Health Behavior Survey 1991

National Statistics Office (Malta). Malta Lifestyle Survey 2003

National Statistics Office (Malta). Malta Lifestyle Survey 2006-2007

National University of Tres de Febrero (Argentina), Secretariat for Programming Drug Abuse Prevention and the Fight against Drug Trafficking (SEDRONAR). Argentina National Psychoactive Substance Consumption Study 2008

National University of Tres de Febrero (Argentina), Secretariat for Programming Drug Abuse Prevention and the Fight against Drug Trafficking (SEDRONAR). Argentina National Psychoactive Substance Consumption Study 2010. Buenos Aires, Argentina: Argentina Observatory on Drugs

Netherlands Continuous Survey of Smoking Habits 1980 as it appears in P.N. Lee Statistics and Computing Ltd. International Mortality and Smoking Statistics Version 4.04. Sutton, United Kingdom: P.N. Lee Statistics and Computing Ltd, 2009

Netherlands Continuous Survey of Smoking Habits 1981 as it appears in P.N. Lee Statistics and Computing Ltd. International Mortality and Smoking Statistics Version 4.04. Sutton, United Kingdom: P.N. Lee Statistics and Computing Ltd, 2009

Netherlands Continuous Survey of Smoking Habits 1982 as it appears in P.N. Lee Statistics and Computing Ltd. International Mortality and Smoking Statistics Version 4.04. Sutton, United Kingdom: P.N. Lee Statistics and Computing Ltd, 2009

Netherlands Continuous Survey of Smoking Habits 1983 as it appears in P.N. Lee Statistics and Computing Ltd. International Mortality and Smoking Statistics Version 4.04. Sutton, United Kingdom: P.N. Lee Statistics and Computing Ltd, 2009

Netherlands Continuous Survey of Smoking Habits 1984 as it appears in P.N. Lee Statistics and Computing Ltd. International Mortality and Smoking Statistics Version 4.04. Sutton, United Kingdom: P.N. Lee Statistics and Computing Ltd, 2009

Netherlands Continuous Survey of Smoking Habits 1985 as it appears in P.N. Lee Statistics and Computing Ltd. International Mortality and Smoking Statistics Version 4.04. Sutton, United Kingdom: P.N. Lee Statistics and Computing Ltd, 2009

Netherlands Continuous Survey of Smoking Habits 1986 as it appears in P.N. Lee Statistics and Computing Ltd. International Mortality and Smoking Statistics Version 4.04. Sutton, United Kingdom: P.N. Lee Statistics and Computing Ltd, 2009

Netherlands Continuous Survey of Smoking Habits 1987 as it appears in P.N. Lee Statistics and Computing Ltd. International Mortality and Smoking Statistics Version 4.04. Sutton, United Kingdom: P.N. Lee Statistics and Computing Ltd, 2009

Netherlands Continuous Survey of Smoking Habits 1988 as it appears in P.N. Lee Statistics and Computing Ltd. International Mortality and Smoking Statistics Version 4.04. Sutton, United Kingdom: P.N. Lee Statistics and Computing Ltd, 2009

Netherlands Continuous Survey of Smoking Habits 1989 as it appears in P.N. Lee Statistics and Computing Ltd. International Mortality and Smoking Statistics Version 4.04. Sutton, United Kingdom: P.N. Lee Statistics and Computing Ltd, 2009

Netherlands Continuous Survey of Smoking Habits 1990 as it appears in P.N. Lee Statistics and Computing Ltd. International Mortality and Smoking Statistics Version 4.04. Sutton, United Kingdom: P.N. Lee Statistics and Computing Ltd, 2009

Netherlands Continuous Survey of Smoking Habits 1991 as it appears in P.N. Lee Statistics and Computing Ltd. International Mortality and Smoking Statistics Version 4.04. Sutton, United Kingdom: P.N. Lee Statistics and Computing Ltd, 2009

Netherlands Continuous Survey of Smoking Habits 1992 as it appears in P.N. Lee Statistics and Computing Ltd. International Mortality and Smoking Statistics Version 4.04. Sutton, United Kingdom: P.N. Lee Statistics and Computing Ltd, 2009

Netherlands Continuous Survey of Smoking Habits 1993 as it appears in P.N. Lee Statistics and Computing Ltd. International Mortality and Smoking Statistics Version 4.04. Sutton, United Kingdom: P.N. Lee Statistics and Computing Ltd, 2009

Netherlands Continuous Survey of Smoking Habits 1994 as it appears in P.N. Lee Statistics and Computing Ltd. International Mortality and Smoking Statistics Version 4.04. Sutton, United Kingdom: P.N. Lee Statistics and Computing Ltd, 2009

Netherlands Continuous Survey of Smoking Habits 1995 as it appears in P.N. Lee Statistics and Computing Ltd. International Mortality and Smoking Statistics Version 4.04. Sutton, United Kingdom: P.N. Lee Statistics and Computing Ltd, 2009

Netherlands Health Interview Survey 1989 as it appears in P.N. Lee Statistics and Computing Ltd. International Mortality and Smoking Statistics Version 4.04. Sutton, United Kingdom: P.N. Lee Statistics and Computing Ltd, 2009

Netherlands Health Interview Survey 1990 as it appears in P.N. Lee Statistics and Computing Ltd. International Mortality and Smoking Statistics Version 4.04. Sutton, United Kingdom: P.N. Lee Statistics and Computing Ltd, 2009

Netherlands Health Interview Survey 1991 as it appears in P.N. Lee Statistics and Computing Ltd. International Mortality and Smoking Statistics Version 4.04. Sutton, United Kingdom: P.N. Lee Statistics and Computing Ltd, 2009

Netherlands Health Interview Survey 1992 as it appears in P.N. Lee Statistics and Computing Ltd. International Mortality and Smoking Statistics Version 4.04. Sutton, United Kingdom: P.N. Lee Statistics and Computing Ltd, 2009

Netherlands Health Interview Survey 1993 as it appears in P.N. Lee Statistics and Computing Ltd. International Mortality and Smoking Statistics Version 4.04. Sutton, United Kingdom: P.N. Lee Statistics and Computing Ltd, 2009

Netherlands Health Interview Survey 1994 as it appears in P.N. Lee Statistics and Computing Ltd. International Mortality and Smoking Statistics Version 4.04. Sutton, United Kingdom: P.N. Lee Statistics and Computing Ltd, 2009

Netherlands Health Interview Survey 1995 as it appears in P.N. Lee Statistics and Computing Ltd. International Mortality and Smoking Statistics Version 4.04. Sutton, United Kingdom: P.N. Lee Statistics and Computing Ltd, 2009

Netherlands National School Survey on Substance Use 1983-1984 as it appears in P.N. Lee Statistics and Computing Ltd. International Mortality and Smoking Statistics Version 4.04. Sutton, United Kingdom: P.N. Lee Statistics and Computing Ltd, 2009

New South Wales Department of Health. Australia - New South Wales Health Behaviors 1997-2009. Sydney, Australia: New South Wales Department of Health

New Zealand Census 1981 as it appears in P.N. Lee Statistics and Computing Ltd. International Mortality and Smoking Statistics Version 4.04. Sutton, United Kingdom: P.N. Lee Statistics and Computing Ltd, 2009

New Zealand Exposure to Secondhand Cigarette Smoke Survey 2001 as it appears in P.N. Lee Statistics and Computing Ltd. International Mortality and Smoking Statistics Version 4.04. Sutton, United Kingdom: P.N. Lee Statistics and Computing Ltd, 2009

New Zealand Health Survey 1996-1997 as it appears in P.N. Lee Statistics and Computing Ltd. International Mortality and Smoking Statistics Version 4.04. Sutton, United Kingdom: P.N. Lee Statistics and Computing Ltd, 2009

New Zealand Omnibus Survey 1983 as it appears in P.N. Lee Statistics and Computing Ltd. International Mortality and Smoking Statistics Version 4.04. Sutton, United Kingdom: P.N. Lee Statistics and Computing Ltd, 2009

New Zealand Omnibus Survey 1984 as it appears in P.N. Lee Statistics and Computing Ltd. International Mortality and Smoking Statistics Version 4.04. Sutton, United Kingdom: P.N. Lee Statistics and Computing Ltd, 2009

New Zealand Omnibus Survey 1985 as it appears in P.N. Lee Statistics and Computing Ltd. International Mortality and Smoking Statistics Version 4.04. Sutton, United Kingdom: P.N. Lee Statistics and Computing Ltd, 2009

New Zealand Omnibus Survey 1986 as it appears in P.N. Lee Statistics and Computing Ltd. International Mortality and Smoking Statistics Version 4.04. Sutton, United Kingdom: P.N. Lee Statistics and Computing Ltd, 2009

New Zealand Omnibus Survey 1987 as it appears in P.N. Lee Statistics and Computing Ltd. International Mortality and Smoking Statistics Version 4.04. Sutton, United Kingdom: P.N. Lee Statistics and Computing Ltd, 2009

New Zealand Omnibus Survey 1988 as it appears in P.N. Lee Statistics and Computing Ltd. International Mortality and Smoking Statistics Version 4.04. Sutton, United Kingdom: P.N. Lee Statistics and Computing Ltd, 2009

New Zealand Omnibus Survey 1989 as it appears in P.N. Lee Statistics and Computing Ltd. International Mortality and Smoking Statistics Version 4.04. Sutton, United Kingdom: P.N. Lee Statistics and Computing Ltd, 2009

New Zealand Omnibus Survey 1990 as it appears in P.N. Lee Statistics and Computing Ltd. International Mortality and Smoking Statistics Version 4.04. Sutton, United Kingdom: P.N. Lee Statistics and Computing Ltd, 2009

New Zealand Omnibus Survey 1991 as it appears in P.N. Lee Statistics and Computing Ltd. International Mortality and Smoking Statistics Version 4.04. Sutton, United Kingdom: P.N. Lee Statistics and Computing Ltd, 2009

New Zealand Omnibus Survey 1992 as it appears in P.N. Lee Statistics and Computing Ltd. International Mortality and Smoking Statistics Version 4.04. Sutton, United Kingdom: P.N. Lee Statistics and Computing Ltd, 2009

New Zealand Omnibus Survey 1993 as it appears in P.N. Lee Statistics and Computing Ltd. International Mortality and Smoking Statistics Version 4.04. Sutton, United Kingdom: P.N. Lee Statistics and Computing Ltd, 2009

New Zealand Omnibus Survey 1994 as it appears in P.N. Lee Statistics and Computing Ltd. International Mortality and Smoking Statistics Version 4.04. Sutton, United Kingdom: P.N. Lee Statistics and Computing Ltd, 2009

New Zealand Omnibus Survey 1995 as it appears in P.N. Lee Statistics and Computing Ltd. International Mortality and Smoking Statistics Version 4.04. Sutton, United Kingdom: P.N. Lee Statistics and Computing Ltd, 2009

New Zealand Omnibus Survey 1996 as it appears in P.N. Lee Statistics and Computing Ltd. International Mortality and Smoking Statistics Version 4.04. Sutton, United Kingdom: P.N. Lee Statistics and Computing Ltd, 2009

New Zealand Omnibus Survey 1997 as it appears in P.N. Lee Statistics and Computing Ltd. International Mortality and Smoking Statistics Version 4.04. Sutton, United Kingdom: P.N. Lee Statistics and Computing Ltd, 2009

New Zealand Omnibus Survey 1998 as it appears in P.N. Lee Statistics and Computing Ltd. International Mortality and Smoking Statistics Version 4.04. Sutton, United Kingdom: P.N. Lee Statistics and Computing Ltd, 2009

New Zealand Omnibus Survey 1999 as it appears in P.N. Lee Statistics and Computing Ltd. International Mortality and Smoking Statistics Version 4.04. Sutton, United Kingdom: P.N. Lee Statistics and Computing Ltd, 2009

New Zealand Omnibus Survey 2000 as it appears in P.N. Lee Statistics and Computing Ltd. International Mortality and Smoking Statistics Version 4.04. Sutton, United Kingdom: P.N. Lee Statistics and Computing Ltd, 2009

New Zealand Omnibus Survey 2001 as it appears in P.N. Lee Statistics and Computing Ltd. International Mortality and Smoking Statistics Version 4.04. Sutton, United Kingdom: P.N. Lee Statistics and Computing Ltd, 2009

New Zealand Omnibus Survey 2002 as it appears in P.N. Lee Statistics and Computing Ltd. International Mortality and Smoking Statistics Version 4.04. Sutton, United Kingdom: P.N. Lee Statistics and Computing Ltd, 2009

New Zealand Omnibus Survey 2003 as it appears in P.N. Lee Statistics and Computing Ltd. International Mortality and Smoking Statistics Version 4.04. Sutton, United Kingdom: P.N. Lee Statistics and Computing Ltd, 2009

New Zealand Omnibus Survey 2004 as it appears in P.N. Lee Statistics and Computing Ltd. International Mortality and Smoking Statistics Version 4.04. Sutton, United Kingdom: P.N. Lee Statistics and Computing Ltd, 2009

New Zealand Omnibus Survey 2005 as it appears in P.N. Lee Statistics and Computing Ltd. International Mortality and Smoking Statistics Version 4.04. Sutton, United Kingdom: P.N. Lee Statistics and Computing Ltd, 2009

Northern Ireland Statistics and Research Agency. Central Survey Unit, Northern Ireland Health and Social Wellbeing Survey, 1997 [computer file]. Colchester, Essex: UK Data Archive [distributor], October 2002. SN: 4589, http://dx.doi.org/10.5255/UKDA-SN-4589-1

Norway Adolescent Smoking Behavior Survey 1980 as it appears in P.N. Lee Statistics and Computing Ltd. International Mortality and Smoking Statistics Version 4.04. Sutton, United Kingdom: P.N. Lee Statistics and Computing Ltd, 2009

Norway Adolescent Smoking Behavior Survey 1985 as it appears in P.N. Lee Statistics and Computing Ltd. International Mortality and Smoking Statistics Version 4.04. Sutton, United Kingdom: P.N. Lee Statistics and Computing Ltd, 2009

Norway Adolescent Smoking Behavior Survey 1990 as it appears in P.N. Lee Statistics and Computing Ltd. International Mortality and Smoking Statistics Version 4.04. Sutton, United Kingdom: P.N. Lee Statistics and Computing Ltd, 2009

Norway Adolescent Smoking Behavior Survey 1995 as it appears in P.N. Lee Statistics and Computing Ltd. International Mortality and Smoking Statistics Version 4.04. Sutton, United Kingdom: P.N. Lee Statistics and Computing Ltd, 2009

Norway Adolescent Smoking Behavior Survey 2000 as it appears in P.N. Lee Statistics and Computing Ltd. International Mortality and Smoking Statistics Version 4.04. Sutton, United Kingdom: P.N. Lee Statistics and Computing Ltd, 2009

Norway Adolescent Smoking Behavior Survey 2005 as it appears in P.N. Lee Statistics and Computing Ltd. International Mortality and Smoking Statistics Version 4.04. Sutton, United Kingdom: P.N. Lee Statistics and Computing Ltd, 2009

Norway Survey of Living Conditions 2002 as it appears in P.N. Lee Statistics and Computing Ltd. International Mortality and Smoking Statistics Version 4.04. Sutton, United Kingdom: P.N. Lee Statistics and Computing Ltd, 2009

Norway Survey of Living Conditions 2008-2009 as it appears in P.N. Lee Statistics and Computing Ltd. International Mortality and Smoking Statistics Version 4.04. Sutton, United Kingdom: P.N. Lee Statistics and Computing Ltd, 2009

Norway Survey of Living Conditions Concerning Health, Care and Social Relations 1998 as it appears in P.N. Lee Statistics and Computing Ltd. International Mortality and Smoking Statistics Version 4.04. Sutton, United Kingdom: P.N. Lee Statistics and Computing Ltd, 2009

Norway Young Adult Drug Use Survey 1998 as it appears in P.N. Lee Statistics and Computing Ltd. International Mortality and Smoking Statistics Version 4.04. Sutton, United Kingdom: P.N. Lee Statistics and Computing Ltd, 2009

Norway Young Adult Drug Use Survey 2002 as it appears in P.N. Lee Statistics and Computing Ltd. International Mortality and Smoking Statistics Version 4.04. Sutton, United Kingdom: P.N. Lee Statistics and Computing Ltd, 2009

Norway Young Adult Drug Use Survey 2006 as it appears in P.N. Lee Statistics and Computing Ltd. International Mortality and Smoking Statistics Version 4.04. Sutton, United Kingdom: P.N. Lee Statistics and Computing Ltd, 2009

Office for National Statistics (United Kingdom). United Kingdom General Household Survey 1990-1991 - UK Data Service

Office for National Statistics (United Kingdom). United Kingdom General Household Survey 2002-2003 - ONS

Office for National Statistics (United Kingdom). United Kingdom General Household Survey 2004-2005 - ONS

Office for National Statistics (United Kingdom). United Kingdom General Lifestyle Survey 2007 - ONS

Office for National Statistics (United Kingdom). United Kingdom Omnibus Survey Tobacco Consumption 2004

Office for National Statistics, Mental Health of Children and Adolescents in Great Britain, 1999 [computer file]. Colchester, Essex: UK Data Archive [distributor], December 2000. SN: 4227, http://dx.doi.org/10.5255/UKDA-SN-4227-1

Office for National Statistics, Psychiatric Morbidity among Adults Living in Private Households, 2000 [computer file]. Colchester, Essex: UK Data Archive [distributor], May 2003. SN: 4653, http://dx.doi.org/10.5255/UKDA-SN-4653-1

Office for National Statistics. Social and Vital Statistics Division et al. , Mental Health of Children and Young People in Great Britain, 2004 [computer file]. Colchester, Essex: UK Data Archive [distributor], October 2005. SN: 5269, http://dx.doi.org/10.5255/UKDA-SN-5269-1

Office for National Statistics. Social and Vital Statistics Division, General Household Survey, 2003-2004 [computer file]. 2nd Edition. Colchester, Essex: UK Data Archive [distributor], February 2006. SN: 5150, http://dx.doi.org/10.5255/UKDA-SN-5150-1

Office for National Statistics. Social and Vital Statistics Division, General Household Survey, 2005 [computer file]. 2nd Edition. Colchester, Essex: UK Data Archive [distributor], November 2007. SN: 5640, http://dx.doi.org/10.5255/UKDA-SN-5640-1

Office for National Statistics. Social and Vital Statistics Division, General Household Survey, 2006 [computer file]. 3rd Edition. Colchester, Essex: UK Data Archive [distributor], February 2009. SN: 5804, http://dx.doi.org/10.5255/UKDA-SN-5804-1

Office for National Statistics. Social Survey Division, General Household Survey, 1996-1997 [computer file]. 3rd Edition. Colchester, Essex: UK Data Archive [distributor], July 2002. SN: 3804, http://dx.doi.org/10.5255/UKDA-SN-3804-1

Office for National Statistics. Social Survey Division, General Household Survey, 2001-2002 [computer file]. 4th Edition. Colchester, Essex: UK Data Archive [distributor], February 2006. SN: 4646, http://dx.doi.org/10.5255/UKDA-SN-4646-1

Office of Population Censuses and Surveys. Social Survey Division, General Household Survey, 1984 [computer file]. Colchester, Essex: UK Data Archive [distributor], December 1986. SN: 2154, http://dx.doi.org/10.5255/UKDA-SN-2154-1

Office of Population Censuses and Surveys. Social Survey Division, General Household Survey, 1986 [computer file]. Colchester, Essex: UK Data Archive [distributor], February 1989. SN: 2569, http://dx.doi.org/10.5255/UKDA-SN-2569-1

Office of Population Censuses and Surveys. Social Survey Division, General Household Survey, 1992-1993 [computer file]. Colchester, Essex: UK Data Archive [distributor], January 1994. SN: 3166, http://dx.doi.org/10.5255/UKDA-SN-3166-1

Office of Population Censuses and Surveys. Social Survey Division, General Household Survey, 1994-1995 [computer file]. Colchester, Essex: UK Data Archive [distributor], May 1996. SN: 3538, http://dx.doi.org/10.5255/UKDA-SN-3538-1

Office of Population Censuses and Surveys. Social Survey Division, Health Survey for England, 1991-1992: Combined Data File. Colchester, Essex: UK Data Archive [distributor], October 1997. SN: 3238, http://dx.doi.org/10.5255/UKDA-SN-3238-1

Office of Population Censuses and Surveys. Social Survey Division, Health Survey for England, 1993 [Computer file]. Colchester, Essex: UK Data Archive [distributor], April 1995. SN: 3316, http://dx.doi.org/10.5255/UKDA-SN-3316-1

Osler M. Smoking habits in Denmark from 1953 to 1991: a comparative analysis of results from three Nationwide Health Surveys among adult Danes in 1953-1954, 1986-1987, and 1990-1991. Int J Epidemiol. 1992; 21(5): 862-71

P.N. Lee Statistics and Computing Ltd. International Mortality and Smoking Statistics Version 4.04. Sutton, United Kingdom: P.N. Lee Statistics and Computing Ltd, 2009

Park HA. The Korea National Health and Nutrition Examination Survey as a Primary Data Source. Korean J Fam Med. 2013; 34(2): 79

Portugal National Health Survey 1987 as it appears in P.N. Lee Statistics and Computing Ltd. International Mortality and Smoking Statistics Version 4.04. Sutton, United Kingdom: P.N. Lee Statistics and Computing Ltd, 2009

Portugal National Health Survey 1995-1996 as it appears in P.N. Lee Statistics and Computing Ltd. International Mortality and Smoking Statistics Version 4.04. Sutton, United Kingdom: P.N. Lee Statistics and Computing Ltd, 2009

Pricewaterhouse Coopers. Iceland Medical Association Survey 2000

Ramboll. Denmark Monitoring Smoking Habits in the Danish Population 2004-2005

Rexrode KM, Lee IM, Cook NR, Hennekens CH, Buring JE. Baseline characteristics of participants in the Women's Health Study. J Womens Health Gend Based Med. 2000; 9(1): 19-27 as it appears in Thun MJ, Hannan LM, Adams-Campbell LL, Boffetta P, Buring JE, Feskanich D, Flanders WD, Jee SH, Katanoda K, Kolonel LN, Lee IM, Marugame T, Palmer JR, Riboli E, Sobue T, Avila-Tang E, Wilkens LR, Samet JM. Lung cancer occurrence in never-smokers: an analysis of 13 cohorts and 22 cancer registry studies. PLoS Med. 2008; 5(9): e185

Robert Koch Institute. Germany Health Interview and Examination Survey 2008-2011, Baseline Cohort

Russell C, Palmer JR, Adams-Campbell LL, Rosenberg L. Follow-up of a large cohort of Black women. Am J Epidemiol. 2001; 154(9): 845-53 as it appears in Thun MJ, Hannan LM, Adams-Campbell LL, Boffetta P, Buring JE, Feskanich D, Flanders WD, Jee SH, Katanoda K, Kolonel LN, Lee IM, Marugame T, Palmer JR, Riboli E, Sobue T, Avila-Tang E, Wilkens LR, Samet JM. Lung cancer occurrence in never-smokers: an analysis of 13 cohorts and 22 cancer registry studies. PLoS Med. 2008; 5(9): e185

Scientific Institute of Public Health (IPH) (Belgium), Statistics Belgium. Belgium Health Interview Survey 2001

Scientific Institute of Public Health (IPH) (Belgium), Statistics Belgium. Belgium Health Interview Survey 2004

Scientific Institute of Public Health (WIV-ISP) (Belgium), Statistics Belgium. Belgium Health Interview Survey 2008

Secretariat for Programming Drug Abuse Prevention and the Fight against Drug Trafficking (SEDRONAR). Argentina National Psychoactive Substance Consumption Study 2006

Secretariat for Programming Drug Abuse Prevention and the Fight against Drug Trafficking (SEDRONAR). Argentina National Survey of High School Students 2001

Secretariat for Programming Drug Abuse Prevention and the Fight against Drug Trafficking (SEDRONAR). Argentina National Survey on Consumption Prevalence of Psychoactive Substances 1999

Spain Eurobarometer 32: The Single European Market, Drugs, Alcohol, and Cancer 1989 as it appears in P.N. Lee Statistics and Computing Ltd. International Mortality and Smoking Statistics Version 4.04. Sutton, United Kingdom: P.N. Lee Statistics and Computing Ltd, 2009

Spain Eurobarometer 34.1: Health Problems 1990 as it appears in P.N. Lee Statistics and Computing Ltd. International Mortality and Smoking Statistics Version 4.04. Sutton, United Kingdom: P.N. Lee Statistics and Computing Ltd, 2009

Spain Eurobarometer 41: Trade Issues, Blood Donation, AIDS, and Smoking 1994 as it appears in P.N. Lee Statistics and Computing Ltd. International Mortality and Smoking Statistics Version 4.04. Sutton, United Kingdom: P.N. Lee Statistics and Computing Ltd, 2009

Spain European Community Household Panel 1998 as it appears in P.N. Lee Statistics and Computing Ltd. International Mortality and Smoking Statistics Version 4.04. Sutton, United Kingdom: P.N. Lee Statistics and Computing Ltd, 2009

Spain European Community Household Panel 1999 as it appears in P.N. Lee Statistics and Computing Ltd. International Mortality and Smoking Statistics Version 4.04. Sutton, United Kingdom: P.N. Lee Statistics and Computing Ltd, 2009

Spain European Community Household Panel 2000 as it appears in P.N. Lee Statistics and Computing Ltd. International Mortality and Smoking Statistics Version 4.04. Sutton, United Kingdom: P.N. Lee Statistics and Computing Ltd, 2009

Spain European Community Household Panel 2001 as it appears in P.N. Lee Statistics and Computing Ltd. International Mortality and Smoking Statistics Version 4.04. Sutton, United Kingdom: P.N. Lee Statistics and Computing Ltd, 2009

Spain European Health Survey 2009-2010 as it appears in P.N. Lee Statistics and Computing Ltd. International Mortality and Smoking Statistics Version 4.04. Sutton, United Kingdom: P.N. Lee Statistics and Computing Ltd, 2009

Spain Lifestyles of School Age Teenagers 2002 as it appears in P.N. Lee Statistics and Computing Ltd. International Mortality and Smoking Statistics Version 4.04. Sutton, United Kingdom: P.N. Lee Statistics and Computing Ltd, 2009

Spain National Health Survey 2003 as it appears in P.N. Lee Statistics and Computing Ltd. International Mortality and Smoking Statistics Version 4.04. Sutton, United Kingdom: P.N. Lee Statistics and Computing Ltd, 2009

Spain National Health Survey 2006-2007 as it appears in P.N. Lee Statistics and Computing Ltd. International Mortality and Smoking Statistics Version 4.04. Sutton, United Kingdom: P.N. Lee Statistics and Computing Ltd, 2009

Statistics Canada. Canada Community Health Survey 2000-2001. Ottawa, Canada: Statistics Canada, 2003

Statistics Canada. Canada Community Health Survey 2003. Ottawa, Canada: Statistics Canada

Statistics Canada. Canada Community Health Survey 2009-2010. Ottawa, Canada: Statistics Canada, 2011

Statistics Canada. Canada National Population Health Survey 1994-1995. Ottawa, Canada: Statistics Canada

Statistics Canada. Canada National Population Health Survey 1996-1997. Ottawa, Canada: Statistics Canada

Statistics Canada. Canada National Population Health Survey 1998-1999. Ottawa, Canada: Statistics Canada

Statistics Iceland. Iceland Smoking Habits 15-79 Years

Statistics Iceland. Iceland Smoking Habits by Sex and Age

Statistics Iceland. Iceland Statistical Yearbook 2011. Reykjavík, Iceland: Statistics Iceland, 2011

Statistics Iceland. Iceland Statistical Yearbook 2012. Reykjavík, Iceland: Statistics Iceland, 2012

Statistics Netherlands. Netherlands Permanent Quality of Life Survey 1997

Statistics Netherlands. Netherlands Permanent Quality of Life Survey 1999

Statistics Netherlands. Netherlands Permanent Quality of Life Survey 2000

Statistics Netherlands. Netherlands Permanent Quality of Life Survey 2001

Statistics Netherlands. Netherlands Permanent Quality of Life Survey 2002

Statistics Netherlands. Netherlands Permanent Quality of Life Survey 2003

Statistics Netherlands. Netherlands Permanent Quality of Life Survey 2004

Statistics Netherlands. Netherlands Permanent Quality of Life Survey 2005

Statistics Netherlands. Netherlands Permanent Quality of Life Survey 2006

Statistics Netherlands. Netherlands Permanent Quality of Life Survey 2007

Statistics Netherlands. Netherlands Permanent Quality of Life Survey 2008

Statistics New Zealand. New Zealand Census 1996

Statistics New Zealand. New Zealand Census 2006

Statistics Norway. Norway Lifestyle Habits by Gender and Age. Oslo, Norway: Statistics Norway

Statistics Sweden, Swedish National Institute of Public Health. Sweden National Survey of Public Health 2004

Statistics Sweden, Swedish National Institute of Public Health. Sweden National Survey of Public Health 2005

Statistics Sweden, Swedish National Institute of Public Health. Sweden National Survey of Public Health 2006

Statistics Sweden, Swedish National Institute of Public Health. Sweden National Survey of Public Health 2007

Statistics Sweden, Swedish National Institute of Public Health. Sweden National Survey of Public Health 2008

Statistics Sweden, Swedish National Institute of Public Health. Sweden National Survey of Public Health 2009

Statistics Sweden, Swedish National Institute of Public Health. Sweden National Survey of Public Health 2012

Statistics Sweden. Sweden Survey of Living Conditions 1980

Statistics Sweden. Sweden Survey of Living Conditions 1982-1983

Statistics Sweden. Sweden Survey of Living Conditions 1984-1985

Statistics Sweden. Sweden Survey of Living Conditions 1986-1987

Statistics Sweden. Sweden Survey of Living Conditions 1988-1989

Statistics Sweden. Sweden Survey of Living Conditions 1990-1991

Statistics Sweden. Sweden Survey of Living Conditions 1992-1993

Statistics Sweden. Sweden Survey of Living Conditions 1994-1995

Statistics Sweden. Sweden Survey of Living Conditions 1996-1997

Statistics Sweden. Sweden Survey of Living Conditions 1999

Statistics Sweden. Sweden Survey of Living Conditions 2000

Statistics Sweden. Sweden Survey of Living Conditions 2001

Statistics Sweden. Sweden Survey of Living Conditions 2003

Statistics Sweden. Sweden Survey of Living Conditions 2008-2009

Statistics Sweden. Sweden Survey of Living Conditions 2010-2011

STIVORO (Netherlands), TNS-NIPO (Netherlands). Netherlands Continuous Survey of Smoking Habits 2013

STIVORO (Netherlands). Netherlands STIVORO Annual National Report 2007. The Hague, Netherlands: STIVORO (Netherlands), 2008

Suh I. Cardiovascular mortality in Korea: a country experiencing epidemiologic transition. Acta Cardiol. 2001; 56(2): 75–81

Sutton-Tyrell, Kim, Faith Selzer, MaryFran Sowers, Joel Finkelstein, Lynda Powell, Ellen Gold, Gail Greendale, Gerson Weiss, and Karen Matthews. Study of Women's Health Across the Nation (SWAN), 2002-2004: Visit 06 Dataset. ICPSR31181-v1. Ann Arbor, MI: Inter-university Consortium for Political and Social Research [distributor], 2014-09-24. http://doi.org/10.3886/ICPSR31181.v1

Sutton-Tyrell, Kim, Faith Selzer, MaryFran Sowers, Joel Finkelstein, Lynda Powell, Ellen Gold, Gail Greendale, Gerson Weiss, and Karen Matthews. Study of Women's Health Across the Nation (SWAN), 2003-2005: Visit 07 Dataset. ICPSR31901-v1. Ann Arbor, MI: Inter-university Consortium for Political and Social Research [distributor], 2014-09-30. http://doi.org/10.3886/ICPSR31901.v1

Sutton-Tyrell, Kim, Faith Selzer, MaryFran Sowers, Joel Finkelstein, Lynda Powell, Ellen Gold, Gail Greendale, Gerson Weiss, and Karen Matthews. Study of Women's Health Across the Nation (SWAN), 2004-2006: Visit 08 Dataset. ICPSR32122-v1. Ann Arbor, MI: Inter-university Consortium for Political and Social Research [distributor], 2014-09-30. http://doi.org/10.3886/ICPSR32122.v1

Sutton-Tyrell, Kim, Faith Selzer, MaryFran Sowers, Joel Finkelstein, Lynda Powell, Ellen Gold, Gail Greendale, Gerson Weiss, and Karen Matthews. Study of Women's Health Across the Nation (SWAN), 2005-2007: Visit 09 Dataset. ICPSR32721-v1. Ann Arbor, MI: Inter-university Consortium for Political and Social Research [distributor], 2014-09-30. http://doi.org/10.3886/ICPSR32721.v1

Sutton-Tyrell, Kim, Faith Selzer, MaryFran Sowers, Robert Neer, Lynda Powell, Ellen Gold, Gail Greendale, Gerson Weiss, Karen Matthews, and Sonja McKinlay. Study of Women's Health Across the Nation (SWAN), 2000-2002: Visit 04 Dataset. ICPSR30142-v1. Ann Arbor, MI: Inter-university Consortium for Political and Social Research[distributor], 2014-02-13. http://doi.org/10.3886/ICPSR30142.v1

Sutton-Tyrell, Kim, Faith Selzer, MaryFran Sowers, Robert Neer, Lynda Powell, Ellen Gold, Gail Greendale, Gerson Weiss, Karen Matthews, and Sonja McKinlay. Study of Women's Health Across the Nation (SWAN), 2001-2003: Visit 05 Dataset. ICPSR30501-v1. Ann Arbor, MI: Inter-university Consortium for Political and Social Research [distributor], 2014-09-02. http://doi.org/10.3886/ICPSR30501.v1

Sutton-Tyrell, Kim, Faith Selzer, MaryFran Sowers, Robert Neer, Lynda Powell, Ellen Gold, Gail Greendale, Gerson Weiss, Karen Matthews, and Sonja McKinlay. Study of Women's Health Across the Nation (SWAN), 2001-2003: Visit 05 Dataset. ICPSR30501-v1. Ann Arbor, MI: Inter-university Consortium for Political and Social Research [distributor], 2014-09-02. http://doi.org/10.3886/ICPSR30501.v1

Sutton-Tyrrell, Kim, Faith Selzer, MaryFran Sowers, Joel Finkelstein, Lynda Powell, Ellen Gold, Gail David, Gerson Weiss, and Karen Matthews. Study of Women Across the Nation (SWAN), 2006-2008: Visit 10 Dataset. ICPSR32961-v1. Ann Arbor, MI: Inter-university Consortium for Political and Social Research [distributor], 2014-10-08. http://doi.org/10.3886/ICPSR32961.v1

Sutton-Tyrrell, Kim, Faith Selzer, MaryFran Sowers, Robert Neer, Lynda Powell, Ellen Gold, Gail Greendale, Gerson Weiss, Karen Matthews, and Sonja McKinlay. Study of Women's Health Across the Nation (SWAN), 1996-1997: Baseline Dataset. ICPSR28762-v2. Ann Arbor, MI: Inter-university Consortium for Political and Social Research[distributor], 2014-02-04. http://doi.org/10.3886/ICPSR28762.v2

Sutton-Tyrrell, Kim, Faith Selzer, MaryFran Sowers, Robert Neer, Lynda Powell, Ellen Gold, Gail Greendale, Gerson Weiss, Karen Matthews, and Sonja McKinlay. Study of Women's Health Across the Nation (SWAN), 1997-1999: Visit 01 Dataset. ICPSR29221-v1. Ann Arbor, MI: Inter-university Consortium for Political and Social Research[distributor], 2014-02-05. http://doi.org/10.3886/ICPSR29221.v1

Sutton-Tyrrell, Kim, Faith Selzer, MaryFran Sowers, Robert Neer, Lynda Powell, Ellen Gold, Gail Greendale, Gerson Weiss, Karen Matthews, and Sonja McKinlay. Study of Women's Health Across the Nation (SWAN), 1998-2000: Visit 02 Dataset. ICPSR29401-v1. Ann Arbor, MI: Inter-university Consortium for Political and Social Research[distributor], 2014-02-06. http://doi.org/10.3886/ICPSR29401.v1

Sutton-Tyrrell, Kim, Faith Selzer, MaryFran Sowers, Robert Neer, Lynda Powell, Ellen Gold, Gail Greendale, Gerson Weiss, Karen Matthews, and Sonja McKinlay. Study of Women's Health Across the Nation (SWAN), 1999-2001: Visit 03 Dataset. ICPSR29701-v1. Ann Arbor, MI: Inter-university Consortium for Political and Social Research [distributor], 2014-02-12. http://doi.org/10.3886/ICPSR29701.v1

Sweden Smoking Habits Survey 1980 as it appears in P.N. Lee Statistics and Computing Ltd. International Mortality and Smoking Statistics Version 4.04. Sutton, United Kingdom: P.N. Lee Statistics and Computing Ltd, 2009

Sweden Smoking Habits Survey 1981 as it appears in P.N. Lee Statistics and Computing Ltd. International Mortality and Smoking Statistics Version 4.04. Sutton, United Kingdom: P.N. Lee Statistics and Computing Ltd, 2009

Sweden Smoking Habits Survey 1982 as it appears in P.N. Lee Statistics and Computing Ltd. International Mortality and Smoking Statistics Version 4.04. Sutton, United Kingdom: P.N. Lee Statistics and Computing Ltd, 2009

Sweden Smoking Habits Survey 1983 - SCB as it appears in P.N. Lee Statistics and Computing Ltd. International Mortality and Smoking Statistics Version 4.04. Sutton, United Kingdom: P.N. Lee Statistics and Computing Ltd, 2009

Sweden Smoking Habits Survey 1983 as it appears in P.N. Lee Statistics and Computing Ltd. International Mortality and Smoking Statistics Version 4.04. Sutton, United Kingdom: P.N. Lee Statistics and Computing Ltd, 2009

Sweden Smoking Habits Survey 1984 as it appears in P.N. Lee Statistics and Computing Ltd. International Mortality and Smoking Statistics Version 4.04. Sutton, United Kingdom: P.N. Lee Statistics and Computing Ltd, 2009

Sweden Smoking Habits Survey 1985 as it appears in P.N. Lee Statistics and Computing Ltd. International Mortality and Smoking Statistics Version 4.04. Sutton, United Kingdom: P.N. Lee Statistics and Computing Ltd, 2009

Sweden Smoking Habits Survey 1986 as it appears in P.N. Lee Statistics and Computing Ltd. International Mortality and Smoking Statistics Version 4.04. Sutton, United Kingdom: P.N. Lee Statistics and Computing Ltd, 2009

Sweden Smoking Habits Survey 1987 as it appears in P.N. Lee Statistics and Computing Ltd. International Mortality and Smoking Statistics Version 4.04. Sutton, United Kingdom: P.N. Lee Statistics and Computing Ltd, 2009

Swedish National Institute of Public Health. Sweden National Public Health Survey Smoking Data 2004-2014

Swedish National Institute of Public Health. Sweden Young People's Tobacco Use, Knowledge, and Attitudes Survey 2003

Swedish National Institute of Public Health. Sweden Young People's Tobacco Use, Knowledge, and Attitudes Survey 2009

Switzerland Health Survey 1981-1982 as it appears in P.N. Lee Statistics and Computing Ltd. International Mortality and Smoking Statistics Version 4.04. Sutton, United Kingdom: P.N. Lee Statistics and Computing Ltd, 2009

Telepanel Foundation (STP), CentERdata. Netherlands Dutch National Bank Household Survey 1993-1994. Tillburg, Netherlands: CentERdata

Telepanel Foundation (STP), CentERdata. Netherlands Dutch National Bank Household Survey 1994. Tillburg, Netherlands: CentERdata

Thefeld W. Verbreitung der Herz-Kreislauf-Risikofaktoren Hypercholesterinämie, Übergewicht, Hypertonie und Rauchen in der Bevölkerung. Bundesgesundheitsblatt. 2000; 43(6): 415-23

Thun MJ, Henley SJ, Burns D, Jemal A, Shanks TG, Calle EE. Lung cancer death rates in lifelong nonsmokers. J Natl Cancer Inst. 2006; 98(10): 691-9 as it appears in Thun MJ, Hannan LM, Adams-Campbell LL, Boffetta P, Buring JE, Feskanich D, Flanders WD, Jee SH, Katanoda K, Kolonel LN, Lee IM, Marugame T, Palmer JR, Riboli E, Sobue T, Avila-Tang E, Wilkens LR, Samet JM. Lung cancer occurrence in never-smokers: an analysis of 13 cohorts and 22 cancer registry studies. PLoS Med. 2008; 5(9): e185

TNS ILRES (Luxembourg). Smoking in Luxembourg 1987

TNS ILRES (Luxembourg). Smoking in Luxembourg 1993

TNS ILRES (Luxembourg). Smoking in Luxembourg 1998

TNS ILRES (Luxembourg). Smoking in Luxembourg 2001

TNS ILRES (Luxembourg). Smoking in Luxembourg 2002

TNS ILRES (Luxembourg). Smoking in Luxembourg 2003

TNS ILRES (Luxembourg). Smoking in Luxembourg 2004

Tobacco in Australia: A Summary of Related Statistics as it appears in P.N. Lee Statistics and Computing Ltd. International Mortality and Smoking Statistics Version 4.04. Sutton, United Kingdom: P.N. Lee Statistics and Computing Ltd, 2009

Tountas Y, Oikonomou N, Pallikarona G, Dimitrakaki C, Tzavara C, Souliotis K, Mariolis A, Pappa E, Kontodimopoulos N, Niakas D. Sociodemographic and socioeconomic determinants of health services utilization in Greece: the Hellas Health I study. Health Serv Manage Res. 2011; 24(1): 8-18

Tran PD, Leclerc A, Chastang JF, Goldberg M. Regional disparities in cardiovascular risk factors in France: a five-year analysis of the GAZEL cohort. Eur J Epidemiol. 1998; 14(6): 535–43

Trinity College Dublin. Ireland Longitudinal Study on Ageing 2009-2011. Dublin, Ireland: Irish Social Science Data Archive, University College Dublin

Tsakraklides V, Mentis A, Efstathiou P, Trichopoulos D. Tobacco smoking by young adult males in Greece. Hygie. 1983; 2(2): 26-30 as it appears in P.N. Lee Statistics and Computing Ltd. International Mortality and Smoking Statistics Version 4.04. Sutton, United Kingdom: P.N. Lee Statistics and Computing Ltd, 2009

Umeå University. Sweden - Norrbotten and Västerbotten Northern Sweden MONICA Study 1990

Umeå University. Sweden - Norrbotten and Västerbotten Northern Sweden MONICA Study 1994

Umeå University. Sweden - Norrbotten and Västerbotten Northern Sweden MONICA Study 1999

Umeå University. Sweden - Norrbotten and Västerbotten Northern Sweden MONICA Study 2004

Umeå University. Sweden - Norrbotten and Västerbotten Northern Sweden MONICA Study 2009

United Kingdom - England Teenage Smoking Attitudes Survey 1996 as it appears in P.N. Lee Statistics and Computing Ltd. International Mortality and Smoking Statistics Version 4.04. Sutton, United Kingdom: P.N. Lee Statistics and Computing Ltd, 2009

United Kingdom - England Teenage Smoking Attitudes Survey 1997 as it appears in P.N. Lee Statistics and Computing Ltd. International Mortality and Smoking Statistics Version 4.04. Sutton, United Kingdom: P.N. Lee Statistics and Computing Ltd, 2009

United Kingdom - England Teenage Smoking Attitudes Survey 1998 as it appears in P.N. Lee Statistics and Computing Ltd. International Mortality and Smoking Statistics Version 4.04. Sutton, United Kingdom: P.N. Lee Statistics and Computing Ltd, 2009

United Kingdom Eurobarometer 32: The Single European Market, Drugs, Alcohol, and Cancer 1989 as it appears in P.N. Lee Statistics and Computing Ltd. International Mortality and Smoking Statistics Version 4.04. Sutton, United Kingdom: P.N. Lee Statistics and Computing Ltd, 2009

United Kingdom General Household Survey 1998-1999 - ONS as it appears in P.N. Lee Statistics and Computing Ltd. International Mortality and Smoking Statistics Version 4.04. Sutton, United Kingdom: P.N. Lee Statistics and Computing Ltd, 2009

United Kingdom Health Related Behavior Questionnaire Data 1984 as it appears in P.N. Lee Statistics and Computing Ltd. International Mortality and Smoking Statistics Version 4.04. Sutton, United Kingdom: P.N. Lee Statistics and Computing Ltd, 2009

United Kingdom Health Related Behavior Questionnaire Data 1985 as it appears in P.N. Lee Statistics and Computing Ltd. International Mortality and Smoking Statistics Version 4.04. Sutton, United Kingdom: P.N. Lee Statistics and Computing Ltd, 2009

United Kingdom Health Related Behavior Questionnaire Data 1986 as it appears in P.N. Lee Statistics and Computing Ltd. International Mortality and Smoking Statistics Version 4.04. Sutton, United Kingdom: P.N. Lee Statistics and Computing Ltd, 2009

United Kingdom Health Related Behavior Questionnaire Data 1987 as it appears in P.N. Lee Statistics and Computing Ltd. International Mortality and Smoking Statistics Version 4.04. Sutton, United Kingdom: P.N. Lee Statistics and Computing Ltd, 2009

United Kingdom Omnibus Survey Tobacco Consumption 1996 as it appears in P.N. Lee Statistics and Computing Ltd. International Mortality and Smoking Statistics Version 4.04. Sutton, United Kingdom: P.N. Lee Statistics and Computing Ltd, 2009

United Kingdom Omnibus Survey Tobacco Consumption 1997 as it appears in P.N. Lee Statistics and Computing Ltd. International Mortality and Smoking Statistics Version 4.04. Sutton, United Kingdom: P.N. Lee Statistics and Computing Ltd, 2009

United Kingdom Omnibus Survey Tobacco Consumption 1999 as it appears in P.N. Lee Statistics and Computing Ltd. International Mortality and Smoking Statistics Version 4.04. Sutton, United Kingdom: P.N. Lee Statistics and Computing Ltd, 2009

United Kingdom Omnibus Survey Tobacco Consumption 2000 as it appears in P.N. Lee Statistics and Computing Ltd. International Mortality and Smoking Statistics Version 4.04. Sutton, United Kingdom: P.N. Lee Statistics and Computing Ltd, 2009

United Kingdom Omnibus Survey Tobacco Consumption 2001 as it appears in P.N. Lee Statistics and Computing Ltd. International Mortality and Smoking Statistics Version 4.04. Sutton, United Kingdom: P.N. Lee Statistics and Computing Ltd, 2009

United Kingdom Omnibus Survey Tobacco Consumption 2002 as it appears in P.N. Lee Statistics and Computing Ltd. International Mortality and Smoking Statistics Version 4.04. Sutton, United Kingdom: P.N. Lee Statistics and Computing Ltd, 2009

United Kingdom Omnibus Survey Tobacco Consumption 2003 as it appears in P.N. Lee Statistics and Computing Ltd. International Mortality and Smoking Statistics Version 4.04. Sutton, United Kingdom: P.N. Lee Statistics and Computing Ltd, 2009

United Kingdom Omnibus Survey Tobacco Consumption 2005 as it appears in P.N. Lee Statistics and Computing Ltd. International Mortality and Smoking Statistics Version 4.04. Sutton, United Kingdom: P.N. Lee Statistics and Computing Ltd, 2009

United Kingdom Smoking Habits Survey 1980 as it appears in P.N. Lee Statistics and Computing Ltd. International Mortality and Smoking Statistics Version 4.04. Sutton, United Kingdom: P.N. Lee Statistics and Computing Ltd, 2009

United Kingdom Smoking Habits Survey 1981 as it appears in P.N. Lee Statistics and Computing Ltd. International Mortality and Smoking Statistics Version 4.04. Sutton, United Kingdom: P.N. Lee Statistics and Computing Ltd, 2009

United Kingdom Smoking Habits Survey 1982 as it appears in P.N. Lee Statistics and Computing Ltd. International Mortality and Smoking Statistics Version 4.04. Sutton, United Kingdom: P.N. Lee Statistics and Computing Ltd, 2009

United Kingdom Smoking Habits Survey 1983 as it appears in P.N. Lee Statistics and Computing Ltd. International Mortality and Smoking Statistics Version 4.04. Sutton, United Kingdom: P.N. Lee Statistics and Computing Ltd, 2009

United Kingdom Smoking Habits Survey 1984 as it appears in P.N. Lee Statistics and Computing Ltd. International Mortality and Smoking Statistics Version 4.04. Sutton, United Kingdom: P.N. Lee Statistics and Computing Ltd, 2009

United Kingdom Smoking Habits Survey 1985 as it appears in P.N. Lee Statistics and Computing Ltd. International Mortality and Smoking Statistics Version 4.04. Sutton, United Kingdom: P.N. Lee Statistics and Computing Ltd, 2009

United Kingdom Smoking Habits Survey 1986 as it appears in P.N. Lee Statistics and Computing Ltd. International Mortality and Smoking Statistics Version 4.04. Sutton, United Kingdom: P.N. Lee Statistics and Computing Ltd, 2009

United Kingdom Smoking Habits Survey 1987 as it appears in P.N. Lee Statistics and Computing Ltd. International Mortality and Smoking Statistics Version 4.04. Sutton, United Kingdom: P.N. Lee Statistics and Computing Ltd, 2009

United States Adult Use of Tobacco Survey 1986 as it appears in P.N. Lee Statistics and Computing Ltd. International Mortality and Smoking Statistics Version 4.04. Sutton, United Kingdom: P.N. Lee Statistics and Computing Ltd, 2009

United States Commonwealth Fund Survey of the Health of Adolescent Girls and Boys 1996-1997 as it appears in P.N. Lee Statistics and Computing Ltd. International Mortality and Smoking Statistics Version 4.04. Sutton, United Kingdom: P.N. Lee Statistics and Computing Ltd, 2009

United States Department of Health and Human Services. National Institutes of Health. National Institute on Drug Abuse. National Household Survey on Drug Abuse, 1985. ICPSR06844-v2. Ann Arbor, MI: Inter-university Consortium for Political and Social Research [distributor], 2008-07-25. doi:10.3886/ICPSR06844.v2

United States Department of Health and Human Services. National Institutes of Health. National Institute on Drug Abuse. National Household Survey on Drug Abuse, 1988. ICPSR09522-v3. Ann Arbor, MI: Inter-university Consortium for Political and Social Research [distributor], 2008-07-23. doi:10.3886/ICPSR09522.v3

United States Department of Health and Human Services. National Institutes of Health. National Institute on Drug Abuse. National Household Survey on Drug Abuse, 1990. ICPSR09833-v4. Ann Arbor, MI: Inter-university Consortium for Political and Social Research [distributor], 2008-07-25. doi:10.3886/ICPSR09833.v4

United States Department of Health and Human Services. National Institutes of Health. National Institute on Drug Abuse. National Household Survey on Drug Abuse, 1991. ICPSR06128-v2. Ann Arbor, MI: Inter-university Consortium for Political and Social Research [distributor], 2008-08-05. doi:10.3886/ICPSR06128.v2

United States Department of Health and Human Services. Substance Abuse and Mental Health Services Administration. Center for Behavioral Health Statistics and Quality. National Survey on Drug Use and Health, 2010. ICPSR32722-v3. Ann Arbor, MI: Inter-university Consortium for Political and Social Research [distributor], 2012-10-19. doi:10.3886/ICPSR32722.v3

United States Department of Health and Human Services. Substance Abuse and Mental Health Services Administration. Center for Behavioral Health Statistics and Quality. National Survey on Drug Use and Health, 2011. ICPSR34481-v1. Ann Arbor, MI: Inter-university Consortium for Political and Social Research [distributor], 2012-11-28. doi:10.3886/ICPSR34481.v1

United States Department of Health and Human Services. Substance Abuse and Mental Health Services Administration. Office of Applied Studies. National Household Survey on Drug Abuse, 1992. ICPSR06887-v2. Ann Arbor, MI: Inter-university Consortium for Political and Social Research [distributor], 2008-08-18. doi:10.3886/ICPSR06887.v2

United States Department of Health and Human Services. Substance Abuse and Mental Health Services Administration. Office of Applied Studies. National Household Survey on Drug Abuse, 1993. ICPSR06852-v1. Ann Arbor, MI: Inter-university Consortium for Political and Social Research [distributor], 2008-10-28. doi:10.3886/ICPSR06852.v1

United States Department of Health and Human Services. Substance Abuse and Mental Health Services Administration. Office of Applied Studies. National Household Survey on Drug Abuse, 1994. ICPSR06949-v1. Ann Arbor, MI: Inter-university Consortium for Political and Social Research [distributor], 2008-10-28. doi:10.3886/ICPSR06949.v1

United States Department of Health and Human Services. Substance Abuse and Mental Health Services Administration. Office of Applied Studies. National Household Survey on Drug Abuse, 1995. ICPSR06950-v1. Ann Arbor, MI: Inter-university Consortium for Political and Social Research [distributor], 2008-10-23. doi:10.3886/ICPSR06950.v1

United States Department of Health and Human Services. Substance Abuse and Mental Health Services Administration. Office of Applied Studies. National Household Survey on Drug Abuse, 1996. ICPSR02391-v1. Ann Arbor, MI: Inter-university Consortium for Political and Social Research [distributor], 2008-10-23. doi:10.3886/ICPSR02391.v1

United States Department of Health and Human Services. Substance Abuse and Mental Health Services Administration. Office of Applied Studies. National Household Survey on Drug Abuse, 1997. ICPSR02755-v2. Ann Arbor, MI: Inter-university Consortium for Political and Social Research [distributor], 2008-10-23. doi:10.3886/ICPSR02755.v2

United States Department of Health and Human Services. Substance Abuse and Mental Health Services Administration. Office of Applied Studies. National Survey on Drug Use and Health, 2002. ICPSR03903-v3. Ann Arbor, MI: Inter-university Consortium for Political and Social Research [distributor], 2006-10-26. doi:10.3886/ICPSR03903.v3

United States Department of Health and Human Services. Substance Abuse and Mental Health Services Administration. Office of Applied Studies. National Survey on Drug Use and Health, 2003. ICPSR04138-v2. Ann Arbor, MI: Inter-university Consortium for Political and Social Research [distributor], 2006-10-17. doi:10.3886/ICPSR04138.v2

United States Department of Health and Human Services. Substance Abuse and Mental Health Services Administration. Office of Applied Studies. National Survey on Drug Use and Health, 2004. ICPSR04373-v1. Ann Arbor, MI: Inter-university Consortium for Political and Social Research [distributor], 2006-05-12. doi:10.3886/ICPSR04373.v1

United States Department of Health and Human Services. Substance Abuse and Mental Health Services Administration. Office of Applied Studies. National Survey on Drug Use and Health, 2005. ICPSR04596-v3. Ann Arbor, MI: Inter-university Consortium for Political and Social Research [distributor], 2013-01-22. doi:10.3886/ICPSR04596.v3

United States Department of Health and Human Services. Substance Abuse and Mental Health Services Administration. Office of Applied Studies. National Survey on Drug Use and Health, 2006. ICPSR21240-v5. Ann Arbor, MI: Inter-university Consortium for Political and Social Research [distributor], 2013-01-07. doi:10.3886/ICPSR21240.v5

United States Department of Health and Human Services. Substance Abuse and Mental Health Services Administration. Office of Applied Studies. National Survey on Drug Use and Health, 2007. ICPSR23782-v3. Ann Arbor, MI: Inter-university Consortium for Political and Social Research [distributor], 2013-01-04. doi:10.3886/ICPSR23782.v3

United States Department of Health and Human Services. Substance Abuse and Mental Health Services Administration. Office of Applied Studies. National Survey on Drug Use and Health, 2008. ICPSR26701-v3. Ann Arbor, MI: Inter-university Consortium for Political and Social Research [distributor], 2012-12-10. doi:10.3886/ICPSR26701.v3

United States Department of Health and Human Services. Substance Abuse and Mental Health Services Administration. Office of Applied Studies. National Survey on Drug Use and Health, 2009. ICPSR29621-v3. Ann Arbor, MI: Inter-university Consortium for Political and Social Research [distributor], 2012-11-16. doi:10.3886/ICPSR29621.v3

United States Monitoring the Future Survey 1980 as it appears in P.N. Lee Statistics and Computing Ltd. International Mortality and Smoking Statistics Version 4.04. Sutton, United Kingdom: P.N. Lee Statistics and Computing Ltd, 2009

United States Monitoring the Future Survey 1981 as it appears in P.N. Lee Statistics and Computing Ltd. International Mortality and Smoking Statistics Version 4.04. Sutton, United Kingdom: P.N. Lee Statistics and Computing Ltd, 2009

United States Monitoring the Future Survey 1982 as it appears in P.N. Lee Statistics and Computing Ltd. International Mortality and Smoking Statistics Version 4.04. Sutton, United Kingdom: P.N. Lee Statistics and Computing Ltd, 2009

United States Monitoring the Future Survey 1983 as it appears in P.N. Lee Statistics and Computing Ltd. International Mortality and Smoking Statistics Version 4.04. Sutton, United Kingdom: P.N. Lee Statistics and Computing Ltd, 2009

United States Monitoring the Future Survey 1984 as it appears in P.N. Lee Statistics and Computing Ltd. International Mortality and Smoking Statistics Version 4.04. Sutton, United Kingdom: P.N. Lee Statistics and Computing Ltd, 2009

United States Monitoring the Future Survey 1985 as it appears in P.N. Lee Statistics and Computing Ltd. International Mortality and Smoking Statistics Version 4.04. Sutton, United Kingdom: P.N. Lee Statistics and Computing Ltd, 2009

United States Monitoring the Future Survey 1986 as it appears in P.N. Lee Statistics and Computing Ltd. International Mortality and Smoking Statistics Version 4.04. Sutton, United Kingdom: P.N. Lee Statistics and Computing Ltd, 2009

United States Monitoring the Future Survey 1987 as it appears in P.N. Lee Statistics and Computing Ltd. International Mortality and Smoking Statistics Version 4.04. Sutton, United Kingdom: P.N. Lee Statistics and Computing Ltd, 2009

United States Monitoring the Future Survey 1988 as it appears in P.N. Lee Statistics and Computing Ltd. International Mortality and Smoking Statistics Version 4.04. Sutton, United Kingdom: P.N. Lee Statistics and Computing Ltd, 2009

United States Monitoring the Future Survey 1989 as it appears in P.N. Lee Statistics and Computing Ltd. International Mortality and Smoking Statistics Version 4.04. Sutton, United Kingdom: P.N. Lee Statistics and Computing Ltd, 2009

United States Monitoring the Future Survey 1990 as it appears in P.N. Lee Statistics and Computing Ltd. International Mortality and Smoking Statistics Version 4.04. Sutton, United Kingdom: P.N. Lee Statistics and Computing Ltd, 2009

United States National Youth Risk Behavior Survey 1990 as it appears in P.N. Lee Statistics and Computing Ltd. International Mortality and Smoking Statistics Version 4.04. Sutton, United Kingdom: P.N. Lee Statistics and Computing Ltd, 2009

United States Teenage Attitudes and Practices Survey 1988-1989 as it appears in P.N. Lee Statistics and Computing Ltd. International Mortality and Smoking Statistics Version 4.04. Sutton, United Kingdom: P.N. Lee Statistics and Computing Ltd, 2009

United States Tobacco Use Supplement to the Current Population Survey 1985 as it appears in P.N. Lee Statistics and Computing Ltd. International Mortality and Smoking Statistics Version 4.04. Sutton, United Kingdom: P.N. Lee Statistics and Computing Ltd, 2009

United States Tobacco Use Supplement to the Current Population Survey 1992-1993 as it appears in P.N. Lee Statistics and Computing Ltd. International Mortality and Smoking Statistics Version 4.04. Sutton, United Kingdom: P.N. Lee Statistics and Computing Ltd, 2009

United States Tobacco Use Supplement to the Current Population Survey 1998-1999 as it appears in P.N. Lee Statistics and Computing Ltd. International Mortality and Smoking Statistics Version 4.04. Sutton, United Kingdom: P.N. Lee Statistics and Computing Ltd, 2009

United States Tobacco Use Supplement to the Current Population Survey 2001-2002 as it appears in P.N. Lee Statistics and Computing Ltd. International Mortality and Smoking Statistics Version 4.04. Sutton, United Kingdom: P.N. Lee Statistics and Computing Ltd, 2009

United States Tobacco Use Supplement to the Current Population Survey 2003 as it appears in P.N. Lee Statistics and Computing Ltd. International Mortality and Smoking Statistics Version 4.04. Sutton, United Kingdom: P.N. Lee Statistics and Computing Ltd, 2009

University of Tampere. Finland Adolescent Health and Lifestyle Survey 2001

University of Tampere. Finland Adolescent Health and Lifestyle Survey 2003

University of Tampere. Finland Adolescent Health and Lifestyle Survey 2005

University of Tampere. Finland Adolescent Health and Lifestyle Survey 2007

University of Tampere. Finland Adolescent Health and Lifestyle Survey 2009

University of Tampere. Finland Adolescent Health and Lifestyle Survey 2011

University of Tampere. Finland Adolescent Health and Lifestyle Survey 2013

University of Zurich. Prevalence of Tobacco Use From 2001 to 2005

University of Zurich. Tobacco Monitoring: A Survey of Tobacco Use in Switzerland

Van Reek J, Knibbe R, van Iwaarden T. Policy relevance of a survey on smoking and drinking behaviour among Dutch school children. Health Policy. 1991; 18(3): 261–8

White V, Hill D, Siahpush M, Bobevski I. How has the prevalence of cigarette smoking changed among Australian adults? Trends in smoking prevalence between 1980 and 1932. Tob Control. 2003; 12(Suppl 2): ii67-ii74

World Health Organization (WHO). Spain World Health Survey 2002-2003. Geneva, Switzerland: World Health Organization (WHO), 2005

World Health Organization (WHO). Uruguay World Health Survey 2002-2003. Geneva, Switzerland: World Health Organization (WHO), 2005

World Health Organization (WHO). WHO Report on the Global Tobacco Epidemic 2009. Geneva, Switzerland: World Health Organization (WHO), 2009

World Health Organization Regional Office for Europe (EURO-WHO). Health Behaviour in School-aged Children: WHO Collaborative Cross-National survey/study (HBSC) 2001

World Health Organization Regional Office for Europe (WHO/Europe). Austria Countrywide Integrated Noncommunicable Diseases Intervention Program 1991

World Health Organization Regional Office for Europe (WHO/Europe). Health and Health Behaviour Among Young People: Health Behaviour in School-Aged Children: A WHO Cross National Study (HBSC) International Report. Copenhagen, Denmark: World Health Organization Regional Office for Europe (WHO/Europe), 2000

World Health Organization Regional Office for Europe (WHO/Europe). Inequalities in Young People's Health: HBSC International Report from the 2005-2006 Survey. Copenhagen, Denmark: World Health Organization Regional Office for Europe (WHO/Europe), 2008

World Health Organization Regional Office for Europe (WHO/Europe). Social Determinants of Health and Well-being Among Young People: Health Behaviour in School-aged Children (HBSC) Study: International Report from the 2009/2010 Survey. Copenhagen, Denmark: World Health Organization Regional Office for Europe (WHO/Europe), 2012

World Health Organization Regional Office for Europe (WHO/Europe). Young People's Health in Context. Health Behaviour in School-aged Children (HBSC) Study: International Report from the 2001-2002 Survey. Copenhagen, Denmark: World Health Organization Regional Office for Europe (WHO/Europe), 2004

Zoller U, Maymon T. Smoking Behavior of High School Students in Israel. J Sch Health. 1983; 53(10): 613-7 as it appears in P.N. Lee Statistics and Computing Ltd. International Mortality and Smoking Statistics Version 4.04. Sutton, United Kingdom: P.N. Lee Statistics and Computing Ltd, 2009

High Fasting Plasma Glucose/Diabetes

A mass survey of diabetes mellitus in a population of 300,000 in 14 provinces and municipalities in China (author's transl). Zhonghua Nei Ke Za Zhi. 1981; 20(11): 678-83

Aarhus University, Addiction Switzerland Research Institute, Alcohol Research Group, Public Health Institute, Centre for Addiction and Mental Health (Canada), Centre for Alcohol Policy Research, Turning Point Alcohol and Drug Centre (Australia), Kettil Bruun Society for Social and Epidemiological Research on Alcohol, University of North Dakota. Austria Gender, Alcohol and Culture: An International Study (GENACIS) 1993

Abebe SM, Berhane Y, Worku A, Assefa A. Diabetes mellitus in North West Ethiopia: a community based study. BMC Public Health. 2014; 97

Abu Sayeed M, Mahtab H, Akter Khanam P, Abul Ahsan K, Banu A, Rashid AN, Azad Khan AK. Diabetes and impaired fasting glycemia in the tribes of Khagrachari hill tracts of Bangladesh. Diabetes Care. 2004; 27(5): 1054-9

Abu Sham’a R a. H, Darwazah AK, Kufri FH, Yassin IH, Torok NI. MetS and cardiovascular risk factors among Palestinians of East Jerusalem. East Mediterr Health J. 2009; 15(6): 1464–73

Adams RJ, Appleton S, Wilson DH, Taylor AW, Dal Grande E, Chittleborough C, Gill T, Ruffin R. Population comparison of two clinical approaches to the metabolic syndrome: implications of the new International Diabetes Federation consensus definition. Diabetes Care. 2005; 28(11): 2777-9

Administrative Department of Science, Techonology, and Innovation (Colombia), Center for Development Projects, Pontifical Xavierian University, Ministry of Social Protection (Colombia), Specialized Information Systems. Colombia National Health Survey 2007-2008

Aga Khan University, All India Institute of Medical Sciences, Centre for Chronic Disease Control (India), Emory University, Madras Diabetes Research Foundation, National Institute of Mental Health and Neuro Sciences (India), Public Health Foundation of India. Center for Cardio-Metabolic Risk Reduction in South Asia Surveillance Study 2010-2011

Aguilar-Salinas CA, Rojas R, Gómez-Pérez FJ, García E, Valles V, Ríos-Torres JM, Franco A, Olaiz G, Sepúlveda J, Rull JA. Prevalence and characteristics of early-onset type 2 diabetes in Mexico. Am J Med. 2002; 113(7): 569-74

Akter S, Rahman MM, Abe SK, Sultana P. Prevalence of diabetes and prediabetes and their risk factors among Bangladeshi adults: a nationwide survey. Bull World Health Organ. 2014; 92(3): 204–13

Al Zenki S, Al Omirah H, Al Hooti S, Al Hamad N, Jackson RT, Rao A, Al Jahmah N, Al Obaid I, Al Ghanim J, Al Somaie M, Zaghloul S, Al Othman A. High prevalence of metabolic syndrome among Kuwaiti adults--a wake-up call for public health intervention. Int J Environ Res Public Health. 2012; 9(5): 1984-96

Alarouj M, Bennakhi A, Alnesef Y, Sharifi M, Elkum N. Diabetes and associated cardiovascular risk factors in the State of Kuwait: the first national survey. Int J Clin Pract. 2013; 67(1): 89-96

Al-Baghli NA, Al-Ghamdi AJ, Al-Turki KA, Al Elq AH, El-Zubaier AG, Bahnassy A. Prevalence of diabetes mellitus and impaired fasting glucose levels in the Eastern Province of Saudi Arabia: results of a screening campaign. Singapore Med J. 2010; 51(12): 923-30

Alberts M, Urdal P, Steyn K, Stensvold I, Tverdal A, Nel JH, Steyn NP. Prevalence of cardiovascular diseases and associated risk factors in a rural black population of South Africa. Eur J Cardio Prev Rehabil. 2005; 12(4): 347-54

Al-Daghri NM, Al-Attas OS, Alokail MS, Alkharfy KM, Yousef M, Sabico SL, Chrousos GP. Diabetes mellitus type 2 and other chronic non-communicable diseases in the central region, Saudi Arabia (Riyadh cohort 2): a decade of an epidemic. BMC Med. 2011; 76

Al-Habori M, Al-Mamari M, Al-Meeri A. Type II diabetes mellitus and impaired glucose tolerance in Yemen: prevalence, associated metabolic changes and risk factors. Diabetes Res Clin Pract. 2004; 65(3): 275-81

Alikor CA, Emem-Chioma PC. EPIDEMIOLOGY OF DIABETES AND IMPAIRED FASTING GLUCOSE IN A RURAL COMMUNITY OF NIGERIAN NIGER DELTA REGION. Niger J Med. 2015; 24(2): 114–24

Alkerwi A, Pagny S, Lair M-L, Delagardelle C, Beissel J. Level of unawareness and management of diabetes, hypertension, and dyslipidemia among adults in Luxembourg: findings from ORISCAV-LUX study. PLoS One. 2013; 8(3): e57920

Alkerwi A, Sauvageot N, Donneau A-F, Lair M-L, Couffignal S, Beissel J, Delagardelle C, Wagener Y, Albert A, Guillaume M. First nationwide survey on cardiovascular risk factors in Grand-Duchy of Luxembourg (ORISCAV-LUX). BMC Public Health. 2010; 10(1): 468

Allal-Elasmi M, Haj Taieb S, Hsairi M, Zayani Y, Omar S, Sanhaji H, Jemaa R, Feki M, Elati J, Mebazaa A, Kaabachi N. The metabolic syndrome: prevalence, main characteristics and association with socio-economic status in adults living in Great Tunis. Diabetes Metab. 2010; 36(3): 204–8

Al-Lawati JA, Mohammed AJ, Al-Hinai HQ, Jousilahti P. Prevalence of the metabolic syndrome among Omani adults. Diabetes Care. 2003; 26(6): 1781-5

Al-Nozha MM, Al-Maatouq MA, Al-Mazrou YY, Al-Harthi SS, Arafah MR, Khalil MZ, Khan NB, Al-Khadra A, Al-Marzouki K, Nouh MS, Abdullah M, Attas O, Al-Shahid MS, Al-Mobeireek A. Diabetes mellitus in Saudi Arabia. Saudi Med J. 2004; 25(11): 1603-10

Al-Riyami AA, Afifi MM. Prevalence and correlates of obesity and central obesity among Omani adults. Saudi Med J. 2003; 24(6): 641-6

Al-Rubeaan K, Al-Manaa H, Khoja T, Ahmad N, Al-Sharqawi A, Siddiqui K, AlNaqeb D, Aburisheh K, Youssef A, Al-Batil A, Al-Otaibi M, Ghamdi AA. The Saudi Abnormal Glucose Metabolism and Diabetes Impact Study (SAUDI-DM). Ann Saudi Med. 2014; 34(6): 465–75

American University of Beirut, World Health Organization (WHO). Lebanon STEPS Noncommunicable Disease Risk Factors Survey 2008-2009

Amini M, Afshin-Nia F, Bashardoost N, Aminorroaya A, Shahparian M, Kazemi M. Prevalence and risk factors of diabetes mellitus in the Isfahan city population (aged 40 or over) in 1993. Diabetes Res Clin Pract. 1997; 38(3): 185-90

Amoah AGB, Owusu SK, Adjei S. Diabetes in Ghana: a community based prevalence study in Greater Accra. Diabetes Res Clin Pract. 2002; 56(3): 197-205

Andersson S, Ekman I, Friberg F, Daka B, Lindblad U, Larsson CA. The association between self-rated health and impaired glucose tolerance in Swedish adults: a cross-sectional study. Scand J Prim Health Care. 2013; 31(2): 111–8

Anjana RM, Pradeepa R, Deepa M, Datta M, Sudha V, Unnikrishnan R, Bhansali A, Joshi SR, Joshi PP, Yajnik CS, Dhandhania VK, Nath LM, Das AK, Rao PV, Madhu SV, Shukla DK, Kaur T, Priya M, Nirmal E, Parvathi SJ, Subhashini S, Subashini R, Ali MK, Mohan V. Prevalence of diabetes and prediabetes (impaired fasting glucose and/or impaired glucose tolerance) in urban and rural India: phase I results of the Indian Council of Medical Research-INdia DIABetes (ICMR-INDIAB) study. Diabetologia. 2011; 54(12): 3022-7

Anokute CC. Epidemiologic studies of diabetes mellitus in Saudi Arabia--Part I--Screening of 3158 males in King Saud University. J R Soc Health. 1990; 110(6): 201-3

Arthur FKN, Adu-Frimpong M, Osei-Yeboah J, Mensah FO, Owusu L. The prevalence of metabolic syndrome and its predominant components among pre-and postmenopausal Ghanaian women. BMC Res Notes. 2013; 6: 446

Aschner P, King H, Triana de Torrado M, Rodriguez BM. Glucose intolerance in Colombia. A population-based survey in an urban community. Diabetes Care. 1993; 16(1): 90-3

Asmar R, Vol S, Pannier B, Brisac AM, Tichet J, El Hasnaoui A. High blood pressure and associated cardiovascular risk factors in France. J Hypertens. 2001; 19(10): 1727-32

Aspray TJ, Mugusi F, Rashid S, Whiting D, Edwards R, Alberti KG, Unwin NC, Essential Non-Communicable Disease Health Intervention Project. Rural and urban differences in diabetes prevalence in Tanzania: the role of obesity, physical inactivity and urban living. Trans R Soc Trop Med Hyg. 2000; 94(6): 637-44

Associates for Community and Population Research (ACPR), International Centre for Diarrhoeal Disease Research, Bangladesh (ICDDR,B), MEASURE Evaluation Project, Carolina Population Center, University of North Carolina, National Institute of Population Research and Training (NIPORT). Bangladesh Urban Health Survey 2006

Australian Agency for International Development (AusAID), Fiji School of Medicine, Menzies Center for Population Health Research, University of Tasmania (Australia), Ministry of Health (Fiji), World Health Organization (WHO). Fiji STEPS Noncommunicable Disease Risk Factors Survey 2002

Awad AI, Alsaleh FM. 10-year risk estimation for type 2 diabetes mellitus and coronary heart disease in Kuwait: a cross-sectional population-based study. PLoS One. 2015; 10(1): e0116742

Azimi-Nezhad M, Ghayour-Mobarhan M, Parizadeh MR, Safarian M, Esmaeili H, Parizadeh SM, Khodaee G, Hosseini J, Abasalti Z, Hassankhani B, Ferns G. Prevalence of type 2 diabetes mellitus in Iran and its relationship with gender, urbanisation, education, marital status and occupation. Singapore Med J. 2008; 49(7): 571-6

Azimi-Nezhad M, Herbeth B, Siest G, Dadé S, Ndiaye NC, Esmaily H, Hosseini SJ, Ghayour-Mobarhan M, Visvikis-Siest S. High prevalence of metabolic syndrome in Iran in comparison with France: what are the components that explain this?. Metab Syndr Relat Disord. 2012; 10(3): 181–8

Baker IDI Heart and Diabetes Institute, International Diabetes Institute (IDI), Monash University (Australia), University of Melbourne. Australia Diabetes, Obesity and Lifestyle Study 2011-2012

Balagopal P, Kamalamma N, Patel TG, Misra R. A community-based participatory diabetes prevention and management intervention in rural India using community health workers. Diabetes Educ. 2012; 38(6): 822-34

Balanda KP, Buckley CM, Barron SJ, Fahy LE, Madden JM, Harrington JM, Perry IJ, Kearney PM. Prevalence of diabetes in the Republic of Ireland: results from the National Health Survey (SLAN) 2007. PLoS One. 2013; 8(10): e78406

Bald, NM, Diallo I, Bald, MD, Barry IS, Kaba L, Diallo MM, Kak, A, Camara A, Bah D, Barry MM, Sangar,-Bah M, Maugendre D. Diabetes and impaired fasting glucose in rural and urban populations in Futa Jallon (Guinea): prevalence and associated risk factors. Diabetes Metab. 2007; 33(2): 114-20

Balijepalli C, Losch C, Bramlage P, Erbel R, Humphries KH, Jockel K-H, Moebus S. Percentile distribution of blood pressure readings in 35683 men and women aged 18 to 99 years. J Hum Hypertens. 2014; 28(3): 193–200

Baragou S, Djibril M, Atta B, Damorou F, Pio M, Balogou A. Prevalence of cardiovascular risk factors in an urban area of Togo: a WHO STEPS-wise approach in Lome, Togo. Cardiovasc J Afr. 2012; 23(6): 309-12

Bar-On H, Friedlander Y, Kidron M, Kark JD. Serum glucose and insulin characteristics and prevalence of diabetes mellitus and impaired glucose tolerance in the adult Jewish population of Jerusalem. Nutr Metab Cardiovasc Dis. 1992; 2: 75-8

Basit A, Hydrie MZ, Ahmed K, Hakeem R. Prevalence of diabetes, impaired fasting glucose and associated risk factors in a rural area of Baluchistan province according to new ADA criteria. J Pak Med Assoc. 2002; 52(8): 357-60

Bates CJ, Mansoor MA, Pentieva KD, Hamer M, Mishra GD. Biochemical risk indices, including plasma homocysteine, that prospectively predict mortality in older British people: the National Diet and Nutrition Survey of People Aged 65 Years and Over. Br J Nutr. 2010; 104(6): 893-9

Bautista LE, Oróstegui M, Vera LM, Prada GE, Orozco LC, Herrán OF. Prevalence and impact of cardiovascular risk factors in Bucaramanga, Colombia: results from the Countrywide Integrated Noncommunicable Disease Intervention Programme (CINDI/CARMEN) baseline survey. Eur J Cardiovasc Prev Rehabil. 2006; 13(5): 769-75

Bayindir Cevik A, Metin Karaaslan M, Kocan S, Pekmezci H, Baydur Sahin S, Kirbas A, Ayaz T. Prevalence and screening for risk factors of type 2 diabetes in Rize, Nourtheast Turkey: findings from a population-based study. Prim Care Diabetes. 2015; 10(1): nan

Bayram F, Kocer D, Gundogan K, Kaya A, Demir O, Coskun R, Sabuncu T, Karaman A, Cesur M, Rizzo M, Toth PP, Gedik V. Prevalence of dyslipidemia and associated risk factors in Turkish adults. J Clin Lipidol. 2014; 8(2): 206–16

Ben Romdhane H, Ben Ali S, Aissi W, Traissac P, Aounallah-Skhiri H, Bougatef S, Maire B, Delpeuch F, Achour N. Prevalence of diabetes in Northern African countries: the case of Tunisia. BMC Public Health. 2014; 86

Bengtsson H, Bergqvist D, Ekberg O, Janzon L. A population based screening of abdominal aortic aneurysms (AAA). Eur J Vasc Surg. 1991; 5(1): 53-7

Bermuda Diabetes Association, Bermuda Hospitals Board (BHB), Caribbean Public Health Agency (CARPHA), World Health Organization (WHO). Bermuda STEPS Noncommunicable Disease Risk Factors Survey 2013-2014

Bhansali A, Dhandania VK, Deepa M, Anjana RM, Joshi SR, Joshi PP, Madhu SV, Rao PV, Subashini R, Sudha V, Unnikrishnan R, Das AK, Shukla DK, Kaur T, Mohan V, Pradeepa R. Prevalence of and risk factors for hypertension in urban and rural India: the ICMR-INDIAB study. J Hum Hypertens. 2015; 29(3): 204–9

Bharati DR, Pal R, Kar S, Rekha R, Yamuna TV, Basu M. Prevalence and determinants of diabetes mellitus in Puducherry, South India. J Pharm Bioallied Sci. 2011; 3(4): 513-8

Bhowmik B, Afsana F, My Diep L, Binte Munir S, Wright E, Mahmood S, Khan AKA, Hussain A. Increasing prevalence of type 2 diabetes in a rural bangladeshi population: a population based study for 10 years. Diabetes Metab. 2013; 37(1): 46-53

Bhowmik B, Munir SB, Ahmed KR, Siddiquee T, Diep LM, Wright E, Hassan Z, Debnath PR, Mahtab H, Azad Khan AK, Hussain A. Anthropometric indices of obesity and type 2 diabetes in Bangladeshi population: Chandra Rural Diabetes Study (CRDS). Obes Res Clin Pract. 2014; 8(3): e201–98

Bianchi G, Tripodi G, Manunta P. Na^+, kidney, hypertension and genes: lessons from rats. J Hypertens. 2004; 22(8): 1461-4

Bjerregaard-Andersen M, Hansen L, da Silva LI, Joaquím LC, Hennild DE, Christiansen L, Aaby P, Benn CS, Christensen K, Sodemann M, Jensen DM, Beck-Nielsen H. Risk of metabolic syndrome and diabetes among young twins and singletons in Guinea-bissau. Diabetes Care. 2013; 36(11): 3549-56

Bo S, Ciccone G, Durazzo M, Ghinamo L, Villois P, Canil S, Gambino R, Cassader M, Gentile L, Cavallo-Perin P. Contributors to the obesity and hyperglycemia epidemics. A prospective study in a population-based cohort. Int J Obes (Lond). 2011; 35(11): 1442–9

Bo S, Durazzo M, Guidi S, Carello M, Sacerdote C, Silli B, Rosato R, Cassader M, Gentile L, Pagano G. Dietary magnesium and fiber intakes and inflammatory and metabolic indicators in middle-aged subjects from a population-based cohort. Am J Clin Nutr. 2006; 84(5): 1062-9

Bocquier A, Cortaredona S, Nauleau S, Jardin M, Verger P. Prevalence of treated diabetes: Geographical variations at the small-area level and their association with area-level characteristics. A multilevel analysis in Southeastern France. Diabetes Metab. 2011; 37(1): 39-46

Bonaldi C, Vernay M, Roudier C, Salanave B, Oleko A, Malon A, Castetbon K, Fagot-Campagna A. A first national prevalence estimate of diagnosed and undiagnosed diabetes in France in 18- to 74-year-old individuals: the French Nutrition and Health Survey 2006/2007. Diabet Med. 2011; 28(5): 583–9

Boronat M, Saavedra P, Perez-Martin N, Lopez-Madrazo MJ, Rodriguez-Perez C, Novoa FJ. High levels of lipoprotein(a) are associated with a lower prevalence of diabetes with advancing age: results of a cross-sectional epidemiological survey in Gran Canaria, Spain. Cardiovasc Diabetol. 2012; 11: 81

Börsch-Supan, A. (2013). Survey of Health, Ageing and Retirement in Europe (SHARE) Wave 1. Release version: 2.6.0. SHARE-ERIC. Data set. DOI: 10.6103/SHARE.w1.260

Bourdel-Marchasson I, Dubroca B, Manciet G, Decamps A, Emeriau JP, Dartigues JF. Prevalence of diabetes and effect on quality of life in older French living in the community: the PAQUID Epidemiological Survey. J Am Geriatr Soc. 1997; 45(3): 295-301

Brazilian Institute of Geography and Statistics (IBGE), Ministry of Health (Brazil), Ministry of Planning, Budget, and Management (Brazil). Brazil National Health Survey 2013. Rio de Janeiro, Brazil: Brazilian Institute of Geography and Statistics (IBGE)

Brian G, Ramke J, Maher L, Page A, Szetu J. The prevalence of diabetes among adults aged 40 years and over in Fiji. N Z Med J. 2010; 123(1327): 68-75

Brian G, Ramke J, Page A, Maher L, Szetu J, Qalo Qoqonokana M. The association of diabetes and BMI among Melanesian and Indian Fijians aged ? 40 years. Br J Nutr. 2011; 105(10): 1539–45

Brohall G, Behre CJ, Hulthe J, Wikstrand J, Fagerberg B. Prevalence of diabetes and impaired glucose tolerance in 64-year-old Swedish women: experiences of using repeated oral glucose tolerance tests. Diabetes Care. 2006; 29(2): 363-7

Busselton Population Medical Research Institute (Australia). Australia - Busselton Health Study 1981

Cabrera de León A, Rodríguez Pérez Mdel C, Almeida González D, Domínguez Coello S, Aguirre Jaime A, Brito Díaz B, González Hernández A, Pérez Méndez LI, grupo CDC. Presentación de la cohorte "CDC de Canarias": objetivos, diseño y resultados preliminares. Rev Esp Salud Publica. 2008; 82(5): 519-534

Cai L, Liu A, Zhang L, Li S, Wang P. Prevalence, awareness, treatment, and control of hypertension among adults in Beijing, China. Clin Exp Hypertens. 2012; 34(1): 45–52

Campos H, Mata L, Siles X, Vives M, Ordovas JM, Schaefer EJ. Prevalence of cardiovascular risk factors in rural and urban Costa Rica. Circulation. 1992; 85(2): 648-58

Caribbean Epidemiology Centre (CAREC), Central Statistical Office (Trinidad and Tobago), Ministry of Health (Trinidad and Tobago), Pan American Health Organization (PAHO), University of the West Indies. Trinidad and Tobago STEPS Noncommunicable Disease Risk Factors Survey 2011

Casiglia E, Tikhonoff V, Mazza A, Piccoli A, Pessina AC. Pulse pressure and coronary mortality in elderly men and women from general population. J Hum Hypertens. 2002; 16(9): 611-20

Castetbon K, Vernay M, Malon A, Salanave B, Deschamps V, Roudier C, Oleko A, Szego E, Hercberg S. Dietary intake, physical activity and nutritional status in adults: the French nutrition and health survey (ENNS, 2006-2007). Br J Nutr. 2009; 102(5): 733–43

CBG Health Research Ltd., Ministry of Health (New Zealand), University of Otago (New Zealand). New Zealand Adult Nutrition Survey 2008-2009

Ceesay MM, Morgan MW, Kamanda MO, Willoughby VR, Lisk DR. Prevalence of diabetes in rural and urban populations in southern Sierra Leone: a preliminary survey. Trop Med Int Health. 1997; 2(3): 272-7

Center for the Study of State and Society (CEDES), Ministry of Health and Environment (Argentina), National Institute of Statistics and Censuses (Argentina). Argentina National Survey of Risk Factors 2005

Centers for Disease Control and Prevention (CDC), Institute of Nutrition of Central America and Panama, Pan American Health Organization (PAHO). El Salvador - Santa Tecla Diabetes, Hypertension, and Chronic Disease Risk Factors Survey 2004

Centers for Disease Control and Prevention (CDC), Institute of Nutrition of Central America and Panama, Pan American Health Organization (PAHO). Guatemala - Villa Nueva Diabetes, Hypertension, and Chronic Disease Risk Factors Survey 2002-2003

Centers for Disease Control and Prevention (CDC), Ministry of Health (Jordan), World Health Organization (WHO). Jordan STEPS Noncommunicable Disease Risk Factors Survey 2007

Centers for Disease Control and Prevention (CDC), Ministry of Health (Rwanda), National Institute of Statistics of Rwanda, Rwanda Biomedical Center, World Health Organization (WHO). Rwanda STEPS Noncommunicable Disease Risk Factor Survey 2012-2013

Central Organization for Statistics and Information Technology (Iraq), Ministry of Health (Iraq), World Health Organization (WHO). Iraq STEPS Noncommunicable Disease Risk Factors Survey 2006

Centre for Physical Activity and Health, University of Sydney (Australia), Department of Health and Social Affairs (Micronesia), Fiji School of Medicine, Micronesia Human Resources Development Center, Pohnpei State Department of Health Services, World Health Organization (WHO). Micronesia - Pohnpei STEPS Noncommunicable Disease Risk Factors Survey 2002

Chen HD, Shaw CK, Tseng WP, Chen HI, Lee ML. Prevalence of diabetes mellitus and impaired glucose tolerance in Aborigines and Chinese in eastern Taiwan. Diabetes Res Clin Pract. 1997; 38(3): 199-205

Chen KT, Chen CJ, Gregg EW, Engelgau MM, Narayan KM. Prevalence of type 2 diabetes mellitus in Taiwan: ethnic variation and risk factors. Diabetes Res Clin Pract. 2001; 51(1): 59-66

Chen KT, Chen CJ, Gregg EW, Williamson DF, Narayan KM. High prevalence of impaired fasting glucose and type 2 diabetes mellitus in Penghu Islets, Taiwan: evidence of a rapidly emerging epidemic? Diabetes Res Clin Pract. 1999; 44(1): 59-69

Cheung BMY, Ong KL, Tso AWK, Leung RYH, Cherny SS, Sham PC, Thomas GN, Lam TH, Lam KSL, Investigators of the Hong Kong Cardiovascular Risk Factor Prevalence Study. Relationship of plasma interleukin-6 and its genetic variants with hypertension in Hong Kong Chinese. Am J Hypertens. 2011; 24(12): 1331–7

Chhetri MR, Chapman RS. Prevalence and determinants of diabetes among the elderly population in the Kathmandu Valley of Nepal. Nepal Med Coll J. 2009; 11(1): 34-8

Chiang P-H, Liu C-L, Lin M-H, Peng L-N, Chen L-K, Chen J-D, Hou S-M. Survival benefits of metabolic syndrome among older men aged 75 years and over in Taiwan. J Nutr Health Aging. 2012; 16(6): 520–4

Chin CY, Pengal S. Cardiovascular disease risk in a semirural community in Malaysia. Asia Pac J Public Health. 2009; 21(4): 410-20

Chinese Center for Disease Control and Prevention (CCDC). China Chronic Disease and Risk Factor Surveillance 2010

Chinese Center for Disease Control and Prevention (CCDC). China Chronic Disease and Risk Factor Surveillance 2013

Cho NH. Diabetes burden and prevention in Korea and the Western Pacific Region. Diabetes Res Clin Pract. 2014; S282-7

Chongsuvivatwong V, Yipintsoi T, Suriyawongpaisal P, Cheepudomwit S, Aekplakorn W, Faramnuayphol P, Tatsanavivat P, Kosulwat V, Thamthitiwat S, Nuntawan C. Comparison of cardiovascular risk factors in five regions of Thailand: InterASIA data. J Med Assoc Thai. 2010; 93(1): 17–26

Chou P, Liao MJ, Kuo HS, Hsiao KJ, Tsai ST. A population survey on the prevalence of diabetes in Kin-Hu, Kinmen. Diabetes Care. 1994; 17(9): 1055-8

Christian Medical College, Vellore (India). India Prevalence of Risk Factors for Non-Communicable Diseases in Rural and Urban Tamil Nadu 2010-2012

Chronic Disease Research Centre, University of the West Indies, Ministry of Health (Barbados). Barbados Health of the Nation Survey 2011

Chuuk Department of Health Services (Micronesia), Department of Health and Social Affairs (Micronesia), World Health Organization (WHO). Micronesia - Chuuk STEPS Noncommunicable Disease Risk Factors Survey 2006

Cífková R, Skodová Z, Lánská V, Adámková V, Novozámská E, Jozífová M, Plásková M, Hejl Z, Petrzílková Z, Galovcová M, Palous D. Prevalence, awareness, treatment, and control of hypertension in the Czech Republic. Results of two nationwide cross-sectional surveys in 1997/1998 and 2000/2001, Czech Post-MONICA Study. J Hum Hypertens. 2004; 18(8): 571-9

Cockram CS, Woo J, Lau E, Chan JC, Chan AY, Lau J, Swaminathan R, Donnan SP. The prevalence of diabetes mellitus and impaired glucose tolerance among Hong Kong Chinese adults of working age. Diabetes Res Clin Pract. 1993; 21(1): 67-73

Colagiuri S, Colagiuri R, Na'ati S, Muimuiheata S, Hussain Z, Palu T. The prevalence of diabetes in the Kingdom of Tonga. Diabetes Care. 2002; 25(2): 1378-83

Conen D, Schon T, Aeschbacher S, Pare G, Frehner W, Risch M, Risch L. Genetic and phenotypic determinants of blood pressure and other cardiovascular risk factors (GAPP). Swiss Med Wkly. 2013; 143: w13728

Cook-Huynh M, Ansong D, Steckelberg RC, Boakye I, Seligman K, Appiah L, Kumar N, Amuasi JH. Prevalence of hypertension and diabetes mellitus in adults from a rural community in Ghana. Ethn Dis. 2012; 22(3): 347-52

Cortez-Dias N, Martins S, Belo A, Fiuza M. Prevalence, management and control of diabetes mellitus and associated risk factors in primary health care in Portugal. Rev Port Cardiol. 2010; 29(4): 509-37

Costa B, Barrio F, Cabre J-J, Pinol J-L, Cos F-X, Sole C, Bolibar B, Castell C, Lindstrom J, Barengo N, Tuomilehto J, DE-PLAN-CAT Research Group. Shifting from glucose diagnostic criteria to the new HbA(1c) criteria would have a profound impact on prevalence of diabetes among a high-risk Spanish population. Diabet Med. 2011; 28(10): 1234–7

Croxson SC, Burden AC, Bodington M, Botha JL. The prevalence of diabetes in elderly people. Diabet Med. 1991; 8(1): 28-31

Cumberbatch CG, Younger NO, Ferguson TS, McFarlane SR, Francis DK, Wilks RJ, Tulloch-Reid MK. Reported hours of sleep, diabetes prevalence and glucose control in jamaican adults: analysis from the Jamaica lifestyle survey 2007-2008. Int J Endocrinol. 2011; 716214

Cunningham-Myrie C, Younger-Coleman N, Tulloch-Reid M, McFarlane S, Francis D, Ferguson T, Gordon-Strachan G, Wilks R. Diabetes mellitus in Jamaica: sex differences in burden, risk factors, awareness, treatment and control in a developing country. Trop Med Int Health. 2013; 18(11): 1365-78

Cuong TQ, Dibley MJ, Bowe S, Hanh TT, Loan TT. Obesity in adults: an emerging problem in urban areas of Ho Chi Minh City, Vietnam. Eur J Clin Nutr. 2007; 61(5): 673-81

Dahiru T, Jibo A, Hassan AA, Mande AT. Prevalence of diabetes in a semi-urban community in Northern Nigeria. Niger J Med. 2008; 17(4): 414-6

Daka B, Rosen T, Jansson PA, Larsson CA, Rastam L, Lindblad U. Low sex hormone-binding globulin is associated with hypertension: a cross-sectional study in a Swedish population. BMC Cardiovasc Disord. 2013; 13: 30

Danaei G, Finucane MM, Lu Y, Singh GM, Cowan MJ, Paciorek CJ, Lin JK, Farzadfar F, Khang YH, Stevens GA, Rao M, Ali MK, Riley LM, Robinson CA, Ezzati M, Global Burden of Metabolic Risk Factors of Chronic Diseases Collaborating Group (Blood Glucose). National, regional, and global trends in fasting plasma glucose and diabetes prevalence since 1980: systematic analysis of health examination surveys and epidemiological studies with 370 country-years and 2.7 million participants. Lancet. 2011; 378(9785): 31-40

Daniel CR, Prabhakaran D, Kapur K, Graubard BI, Devasenapathy N, Ramakrishnan L, George PS, Shetty H, Ferrucci LM, Yurgalevitch S, Chatterjee N, Reddy KS, Rastogi T, Gupta PC, Mathew A, Sinha R. A cross-sectional investigation of regional patterns of diet and cardio-metabolic risk in India. Open Nutr J. 2011; 12

Davey A, Lele U, Elias MF, Dore GA, Siegler IC, Johnson MA, Hausman DB, Tenover JL, Poon LW. Diabetes mellitus in centenarians. J Am Geriatr Soc. 2012; 60(3): 468-73

De Macedo ME, Lima MJ, Silva AO, Alcântara P, Ramalhinho V, Carmona J. Prevalence, awareness, treatment and control of hypertension in Portugal. The PAP study. Rev Port Cardiol. 2007; 26(1): 21-39

de Miranda VA, Cruz Filho RA, de Oliveira TS, Moscavitch SD, Kang HC, Miranda Chagas SV, Costa DM, Vianna Araujo D, Garcia Rosa ML. Racial differences in HbA1c: a cross-sectional analysis of a Brazilian public primary care population. Prim Care Diabetes. 2013; 7(2): 135–41

De Nicola L, Donfrancesco C, Minutolo R, Lo Noce C, Palmieri L, De Curtis A, Iacoviello L, Zoccali C, Gesualdo L, Conte G, Vanuzzo D, Giampaoli S, ANMCO-SIN Research Group. Prevalence and cardiovascular risk profile of chronic kidney disease in Italy: results of the 2008-12 National Health Examination Survey. Nephrol Dial Transplant. 2015; 30(5): 806–14

DECODE Study Group. Age- and sex-specific prevalences of diabetes and impaired glucose regulation in 13 European cohorts. Diabetes Care. 2003; 26(1): 61-9

Dedov I, Shestakova M, Benedetti MM, Simon D, Pakhomov I, Galstyan G. Prevalence of type 2 diabetes mellitus (T2DM) in the adult Russian population (NATION study). Diabetes Res Clin Pract. 2016; 115: 90–5

Deepa M, Anjana RM, Manjula D, Narayan KMV, Mohan V. Convergence of prevalence rates of diabetes and cardiometabolic risk factors in middle and low income groups in urban India: 10-year follow-up of the Chennai Urban Population Study. J Diabetes Sci Technol. 2011; 5(4): 918–27

Defay R, Delcourt C, Ranvier M, Lacroux A, Papoz L. Relationships between physical activity, obesity and diabetes mellitus in a French elderly population: the POLA study. Pathologies Oculaires lieés á l' Age. Int J Obes Relat Metab Disord. 2001; 25(4): 512-8

Department of Epidemiology and Public Health, University College London, National Centre for Social Research (NatCen). United Kingdom Health Survey for England 2009-2010 - HSCIC

Department of Health (American Samoa), Monash University (Australia), World Health Organization (WHO). American Samoa STEPS Noncommunicable Disease Risk Factors Survey 2004

Diabetic and Cancer Society of Maldives (DCSM), Indhira Gandhi Memorial Hospital (Maldives), Ministry of Health (Maldives), World Health Organization (WHO). Maldives - Malé STEPS Noncommunicable Disease Risk Factors Survey 2004

Díaz-Díaz O, Hernández M, Collado F, Seuc A, Márquez A. Prevalence of Diabetes Mellitus and Impaired Glucose Tolerance, and Their Changes in 20 Years in a Community of Havana. First Joint Scientic Meeting GLED/EDEG, Scientifc Program, Buenos Aires, Argentina, 1999

Dijkema MBA, Mallant SF, Gehring U, van den Hurk K, Alssema M, van Strien RT, Fischer PH, Nijpels G, Stehouwer CDA, Hoek G, Dekker JM, Brunekreef B. Long-term exposure to traffic-related air pollution and type 2 diabetes prevalence in a cross-sectional screening-study in the Netherlands. Environ Health. 2011; 10: 76

Ding L, Song A, Dai M, Xu M, Sun W, Xu B, Sun J, Wang T, Xu Y, Lu J, Wang W, Bi Y, Ning G. Serum lipoprotein (a) concentrations are inversely associated with T2D, prediabetes, and insulin resistance in a middle-aged and elderly Chinese population. J Lipid Res. 2015; 56(4): 920–6

Dionadji M, Boy B, Mouanodji M, Batakao G. Prevalence of diabetes mellitus in rural areas in Chad. Med Trop (Mars). 2010; 70(4): 414-5

Directorate General of Health-Duhok (Iraq), Kurdistan Regional Government (Iraq), Ministry of Health (Iraq), World Health Organization (WHO). Iraq - Dah?k STEPS Noncommunicable Disease Risk Factors Survey 2003-2004

Divison Garrote JA, Masso Orozco J, Carrion Valero L, Lopez Abril J, Carbayo Herencia JA, Artigao Rodenas LM, Gil Guillen V, Grupo de Enfermedades Vasculares de Albacete (GEVA). [Trends in prevalence of risk factors and global cardiovascular risk in general population of albacete, Spain (1992-94 a 2004-06)]. Rev Esp Salud Publica. 2011; 85(3): 275–84

Dogan N, Toprak D, Demir S. Hypertension prevalence and risk factors among adult population in Afyonkarahisar region: a cross-sectional research. Anatol J Cardiol. 2012; 12(1): 47–52

Dominican Institute of Cardiology (IDC). Dominican Republic Study of Cardiovascular Risk Factors and Metabolic Syndrome 1996-1998

Dorynska A, Polak M, Kozela M, Szafraniec K, Piotrowski W, Bielecki W, Drygas W, Kozakiewicz K, Piwonski J, Tykarski A, Zdrojewski T, Pajak A. Cardiovascular disease (CVD) risk factors in Krakow and in the whole Poland adult population. Results from the WOBASZ study and Polish arm of the HAPIEE project. Przegl Epidemiol. 2015; 69(1): 79–86

Doupis J, Tentolouris N, Mastrokostopoulos A, Kokkinos A, Doupis C, Zdrava A, Kafantogias A. Prevalence of type 2 diabetes in the southwest Albanian adult population. Rural Remote Health. 2007; 7(2): 744

Drivsholm T, Ibsen H, Schroll M, Davidsen M, Borch-Johnsen K. Increasing prevalence of diabetes mellitus and impaired glucose tolerance among 60-year-old Danes. Diabet Med. 2001; 18(2): 126-32

Droumaguet C, Balkau B, Simon D, Caces E, Tichet J, Charles MA, Eschwege E, DESIR Study Group. Use of HbA1c in predicting progression to diabetes in French men and women: data from an Epidemiological Study on the Insulin Resistance Syndrome (DESIR). Diabetes Care. 2006; 29(7): 1619–25

Ducorps M, Baleynaud S, Mayaudon H, Castagne C, Bauduceau B. A prevalence survey of diabetes in Mauritania. Diabetes Care. 1996; 19(7): 761-3

Duda RB, Anarfi JK, Adanu RMK, Seffah J, Darko R, Hill AG. The health of the “older women” in Accra, Ghana: results of the Women’s Health Study of Accra. J Cross Cult Gerontol. 2011; 26(3): 299–314

Dunstan DW, Zimmet PZ, Welborn TA, Cameron AJ, Shaw J, de Courten M, Jolley D, McCarty DJ. The Australian Diabetes, Obesity and Lifestyle Study (AusDiab)--methods and response rates. Diabetes Res Clin Pract. 2002; 57(2): 119-29

Echouffo-Tcheugui JB, Dzudie A, Epacka ME, Choukem SP, Doualla MS, Luma H, Kengne AP. Prevalence and determinants of undiagnosed diabetes in an urban sub-Saharan African population. Prim Care Diabetes. 2012; 6(3): 229–34

Eggertsen R, Lapidus L, Lindstedt G, Nilsson T, Nyström E. [A study of 56-65 years old persons in Mölnlycke. No association between Helicobacter and heart disease or thyroid disorder]. Lakartidningen. 2002; 99(6): 508-9

Eglit T, Rajasalu T, Lember M. Prevalence of diabetes and impaired glucose regulation in Estonia. Diabet Med. 2011; 28(4): 504-5

Ejim EC, Onwubere BJ, Okafor CI, Ulasi II, Emehel A, Onyia U, Akabueze J, Mendis S. Cardiovascular risk factors in middle-aged and elderly residents in South-East Nigeria: the influence of urbanization. Niger J Med. 2013; 22(4): 286–91

Elbagir MN, Eltom MA, Elmahadi EM, Kadam IM, Berne C. A high prevalence of diabetes mellitus and impaired glucose tolerance in the Danagla community in northern Sudan. Diabet Med. 1998; 15(2): 164-9

Elbagir MN, Eltom MA, Elmahadi EM, Kadam IM, Berne C. A population-based study of the prevalence of diabetes and impaired glucose tolerance in adults in northern Sudan. Diabetes Care. 1996; 19(10): 1126-8

El-Hazmi MA, Al-Swailem A, Warsy AS, Al-Sudairy F, Sulaimani R, Al-Swailem A, Al-Meshari A. The prevalence of diabetes mellitus and impaired glucose tolerance in the population of Riyadh. Ann Saudi Med. 1995; 15(6): 598-601

El-Hazmi MA, Warsy AS, Al-Swailem AR, Al-Swailem AM, Sulaimani R, Al-Meshari AA. Diabetes mellitus and impaired glucose tolerance in Saudi Arabia. Ann Saudi Med. 1996; 16(4): 381-5

Escobedo J, Buitron LV, Velasco MF, Ramirez JC, Hernandez R, Macchia A, Pellegrini F, Schargrodsky H, Boissonnet C, Champagne BM. High prevalence of diabetes and impaired fasting glucose in urban Latin America: the CARMELA Study. Diabet Med. 2009; 26(9): 864-71

Espelt A, Goday A, Franch J, Borrell C. Validity of self-reported diabetes in health interview surveys for measuring social inequalities in the prevalence of diabetes. J Epidemiol Community Health. 2012; 66(7): e15

Evaristo-Neto AD, Foss-Freitas MC, Foss MC. Prevalence of diabetes mellitus and impaired glucose tolerance in a rural community of Angola. Diabetol Metab Syndr. 2010; 63

Faeh D, William J, Tappy L, Ravussin E, Bovet P. Prevalence, awareness and control of diabetes in the Seychelles and relationship with excess body weight. BMC Public Health. 2007; 163

Fall CHD, Sachdev HS, Osmond C, Lakshmy R, Biswas SD, Prabhakaran D, Tandon N, Ramji S, Reddy KS, Barker DJP, Bhargava SK. Adult metabolic syndrome and impaired glucose tolerance are associated with different patterns of BMI gain during infancy: Data from the New Delhi Birth Cohort. Diabetes Care. 2008; 31(12): 2349-56

Fano V, Pezzotti P, Gnavi R, Bontempi K, Miceli M, Pagnozzi E, Giarrizzo ML, Fortino A. The role of socio-economic factors on prevalence and health outcomes of persons with diabetes in Rome, Italy. Eur J Public Health. 2013; 23(6): 991-7

Farzadfar F, Murray CJL, Gakidou E, Bossert T, Namdaritabar H, Alikhani S, Moradi G, Delavari A, Jamshidi H, Ezzati M. Effectiveness of diabetes and hypertension management by rural primary health-care workers (Behvarz workers) in Iran: a nationally representative observational study. Lancet. 2012; 379(9810): 47–54

Fava C, Sjogren M, Montagnana M, Danese E, Almgren P, Engstrom G, Nilsson P, Hedblad B, Guidi GC, Minuz P, Melander O. Prediction of blood pressure changes over time and incidence of hypertension by a genetic risk score in Swedes. Hypertension. 2013; 61(2): 319–26

Fawwad A, Alvi SFD, Basit A, Ahmed K, Ahmedani MY, Hakeem R. Changing pattern in the risk factors for diabetes in young adults from the rural area of Baluchistan. J Pak Med Assoc. 2013; 63(9): 1089–93

Federal Ministry of Health (Sudan), World Health Organization (WHO). Sudan - Khartoum STEPS Noncommunicable Disease Risk Factors Survey 2005-2006

Félix-Redondo FJ, Baena-Díez JM, Grau M, Tormo MÁ, Fernández-Bergés D. Prevalence of obesity and cardiovascular risk in the general population of a health area in Extremadura (Spain): the Hermex study. Endocrinol Nutr. 2012; 59(3): 160–8

Ferguson TS, Younger N, Tulloch-Reid MK, Lawrence-Wright MB, Forrester TE, Cooper RS, Van den Broeck J, Wilks RJ. Progression from prehypertension to hypertension in a Jamaican cohort: incident hypertension and its predictors. West Indian Med J. 2010; 59(5): 486–93

Ferguson TS, Younger NOM, Tulloch-Reid MK, Wright MBL, Ward EM, Ashley DE, Wilks RJ. Prevalence of prehypertension and its relationship to risk factors for cardiovascular disease in Jamaica: analysis from a cross-sectional survey. BMC Cardiovasc Disord. 2008; 8: 20

Ferland A, Lamarche B, Chateau-Degat M-L, Counil E, Anassour-Laouan-Sidi E, Abdous B, Dewailly E. Dairy product intake and its association with body weight and cardiovascular disease risk factors in a population in dietary transition. J Am Coll Nutr. 2011; 30(2): 92–9

Fernando DJ, Siribaddana S, de Silva D. Impaired glucose tolerance and diabetes mellitus in a suburban Sri Lankan community. Postgrad Med J. 1994; 70(823): 347-9

Ferrer A, Padrós G, Formiga F, Rojas-Farreras S, Perez JM, Pujol R. Diabetes mellitus: prevalence and effect of morbidities in the oldest old. The Octabaix study. J Am Geriatr Soc. 2012; 60(3): 462-7

Fiji School of Medicine, Menzies Center for Population Health Research, University of Tasmania (Australia), Ministry of Health (Marshall Islands), World Health Organization (WHO). Marshall Islands STEPS Noncommunicable Disease Risk Factors Survey 2002

Fiji School of Medicine, Ministry of Health and Medical Services (Solomon Islands), World Health Organization (WHO). Solomon Islands STEPS Noncommunicable Disease Risk Factors Survey 2005-2006

Firmann M, Marques-Vidal P, Paccaud F, Mooser V, Rodondi N, Waeber G, Vollenweider P. Prevalence, treatment and control of dyslipidaemia in Switzerland: still a long way to go. Eur J Cardiovasc Prev Rehabil. 2010; 17(6): 682–7

Florez H, Silva E, Fernández V, Ryder E, Sulbarán T, Campos G, Calmón G, Clavel E, Castillo-Florez S, Goldberg R. Prevalence and risk factors associated with the metabolic syndrome and dyslipidemia in White, Black, Amerindian and Mixed Hispanics in Zulia State, Venezuela. Diabetes Res Clin Pract. 2005; 69(1): 63-77

Forouhi NG, Luan J, Hennings S, Wareham NJ. Incidence of Type 2 diabetes in England and its association with baseline impaired fasting glucose: the Ely study 1990-2000. Diabet Med. 2007; 24(2): 200-7

Freitas MPD, Loyola Filho AI de, Lima-Costa MF. Birth cohort differences in cardiovascular risk factors in a Brazilian population of older elderly: the Bambuí Cohort Study of Aging (1997 and 2008). Cad Saude Publica. 2011; S409-417

French Institute of Health and Medical Research (INSERM), University of Victor Segalen Bordeaux (France). France Three-City Cohort Study 1999-2001

Fuh JL, Wang SJ, Hwu CM, Lu SR. Glucose tolerance status and cognitive impairment in early middle-aged women. Diabet Med. 2007; 24(7): 788-91

Gakidou E, Mallinger L, Abbott-Klafter J, Guerrero R, Villalpando S, Ridaura RL, Aekplakorn W, Naghavi M, Lim S, Lozano R, Murray CJ. Management of diabetes and associated cardiovascular risk factors in seven countries: a comparison of data from national health examination surveys. Bull World Health Organ. 2011; 89(3): 172-83

Gall SL, Jamrozik K, Blizzard L, Dwyer T, Venn A. Healthy lifestyles and cardiovascular risk profiles in young Australian adults: the Childhood Determinants of Adult Health Study. Eur J Cardiovasc Prev Rehabil. 2009; 16(6): 684–9

Gao WG, Dong YH, Pang ZC, Nan HR, Zhang L, Wang SJ, Ren J, Ning F, Qiao Q. Increasing trend in the prevalence of Type 2 diabetes and pre-diabetes in the Chinese rural and urban population in Qingdao, China. Diabet Med. 2009; 26(12): 1220-7

Gao WG, Qiao Q, Pitkaniemi J, Wild S, Magliano D, Shaw J, Soderberg S, Zimmet P, Chitson P, Knowlessur S, Alberti G, Tuomilehto J. Risk prediction models for the development of diabetes in Mauritian Indians. Diabet Med. 2009; 26(10): 996–1002

Gardete-Correia L, Boavida JM, Raposo JF, Mesquita AC, Fona C, Carvalho R, Massano-Cardoso S. First diabetes prevalence study in Portugal: PREVADIAB study. Diabet Med. 2010; 27(8): 879-81

Gatling W, Budd S, Walters D, Mullee MA, Goddard JR, Hill RD. Evidence of an increasing prevalence of diagnosed diabetes mellitus in the Poole area from 1983 to 1996. Diabet Med. 1998; 15(12): 1015-21

Gause-Nilsson I, Gherman S, Kumar Dey D, Kennerfalk A, Steen B. Prevalence of metabolic syndrome in an elderly Swedish population. Acta Diabetol. 2006; 43(4): 120-6

Ghana Health Service, World Health Organization (WHO). Ghana - Greater Accra Region STEPS Noncommunicable Disease Risk Factors Survey 2006

Gharbi M, Akrout M, Zouari B. Prevalence and risk factors of non-insulin-dependent diabetes mellitus in the rural and urban population of Tunisia. Rev Epidemiol Sante Publique. 2002; 50(4): 349-55

Giri BR, Sharma KP, Chapagai RN, Palzom D. Diabetes and hypertension in urban bhutanese men and women. Indian J Community Med. 2013; 38(3): 138-43

Glumer C, Jorgensen T, Borch-Johnsen K, Inter99 study. Prevalences of diabetes and impaired glucose regulation in a Danish population: the Inter99 study. Diabetes Care. 2003; 26(8): 2335–40

Golozar A, Khademi H, Kamangar F, Poutschi H, Islami F, Abnet CC, Freedman ND, Taylor PR, Pharoah P, Boffetta P, Brennan PJ, Dawsey SM, Malekzadeh R, Etemadi A. Diabetes mellitus and its correlates in an Iranian adult population. PLoS One. 2011; 6(10): e26725

Gomez-Huelgas R, Mancera-Romero J, Bernal-Lopez MR, Jansen-Chaparro S, Baca-Osorio AJ, Toledo E, Perez-Gonzalez R, Guijarro-Merino R, Tinahones FJ, Martinez-Gonzalez MA. Prevalence of cardiovascular risk factors in an urban adult population from southern Spain. IMAP Study. Int J Clin Pract. 2011; 65(1): 35–40

Gosse P, Dauphinot V, Roche F, Pichot V, Celle S, Barthelemy J-C. Prevalence of clinical and ambulatory hypertension in a population of 65-year-olds: the PROOF study. J Clin Hypertens (Greenwich). 2010; 12(3): 160–5

Gourdy P, Ruidavets JB, Ferrieres J, Ducimetiere P, Amouyel P, Arveiler D, Cottel D, Lamamy N, Bingham A, Hanaire-Broutin H; MONICA Study. Prevalence of type 2 diabetes and impaired fasting glucose in the middle-aged population of three French regions - the MONICA study 1995-97. Diabetes Metab. 2001; 27(3): 347-58

Gouveri ET, Tzavara C, Drakopanagiotakis F, Tsaoussoglou M, Marakomichelakis GE, Tountas Y, Diamantopoulos EJ. Mediterranean diet and metabolic syndrome in an urban population: the Athens Study. Nutr Clin Pract. 2011; 26(5): 598–606

Grabe HJ, Schwahn C, Barnow S, Spitzer C, John U, Freyberger HJ, Schminke U, Felix S, Völzke H. Alexithymia, hypertension, and subclinical atherosclerosis in the general population. J Psychosom Res. 2010; 68(2): 139–47

Graciani A, Leon-Munoz LM, Guallar-Castillon P, Rodriguez-Artalejo F, Banegas JR. Cardiovascular health in a southern Mediterranean European country: a nationwide population-based study. Circ Cardiovasc Qual Outcomes. 2013; 6(1): 90–8

Graff-Iversen S, Jenum AK, Grøtvedt L, Bakken B, Selmer RM, Søgaard AJ. Risikofaktorer for hjerteinfarkt, hjerneslag og diabetes i Norge. Tidsskr Nor Laegeforen. 2007; 127(19): 2537-41

Grandinetti A, Kaholokula JK, Theriault AG, Mor JM, Chang HK, Waslien C. Prevalence of diabetes and glucose intolerance in an ethnically diverse rural community of Hawaii. Ethn Dis. 2007; 17(2): 250-5

Gregory CO, Dai J, Ramirez-Zea M, Stein AD. Occupation is more important than rural or urban residence in explaining the prevalence of metabolic and cardiovascular disease risk in Guatemalan adults. J Nutr. 2007; 137(5): 1314-9

Gudmundsdottir SL, Flanders WD, Augestad LB. Physical activity and cardiovascular risk factors at menopause: the Nord-Trondelag health study. Climacteric. 2013; 16(4): 438–46

Gunaid AA, Assabri AM. Prevalence of type 2 diabetes and other cardiovascular risk factors in a semirural area in Yemen. East Mediterr Health J. 2008; 14(1): 42-56

Gundogan K, Bayram F, Capak M, Tanriverdi F, Karaman A, Ozturk A, Altunbas H, Gokce C, Kalkan A, Yazici C. Prevalence of metabolic syndrome in the Mediterranean region of Turkey: evaluation of hypertension, diabetes mellitus, obesity, and dyslipidemia. Metab Syndr Relat Disord. 2009; 7(5): 427–34

Gupta A, Gupta R, Sarna M, Rastogi S, Gupta VP, Kothari K. Prevalence of diabetes, impaired fasting glucose and insulin resistance syndrome in an urban Indian population. Diabetes Res Clin Pract. 2003; 61(1): 69-76

Gupta R, Misra A, Vikram NK, Kondal D, Gupta SS, Agrawal A, Pandey RM. Younger age of escalation of cardiovascular risk factors in Asian Indian subjects. BMC Cardiovasc Disord. 2009; 28

Haba-Rubio J, Marques-Vidal P, Andries D, Tobback N, Preisig M, Vollenweider P, Waeber G, Luca G, Tafti M, Heinzer R. Objective sleep structure and cardiovascular risk factors in the general population: the HypnoLaus Study. Sleep. 2015; 38(3): 391–400

Hadaegh F, Shafiee G, Hatami M, Azizi F. Systolic and diastolic blood pressure, mean arterial pressure and pulse pressure for prediction of cardiovascular events and mortality in a Middle Eastern population. Blood Press. 2012; 21(1): 12–8

Haghighatdoost F, Sarrafzadegan N, Mohammadifard N, Sajjadi F, Maghroon M, Boshtam M, Alikhasi H, Azadbakht L. Healthy eating index and cardiovascular risk factors among Iranians. J Am Coll Nutr. 2013; 32(2): 111–21

Hairong Nan, Zengchang Pang, Shaojie Wang, Weiguo Gao, Lei Zhang, Jie Ren, Feng Ning, Tuomilehto J, Qing Qiao. Serum uric acid, plasma glucose and diabetes. Diab Vasc Dis Res. 2010; 7(1): 40–6

Hajat C, Harrison O, Al Siksek Z. Diagnostic testing for diabetes using HbA(1c) in the Abu Dhabi population: Weqaya: the Abu Dhabi cardiovascular screening program. Diabetes Care. 2011; 34(11): 2400–2

Hamer M, Kengne AP, Batty GD, Cooke D, Stamatakis E. Temporal trends in diabetes prevalence and key diabetes risk factors in Scotland, 2003-2008. Diabet Med. 2011; 28(5): 595-8

Hammami S, Mehri S, Hajem S, Koubaa N, Souid H, Hammami M. Prevalence of diabetes mellitus among non institutionalized elderly in Monastir City. BMC Endocr Disord. 2012; 15

Han L, Wang Y, Li J, Zhang X, Bian C, Wang H, Du S, Suo L. Gender differences in associations of serum ferritin and diabetes, metabolic syndrome, and obesity in the China Health and Nutrition Survey. Mol Nutr Food Res. 2014; 58(11): 2189–95

Hanninen M-RA, Niiranen TJ, Puukka PJ, Johansson J, Jula AM. Prognostic significance of masked and white-coat hypertension in the general population: the Finn-Home Study. J Hypertens. 2012; 30(4): 705–12

Hansen TW, Staessen JA, Torp-Pedersen C, Rasmussen S, Li Y, Dolan E, Thijs L, Wang J-G, O Brien E, Ibsen H. Ambulatory arterial stiffness index predicts stroke in a general population. J Hypertens. 2006; 24(11): 2247-54

Harati H, Hadaegh F, Saadat N, Azizi F. Population-based incidence of Type 2 diabetes and its associated risk factors: results from a six-year cohort study in Iran. BMC Public Health. 2009; 186

Hayes L, Hawthorne G, Unwin N. Undiagnosed diabetes in the over-60s: performance of the Association of Public Health Observatories (APHO) Diabetes Prevalence Model in a general practice. Diabet Med. 2012; 29(1): 115–20

He Y-H, Chen Y-C, Jiang G-X, Huang H-E, Li R, Li X-Y, Ning G, Cheng Q. Evaluation of anthropometric indices for metabolic syndrome in Chinese adults aged 40 years and over. Eur J Nutr. 2012; 51(1): 81–7

He Y-H, Jiang G-X, Yang Y, Huang H-E, Li R, Li X-Y, Ning G, Cheng Q. Obesity and its associations with hypertension and type 2 diabetes among Chinese adults age 40 years and over. Nutrition. 2009; 25(11-12): 1143–9

Health Canada, Public Health Agency of Canada, Statistics Canada. Canada Health Measures Survey 2007-2009. Ottawa, Canada: Statistics Canada

Health Canada, Public Health Agency of Canada, Statistics Canada. Canada Health Measures Survey 2009-2011. Ottawa, Canada: Statistics Canada

Health of Populations in Transition (HoPiT) Research Group (Cameroon), Ministry of Public Health (Cameroon), World Diabetes Foundation (WDF), World Health Organization (WHO). Cameroon STEPS Noncommunicable Disease Risk Factors Survey 2003

Hellgren MI, Petzold M, Bjorkelund C, Wedel H, Jansson P-A, Lindblad U. Feasibility of the FINDRISC questionnaire to identify individuals with impaired glucose tolerance in Swedish primary care. A cross-sectional population-based study. Diabet Med. 2012; 29(12): 1501–5

Henry RMA, Ferreira I, Dekker JM, Nijpels G, Scheffer PG, Stehouwer CDA. The metabolic syndrome in elderly individuals is associated with greater muscular, but not elastic arterial stiffness, independent of low-grade inflammation, endothelial dysfunction or insulin resistance--The Hoorn Study. J Hum Hypertens. 2009; 23(11): 718–27

Hernández RE, Cardonnet LJ, Libman C, Gagliardino JJ. Prevalence of diabetes and obesity in an urban population of Argentina. Diabetes Res Clin Pract. 1987; 3(5): 277-83

Hightower JD, Hightower CM, Vázquez BYS, Intaglietta M. Incident prediabetes/diabetes and blood pressure in urban and rural communities in the Democratic Republic of Congo. Vasc Health Risk Manag. 2011; 7: 483–9

Hildrum B, Mykletun A, Hole T, Midthjell K, Dahl AA. Age-specific prevalence of the metabolic syndrome defined by the International Diabetes Federation and the National Cholesterol Education Program: the Norwegian HUNT 2 study. BMC Public Health. 2007; 7: 220

Hiltunen L, Luukinen H, Koski K, Kivel„ SL. Prevalence of diabetes mellitus in an elderly Finnish population. Diabet Med. 1994; 11(3): 241-9

Hirani V. Relationship between vitamin D and hyperglycemia in older people from a nationally representative population survey. J Am Geriatr Soc. 2011; 59(10): 1786–92

Hollman G, Kristenson M. The prevalence of the metabolic syndrome and its risk factors in a middle-aged Swedish population--mainly a function of overweight?. Eur J Cardiovasc Nurs. 2008; 7(1): 21-6

Holt RIG, Phillips DIW, Jameson KA, Cooper C, Dennison EM, Peveler RC, Hertfordshire Cohort Study Group. The relationship between depression, anxiety and cardiovascular disease: findings from the Hertfordshire Cohort Study. J Affect Disord. 2013; 150(1): 84–90

Honorary Commission for Cardiovascular Health (Uruguay). Uruguay Cardiovascular Disease, Epidemiology, and Statistics 1990-1992. Montevideo, Uruguay: Honorary Commission for Cardiovascular Health (Uruguay), 1996

Hou X, Lu J, Weng J, Ji L, Shan Z, Liu J, Tian H, Ji Q, Zhu D, Ge J, Lin L, Chen L, Guo X, Zhao Z, Li Q, Zhou Z, Shan G, Yang Z, Yang W, Jia W, China National Diabetes and Metabolic Disorders Study Group. Impact of waist circumference and body mass index on risk of cardiometabolic disorder and cardiovascular disease in Chinese adults: a national diabetes and metabolic disorders survey. PLoS One. 2013; 8(3): e57319

Hu Y, Teng W, Liu L, Chen K, Liu L, Hua R, Chen J, Zhou Y, Chen L. Prevalence and risk factors of diabetes and diabetic retinopathy in Liaoning province, China: a population-based cross-sectional study. PLoS One. 2015; 10(3): e0121477

Huffman MD, Prabhakaran D, Osmond C, Fall CHD, Tandon N, Lakshmy R, Ramji S, Khalil A, Gera T, Prabhakaran P, Biswas SKD, Reddy KS, Bhargava SK, Sachdev HS, New Delhi Birth Cohort. Incidence of cardiovascular risk factors in an Indian urban cohort results from the New Delhi birth cohort. J Am Coll Cardiol. 2011; 57(17): 1765-74

Human Sciences Research Council, South African Medical Research Council. South Africa National Health and Nutrition Examination Survey 2012

Hussain A, Rahim MA, Azad Khan AK, Ali SM, Vaaler S. Type 2 diabetes in rural and urban population: diverse prevalence and associated risk factors in Bangladesh. Diabet Med. 2005; 22(7): 931-6

Husseini A, Abdul-Rahim H, Awartani F, Jervell J, Bjertness E. Prevalence of diabetes mellitus and impaired glucose tolerance in a rural Palestinian population. East Mediterr Health J. 2000; 6(5-6): 1039-45

Hwang JH, Kam S, Shin J, Kim J-Y, Lee K-E, Kwon G-H, Chun B-Y, Chae SC, Yang DH, Park HS, Hwang T-Y. Incidence of metabolic syndrome and relative importance of five components as a predictor of metabolic syndrome: 5-year follow-up study in Korea. J Korean Med Sci. 2013; 28(12): 1768–73

ICF International, Ministry of Health and Social Services (Namibia), Namibia Institute of Pathology, Namibia Statistics Agency. Namibia Demographic and Health Survey 2013. Fairfax, United States: ICF International, 2015

ICF Macro, Mitra and Associates, National Institute of Population Research and Training (NIPORT). Bangladesh Demographic and Health Survey 2011-2012. Calverton, United States: ICF Macro

Icks A, Kruse J, Dragano N, Broecker-Preuss M, Slomiany U, Mann K, Jöckel KH, Erbel R, Giani G, Moebus S; Heinz Nixdorf Recall Study Investigator Group. Are symptoms of depression more common in diabetes? Results from the Heinz Nixdorf Recall study. Diabet Med. 2008; 25(11): 1330-6

Ilow R, Regulska-Ilow B, Rozanska D, Kowalisko A, Biernat J. Prevalence of metabolic syndrome among 40- and 50-year-old inhabitants of Wroclaw, Poland. Ann Agric Environ Med. 2012; 19(3): 551–6

International Diabetes Institute (IDI). Australia Diabetes, Obesity and Lifestyle Study 1999-2000. Melbourne, Australia: International Diabetes Institute (IDI)

International Food Policy Research Institute (IFPRI), University of Natal, University of Wisconsin, Southern Africa Labour Development Research Unit (SALDRU), School of Economics, University of Cape Town. South Africa KwaZulu-Natal Income Dynamics Study 1993. Durban, South Africa: University of Natal

International Institute for Population Sciences (India). India District Level Household Survey 2012-2014. New Delhi, India: Ministry of Health and Family Welfare (India)

Isara AR, Okundia PO. The burden of hypertension and diabetes mellitus in rural communities in southern Nigeria. Pan Afr Med J. 2015; 103

Jacobs JM, Stessman J, Ein-Mor E, Bursztyn M. Hypertension and 5-year mortality among 85-year-olds: the Jerusalem Longitudinal Study. J Am Med Dir Assoc. 2012; 13(8): 759e1–6

Jadue L, Vega J, Escobar MC, Delgado I, Garrido C, Lastra P, Espejo F, Peruga A. Risk factors for non communicable diseases: methods and global results of the CARMEN program basal survey. Rev Med Chil. 1999; 127(8): 1004-13

Jaipakdee J, Jiamjarasrangsri W, Lohsoonthorn V, Lertmaharit S. Prevalence of metabolic syndrome and its association with serum uric acid levels in Bangkok Thailand. Southeast Asian J Trop Med Public Health. 2013; 44(3): 512–22

Janghorbani M, Amini M. Associations of hip circumference and height with incidence of type 2 diabetes: the Isfahan diabetes prevention study. Acta Diabetol. 2012; 49 Suppl 1: S107–114

Janus ED, Watt NM, Lam KS, Cockram CS, Siu ST, Liu LJ, Lam TH. The prevalence of diabetes, association with cardiovascular risk factors and implications of diagnostic criteria (ADA 1997 and WHO 1998) in a 1996 community-based population study in Hong Kong Chinese. Diabet Med. 2000; 17(10): 741-5

Jeenduang N, Whanmasae S, Seepawin P, Kullabootr S. The prevalence of dyslipidemia among a rural Thai population in the Nakhon Si Thammarat province. J Med Assoc Thai. 2013; 96(8): 992–1000

Jeon JY, Ko SH, Kwon HS, Kim NH, Kim JH, Kim CS, Song KH, Won JC, Lim S, Choi SH, Jang MJ, Kim Y, Oh K, Kim DJ, Cha BY. Prevalence of Diabetes and Prediabetes according to Fasting Plasma Glucose and HbA1c. Diabetes Metab J. 2013; 37(5): 349-57

Jeppesen C, Bjerregaard P, Jorgensen ME. Dietary patterns in Greenland and their relationship with type 2 diabetes mellitus and glucose intolerance. Public Health Nutr. 2014; 17(2): 462–70

Jermendy G, Nadas J, Szigethy E, Szeles G, Nagy A, Hidvegi T, Paragh G, Adany R. Prevalence rate of diabetes mellitus and impaired fasting glycemia in Hungary: cross-sectional study on nationally representative sample of people aged 20-69 years. Croat Med J. 2010; 51(2): 151-6

Jimenez JT, Palacios M, Cañete F, Barriocanal LA, Medina U, Figueredo R, Martinez S, de Melgarejo MV, Weik S, Kiefer R, Alberti KG, Moreno-Azorero R. Prevalence of diabetes mellitus and associated cardiovascular risk factors in an adult urban population in Paraguay. Diabet Med. 1998; 15(4): 334-8

Joint Health Surveys Unit of Social and Community Planning Research and University College London, Health Survey for England, 1994 [computer file]. 4th ed. Colchester, Essex: UK Data Archive [distributor], 26 March 2001. SN: 3640

Joint Health Surveys Unit, University College London and Medical Research Council. Social and Public Health Sciences Unit, Scottish Health Survey, 2003 [computer file]. Colchester, Essex: UK Data Archive [distributor], February 2006. SN: 5318

Jørgensen ME, Borch-Johnsen K, Witte DR, Bjerregaard P. Diabetes in Greenland and its relationship with urbanization. Diabet Med. 2012; 29(6): 755-60

Kabeya Y, Kato M, Isogawa A, Takahashi Y, Matsushita Y, Goto A, Iso H, Inoue M, Mizoue T, Tsugane S, Kadowaki T, Noda M. Descriptive epidemiology of diabetes prevalence and HbA1c distributions based on a self-reported questionnaire and a health checkup in the JPHC diabetes study. J Epidemiol. 2014; 24(6): 460–8

Kadiki OA, Roaed RB. Epidemiological and clinical patterns of diabetes mellitus in Benghazi, Libyan Arab Jamahiriya. East Mediterr Health J. 1999; 5(1): 6-13

Kadiki OA, Roaeid RB. Prevalence of diabetes mellitus and impaired glucose tolerance in Benghazi Libya. Diabetes Metab. 2001; 27(6): 647-54

Kaiser A, Vollenweider P, Waeber G, Marques-Vidal P. Prevalence, awareness and treatment of type 2 diabetes mellitus in Switzerland: the CoLaus study. Diabet Med. 2012; 29(2): 190-7

Kalits I, Podar T. Incidence and prevalence of type 1 (insulin-dependent) diabetes in Estonia in 1988. Diabetologia. 1990; 33(6): 346-9

Kalter-Leibovici O, Chetrit A, Lubin F, Atamna A, Alpert G, Ziv A, Abu-Saad K, Murad H, Eilat-Adar S, Goldbourt U. Adult-onset diabetes among Arabs and Jews in Israel: a population-based study. Diabet Med. 2012; 29(6): 748-54

Kamble P, Deshmukh PR, Garg N. Metabolic syndrome in adult population of rural Wardha, central India. Indian J Med Res. 2010; 132: 701–5

Karalis IK, Alegakis AK, Kafatos AG, Koutis AD, Vardas PE, Lionis CD. Risk factors for ischaemic heart disease in a Cretan rural population: a twelve year follow-up study. BMC Public Health. 2007; 351

Karpati T, Cohen-Stavi CJ, Leibowitz M, Hoshen M, Feldman BS, Balicer RD. Towards a subsiding diabetes epidemic: trends from a large population-based study in Israel. Popul Health Metr. 2014; 12(1): 32

Kasiam LO, Longo-Mbenza B, Nge OA, Kangola KN, Mbungu FS, Milongo DG. Classification and dramatic epidemic of diabetes mellitus in Kinshasa Hinterland: the prominent role of type 2 diabetes and lifestyle changes among Africans. Niger J Med. 2009; 18(3): 311-20

Katchunga P, Masumbuko B, Belma M, Kashongwe Munogolo Z, Hermans MP, M'buyamba-Kabangu JR. Age and living in an urban environment are major determinants of diabetes among South Kivu Congolese adults. Diabetes Metab. 2012; 38(4): 324-31

Katte J-C, Dzudie A, Sobngwi E, Mbong EN, Fetse GT, Kouam CK, Kengne A-P. Coincidence of diabetes mellitus and hypertension in a semi-urban Cameroonian population: a cross-sectional study. BMC Public Health. 2014; 14: 696

Katulanda P, Ranasinghe P, Jayawardena R, Constantine GR, Rezvi Sheriff MH, Matthews DR. The prevalence, predictors and associations of hypertension in Sri Lanka: a cross-sectional population based national survey. Clin Exp Hypertens. 2014; 36(7): 484–91

Kelestimur F, Cetin M, Pa?ao?lu H, Coksevim B, Cetinkaya F, Unlühizarci K, Unal S, Köker AH. The prevalence and identification of risk factors for type 2 diabetes mellitus and impaired glucose tolerance in Kayseri, central Anatolia, Turkey. Acta Diabetol. 1999; 36(1-2): 85-91

Kenya National Bureau of Statistics, Ministry of Health (Kenya), World Health Organization (WHO). Kenya STEPS Noncommunicable Disease Risk Factors Survey 2015

Khader Y, Batieha A, Ajlouni H, El-Khateeb M, Ajlouni K. Obesity in Jordan: prevalence, associated factors, comorbidities, and change in prevalence over ten years. Metab Syndr Relat Disord. 2008; 6(2): 113-20

Khambalia A, Phongsavan P, Smith BJ, Keke K, Dan L, Fitzhardinge A, Bauman AE. Prevalence and risk factors of diabetes and impaired fasting glucose in Nauru. BMC Public Health. 2011; 11: 719

Khan FS, Lotia-Farrukh I, Khan AJ, Siddiqui ST, Sajun SZ, Malik AA, Burfat A, Arshad MH, Codlin AJ, Reininger BM, McCormick JB, Afridi N, Fisher-Hoch SP. The burden of non-communicable disease in transition communities in an Asian megacity: baseline findings from a cohort study in Karachi, Pakistan. PLoS One. 2013; 8(2): e56008

Kheirandish M, Asgari S, Lotfaliany M, Bozorgmanesh M, Saadat N, Tohidi M, Azizi F, Hadaegh F. Secular trends in serum lipid levels of a Middle Eastern adult population; 10 years follow up in Tehran lipid and glucose study. Lipids Health Dis. 2014; 13: 20

Kim JM. Vascular disease/risk and late-life depression in a Korean community population. Br J Psychiatry. 2004; 185(2): 102-7

Kim SR, Han K, Choi JY, Ersek J, Liu J, Jo SJ, Lee KS, Yim HW, Lee WC, Park YG, Lee SH, Park YM. Age- and sex-specific relationships between household income, education, and diabetes mellitus in Korean adults: the Korea National Health and Nutrition Examination Survey, 2008-2010. PLoS One. 2015; 10(1): e0117034

King H, Collins V, King LF, Finch C, Alpers MP. Blood pressure, hypertension and other cardiovascular risk factors in six communities in Papua New Guinea, 1985-1986. P N G Med J. 1994; 37(2): 100-9

King H, Taylor R, Koteka G, Nemaia H, Zimmet P, Bennett PH, Raper LR. Glucose tolerance in Polynesia. Population-based surveys in Rarotonga and Niue. Med J Aust. 1986; 145(10): 505-10

Kobayashi J, Nishimura K, Matoba M, Maekawa N, Mabuchi H. Generation and Gender Differences in the Components Contributing to the Diagnosis of the Metabolic Syndrome According to the Japanese Criteria. Circ J. 2007; 71(11): 1734-7

Kobayashi Y, Niu K, Guan L, Momma H, Guo H, Cui Y, Nagatomi R. Oral health behavior and metabolic syndrome and its components in adults. J Dent Res. 2012; 91(5): 479–84

Komulainen P, Pedersen M, Hanninen T, Bruunsgaard H, Lakka T, Kivipelto M, Hassinen M, Rauramaa T, Pedersen B, Rauramaa R. BDNF is a novel marker of cognitive function in ageing women: The DR's EXTRA Study. Neurobiol Learn Mem. 2008; 90(4): 596-603

Koo BK, Kim EK, Choi H, Park KS, Moon MK. Decreasing trends of the prevalence of diabetes and obesity in Korean women aged 30-59 years over the past decade: results from the Korean National Health and Nutrition Examination Survey, 2001-2010. Diabetes Care. 2013; 36(7): e95-6

Koopman JJE, van Bodegom D, Jukema JW, Westendorp RGJ. Risk of Cardiovascular Disease in a Traditional African Population with a High Infectious Load: A Population-Based Study. PLoS One. 2012; 7(10)

Korea Centers for Disease Control and Prevention. South Korea National Health and Nutrition Examination Survey 1998

Korea Centers for Disease Control and Prevention. South Korea National Health and Nutrition Examination Survey 2001

Korea Centers for Disease Control and Prevention. South Korea National Health and Nutrition Examination Survey 2005

Korea Centers for Disease Control and Prevention. South Korea National Health and Nutrition Examination Survey 2007

Korea Centers for Disease Control and Prevention. South Korea National Health and Nutrition Examination Survey 2008

Korea Centers for Disease Control and Prevention. South Korea National Health and Nutrition Examination Survey 2009

Korea Centers for Disease Control and Prevention. South Korea National Health and Nutrition Examination Survey 2011

Korea Centers for Disease Control and Prevention. South Korea National Health and Nutrition Examination Survey 2012

Korhonen PE, Kautiainen H, Mantyselka P. Screening for cardiovascular risk factors and self-rated health in a community setting: a cross-sectional study in Finland. Br J Gen Pract. 2014; 64(627): e611–5

Kotronen A, Yki-Jarvinen H, Mannisto S, Saarikoski L, Korpi-Hyovalti E, Oksa H, Saltevo J, Saaristo T, Sundvall J, Tuomilehto J, Peltonen M. Non-alcoholic and alcoholic fatty liver disease - two diseases of affluence associated with the metabolic syndrome and type 2 diabetes: the FIN-D2D survey. BMC Public Health. 2010; 10: 237

Kutty VR, Soman CR, Joseph A, Pisharody R, Vijayakumar K. Type 2 diabetes in southern Kerala: variation in prevalence among geographic divisions within a region. Natl Med J India. 2000; 13(6): 287-92

Kuusisto J, Mykkänen L, Pyörälä K, Laakso M. Non-insulin-dependent diabetes and its metabolic control are important predictors of stroke in elderly subjects. Stroke. 1994; 25(6): 1157-64

Kweon S-S, Shin M-H, Park K-S, Nam H-S, Jeong S-K, Ryu S-Y, Chung E-K, Choi J-S. Distribution of the ankle-brachial index and associated cardiovascular risk factors in a population of middle-aged and elderly koreans. J Korean Med Sci. 2005; 20(3): 373-8

Laccetti R, Pota A, Stranges S, Falconi C, Memoli B, Bardaro L, Guida B. Evidence on the prevalence and geographic distribution of major cardiovascular risk factors in Italy. Public Health Nutr. 2013; 16(2): 305–15

Laclaustra M, Ordonez B, Leon M, Andres EM, Cordero A, Pascual-Calleja I, Grima A, Luengo E, Alegria E, Pocovi M, Civeira F, Casasnovas-Lenguas JA. Metabolic syndrome and coronary heart disease among Spanish male workers: a case-control study of MESYAS. Nutr Metab Cardiovasc Dis. 2012; 22(6): 510–6

Lai S-W, Tan C-K. Epidemiology of Hyperglycemia in Elderly Persons. J Gerontol A Biol Sci Med Sci. 2000; 55A(5): M257-9

Lao XQ, Ma WJ, Sobko T, Zhang YH, Xu YJ, Xu XJ, Yu DM, Nie SP, Cai QM, Wei XL, Xia L, Wong MC-S. Dramatic escalation in metabolic syndrome and cardiovascular risk in a Chinese population experiencing rapid economic development. BMC Public Health. 2014; 14: 983

Larsson CA, Kroll L, Bennet L, Gullberg B, Rastam L, Lindblad U. Leisure time and occupational physical activity in relation to obesity and insulin resistance: a population-based study from the Skaraborg Project in Sweden. Metab Clin Exp. 2012; 61(4): 590–8

Laux TS, Bert PJ, Gonzalez M, Unruh M, Aragon A, Lacourt CT. Prevalence of obesity, tobacco use, and alcohol consumption by socioeconomic status among six communities in Nicaragua. Rev Panam Salud Publica. 2012; 32(3): 217–25

Le C, Jun D, Zhankun S, Yichun L, Jie T. Socioeconomic differences in diabetes prevalence, awareness, and treatment in rural southwest China. Trop Med Int Health. 2011; 16(9): 1070-6

Lee H-S, Park Y-M, Kwon H-S, Lee J-H, Park YJ, Lim SY, Lee S-H, Yoon K-H, Son H-Y, Kim DS, Yim HW, Lee W-C. Prevalence, awareness, treatment, and control of hypertension among people over 40 years old in a rural area of South Korea: The Chungju Metabolic Disease Cohort (CMC) Study. Clin Exp Hypertens. 2010; 32(3): 166–78

Lee HY, Won JC, Kang YJ, Yoon SH, Choi EO, Bae JY, Sung MH, Kim H-R, Yang JH, Oh J, Lee YM, Park NH, Ko KS, Rhee BD. Type 2 diabetes in urban and rural districts in Korea: factors associated with prevalence difference. J Korean Med Sci. 2010; 25(12): 1777-83

Lee JH, Yang DH, Park HS, Cho Y, Jun JE, Park WH, Chun BY, Shin J-Y, Shin DH, Lee KS, Kim K-S, Kim K-B, Kim YJ, Chae SC, HYpertension-Diabetes Daegu Initiative Study Investigators. Incidence of hypertension in Korea: 5-year follow-up study. J Korean Med Sci. 2011; 26(10): 1286–92

Lee MY, Kim MY, Kim SY, Kim JH, Kim BH, Shin JY, Shin YG, Yun JH, Ryu SY, Lee TY, Koh SB, Chung CH. Association between alcohol intake amount and prevalence of metabolic syndrome in Korean rural male population. Diabetes Res Clin Pract. 2010; 88(2): 196–202

Lerner AG, Bernabe-Ortiz A, Gilman RH, Smeeth L, Miranda JJ. The "rule of halves" does not apply in Peru: awareness, treatment, and control of hypertension and diabetes in rural, urban, and rural-to-urban migrants. Crit Pathw Cardiol. 2013; 12(2): 53-8

Lessa I, Magalhães L, Araújo MJ, de Almeida Filho N, Aquino E, Oliveira MM. Arterial hypertension in the adult population of Salvador (BA)--Brazil. Arq Bras Cardiol. 2006; 87(6): 747-56

Levitt NS, Katzenellenbogen JM, Bradshaw D, Hoffman MN, Bonnici F. The prevalence and identification of risk factors for NIDDM in urban Africans in Cape Town, South Africa. Diabetes Care. 1993; 16(4): 601-7

Li R, Lu W, Jiang QW, Li YY, Zhao GM, Shi L, Yang QD, Ruan Y, Jiang J, Zhang SN, Xu WH, Zhong WJ. Increasing prevalence of type 2 diabetes in Chinese adults in Shanghai. Diabetes Care. 2012; 35(5): 1028-30

Li W, Wang Y, Chen L, Horswell R, Xiao K, Besse J, Johnson J, Ryan DH, Hu G. Increasing prevalence of diabetes in middle or low income residents in Louisiana from 2000 to 2009. Diabetes Res Clin Pract. 2011; 94(2): 262-8

Lilja M, Eliasson M, Eriksson M, Soderberg S. A rightward shift of the distribution of fasting and post-load glucose in northern Sweden between 1990 and 2009 and its predictors. Data from the Northern Sweden MONICA study. Diabet Med. 2013; 30(9): 1054–62

Lim N-K, Park S-H, Choi S-J, Lee K-S, Park H-Y. A risk score for predicting the incidence of type 2 diabetes in a middle-aged Korean cohort: the Korean genome and epidemiology study. Circ J. 2012; 76(8): 1904–10

Lin C-C, Liu C-S, Lai M-M, Li C-I, Chen C-C, Chang P-C, Lin W-Y, Lee Y-D, Lin T, Li T-C. Metabolic syndrome in a Taiwanese metropolitan adult population. BMC Public Health. 2007; 239

Lin C-C, Liu C-S, Li T-C, Chen C-C, Li C-I, Lin W-Y. Microalbuminuria and the metabolic syndrome and its components in the Chinese population. Eur J Clin Invest. 2007; 37(10): 783-90

Lin L, Chen G, Zou X, Zhao J, Zhu F, Tu M, Xu S, Lin W, Yang S, Zhang Y, Lin M, Chen N, Huang H, Liang J, Li L, Yao J. Diabetes, pre-diabetes and associated risks on Minnesota code-indicated major electrocardiogram abnormality among Chinese: a cross-sectional diabetic study in Fujian province, southeast China. Obes Rev. 2009; 10(4): 420-30

Lin S, Cheng TO, Liu X, Mai J, Rao X, Gao X, Deng H, Shi M. Impact of dysglycemia, body mass index, and waist-to-hip ratio on the prevalence of systemic hypertension in a lean Chinese population. Am J Cardiol. 2006; 97(6): 839-42

Lind H, Nilsson P, Holthuis N, Lindholm L. Non-obese men with high lipoprotein(a) values-- a cardiovascular risk group different from those with the metabolic syndrome?. Scand J Clin Lab Invest. 1994; 54(2): 177-83

Lindblad U, Ek J, Eckner J, Larsson CA, Shan G, Rastam L. Prevalence, awareness, treatment, and control of hypertension: rule of thirds in the Skaraborg project. Scand J Prim Health Care. 2012; 30(2): 88–94

Lindroth M, Lundqvist R, Lilja M, Eliasson M. Cardiovascular risk factors differ between rural and urban Sweden: the 2009 Northern Sweden MONICA cohort. BMC Public Health. 2014; 14: 825

Lintott CJ, Hanger HC, Scott RS, Sainsbury R, Frampton C. Prevalence of diabetes mellitus in an ambulant elderly New Zealand population. Diabetes Res Clin Pract. 1992; 16(2): 131-6

Liu F, Ma Y-T, Yang Y-N, Zhen Y-J, Xie X, Li X-M, Ma X, Chen B-D, Huang Y, Shan C-F, Gao X-M. The prevalence of isolated systolic hypertension in adult populations from the Han, Uygur and Kazakh ethnic groups in Xinjiang, China. Blood Press. 2014; 23(3): 154–9

Liu L, Zhou C, Du H, Zhang K, Huang D, Wu J. The prevalences of impaired fasting glucose and diabetes mellitus in working age men of North China: Anshan Worker Health Survey. Sci Rep. 2014; 4835

Liu S, Wang W, Zhang J, He Y, Yao C, Zeng Z, Piao J, Howard BV, Fabsitz RR, Best L, Yang X, Lee ET. Prevalence of diabetes and impaired fasting glucose in Chinese adults, China National Nutrition and Health Survey, 2002. Prev Chronic Dis. 2011; 8(1): A13

Longo-Mbenza B, On'kin JBKL, Okwe AN, Kabangu NK, Fuele SM. Metabolic syndrome, aging, physical inactivity, and incidence of type 2 diabetes in general African population. Diab Vasc Dis Res. 2010; 7(1): 28-39

Lu B, Hu J, Wen J, Zhang Z, Zhou L, Li Y, Hu R. Determination of peripheral neuropathy prevalence and associated factors in Chinese subjects with diabetes and pre-diabetes - ShangHai Diabetic neuRopathy Epidemiology and Molecular Genetics Study (SH-DREAMS). PLoS One. 2013; 8(4): e61053

Lu FH, Yang YC, Wu JS, Wu CH, Chang CJ. A population-based study of the prevalence and associated factors of diabetes mellitus in southern Taiwan. Diabet Med. 1998; 15(7): 564-72

Lu K, Chen J, Wu S, Chen J, Hu D. Interaction of Sleep Duration and Sleep Quality on Hypertension Prevalence in Adult Chinese Males. J Epidemiol. 2015; 25(6): 415–22

Luksiene D, Tamosiunas A, Baceviciene M, Radisauskas R, Malinauskiene V, Peasey A, Bobak M. Trends in prevalence of dyslipidaemias and the risk of mortality in Lithuanian urban population aged 45-64 in relation to the presence of the dyslipidaemias and the other cardiovascular risk factors. PLoS One. 2014; 9(6): e100158

Lv Y-B, Yin Z-X, Chei C-L, Qian H-Z, Kraus VB, Zhang J, Brasher MS, Shi X-M, Matchar DB, Zeng Y. Low-density lipoprotein cholesterol was inversely associated with 3-year all-cause mortality among Chinese oldest old: data from the Chinese Longitudinal Healthy Longevity Survey. Atherosclerosis. 2015; 239(1): 137–42

Máchová L, Janout V, Cízek L, Beska F, Lorenc J, Koutná J. Risk factors for tumors, cardiovascular and metabolic diseases in the population of the Sumperk District. Cas Lek Cesk. 2004; 143(2): 90-3

Magliano DJ, Barr EL, Zimmet PZ, Cameron AJ, Dunstan DW, Colagiuri S, Jolley D, Owen N, Phillips P, Tapp RJ, Welborn TA, Shaw JE. Glucose indices, health behaviors, and incidence of diabetes in Australia: the Australian Diabetes, Obesity and Lifestyle Study. Diabetes Care. 2008; 31(2): 267-72

Mahanta TG, Joshi R, Mahanta BN, Xavier D. Prevalence of modifiable cardiovascular risk factors among tea garden and general population in Dibrugarh, Assam, India. J Epidemiol Glob Health. 2013; 3(3): 147–56

Makdisse M, Pereira A da C, Brasil D de P, Borges JL, Machado-Coelho GLL, Krieger JE, Nascimento Neto RM, Chagas ACP, Hearts of Brazil Study and Peripheral Arterial Disease Committee of the Brazilian Society of Cardiology/Funcor. Prevalence and risk factors associated with peripheral arterial disease in the Hearts of Brazil Project. Arq Bras Cardiol. 2008; 91(6): 370-82

Malavige GN, de Alwis NM, Weerasooriya N, Fernando DJ, Siribaddana SH. Increasing diabetes and vascular risk factors in a sub-urban Sri Lankan population. Diabetes Res Clin Pract. 2002; 57(2): 143-5

Malerbi DA, Franco LJ. Multicenter study of the prevalence of diabetes mellitus and impaired glucose tolerance in the urban Brazilian population aged 30-69 yr. The Brazilian Cooperative Group on the Study of Diabetes Prevalence. Diabetes Care. 1992; 15(11): 1509-16

Malhotra P, Kumari S, Kumar R, Jain S, Sharma BK. Prevalence and determinants of hypertension in an un-industrialised rural population of North India. J Hum Hypertens. 1999; 13(7): 467-72

Mancia G, Sega R, Milesi C, Cesana G, Zanchetti A. Blood-pressure control in the hypertensive population. Lancet. 1997; 349(9050): 454-7

Mannami T, Baba S, Ogata J. Potential of Carotid Enlargement as a Useful Indicator Affected by High Blood Pressure in a Large General Population of a Japanese City The Suita Study. Stroke. 2000; 31(12): 2958-65

Marshall SL, Edidin D, Arena VC, Becker DJ, Bunker CH, Gishoma C, Gishoma F, LaPorte RE, Kaberuka V, Ogle G, Sibomana L, Orchard TJ. Prevalence and incidence of clinically recognized cases of Type 1 diabetes in children and adolescents in Rwanda, Africa. Diabet Med. 2015; nan

Martinez-Hervas S, Carmena R, Ascaso JF, Real JT, Masana L, Catala M, Vendrell J, Vázquez JA, Valdes S, Urrutia I, Soriguer F, Serrano-Rios M, Rojo-Martínez G, Pascual-Manich G, Ortega E, Mora-Peces I, Menendez E, Martínez-Larrad MT, Lopez-Alba A, Gomis R, Goday A, Girbes J, Gaztambide S, Franch J, Delgado E, Castell C, Castano L, Casamitjana R, Calle-Pascual A, Bordiu E. Prevalence of plasma lipid abnormalities and its association with glucose metabolism in Spain: the diabetes study. Clin Investig Arterioscler. 2014; 26(3): 107–14

Masiá R, Sala J, Rohlfs I, Piulats R, Manresa JM, Marrugat J; Investigadores del estudio REGICOR. Prevalence of diabetes mellitus in the province of Girona, Spain: the REGICOR study. Rev Esp Cardiol. 2004; 57(3): 261-4

Mbanya JC, Ngogang J, Salah JN, Minkoulou E, Balkau B. Prevalence of NIDDM and impaired glucose tolerance in a rural and an urban population in Cameroon. Diabetologia. 1997; 40(7): 824-9

Mc Donald P AJ, Montenegro G JA, Cruz G CE, Moreno de Rivera AL, Cumbrera O A. Prevalence, sociodemographic distribution, treatment and control of diabetes mellitus in Panama. Diabetol Metab Syndr. 2013; 5(1): 69

McGarvey ST. Cardiovascular disease (CVD) risk factors in Samoa and American Samoa, 1990-95. Pac Health Dialog. 2001; 8(1): 157-62

Mèantyselkèa P, Miettola J, Niskanen L, Kumpusalo E. Glucose regulation and chronic pain at multiple sites. Rheumatology (Oxford). 2008; 47(8): 1235-8

Medina-Lezama J, Zea-Diaz H, Morey-Vargas OL, Bolaños-Salazar JF, Postigo-Macdowall M, Paredes-Díaz S, Corrales-Medina F, Valdivia-Ascuña Z, Cuba-Bustinza C, Villalobos-Tapia P, Muñoz-Atahualpa E, Chirinos-Pacheco J, Raij L, Chirinos JA. Prevalence and patterns of hypertension in Peruvian Andean Hispanics: the PREVENCION study. J Am Soc Hypertens. 2007; 1(3): 216-25

Mehta KD, Karki P, Lamsal M, Paudel IS, Majhi S, Das BKL, Sharma S, Jha N, Baral N. Hyperglycemia, glucose intolerance, hypertension and socioeconomic position in eastern Nepal. Southeast Asian J Trop Med Public Health. 2011; 42(1): 197–207

Melander O, Maisel AS, Almgren P, Manjer J, Belting M, Hedblad B, Engstrom G, Kilger U, Nilsson P, Bergmann A, Orho-Melander M. Plasma proneurotensin and incidence of diabetes, cardiovascular disease, breast cancer, and mortality. JAMA. 2012; 308(14): 1469–75

Mennen LI, Mbanya JC, Cade J, Balkau B, Sharma S, Chungong S, Cruickshank JK. The habitual diet in rural and urban Cameroon. Eur J Clin Nutr. 2000; 54(2): 150-4

Menotti A, Lanti M, Angeletti M, Panarelli W, Scavizzi P, Botta G, Cirillo M, Laurenzi M, Mancini M, Terradura-Vagnarelli O, Zanchetti A. Twenty-year cardiovascular and all-cause mortality trends and changes in cardiovascular risk factors in Gubbio, Italy: The role of blood pressure changes. J Hypertens. 2009; 27(2): 266-74

Metelskaya VA, Shkolnikova MA, Shalnova SA, Andreev EM, Deev AD, Jdanov DA, Shkolnikov VM, Vaupel JW. Prevalence, components, and correlates of metabolic syndrome (MetS) among elderly Muscovites. Arch Gerontol Geriatr. 2012; 55(2): 231–7

Mezuk B, Chen Y, Yu C, Guo Y, Bian Z, Collins R, Chen J, Pang Z, Wang H, Peto R, Que X, Zhang H, Tan Z, Kendler KS, Li L, Chen Z. Depression, anxiety, and prevalent diabetes in the Chinese population: findings from the China Kadoorie Biobank of 0.5 million people. J Psychosom Res. 2013; 75(6): 511–7

Midthjell K, Bjorndal A, Holmen J, Kr�ger O, Bjartveit K. Prevalence of known and previously unknown diabetes mellitus and impaired glucose tolerance in an adult Norwegian population. Indications of an increasing diabetes prevalence. The Nord-Tr?ndelag Diabetes Study. Scand J Prim Health Care. 1995; 13(3): 229-35

Miettola J, Nykanen I, Kumpusalo E. Health views and metabolic syndrome in a Finnish rural community: a cross-sectional population study. Can J Rural Med. 2012; 17(1): 10–6

Ministry of Health (Argentina), National Institute of Statistics and Censuses (Argentina). Argentina National Survey of Risk Factors 2009

Ministry of Health (Barbados). Barbados STEPS Noncommunicable Disease Risk Factors Survey 2007

Ministry of Health (Benin), World Health Organization (WHO). Benin - Littoral STEPS Noncommunicable Disease Risk Factors Survey 2007

Ministry of Health (Benin), World Health Organization (WHO). Benin STEPS Noncommunicable Disease Risk Factors Survey 2008

Ministry of Health (Bhutan), World Health Organization (WHO). Bhutan - Thimphu STEPS Noncommunicable Disease Risk Factors Survey 2007

Ministry of Health (Bhutan), World Health Organization (WHO). Bhutan STEPS Noncommunicable Disease Risk Factors Survey 2014

Ministry of Health (Cambodia), University of Health Sciences (Cambodia), World Health Organization (WHO). Cambodia STEPS Noncommunicable Disease Risk Factors Survey 2010

Ministry of Health (Indonesia), World Health Organization (WHO). Indonesia - Jawa Barat STEPS Noncommunicable Disease Risk Factors Survey 2006

Ministry of Health (Indonesia), World Health Organization (WHO). Indonesia STEPS Noncommunicable Disease Risk Factors Survey 2001

Ministry of Health (Italy), National Association of Hospital Cardiologists (Italy), National Center for Epidemiology, Surveillance, and Health Promotion (CNESPS) (Italy), National Institute of Health (Italy). Italy Health Examination Survey 2008-2012

Ministry of Health (Kuwait), World Health Organization (WHO). Kuwait STEPS Noncommunicable Disease Risk Factors Survey 2006

Ministry of Health (Kuwait), World Health Organization (WHO). Kuwait STEPS Noncommunicable Disease Risk Factors Survey 2014

Ministry of Health (Malawi), World Health Organization (WHO). Malawi STEPS Noncommunicable Disease Risk Factors Survey 2009

Ministry of Health (Malaysia), World Health Organization (WHO). Malaysia STEPS Noncommunicable Disease Risk Factors Survey 2005-2006

Ministry of Health (Mauritania), World Health Organization (WHO). Mauritania - Nouakchott STEPS Noncommunicable Disease Risk Factors Survey 2006

Ministry of Health (Moldova), National Bureau of Statistics (Moldova), National Center of Public Health (Moldova), World Health Organization (WHO). Moldova STEPS Noncommunicable Disease Risk Factors Survey 2013

Ministry of Health (Mozambique), World Health Organization (WHO). Mozambique STEPS Noncommunicable Disease Risk Factors Survey 2005

Ministry of Health (Myanmar), Myanmar Medical Association, World Health Organization (WHO). Mynamar STEPS Noncommunicable Disease Risk Factors Survey 2014

Ministry of Health (Myanmar), World Health Organization (WHO). Myanmar - Yangon STEPS Noncommunicable Disease Risk Factors Survey 2003

Ministry of Health (Niger), World Health Organization (WHO). Niger STEPS Noncommunicable Disease Risk Factors Survey 2007

Ministry of Health (Oman), World Health Organization (WHO). Oman - Ash Sharq?yah STEPS Noncommunicable Disease Risk Factors Survey 2006

Ministry of Health (Oman), World Health Organization (WHO). Oman World Health Survey 2007-2008

Ministry of Health (Oman). Oman National Health Survey 2000. Muscat, Oman: Ministry of Health (Oman)

Ministry of Health (Palestine), World Health Organization (WHO). Palestine STEPS Noncommunicable Disease Risk Factors Survey 2010-2011. Geneva, Switzerland: World Health Organization (WHO)

Ministry of Health (Saint Lucia), World Health Organization (WHO). Saint Lucia STEPS Noncommunicable Disease Risk Factors Survey 2012

Ministry of Health (Samoa). Samoa STEPS Noncommunicable Disease Risk Factors Survey 2002

Ministry of Health (Singapore). Singapore National Health Survey 1992

Ministry of Health (Singapore). Singapore National Health Survey 1998

Ministry of Health (Singapore). Singapore National Health Survey 2004

Ministry of Health (Swaziland), World Health Organization (WHO). Swaziland STEPS Noncommunicable Disease Risk Factors Survey 2007

Ministry of Health (Swaziland), World Health Organization (WHO). Swaziland STEPS Noncommunicable Disease Risk Factors Survey 2014

Ministry of Health (Timor-Leste), National University of East Timor, World Health Organization (WHO). Timor-Leste STEPS Noncommunicable Disease Risk Factors Survey 2014

Ministry of Health (Togo), West African Health Organization, World Health Organization (WHO). Togo STEPS Noncommunicable Disease Risk Factors Survey 2010-2011

Ministry of Health (Tonga), World Health Organization (WHO). Tonga STEPS Noncommunicable Disease Risk Factors Survey 2004

Ministry of Health (Turkey). Turkey Chronic Diseases and Risk Factors Study 2011

Ministry of Health (Uganda), World Health Organization (WHO). Uganda STEPS Noncommunicable Disease Risk Factors Survey 2014

Ministry of Health (Uzbekistan), World Bank, World Health Organization (WHO). Uzbekistan STEPS Noncommunicable Disease Risk Factors Survey 2014

Ministry of Health (Vanuatu), Secretariat of the Pacific Community (SPC). Vanuatu Noncommunicable Disease Survey 1998

Ministry of Health (Vanuatu), World Health Organization (WHO). Vanuatu STEPS Noncommunicable Disease Risk Factors Survey 2011

Ministry of Health (Zambia), World Health Organization (WHO). Zambia - Lusaka STEPS Noncommunicable Disease Risk Factors Survey 2008

Ministry of Health (Zanzibar), World Health Organization (WHO). Tanzania - Zanzibar STEPS Noncommunicable Disease Risk Factors Survey 2011

Ministry of Health and Child Welfare (Zimbabwe), University of Zimbabwe, World Health Organization (WHO). Zimbabwe STEPS Noncommunicable Disease Risk Factors Survey 2005

Ministry of Health and Medical Services (Kiribati), World Health Organization (WHO). Kiribati STEPS Noncommunicable Disease Risk Factors Survey 2004-2006

Ministry of Health and Population (Egypt), USAID, World Health Organization (WHO). Egypt STEPS Noncommunicable Disease Risk Factors Survey 2005

Ministry of Health and Population (Nepal), Nepal Health Research Council (NHRC), World Health Organization (WHO). Nepal STEPS Noncommunicable Disease Risk Factors Survey 2012-2013

Ministry of Health and Social Welfare (Lesotho), World Health Organization (WHO). Lesotho STEPS Noncommunicable Disease Risk Factors Survey 2012

Ministry of Health and Welfare (Japan), National Institute of Health and Nutrition (Japan). Japan National Nutrition Survey 1999

Ministry of Health and Welfare (Japan). Japan National Nutrition Survey 1991

Ministry of Health and Welfare (Japan). Japan National Nutrition Survey 1992

Ministry of Health and Welfare (Japan). Japan National Nutrition Survey 1993

Ministry of Health and Welfare (Japan). Japan National Nutrition Survey 1994

Ministry of Health and Welfare (Japan). Japan National Nutrition Survey 1995

Ministry of Health and Welfare (Japan). Japan National Nutrition Survey 1996

Ministry of Health and Welfare (Japan). Japan National Nutrition Survey 1997

Ministry of Health and Welfare (Japan). Japan National Nutrition Survey 1998

Ministry of Health, Labour and Welfare (Japan), National Institute of Health and Nutrition (Japan). Japan National Health and Nutrition Survey 2012

Ministry of Health, Labour and Welfare (Japan). Japan National Health and Nutrition Survey 2003

Ministry of Health, Labour and Welfare (Japan). Japan National Health and Nutrition Survey 2004

Ministry of Health, Labour and Welfare (Japan). Japan National Health and Nutrition Survey 2006

Ministry of Health, Labour and Welfare (Japan). Japan National Health and Nutrition Survey 2007

Ministry of Health, Labour and Welfare (Japan). Japan National Health and Nutrition Survey 2008

Ministry of Health, Labour and Welfare (Japan). Japan National Health and Nutrition Survey 2009

Ministry of Health, Labour and Welfare (Japan). Japan National Health and Nutrition Survey 2010

Ministry of Health, Labour and Welfare (Japan). Japan National Health and Nutrition Survey 2011

Ministry of Health, Population and Hospital Reform (Algeria), World Health Organization (WHO). Algeria - Sétif and Mostaganem STEPS Noncommunicable Disease Risk Factors Survey 2003

Ministry of Public Health (Chad), World Health Organization (WHO). Chad - Ville de N'Djaména STEPS Noncommunicable Disease Risk Factors Survey 2008

Ministry of Public Health (Congo, DR), World Health Organization (WHO). Democratic Republic of the Congo - Kinshasa STEPS Noncommunicable Disease Risk Factors Survey 2005

Ministry of Public Health (Ecuador), National Institute of Statistics and Censuses (Ecuador). Ecuador National Health and Nutrition Survey 2012

Ministry of Public Health (Thailand). Thailand National Health and Examination Survey 2003-2004

Ministry of Public Health (Thailand). Thailand National Health Examination Survey 1991-1992

Miquel JF, Covarrubias C, Villaroel L, Mingrone G, Greco AV, Puglielli L, Carvallo P, Marshall G, Del Pino G, Nervi F. Genetic epidemiology of cholesterol cholelithiasis among Chilean Hispanics, Amerindians, and Maoris. Gastroenterology. 1998; 115(4): 937-46

Mirmiran P, Hajifaraji M, Bahadoran Z, Sarvghadi F, Azizi F. Dietary protein intake is associated with favorable cardiometabolic risk factors in adults: Tehran Lipid and Glucose Study. Nutr Res. 2012; 32(3): 169–76

Modesti PA, Bamoshmoosh M, Rapi S, Massetti L, Al-Hidabi D, Al Goshae H. Epidemiology of hypertension in Yemen: effects of urbanization and geographical area. Hypertens Res. 2013; 36(8): 711–7

Modesti PA, Bamoshmoosh M, Rapi S, Massetti L, Bianchi S, Al-Hidabi D, Al Goshae H. Relationship between hypertension, diabetes and proteinuria in rural and urban households in Yemen. J Hum Hypertens. 2013; 27(9): 572-9

Mohan V, Deepa M, Anjana RM, Lanthorn H, Deepa R. Incidence of diabetes and pre-diabetes in a selected urban south Indian population (CUPS-19). J Assoc Physicians India. 2008; 152-7

Mohanna S, Baracco R, Seclén S. Lipid profile, waist circumference, and body mass index in a high altitude population. High Alt Med Biol. 2006; 7(3): 245-55

Möller CS, Zethelius B, Sundström J, Lind L. Impact of follow-up time and re-measurement of the electrocardiogram and conventional cardiovascular risk factors on their predictive value for myocardial infarction. J Intern Med. 2006; 260(1): 22-30

Moon H-W, Park CM, Hong SN, Park S, Hur M, Yun Y-M. Assessment of apoB dyslipoproteinemia in Korean population. Clin Biochem. 2013; 46(12): 1041–6

Morales DD, Punzalan FER, Paz-Pacheco E, Sy RG, Duante CA. Metabolic syndrome in the Philippine general population: prevalence and risk for atherosclerotic cardiovascular disease and diabetes mellitus. Diab Vasc Dis Res. 2008; 5(1): 36-43

Mostafa A, Mohamed MK, Saeed M, Hasan A, Fontanet A, Godsland I, Coady E, Esmat G, El-Hoseiny M, Abdul-Hamid M, Hughes A, Chaturvedi N. Hepatitis C infection and clearance: impact on atherosclerosis and cardiometabolic risk factors. Gut. 2010; 59(8): 1135-40

Motala AA, Esterhuizen T, Gouws E, Pirie FJ, Omar MA. Diabetes and other disorders of glycemia in a rural South African community: prevalence and associated risk factors. Diabetes Care. 2008; 31(9): 1783-8

Motta M, Bennati E, Capri M, Ferlito L, Malaguarnera M. Diabetes mellitus in the extreme longevity. Exp Gerontol. 2008; 43(2): 102-5

Muiesan ML, Padovani A, Salvetti M, Monteduro C, Poisa P, Bonzi B, Paini A, Cottini E, Agosti C, Castellano M, Rizzoni D, Vignolo A, Agabiti-Rosei E. Headache: Prevalence and relationship with office or ambulatory blood pressure in a general population sample (the Vobarno Study). Blood Press. 2006; 15(1): 14-9

Muiesan ML, Salvetti M, Paini A, Monteduro C, Rosei CA, Aggiusti C, Belotti E, Bertacchini F, Galbassini G, Stassaldi D, Castellano M, Rosei EA. Pulse wave velocity and cardiovascular risk stratification in a general population: The Vobarno study. J Hypertens. 2010; 28(9): 1935-43

Mukai N, Doi Y, Ninomiya T, Hata J, Hirakawa Y, Fukuhara M, Iwase M, Kiyohara Y. Cut-off values of fasting and post-load plasma glucose and HbA1c for predicting Type 2 diabetes in community-dwelling Japanese subjects: the Hisayama Study. Diabet Med. 2012; 29(1): 99–106

Mukai N, Doi Y, Ninomiya T, Hirakawa Y, Nagata M, Yoshida D, Hata J, Fukuhara M, Nakamura U, Kitazono T, Kiyohara Y. Trends in the prevalence of type 2 diabetes and prediabetes in community-dwelling Japanese subjects: The Hisayama Study. J Diabetes Investig. 2014; 5(2): 162-9

Muktabhant B, Sanchaisuriya P, Sarakarn P, Tawityanon W, Trakulwong M, Worawat S, Schelp FP. Use of glucometer and fasting blood glucose as screening tools for diabetes mellitus type 2 and glycated haemoglobin as clinical reference in rural community primary care settings of a middle income country. BMC Public Health. 2012; 12: 349

Muninarayana C, Balachandra G, Hiremath SG, Iyengar K, Anil NS. Prevalence and awareness regarding diabetes mellitus in rural Tamaka, Kolar. Int J Diabetes Dev Ctries. 2010; 30(1): 18-21

Muniz J, Hervada J, Juane R, Lopez-Rodriguez I, Castro-Beiras A. Prevalence of diabetes mellitus in the population aged 40-69 years in Galicia, northwest Spain. Diabetes Res Clin Pract. 1995; 30(2): 137-42

Muntoni S, Atzori L, Mereu R, Manca A, Satta G, Gentilini A, Bianco P, Baule A, Baule GM, Muntoni S. Prevalence of diagnosed and undiagnosed diabetes mellitus and impaired fasting glucose in Sardinia. Acta Diabetol. 2009; 46(3): 227–31

Murdock D, Salit J, Stoffel M, Friedman JM, Pe’er I, Breslow JL, Bonnen PE. Longitudinal study shows increasing obesity and hyperglycemia in micronesia. Obesity (Silver Spring). 2013; 21(9): E421–7

Murphy GA, Asiki G, Ekoru K, Nsubuga RN, Nakiyingi-Miiro J, Young EH, Seeley J, Sandhu MS, Kamali A. Sociodemographic distribution of non-communicable disease risk factors in rural Uganda: a cross-sectional study. Int J Epidemiol. 2013; 42(6): 1740–53

Muyer MT, Muls E, Mapatano MA, Makulo JR, Mvitu M, Kimenyembo W, Mandja BA, Kimbondo P, Bieleli CB, Kaimbo Wa Kaimbo D, Buntinx F. Diabetes and intermediate hyperglycaemia in Kisantu, DR Congo: a cross-sectional prevalence study. BMJ Open. 2012; 2(6): nan

Mykkänen L, Laakso M, Uusitupa M, Pyörälä K. Prevalence of diabetes and impaired glucose tolerance in elderly subjects and their association with obesity and family history of diabetes. Diabetes Care. 1990; 13(11): 1099-105

Nakagami T, Qiao Q, Carstensen B, Nhr-Hansen C, Hu G, Tuomilehto J, Balkau B, Borch-Johnsen K. Age, body mass index and Type 2 diabetes-associations modified by ethnicity. Diabetologia. 2003; 46(8): 1063-70

Nakagami T, Tominaga M, Nishimura R, Yoshiike N, Daimon M, Oizumi T, Tajima N. Is the measurement of glycated hemoglobin A1c alone an efficient screening test for undiagnosed diabetes? Japan National Diabetes Survey. Diabetes Res Clin Pract. 2007; 76(2): 251-6

Nangia V, Jonas JB, Sinha A, Matin A, Kulkarni M, Panda-Jonas S. Ocular axial length and its associations in an adult population of Central Rural India. The Central India Eye and Medical Study [Unpublished data]. Ophthalmology 2010;117(7):1360-6.

Napoli N, Mottini G, Arigliani M, Creta A, Giua R, Incammisa A, Carotti S, Sihom F, Yimagou I, Alombah R, Mbanya JC, Pozzilli P. Unexpectedly high rates of obesity and dysglycemia among villagers in Cameroon. Diabetes Metab Res Rev. 2010; 26(1): 10–12

NatCen Social Research and Royal Free and University College Medical School. Department of Epidemiology and Public Health, Health Survey for England, 2010 [computer file]. 2nd Edition. Colchester, Essex: UK Data Archive [distributor], July 2012. SN: 6986, http://dx.doi.org/10.5255/UKDA-SN-6986-2

NatCen Social Research and University College London. Department of Epidemiology and Public Health, Health Survey for England, 2011 [computer file]. Colchester, Essex: UK Data Archive [distributor], April 2013. SN: 7260, http://dx.doi.org/10.5255/UKDA-SN-7260-1

NatCen Social Research and University College London. Department of Epidemiology and Public Health, Health Survey for England, 2012 [computer file]. Colchester, Essex: UK Data Archive [distributor], April 2014. SN: 7480, http://dx.doi.org/10.5255/UKDA-SN-7480-1

NatCen Social Research and University College London. Department of Epidemiology and Public Health, Health Survey for England, 2013 [computer file]. Colchester, Essex: UK Data Archive [distributor], January 2015. SN: 7649, http://dx.doi.org/10.5255/UKDA-SN-7649-1

National Board of Health and Welfare (Sweden), Statistics Sweden. Sweden - Breast-feeding, Children Born 1998. Stockholm, Sweden: Statistics Sweden, 2000

National Center for Health Statistics (NCHS), Centers for Disease Control and Prevention (CDC). United States National Health and Nutrition Examination Survey 1988-1994. Hyattsville, United States: National Center for Health Statistics (NCHS), Centers for Disease Control and Prevention (CDC)

National Center for Health Statistics (NCHS), Centers for Disease Control and Prevention (CDC). United States National Health and Nutrition Examination Survey 1999-2000. Hyattsville, United States: National Center for Health Statistics (NCHS), Centers for Disease Control and Prevention (CDC)

National Center for Health Statistics (NCHS), Centers for Disease Control and Prevention (CDC). United States National Health and Nutrition Examination Survey 2001-2002. Hyattsville, United States: National Center for Health Statistics (NCHS), Centers for Disease Control and Prevention (CDC)

National Center for Health Statistics (NCHS), Centers for Disease Control and Prevention (CDC). United States National Health and Nutrition Examination Survey 2003-2004. Hyattsville, United States: National Center for Health Statistics (NCHS), Centers for Disease Control and Prevention (CDC)

National Center for Health Statistics (NCHS), Centers for Disease Control and Prevention (CDC). United States National Health and Nutrition Examination Survey 2005-2006. Hyattsville, United States: National Center for Health Statistics (NCHS), Centers for Disease Control and Prevention (CDC), 2007

National Center for Health Statistics (NCHS), Centers for Disease Control and Prevention (CDC). United States National Health and Nutrition Examination Survey 2007-2008. Hyattsville, United States: National Center for Health Statistics (NCHS), Centers for Disease Control and Prevention (CDC), 2009

National Center for Health Statistics (NCHS), Centers for Disease Control and Prevention (CDC). United States National Health and Nutrition Examination Survey 2009-2010. Hyattsville, United States: National Center for Health Statistics (NCHS), Centers for Disease Control and Prevention (CDC), 2011

National Center for Health Statistics (NCHS), Centers for Disease Control and Prevention (CDC). United States National Health and Nutrition Examination Survey 2011-2012. Hyattsville, United States: National Center for Health Statistics (NCHS), Centers for Disease Control and Prevention (CDC), 2013

National Center for Health Statistics (NCHS), Centers for Disease Control and Prevention (CDC). United States National Health and Nutrition Examination Survey 2013-2014. Hyattsville, United States: National Center for Health Statistics (NCHS), Centers for Disease Control and Prevention (CDC)

National Centre for Social Research and University College London. Department of Epidemiology and Public Health, Health Survey for England, 2003 [computer file]. Colchester, Essex: UK Data Archive [distributor], March 2005. SN: 5098

National Centre for Social Research and University College London. Department of Epidemiology and Public Health, Health Survey for England, 2004 [computer file]. Colchester, Essex: UK Data Archive [distributor], July 2006. SN: 5439

National Centre for Social Research and University College London. Department of Epidemiology and Public Health, Health Survey for England, 2006 [computer file]. 4th Edition. Colchester, Essex: UK Data Archive [distributor], July 2011. SN: 5809, http://dx.doi.org/10.5255/UKDA-SN-5809-1

National Centre for Social Research and University College London. Department of Epidemiology and Public Health, Health Survey for England, 1999 [computer file]. 3rd Edition. Colchester, Essex: UK Data Archive [distributor], February 2002. SN: 4365

National Centre for Social Research, University College London Department of Epidemiology and Public Health, Health Survey for England, 1998 [computer file]. 4th ed. Colchester, Essex: UK Data Archive [distributor], 30 November 2002. SN: 4150

National Health Examination Survey Office (Thailand). Thailand National Health and Examination Survey 2008-2009

National Institute for Medical Research (Tanzania), World Health Organization (WHO). Tanzania STEPS Noncommunicable Disease Risk Factors Survey 2012

National Institute for Public Health and the Environment (Netherlands), Statistics Netherlands. Netherlands Risk Factors and Health Survey 1998-2001

National Institute for Public Health and the Environment (Netherlands), Statistics Netherlands. Netherlands Risk Factors and Health Survey 1999

National Institute for Public Health and the Environment (Netherlands), Statistics Netherlands. Netherlands Risk Factors and Health Survey 2000

National Institute of Public Health (Czech Republic). Czech Republic HELEN Study - Health, Lifestyle and Environment Phase II 2004-2005

National Institute of Public Health (Mexico). Mexico National Health Survey 1999-2000

National Institute of Public Health (Mexico). Mexico National Survey of Health and Nutrition 2005-2006. Cuernavaca, Mexico: National Institute of Public Health (Mexico)

National Institute of Public Health (Mexico). Mexico National Survey of Health and Nutrition 2011-2012. Cuernavaca, Mexico: National Institute of Public Health (Mexico)

National Institute of Public Health, Ministry of Health, Population, and Hospital Reform (Algeria). Algeria National Health Survey 2005

National Institute of Statistics and Censuses (Argentina). Argentina National Survey of Risk Factors 2013. Buenos Aires, Argentina: National Institute of Statistics and Censuses (Argentina)

National University of Singapore. National University of Singapore Heart Study 1993-1995

Nazir A, Papita R, Anbalagan VP, Anjana RM, Deepa M, Mohan V. Prevalence of diabetes in Asian Indians based on glycated hemoglobin and fasting and 2-H post-load (75-g) plasma glucose (CURES-120). Diabetes Technol Ther. 2012; 14(8): 665–8

Nilsson PM, Møller L, Solstad K. Adverse effects of psychosocial stress on gonadal function and insulin levels in middle-aged males. J Intern Med. 1995; 237(5): 479-86

Njelekela MA, Mpembeni R, Muhihi A, Mligiliche NL, Spiegelman D, Hertzmark E, Liu E, Finkelstein JL, Fawzi WW, Willett WC, Mtabaji J. Gender-related differences in the prevalence of cardiovascular disease risk factors and their correlates in urban Tanzania. BMC Cardiovasc Disord. 2009; 9: 30

Noto D, Barbagallo CM, Cefalu' AB, Cavera G, Sapienza M, Notarbartolo A, Davi' G, Averna MR. Factor VII activity is an independent predictor of cardiovascular mortality in elderly women of a Sicilian population: results of an 11-year follow-up. Thromb Haemost. 2002; 87(2): 206-10

Nyenwe EA, Odia OJ, Ihekwaba AE, Ojule A, Babatunde S. Type 2 diabetes in adult Nigerians: a study of its prevalence and risk factors in Port Harcourt, Nigeria. Diabetes Res Clin Pract. 2003; 62(3): 177-85

O Connor JM, Millar SR, Buckley CM, Kearney PM, Perry IJ. The prevalence and determinants of undiagnosed and diagnosed type 2 diabetes in middle-aged irish adults. PLoS One. 2013; 8(11): e80504

Ohkura T, Taniguchi S-I, Osaki Y, Yamamoto N, Sumi K, Fujioka Y, Matsuzawa K, Izawa S, Shiochi H, Kinoshita H, Inoue K, Takechi M, Kishimoto T, Shigemasa C. Lower fasting plasma glucose criteria and high triglycerides are effective for screening diabetes mellitus in the rural Japanese population: the Tottori-Kofu Study. Rural Remote Health. 2011; 11(3): 1697

Ohmura T, Ueda K, Kiyohara Y, Kato I, Iwamoto H, Nakayama K, Nomiyama K, Ohmori S, Yoshitake T, Shinkawu A. Prevalence of type 2 (non-insulin-dependent) diabetes mellitus and impaired glucose tolerance in the Japanese general population: the Hisayama Study. Diabetologia. 1993; 36(11): 1198-203

Ohsawa M, Itai K, Tanno K, Onoda T, Ogawa A, Nakamura M, Kuribayashi T, Yoshida Y, Kawamura K, Sasaki S, Sakata K, Okayama A. Cardiovascular risk factors in the Japanese northeastern rural population. Int J Cardiol. 2009; 137(3): 226–35

Okada K, Furusyo N, Sawayama Y, Kanamoto Y, Murata M, Hayashi J. Prevalence and risk factors for diabetes: a ten year follow-up study of the Yaeyama district of Okinawa. Hukuoka Acta Med. 2010; 101(10): 215-24

Okesina AB, Oparinde DP, Akindoyin KA, Erasmus RT. Prevalence of some risk factors of coronary heart disease in a rural Nigerian population. East Afr Med J. 1999; 76(4): 212-6

Oladapo OO, Salako L, Sodiq O, Shoyinka K, Adedapo K, Falase AO. A prevalence of cardiometabolic risk factors among a rural Yoruba south-western Nigerian population: a population-based survey. Cardiovasc J Afr. 2010; 21(1): 26–31

Omboni S, Carabelli G, Ghirardi E, Carugo S. Awareness, treatment, and control of major cardiovascular risk factors in a small-scale Italian community: results of a screening campaign. Vasc Health Risk Manag. 2013; 9: 177–85

Onat A, Hergenc G, Bulur S, Ugur M, Kucukdurmaz Z, Can G. The paradox of high apolipoprotein A-I levels independently predicting incident type-2 diabetes among Turks. Int J Cardiol. 2010; 142(1): 72–9

Ono K, Limbu YR, Rai SK, Kurokawa M, Yanagida J, Rai G, Gurung N, Sharma M, Rai CK. The prevalence of type 2 diabetes mellitus and impaired fasting glucose in semi-urban population of Nepal. Nepal Med Coll J. 2007; 9(3): 154-6

Ostovaneh MR, Zamani F, Sharafkhah M, Ansari-Moghaddam A, Akhavan Khaleghi N, Saeedian FS, Rohani Z, Motamed N, Maadi M, Malekzadeh R, Poustchi H. Prevalence of metabolic syndrome in Amol and Zahedan, Iran: a population based study. Arch Iran Med. 2014; 17(7): 477–82

Ostrauskas R, Zalinkevicius R, Jurgeviciene N, Radzeviciene L, Lasaite L. The incidence of type 1 diabetes mellitus among 15-34 years aged Lithuanian population: 18-year incidence study based on prospective databases. BMC Public Health. 2011; 813

Ostrauskas R. The prevalence of type 1 diabetes mellitus among adolescents and adults in Lithuania during 1991-2004. Medicina (Kaunas). 2007; 43(3): 242-50

Oti SO, van de Vijver SJM, Agyemang C, Kyobutungi C. The magnitude of diabetes and its association with obesity in the slums of Nairobi, Kenya: results from a cross-sectional survey. Trop Med Int Health. 2013; 18(12): 1520-30

Palacios C, Pérez CM, Guzmán M, Ortiz AP, Ayala A, Suárez E. Association between adiposity indices and cardiometabolic risk factors among adults living in Puerto Rico. Public Health Nutr. 2011; 14(10): 1714–23

Palomo L, Felix-Redondo F-J, Lozano-Mera L, Perez-Castan J-F, Fernandez-Berges D, Buitrago F. Cardiovascular risk factors, lifestyle, and social determinants: a cross-sectional population study. Br J Gen Pract. 2014; 64(627): e627–33

Pan American Health Organization (PAHO), Center for Demography and Ecology, University of Wisconsin-Madison, Inter-University Consortium for Political and Social Research (ICPSR), College of the Northern Border (COLEF), Research in Health and Demographics (INSAD), National Institute of Medical Sciences and Nutrition Salvador Zubirán. Mexico - Mexico City Survey on Health, Well-Being, and Aging in Latin America and the Caribbean 1999-2000. Ann Arbor, United States: Inter-University Consortium for Political and Social Research (ICPSR)

Pan American Health Organization (PAHO). Central America Diabetes Initiative (CAMDI): Survey of Diabetes, Hypertension and Chronic Disease Risk Factors. Belize, San José, San Salvador, Guatemala City, Managua and Tegucigalpa. Washington, D.C., United States: Pan American Health Organization (PAHO), 2011

Pan American Health Organization (PAHO). Honduras - Tegucigalpa Diabetes, Hypertension, and Non-Communicable Disease Risk Factors Survey 2003-2004

Pan W-H, Wu H-J, Yeh C-J, Chuang S-Y, Chang H-Y, Yeh N-H, Hsieh Y-T. Diet and health trends in Taiwan: comparison of two nutrition and health surveys from 1993-1996 and 2005-2008. Asia Pac J Clin Nutr. 2011; 20(2): 238–50

Pan X, Yang W, Liu J. Prevalence of diabetes and its risk factors in China 1994. National Diabetes Prevention and Control Cooperative Group. Chin J Intern Med. 1997; 36(6): 384-9

Papantoniou K, Fito M, Covas M-I, Munoz D, Schroder H. trans Fatty acid consumption, lifestyle and type 2 diabetes prevalence in a Spanish population. Eur J Nutr. 2010; 49(6): 357–64

Papazoglou N, Manes C, Chatzimitrofanous P, Papadeli E, Tzounas K, Scaragas G, Kontogiannis I, Alexiades D. Epidemiology of diabetes mellitus in the elderly in northern Greece: a population study. Diabet Med. 1995; 12(5): 397-400

Park Y, Lee H, Koh CS, Min H. Community-based epidemiologic study on atherosclerotic cardiovascular risk factors. Diabetes Res Clin Pract. 1996; S65-72

Pasalic D, Dodig S, Corovic N, Pizent A, Jurasovic J, Pavlovic M. High prevalence of metabolic syndrome in an elderly Croatian population - a multicentre study. Public Health Nutr. 2011; 14(9): 1650–7

Patandin S, Bots ML, Abel R, Valkenburg HA. Impaired glucose tolerance and diabetes mellitus in a rural population in south India. Diabetes Res Clin Pract. 1994; 24(1): 47-53

Pedersen ML. Diabetes mellitus in Greenland. Dan Med J. 2012; 59(2): B4386

Pemminati S, Prabha Adhikari MR, Pathak R, Pai MRSM. Prevalence of metabolic syndrome (METS) using IDF 2005 guidelines in a semi urban south Indian (Boloor Diabetes Study) population of Mangalore. J Assoc Physicians India. 2010; 58: 674–7

Pérez CM, Guzmán M, Ortiz AP, Estrella M, Valle Y, Pérez N, Haddock L, Suárez E. Prevalence of the metabolic syndrome in San Juan, Puerto Rico. Ethn Dis. 2008; 18(4): 434-41

Pessinaba S, Mbaye A, Yabeta G-A-D, Kane A, Ndao CT, Ndiaye MB, Harouna H, Bodian M, Diao M, Mbaye MN, Diagne MN, Diack B, Kane M, Niang K, Mathieu J-BS, Kane A. Prevalence and determinants of hypertension and associated cardiovascular risk factors: data from a population-based, cross-sectional survey in Saint Louis, Senegal. Cardiovasc J Afr. 2013; 24(5): 180–3

Pinidiyapathirage MJ, Kasturiratne A, Ranawaka UK, Gunasekara D, Wijekoon N, Medagoda K, Perera S, Takeuchi F, Kato N, Warnakulasuriya T, Wickremasinghe AR. The burden of diabetes mellitus and impaired fasting glucose in an urban population of Sri Lanka. Diabet Med. 2013; 30(3): 326-32

Polakowska M, Piotrowski W. Incidence of diabetes in the Polish population: results of the Multicenter Polish Population Health Status Study--WOBASZ. Pol Arch Med Wewn. 2011; 121(5): 156-63

Pongchaiyakul C, Kotruchin P, Wanothayaroj E, Nguyen TV. An innovative prognostic model for predicting diabetes risk in the Thai population. Diabetes Res Clin Pract. 2011; 94(2): 193–8

Pramono LA, Setiati S, Soewondo P, Subekti I, Adisasmita A, Kodim N, Sutrisna B. Prevalence and predictors of undiagnosed diabetes mellitus in Indonesia. Acta Med Indones. 2010; 42(4): 216-23

Prasad DS, Kabir Z, Dash AK, Das BC. Prevalence and risk factors for diabetes and impaired glucose tolerance in Asian Indians: a community survey from urban eastern India. Diabetes Metab Syndr. 2012; 6(2): 96-101

Public Health Foundation of India. India - Jaipur Heart Watch Study Data on Blood Pressure, Cholesterol, BMI, and Fasting Blood Glucose 1993-2001

Pubudu De Silva A, Padmal De Silva SH, Liyanage IK, Rajapakse LC, Jayasinghe KSA, Katulanda P, Wijeratne CN, Wijeratne S. Social, cultural and economical determinants of diabetes mellitus in Kalutara district, Sri Lanka: a cross sectional descriptive study. Int J Equity Health. 2012; 11: 76

Qatar Statistics Authority, Supreme Council of Health (Qatar), World Health Organization (WHO). Qatar STEPS Noncommunicable Disease Risk Factors Survey 2012

Qi L, Feng L, Ding X, Mao D, Wang Y, Xiong H. Prevalence of diabetes and impaired fasting glucose among residents in the Three Gorges Reservoir Region, China. BMC Public Health. 2014; 14: 1152

Qian Q, Li X, Huang X, Fu M, Meng Z, Chen M, Feng B. Glucose metabolism among residents in Shanghai: natural outcome of a 5-year follow-up study. J Endocrinol Invest. 2012; 35(5): 453–8

Qin X, Li J, Zhang Y, Ma W, Fan F, Wang B, Xing H, Tang G, Wang X, Xu X, Xu X, Huo Y. Prevalence and associated factors of diabetes and impaired fasting glucose in Chinese hypertensive adults aged 45 to 75 years. PLoS One. 2012; 7(8): e42538

Quan HL, Blizzard CL, Venn AJ, Thuy AB, Luc PH, Sharman JE. Blood pressure and body mass index: a comparison of the associations in the Caucasian and Asian populations. Hypertens Res. 2012; 35(5): 523-30

Quoc PS, Charles MA, Cuong NH, Lieu LH, Tuan NA, Thomas M, Balkau B, Simon D. Blood glucose distribution and prevalence of diabetes in Hanoi (Vietnam). Am J Epidemiol. 1994; 139(7): 713-22

Radhakrishnan S, Balamurugan S. Prevalence of diabetes and hypertension among geriatric population in a rural community of Tamilnadu. Indian J Med Sci. 2013; 67(5-6): 130-8

Rahman MM, Rahim MA, Nahar Q. Prevalence and risk factors of type 2 diabetes in an urbanizing rural community of Bangladesh. Bangladesh Med Res Counc Bull. 2007; 33(2): 48-54

Rahman MS, Akter S, Abe SK, Islam MR, Mondal MN, Rahman JA, Rahman MM. Awareness, treatment, and control of diabetes in Bangladesh: a nationwide population-based study. PLoS One. 2015; 10(2): e0118365

Raiko JRH, Viikari JSA, Ilmanen A, Hutri-Kahonen N, Taittonen L, Jokinen E, Pietikainen M, Jula A, Loo B-M, Marniemi J, Lehtimaki T, Kahonen M, Ronnemaa T, Raitakari OT, Juonala M. Follow-ups of the Cardiovascular Risk in Young Finns Study in 2001 and 2007: levels and 6-year changes in risk factors. J Intern Med. 2010; 267(4): 370–84

Rajala U, Keinanen-Kiukaanniemi S, Uusimaki A, Reijula K, Kivela SL. Prevalence of diabetes mellitus and impaired glucose tolerance in a middle-aged Finnish population. Scand J Prim Health Care. 1995; 13(3): 222-8

Rajput R, Rajput M, Singh J, Bairwa M. Prevalence of diabetes mellitus among the adult population in rural blocks of Haryana, India: a community-based study. Metab Syndr Relat Disord. 2012; 10(6): 443-6

Ramachandran A, Snehalatha C, Kapur A, Vijay V, Mohan V, Das AK, Rao PV, Yajnik CS, Prasanna Kumar KM, Nair JD. High prevalence of diabetes and impaired glucose tolerance in India: National Urban Diabetes Survey. Diabetologia. 2001; 44(9): 1094-101

Raman Kutty V, Joseph A, Soman CR. High prevalence of type 2 diabetes in an urban settlement in Kerala, India. Ethn Health. 1999; 4(4): 231-9

Ramke J, Lee L, Brian G. Prevalence of diabetes among adults aged ?40 years in Timor-Leste. J Diabetes. 2012; 4(4): 392-4

Rampal S, Mahadeva S, Guallar E, Bulgiba A, Mohamed R, Rahmat R, Arif MT, Rampal L. Ethnic differences in the prevalence of metabolic syndrome: results from a multi-ethnic population-based survey in Malaysia. PLoS One. 2012; 7(9): e46365

Rampal S, Rampal L, Rahmat R, Zain AM, Yap YG, Mohamed M, Taha M. Variation in the prevalence, awareness, and control of diabetes in a multiethnic population: a nationwide population study in Malaysia. Asia Pac J Public Health. 2010; 22(2): 194-202

Rao CR, Kamath VG, Shetty A, Kamath A. A study on the prevalence of type 2 diabetes in coastal Karnataka. Int J Diabetes Dev Ctries. 2010; 30(2): 80-5

Rathmann W, Haastert B, Icks A, Löwel H, Meisinger C, Holle R, Giani G. High prevalence of undiagnosed diabetes mellitus in Southern Germany: target populations for efficient screening. The KORA survey 2000. Diabetologia. 2003; 46(2): 182-9

Rathmann W, Kowall B, Tamayo T, Giani G, Holle R, Thorand B, Heier M, Huth C, Meisinger C. Hemoglobin A1c and glucose criteria identify different subjects as having type 2 diabetes in middle-aged and older populations: the KORA S4/F4 Study. Ann Med. 2012; 44(2): 170–7

Rathmann W, Strassburger K, Heier M, Holle R, Thorand B, Giani G, Meisinger C. Incidence of Type 2 diabetes in the elderly German population and the effect of clinical and lifestyle risk factors: KORA S4/F4 cohort study. Diabet Med. 2009; 26(12): 1212-9

Ravikumar P, Bhansali A, Ravikiran M, Bhansali S, Walia R, Shanmugasundar G, Thakur JS, Kumar Bhadada S, Dutta P. Prevalence and risk factors of diabetes in a community-based study in North India: the Chandigarh Urban Diabetes Study (CUDS). Diabetes Metab. 2011; 37(3): 216-21

Rey A, Thoenes M, Fimmers R, Meier CA, Bramlage P. Diabetes prevalence and metabolic risk profile in an unselected population visiting pharmacies in Switzerland. Vasc Health Risk Manag. 2012; 541-7

Rigo Carratala F, Frontera Juan G, Canaves Llobera J, Rodriguez Ruiz T, Borras Bosch T, Fuentespina Vidal E, . Prevalencia de factores de riesgo cardiovascular en IslasBaleares (estudio CORSAIB). Rev Esp Cardiol. 2005; 58(12): 1411-9

Rigo JC, Vieira JL, Dalacorte RR, Reichert CL. Prevalence of metabolic syndrome in an elderly community: comparison between three diagnostic methods. Arq Bras Cardiol. 2009; 93(2): 85-91

Ritchie GE, Kengne AP, Joshi R, Chow C, Neal B, Patel A, Zoungas S. Comparison of near-patient capillary glucose measurement and a risk assessment questionnaire in screening for type 2 diabetes in a high-risk population in rural India. Diabetes Care. 2011; 34(1): 44–9

Rönnemaa E, Zethelius B, Lannfelt L, Kilander L. Vascular risk factors and dementia: 40-year follow-up of a population-based cohort. Dement Geriatr Cogn Disord. 2011; 31(6): 460–6

Rosado Martín J, Martínez López MÁ, Mantilla Morató T, Dujovne Kohan I, Palau Cuevas FJ, Torres Jiménez R, García Puig J. [Prevalence of diabetes in an adult population in the region of Madrid (Spain). The Madrid Cardiovascular Risk study]. Gac Sanit. 2012; 26(3): 243-50

Rosero-Bixby, Luis , Xinia Fernández, and William H. Dow. CRELES: Costa Rican Longevity and Healthy Aging Study, 2005 (Costa Rica Estudio de Longevidad y Envejecimiento Saludable) [Computer file]. ICPSR26681-v1. Ann Arbor, MI: Inter-university Consortium for Political and Social Research [distributor], 2010-07-21. doi:10.3886/ICPSR26681

Rosmond R, Björntorp P. Blood pressure in relation to obesity, insulin and the hypothalamic-pituitary-adrenal axis in Swedish men. J Hypertens. 1998; 16(12): 1721-6

Rosso D, Campagna S, Di Stefano F, Romano G, Maugeri D, Maggi S, Motta M, Catanzaro S, Carnazzo G. Prevalence of diabetes mellitus in a sample of the elderly population of the city of Catania. Arch Gerontol Geriatr. 1998; 27(3): 223-35

Rouvre M, Vol S, Gusto G, Born C, Lantieri O, Tichet J, Lecomte P. Low high density lipoprotein cholesterol: prevalence and associated risk-factors in a large French population. Ann Epidemiol. 2011; 21(2): 118–27

Rubio-Martin E, Soriguer F, Gutierrez-Repiso C, Garrido-Sanchez L, de Adana MSR, Garcia-Fuentes E, Morcillo S, Esteva I, Chaves FJ, Rojo-Martinez G. C-reactive protein and incidence of type 2 diabetes in the Pizarra study. Eur J Clin Invest. 2013; 43(2): 159–67

Rutkowski M, Bandosz P, Czupryniak L, Gaciong Z, Solnica B, Jasiel-Wojculewicz H, Wyrzykowski B, Pencina MJ, Zdrojewski T. Prevalence of diabetes and impaired fasting glucose in Poland--the NATPOL 2011 Study. Diabet Med. 2014; 31(12): 1568–71

Saadi H, Carruthers SG, Nagelkerke N, Al-Maskari F, Afandi B, Reed R, Lukic M, Nicholls MG, Kazam E, Algawi K, Al-Kaabi J, Leduc C, Sabri S, El-Sadig M, Elkhumaidi S, Agarwal M, Benedict S. Prevalence of diabetes mellitus and its complications in a population-based sample in Al Ain, United Arab Emirates. Diabetes Res Clin Pract. 2007; 78(3): 369-77

Sabir A, Ohwovoriole A, Isezuo S, Fasanmade O, Abubakar S, Iwuala S. Type 2 diabetes mellitus and its risk factors among the rural Fulanis of Northern Nigeria. Ann Afr Med. 2013; 12(4): 217-22

Sadeghi M, Roohafza H, Shirani S, Poormoghadas M, Kelishadi R, Baghaii A, Sarraf-Zadegan N. Diabetes and associated cardiovascular risk factors in Iran: the Isfahan Healthy Heart Programme. Ann Acad Med Singapore. 2007; 36(3): 175-80

Sadikot SM, Nigam A, Das S, Bajaj S, Zargar AH, Prasannakumar KM, Sosale A, Munichoodappa C, Seshiah V, Singh SK, Jamal A, Sai K, Sadasivrao Y, Murthy SS, Hazra DK, Jain S, Mukherjee S, Bandyopadhay S, Sinha NK, Mishra R, Dora M, Jena B, Patra P, Goenka K. The burden of diabetes and impaired glucose tolerance in India using the WHO 1999 criteria: prevalence of diabetes in India study (PODIS). Diabetes Res Clin Pract. 2004; 66(3): 301-7

Safari M, Yazdanpanah B, Yazdanpanah B, Mobasheri A. A population-based screening of type 2 diabetes in high-risk population of Yasuj, Iran. J Health Popul Nutr. 2014; 32(4): 677–86

Salazar MR, Carbajal HA, Espeche WG, Aizpurua M, Leiva Sisnieguez CE, March CE, Balbin E, Stavile RN, Reaven GM. Identifying cardiovascular disease risk and outcome: use of the plasma triglyceride/high-density lipoprotein cholesterol concentration ratio versus metabolic syndrome criteria. J Intern Med. 2013; 273(6): 595–601

Samuel P, Antonisamy B, Raghupathy P, Richard J, Fall CHD. Socio-economic status and cardiovascular risk factors in rural and urban areas of Vellore, Tamilnadu, South India. Int J Epidemiol. 2012; 41(5): 1315-27

Sanisoglu SY, Oktenli C, Hasimi A, Yokusoglu M, Ugurlu M. Prevalence of metabolic syndrome-related disorders in a large adult population in Turkey. BMC Public Health. 2006; 92

Santander Center for Public Health (Colombia), Santander Ministry of Health (Colombia), World Health Organization (WHO). Colombia - Santander STEPS Noncommunicable Disease Risk Factors Survey 2010

Saquib N, Khanam MA, Saquib J, Anand S, Chertow GM, Barry M, Ahmed T, Cullen MR. High prevalence of type 2 diabetes among the urban middle class in Bangladesh. BMC Public Health. 2013; 1032

Sasaki H, Kawasaki T, Ogaki T, Kobayashi S, Itoh K, Yoshimizu Y, Sharma S, Acharya GP. The prevalence of diabetes mellitus and impaired fasting glucose/glycaemia (IFG) in suburban and rural Nepal-the communities--based cross-sectional study during the democratic movements in 1990. Diabetes Res Clin Pract. 2005; 67(2): 167-74

Satoh H, Fujii S, Furumoto T, Kishi R, Tsutsui H. Waist circumference can predict the occurrence of multiple metabolic risk factors in middle-aged Japanese subjects. Ind Health. 2010; 48(4): 447–51

Satoh H, Kishi R, Tsutsui H. Metabolic syndrome is a significant and independent risk factor for increased arterial stiffness in Japanese subjects. Hypertens Res. 2009; 32(12): 1067–71

Sayeed MA, Hussain MZ, Banu A, Rumi MA, Azad Khan AK. Prevalence of diabetes in a suburban population of Bangladesh. Diabetes Res Clin Pract. 1997; 34(3): 149-55

Sayeed MA, Mahtab H, Akter Khanam P, Abdul Latif Z, Keramat Ali SM, Banu A, Ahren B, Azad Khan AK. Diabetes and impaired fasting glycemia in a rural population of Bangladesh. Diabetes Care. 2003; 26(4): 1034-9

Sayeed MA, Mahtab H, Khanam PA, Latif ZA, Banu A, Khan AK. Prevalence of diabetes and impaired fasting glucose in urban population of Bangladesh. Bangladesh Med Res Counc Bull. 2007; 33(1): 1-12

Schaan BD, Harzheim E, Gus I. [Cardiac risk profile in diabetes mellitus and impaired fasting glucose]. Rev Saude Publica. 2004; 38(4): 529-36

Scheltens T, Bots ML, Numans ME, Grobbee DE, Hoes AW. Awareness, treatment and control of hypertension: the "rule of halves" in an era of risk-based treatment of hypertension. J Hum Hypertens. 2007; 21(2): 99-106

Schmid R, Vollenweider P, Waeber G, Marques-Vidal P. Estimating the risk of developing type 2 diabetes: a comparison of several risk scores: the Cohorte Lausannoise study. Diabetes Care. 2011; 34(8): 1863–8

Schottker B, Herder C, Rothenbacher D, Perna L, Muller H, Brenner H. Serum 25-hydroxyvitamin D levels and incident diabetes mellitus type 2: a competing risk analysis in a large population-based cohort of older adults. Eur J Epidemiol. 2013; 28(3): 267–75

Schöttker B, Raum E, Rothenbacher D, Müller H, Brenner H. Prognostic value of haemoglobin A1c and fasting plasma glucose for incident diabetes and implications for screening. Eur J Epidemiol. 2011; 26(10): 779–87

Scragg R, Baker J, Metcalf P, Dryson E. Prevalence of diabetes mellitus and impaired glucose tolerance in a New Zealand multiracial workforce. N Z Med J. 1991; 104(920): 395-7

Scuteri A, Najjar SS, Orru' M, Albai G, Strait J, Tarasov KV, Piras MG, Cao A, Schlessinger D, Uda M, Lakatta EG. Age- and gender-specific awareness, treatment, and control of cardiovascular risk factors and subclinical vascular lesions in a founder population: the SardiNIA Study. Nutr Metab Cardiovasc Dis. 2009; 19(8): 532–41

Seclen SN, Rosa ME, Arias AJ, Huayta E, Medina CA. Prevalence of diabetes and impaired fasting glucose in Peru: report from PERUDIAB, a national urban population-based longitudinal study. BMJ Open Diabetes Res Care. 2015; 3(e000110)

Secretariat of Health and Environment (Libya), World Health Organization (WHO). Libya STEPS Noncommunicable Disease Risk Factors Survey 2009

Sekikawa A, Tominaga M, Takahashi K, Eguchi H, Igarashi M, Ohnuma H, Sugiyama K, Manaka H, Sasaki H, Fukuyama H. Prevalence of diabetes and impaired glucose tolerance in Funagata area, Japan. Diabetes Care. 1993; 16(4): 570-4

Sekita A, Arima H, Ninomiya T, Ohara T, Doi Y, Hirakawa Y, Fukuhara M, Hata J, Yonemoto K, Ga Y, Kitazono T, Kanba S, Kiyohara Y. Elevated depressive symptoms in metabolic syndrome in a general population of Japanese men: a cross-sectional study. BMC Public Health. 2013; 13: 862

Shab-Bidar S, Hosseini-Esfahani F, Mirmiran P, Hosseinpour-Niazi S, Azizi F. Metabolic syndrome profiles, obesity measures and intake of dietary fatty acids in adults: Tehran Lipid and Glucose Study. J Hum Nutr Diet. 2014; 27 Suppl 2: 98–108

Shah A, Afzal M. Prevalence of diabetes and hypertension and association with various risk factors among different Muslim populations of Manipur, India. J Diabetes Metab Disord. 2013; 12(1): 52

Shah C, Sheth NR, Solanki B, Shah N. To assess the prevalence of impaired glucose tolerance and impaired fasting glucose in Western Indian population. J Assoc Physicians India. 2013; 61(3): 179–84

Shan Y, Zhang Q, Liu Z, Hu X, Liu D. Prevalence and risk factors associated with chronic kidney disease in adults over 40 years: a population study from Central China. Nephrology (Carlton). 2010; 15(3): 354-61

Sharma SK, Ghimire A, Radhakrishnan J, Thapa L, Shrestha NR, Paudel N, Gurung K, R M, Budathoki A, Baral N, Brodie D. Prevalence of hypertension, obesity, diabetes, and metabolic syndrome in Nepal. Int J Hypertens. 2011; 821971

Sheng C-S, Liu M, Kang Y-Y, Wei F-F, Zhang L, Li G-L, Dong Q, Huang Q-F, Li Y, Wang J-G. Prevalence, awareness, treatment and control of hypertension in elderly Chinese. Hypertens Res. 2013; 36(9): 824–8

Shera AS, Basit A, Fawwad A, Hakeem R, Ahmedani MY, Hydrie MZI, Khwaja IA. Pakistan National Diabetes Survey: prevalence of glucose intolerance and associated factors in the Punjab Province of Pakistan. Prim Care Diabetes. 2010; 4(2): 79–83

Shera AS, Rafique G, Khawaja IA, Baqai S, King H. Pakistan National Diabetes Survey: prevalence of glucose intolerance and associated factors in Baluchistan province. Diabetes Res Clin Pract. 1999; 44(1): 49-58

Shi Z, Yuan B, Zhang C, Zhou M, Holmboe-Ottesen G. Egg consumption and the risk of diabetes in adults, Jiangsu, China. Nutrition. 2011; 27(2): 194–8

Shimizu Y, Nakazato M, Sekita T, Kadota K, Yamasaki H, Takamura N, Aoyagi K, Kusano Y, Maeda T. Association between alkaline phosphatase and hypertension in a rural Japanese population: the Nagasaki Islands study. J Physiol Anthropol. 2013; 32(1): 10

Shin CY, Yun KE, Park HS. Blood pressure has a greater impact on cardiovascular mortality than other components of metabolic syndrome in Koreans. Atherosclerosis. 2009; 205(2): 614–9

Shirani S, Heidari K, Sabzghabaee AM, Mirmoghtadaee P, Hoseini L, Aalifar H, Fadaei H, Esnaashari H, Soltani R. The modifiable noncommunicable risk factors among an Iranian population. Southeast Asian J Trop Med Public Health. 2012; 43(5): 1227-32

Shirani S, Kelishadi R, Sarrafzadegan N, Khosravi A, Sadri G, Amani A, Heidari S, Ramezani MA. Awareness, treatment and control of hypertension, dyslipidaemia and diabetes mellitus in an Iranian population: the IHHP study. East Mediterr Health J. 2009; 15(6): 1455–63

Shrestha UK, Singh DL, Bhattarai MD. The prevalence of hypertension and diabetes defined by fasting and 2-h plasma glucose criteria in urban Nepal. Diabet Med. 2006; 23(10): 1130-5

Silva H, Hernandez-Hernandez R, Vinueza R, Velasco M, Boissonnet CP, Escobedo J, Silva HE, Pramparo P, Wilson E, CARMELA Study Investigators. Cardiovascular risk awareness, treatment, and control in urban Latin America. Am J Ther. 2010; 17(2): 159–66

Silva-Matos C, Gomes A, Azevedo A, Damasceno A, Prista A, Lunet N. Diabetes in Mozambique: prevalence, management and healthcare challenges. Diabetes Metab. 2011; 37(3): 237-44

Simmons D, McKenzie A, Eaton S, Shaw J, Zimmet P. Prevalence of diabetes in rural Victoria. Diabetes Res Clin Pract. 2005; 70(3): 287-90

Singapore Cardiovascular Cohort Study Metabolics Data, as provided by the Global Burden of Disease 2010 Metabolics Expert Group

Singh AK, Mani K, Krishnan A, Aggarwal P, Gupta SK. Prevalence, awareness, treatment and control of diabetes among elderly persons in an urban slum of delhi. Indian J Community Med. 2012; 37(4): 236-9

Singh DL, Bhattarai MD. High prevalence of diabetes and impaired fasting glycaemia in urban Nepal. Diabet Med. 2003; 20(2): 170-1

Smith KJ, Blizzard L, McNaughton SA, Gall SL, Dwyer T, Venn AJ. Daily eating frequency and cardiometabolic risk factors in young Australian adults: cross-sectional analyses. Br J Nutr. 2012; 108(6): 1086–94

Söderberg S, Zimmet P, Tuomilehto J, de Courten M, Dowse GK, Chitson P, Gareeboo H, Alberti KGMM, Shaw JE. Increasing prevalence of Type 2 diabetes mellitus in all ethnic groups in Mauritius. Diabet Med. 2005; 22(1): 61-8

Soegondo S, Widyahening IS, Istantho R, Yunir E. Prevalence of diabetes among suburban population of Ternate--a small remote island in the eastern part of Indonesia. Acta Med Indones. 2011; 43(2): 99-104

Solet J-L, Baroux N, Pochet M, Benoit-Cattin T, De Montera A-M, Sissoko D, Favier F, Fagot-Campagna A. Prevalence of type 2 diabetes and other cardiovascular risk factors in Mayotte in 2008: the MAYDIA study. Diabetes Metab. 2011; 37(3): 201-7

Song K-H, Nam-Goomg IS, Han S-M, Kim M-S, Lee E-J, Lee YS, Lee MS, Yoon S, Lee K-U, Park J-Y. Change in prevalence and 6-year incidence of diabetes and impaired fasting glucose in Korean subjects living in a rural area. Diabetes Res Clin Pract. 2007; 78(3): 378-84

Soria MLB, Sy RG, Vega BS, Ty-Willing T, Abenir-Gallardo A, Velandria F, Punzalan FE. The incidence of type 2 diabetes mellitus in the Philippines: a 9-year cohort study. Diabetes Res Clin Pract. 2009; 86(2): 130-3

Soriguer F, Colomo N, Olveira G, Garcia-Fuentes E, Esteva I, Ruiz de Adana MS, Morcillo S, Porras N, Valdes S, Rojo-Martinez G. White rice consumption and risk of type 2 diabetes. Clin Nutr. 2013; 32(3): 481–4

Soriguer F, Goday A, Bosch-Comas A, Bordiú E, Calle-Pascual A, Carmena R, Casamitjana R, Castaño L, Castell C, Catalá M, Delgado E, Franch J, Gaztambide S, Girbés J, Gomis R, Gutiérrez G, López-Alba A, Martínez-Larrad MT, Menéndez E, Mora-Peces I, Ortega E, Pascual-Manich G, Rojo-Martínez G, Serrano-Rios M, Valdés S, Vázquez JA, Vendrell J. Prevalence of diabetes mellitus and impaired glucose regulation in Spain: the Di@bet.es Study. Diabetologia. 2012; 55(1): 88-93

Sossa C, Delisle H, Agueh V, Sodjinou R, Ntandou G, Makoutode M. Lifestyle and dietary factors associated with the evolution of cardiometabolic risk over four years in West-African adults: the Benin study. J Obes. 2013; 2013: 298024

Soysal A, Demiral Y, Soysal D, Uçku R, Köseoglu M, Aksakoglu G. The prevalence of metabolic syndrome among young adults in Izmir, Turkey. Anatol J Cardiol. 2005; 5(3): 196-201

Sun G-Z, Li Z, Guo L, Zhou Y, Yang H-M, Sun Y-X. High prevalence of dyslipidemia and associated risk factors among rural Chinese adults. Lipids Health Dis. 2014; 13: 189

Sun K, Li F, Lin D, Qi Y, Xu M, Li N, Huang C, Ren M, Li Y, Yan L. Serum gamma - glutamyltransferase is associated with albuminuria: a population-based study. PLoS One. 2014; 9(12): e114970

Swai AB, Mclarty DG, Mtinangi BL, Tatala S, Kitange HM, Mlingi N, Rosling H, Howlett WP, Brubaker GR, Alberti KG. Diabetes is not caused by cassava toxicity. A study in a Tanzanian community. Diabetes Care. 1992; 15(10): 1378-85

Sy RG, Morales DD, Dans AL, Paz-Pacheco E, Punzalan FER, Abelardo NS, Duante CA. Prevalence of atherosclerosis-related risk factors and diseases in the Philippines. J Epidemiol. 2012; 22(5): 440-7

Takata Y, Ansai T, Soh I, Awano S, Nakamichi I, Akifusa S, Goto K, Yoshida A, Fujii H, Fujisawa R, Sonoki K. Serum total cholesterol concentration and 10-year mortality in an 85-year-old population. Clin Interv Aging. 2014; 9: 293–300

Tamosiunas A, Luksiene D, Baceviciene M, Bernotiene G, Radisauskas R, Malinauskiene V, Kranciukaite-Butylkiniene D, Virviciute D, Peasey A, Bobak M. Health factors and risk of all-cause, cardiovascular, and coronary heart disease mortality: findings from the MONICA and HAPIEE studies in Lithuania. PLoS One. 2014; 9(12): e114283

Tan A, Gao Y, Yang X, Zhang H, Qin X, Mo L, Peng T, Xia N, Mo Z. Low serum osteocalcin level is a potential marker for metabolic syndrome: results from a Chinese male population survey. Metab Clin Exp. 2011; 60(8): 1186–92

Temmar M, Labat C, Benkhedda S, Charifi M, Thomas F, Bouafia MT, Bean K, Darne B, Safar ME, Benetos A. Prevalence and determinants of hypertension in the Algerian Sahara. J Hypertens. 2007; 25(11): 2218-26

The InterASIA Collaborative Group. Cardiovascular risk factor levels in urban and rural Thailand - The International Collaborative Study of Cardiovascular Disease in Asia (InterASIA). Eur J Cardiovasc Prev Rehabil. 2003; 10(4): 249-57

Thomas F, Bean K, London G, Danchin N, Pannier B. [Incidence of arterial hypertension in French population after 60 years]. Ann Cardiol Angeiol (Paris). 2012; 61(3): 140–4

Thuesen BH, Cerqueira C, Aadahl M, Ebstrup JF, Toft U, Thyssen JP, Fenger RV, Hersoug L-G, Elberling J, Pedersen O, Hansen T, Johansen JD, Jørgensen T, Linneberg A. Cohort Profile: the Health2006 cohort, research centre for prevention and health. Int J Epidemiol. 2014; 43(2): 568–75

Tillin T, Hughes AD, Mayet J, Whincup P, Sattar N, Forouhi NG, McKeigue PM, Chaturvedi N. The relationship between metabolic risk factors and incident cardiovascular disease in Europeans, South Asians, and African Caribbeans: SABRE (Southall and Brent Revisited) -- a prospective population-based study. J Am Coll Cardiol. 2013; 61(17): 1777–86

Torquato MT, Montenegro Junior RM, Viana LA, de Souza RA, Lanna CM, Lucas JC, Bidurin C, Foss MC. Prevalence of diabetes mellitus and impaired glucose tolerance in the urban population aged 30-69 years in Ribeirao Preto (Sao Paulo), Brazil. Sao Paulo Med J. 2003; 121(6): 224-30

Truven Health Analytics. United States MarketScan Claims and Medicare Data - 2000. Ann Arbor, United States: Truven Health Analytics

Truven Health Analytics. United States MarketScan Claims and Medicare Data - 2010. Ann Arbor, United States: Truven Health Analytics

Truven Health Analytics. United States MarketScan Claims and Medicare Data - 2012. Ann Arbor, United States: Truven Health Analytics

Tufton N, Chowdhury T. Prevalence of Diabetes on Santa Cruz Island in Galapagos Archipelago. Prev Chronic Dis. 2015; E94

Tuomilehto J, Nissinen A, Kivelä SL, Pekkanen J, Kaarsalo E, Wolf E, Aro A, Punsar S, Karvonen MJ. Prevalence of diabetes mellitus in elderly men aged 65 to 84 years in eastern and western Finland. Diabetologia. 1986; 29(9): 611-5

Turin TC, Murakami Y, Miura K, Rumana N, Kita Y, Hayakawa T, Okamura T, Okayama A, Ueshima H, NIPPON DATA80/90 Research Group. Hypertension and life expectancy among Japanese: NIPPON DATA80. Hypertens Res. 2012; 35(9): 954–8

Ueshima H, Kita Y, Choudhury SR, Asia Pacific Cohort Studies Collaboration. Japan - Konan Health and Nutrition Study 1987-1995

Ulmer H, Kelleher CC, Fitz-Simon N, Diem G, Concin H. Secular trends in cardiovascular risk factors: an age-period cohort analysis of 698,954 health examinations in 181,350 Austrian men and women. J Intern Med. 2007; 261(6): 566-76

Unal B, Sozmen K, Ucku R, Ergor G, Soysal A, Baydur H, Meseri R, Simsek H, Gercekliogl u G, Doganay S, Budak R, Kilic B, Gunay T, Ergor A, Demiral Y, Aslan O, Cimrin D, Akvardar Y, Tuncel P. High prevalence of cardiovascular risk factors in a Western urban Turkish population: a community-based study. Anatol J Cardiol. 2013; 13(1): 9–17

University of the West Indies. Jamaica Health and Lifestyle Survey 2007-2008

Vaara JP, Kyrolainen H, Fogelholm M, Santtila M, Hakkinen A, Hakkinen K, Vasankari T. Associations of leisure time, commuting, and occupational physical activity with physical fitness and cardiovascular risk factors in young men. J Phys Act Health. 2014; 11(8): 1482–91

Vaz NC, Ferreira AM, Kulkarni MS, Vaz FS. Prevalence of diabetes mellitus in a rural population of Goa, India. Natl Med J India. 2011; 24(1): 16-8

Verrillo A, de Teresa A, La Rocca S, Giarrusso PC. Prevalence of diabetes mellitus and impaired glucose tolerance in a rural area of Italy. Diabetes Res. 1985; 2(6): 301-6

Vetrano DL, Martone AM, Mastropaolo S, Tosato M, Colloca G, Marzetti E, Onder G, Bernabei R, Landi F. Prevalence of the seven cardiovascular health metrics in a Mediterranean country: results from a cross-sectional study. Eur J Public Health. 2013; 23(5): 858–62

Wakabayashi I. Cross-sectional relationship between alcohol consumption and prevalence of metabolic syndrome in Japanese men and women. J Atheroscler Thromb. 2010; 17(7): 695–704

Wakasugi M, James Kazama J, Narita I. Associations between the intake of miso soup and Japanese pickles and the estimated 24-hour urinary sodium excretion: a population-based cross-sectional study. Intern Med. 2015; 54(8): 903–10

Wandell PE, Carlsson AC, de Faire U, Hellenius M-L. Prevalence of blood lipid disturbances in Swedish and foreign-born 60-year-old men and women in Stockholm, Sweden. Nutr Metab Cardiovasc Dis. 2011; 21(3): 173–81

Wang C, Hou X, Bao Y, Pan J, Zuo Y, Zhong W, Jia W, Xiang K. The metabolic syndrome increased risk of cardiovascular events in Chinese--a community based study. Int J Cardiol. 2010; 139(2): 159–65

Wang C, Li L, Wang L, Ping Z, Flory MT, Wang G, Xi Y, Li W. Evaluating the risk of type 2 diabetes mellitus using artificial neural network: an effective classification approach. Diabetes Res Clin Pract. 2013; 100(1): 111–8

Wang F, Ye P, Luo L, Xiao W, Qi L, Bian S, Wu H, Sheng L, Xiao T, Xu R. Association of serum lipids with arterial stiffness in a population-based study in Beijing. Eur J Clin Invest. 2011; 41(9): 929–36

Wang H, Qiu Q, Tan LL, Liu T, Deng XQ, Chen YM, Chen W, Yu XQ, Hu BJ, Chen WQ. Prevalence and determinants of diabetes and impaired fasting glucose among urban community-dwelling adults in Guangzhou, China. Diabetes Metab. 2009; 35(5): 378-84

Wang T, Bi Y, Xu M, Huang Y, Xu Y, Li X, Wang W, Ning G. Serum uric acid associates with the incidence of type 2 diabetes in a prospective cohort of middle-aged and elderly Chinese. Endocrine. 2011; 40(1): 109–16

Wang W, Zhao D, Sun JY, Liu J, Qin LP, Wu ZS. Impact of new criterion of glucose level on the prevalence of impaired fasting glucose and risk of ischemic cardiovascular diseases. Chin J Intern Med. 2007; 46(1): 20-4

Ware LJ, Rennie KL, Kruger HS, Kruger IM, Greeff M, Fourie CMT, Huisman HW, Scheepers JDW, Uys AS, Kruger R, Van Rooyen JM, Schutte R, Schutte AE. Evaluation of waist-to-height ratio to predict 5 year cardiometabolic risk in sub-Saharan African adults. Nutr Metab Cardiovasc Dis. 2014; 24(8): 900–7

Warsy AS, el-Hazmi MA. Diabetes mellitus, hypertension and obesity--common multifactorial disorders in Saudis. East Mediterr Health J. 1999; 5(6): 1236-42

Wei Q, Sun J, Huang J, Zhou HY, Ding YM, Tao YC, He SM, Liu YL, Niu JQ. Prevalence of hypertension and associated risk factors in Dehui City of Jilin Province in China. J Hum Hypertens. 2015; 29(1): 64–8

Welin L, Eriksson H, Larsson B, Ohlson LO, Svärdsudd K, Tibblin G. Hyperinsulinaemia is not a major coronary risk factor in elderly men. The study of men born in 1913. Diabetologia. 1992; 35(8): 766-70

Whisman MA. Loneliness and the metabolic syndrome in a population-based sample of middle-aged and older adults. Health Psychol. 2010; 29(5): 550–4

Wijewardene K, Mohideen MR, Mendis S, Fernando DS, Kulathilaka T, Weerasekara D, Uluwitta P. Prevalence of hypertension, diabetes and obesity: baseline findings of a population based survey in four provinces in Sri Lanka. Ceylon Med J. 2005; 50(2): 62-70

Wilks R, Rotimi C, Bennett F, McFarlane-Anderson N, Kaufman JS, Anderson SG, Cooper RS, Cruickshank JK, Forrester T. Diabetes in the Caribbean: results of a population survey from Spanish Town, Jamaica. Diabet Med. 1999; 16(10): 875-83

Williams ED, Tapp RJ, Magliano DJ, Shaw JE, Zimmet PZ, Oldenburg BF. Health behaviours, socioeconomic status and diabetes incidence: the Australian Diabetes Obesity and Lifestyle Study (AusDiab). Diabetologia. 2010; 53(12): 2538-45

Wiltink J, Beutel ME, Till Y, Ojeda FM, Wild PS, Munzel T, Blankenberg S, Michal M. Prevalence of distress, comorbid conditions and well being in the general population. J Affect Disord. 2011; 130(3): 429–37

Wiltink J, Michal M, Wild PS, Schneider A, Konig J, Blettner M, Munzel T, Schulz A, Weber M, Fottner C, Pfeiffer N, Lackner K, Beutel ME. Associations between depression and diabetes in the community: do symptom dimensions matter? Results from the Gutenberg Health Study. PLoS One. 2014; 9(8): e105499

Winkler G, Hidvegi T, Vandorfi G, Balogh S, Jermendy G. Prevalence of undiagnosed abnormal glucose tolerance in adult patients cared for by general practitioners in Hungary. Results of a risk-stratified screening based on FINDRISC questionnaire. Med Sci Monit. 2013; 19: 67–72

Wittek A, Sokalski B, Grzeszczak W, Strojek K. Prevalence of diabetes and cardiovascular risk factors of industrial area in southern Poland. Exp Clin Endocrinol Diabetes. 2009; 117(7): 350-3

World Health Organization (WHO), Ministry of Health (Mongolia), National Medical Research Institute (Mongolia), Health Sciences University (Mongolia), National Oncology Center of Mongolia (Mongolia). Mongolia STEPS Noncommunicable Disease Risk Factors Survey 2005

World Health Organization (WHO), Ministry of Health (Mongolia), National Medical Research Institute (Mongolia), Health Sciences University (Mongolia), National Oncology Center of Mongolia (Mongolia). Mongolia STEPS Noncommunicable Disease Risk Factors Survey 2009

World Health Organization (WHO), Ministry of Health (Seychelles), Institute of Social and Preventive Medicine, University of Lausanne (Switzerland), University Hospital Center (Switzerland). Seychelles STEPS Noncommunicable Disease Risk Factors Survey 2004

World Health Organization (WHO), Ministry of Health and Medical Education (Iran), Center for Non-Communicable Diseases Control (Iran). Iran STEPS Noncommunicable Disease Risk Factors Survey 2005

World Health Organization (WHO), Ministry of Health and Medical Education (Iran), Center for Non-Communicable Diseases Control (Iran). Iran STEPS Noncommunicable Disease Risk Factors Survey 2007

World Health Organization (WHO). Cape Verde STEPS Noncommunicable Disease Risk Factors Survey 2007

World Health Organization (WHO). Comoros STEPS Noncommunicable Disease Risk Factors Survey 2011

World Health Organization (WHO). Dominica STEPS Noncommunicable Disease Risk Factors Survey 2007-2008

World Health Organization (WHO). Georgia STEPS Noncommunicable Disease Risk Factors Survey 2010

World Health Organization (WHO). Guinea - Conakry STEPS Noncommunicable Disease Risk Factors Survey 2009

World Health Organization (WHO). Liberia STEPS Noncommunicable Disease Risk Factors Survey 2011

World Health Organization (WHO). Papua New Guinea STEPS Noncommunicable Disease Risk Factors Survey 2007-2008

World Health Organization (WHO). Sao Tome and Principe STEPS Noncommunicable Disease Risk Factors Survey 2008

World Health Organization (WHO). Vietnam - C?n Th? STEPS Noncommunicable Disease Risk Factors Survey 2005

Wu D, Kendall D, Lunt H, Willis J, Darlow B, Frampton C. Prevalence of Type 1 diabetes in New Zealanders aged 0-24 years. N Z Med J. 2005; 118(1218): U1557

Wu J, Qiu L, Yan W, Cheng X, Wu W, Guo X, Ding H, Han H, Han S, Zhu G. Serum ?-glutamyltransferase and uric acid levels are associated with impaired fasting glucose in adults from Inner Mongolia, China. BMC Public Health. 2013; 13: 294

Wu Y, Li H, Loos RJ, Yu Z, Ye X, Chen L, Pan A, Hu FB, Lin X. Common variants in CDKAL1, CDKN2A/B, IGF2BP2, SLC30A8, and HHEX/IDE genes are associated with type 2 diabetes and impaired fasting glucose in a Chinese Han population. Diabetes. 2008; 57(10): 2834-42

Wu Y, Li M, Xu M, Bi Y, Li X, Chen Y, Ning G, Wang W. Low serum total bilirubin concentrations are associated with increased prevalence of metabolic syndrome in Chinese. J Diabetes. 2011; 3(3): 217–24

Wyka J, Biernat J, Kiedik D. Nutritional determination of the health status in Polish elderly people from an urban environment. J Nutr Health Aging. 2010; 14(1): 67-71

Xiang Y, Huang G, Zhou W, Che Z, Zhou P, Zhou Z. Prevalence of metabolic syndrome (MetS) in Chinese subjects gradually increased with impaired glucose homeostasis: a multicenter, clinical based, cross-sectional study. BMC Public Health. 2012; 12: 675

Xie J, Guan F, Wang J-H, Hu D-Y. Reasons for the upsetting cholesterol level during the community investigation from residents, physicians, and social aspects: the China Cholesterol Education Program (CCEP). Chin Med J (Engl). 2011; 124(19): 3030–4

Xin Z, Yuan M-X, Li H-X, Hua L, Feng J-P, Shi J, Zhu X-R, Cao X, Yang J-K. Evaluation for fasting and 2-hour glucose and HbA1c for diagnosing diabetes based on prevalence of retinopathy in a Chinese population. PLoS One. 2012; 7(7): e40610

Xu L, Jiang CQ, Lam TH, Cheng KK, Yue XJ, Lin JM, Zhang WS, Thomas GN. Impact of impaired fasting glucose and impaired glucose tolerance on arterial stiffness in an older Chinese population: the Guangzhou Biobank Cohort Study-CVD. Metab Clin Exp. 2010; 59(3): 367–72

Xu L, Jiang CQ, Schooling CM, Zhang WS, Cheng KK, Lam TH. Prediction of 4-year incident diabetes in older Chinese: recalibration of the Framingham diabetes score on Guangzhou Biobank Cohort Study. Prev Med. 2014; 69: 63–8

Xu S, Ming J, Xing Y, Gao B, Yang C, Ji Q, Chen G. Regional differences in diabetes prevalence and awareness between coastal and interior provinces in China: a population-based cross-sectional study. BMC Public Health. 2013; 299

Xu Y, Wang L, He J, Bi Y, Li M, Wang T, Wang L, Jiang Y, Dai M, Lu J, Xu M, Li Y, Hu N, Li J, Mi S, Chen CS, Li G, Mu Y, Zhao J, Kong L, Chen J, Lai S, Wang W, Zhao W, Ning G. Prevalence and control of diabetes in Chinese adults. JAMA. 2013; 310(9): 948-59

Yamamoto Kimura L, Zamora Gonzalez J, Garcia de la Torre G, Cardoso Saldana G, Fajardo Gutierrez A, Ayala Barajas C, Posadas Romero C. Prevalence of high blood pressure and associated coronary risk factors in an adult population of Mexico city. Arch Med Res. 1998; 29(4): 341-9

Yang W, Lu J, Weng J, Jia W, Ji L, Xiao J, Shan Z, Liu J, Tian H, Ji Q, Zhu D, Ge J, Lin L, Chen L, Guo X, Zhao Z, Li Q, Zhou Z, Shan G, He J; China National Diabetes and Metabolic Disorders Study Group. Prevalence of diabetes among men and women in China. N Engl J Med. 2010; 36(12): 1090-101

Yang Y-N, Xie X, Ma Y-T, Li X-M, Fu Z-Y, Ma X, Huang D, Chen B-D, Liu F, Huang Y, Liu C, Zheng Y-Y, Baituola G, YU Z-X, Chen Y. Type 2 Diabetes in Xinjiang Uygur Autonomous Region, China. PLoS One. 2012; 7(4)

Yang Z-J, Liu J, Ge J-P, Chen L, Zhao Z-G, Yang W-Y, China National Diabetes and Metabolic Disorders Study Group. Prevalence of cardiovascular disease risk factor in the Chinese population: the 2007-2008 China National Diabetes and Metabolic Disorders Study. Eur Heart J. 2012; 33(2): 213–20

Yi Z, Jing J, Xiu-ying L, Hongxia X, Jianjun Y, Yuhong Z. Prevalence of the metabolic syndrome among rural original adults in NingXia, China. BMC Public Health. 2010; 10: 140

Yim-Lui Cheung C, Wong TY, Lamoureux EL, Sabanayagam C, Li J, Lee J, Tai ES. C-Reactive Protein and Retinal Microvascular Caliber in a Multiethnic Asian Population. Am J Epidemiol. 2009; 171(2): 206-13

Yoon YS, Oh SW, Baik HW, Park HS, Kim WY. Alcohol consumption and the metabolic syndrome in Korean adults: the 1998 Korean National Health and Nutrition Examination Survey. Am J Clin Nutr. 2004; 80(1): 217-24

Yu S, Guo X, Yang H, Zheng L, Sun Y. An update on the prevalence of metabolic syndrome and its associated factors in rural northeast China. BMC Public Health. 2014; 14: 877

Zafar J, Bhatti F, Akhtar N, Rasheed U, Bashir R, Humayun S, Waheed A, Younus F, Nazar M, Umaimato. Prevalence and risk factors for diabetes mellitus in a selected urban population of a city in Punjab. J Pak Med Assoc. 2011; 61(1): 40-7

Zaman FA, Borang A. Prevalence of diabetes mellitus amongst rural hilly population of North Eastern India and its relationship with associated risk factors and related co-morbidities. J Nat Sci Biol Med. 2014; 5(2): 383–8

Zargar AH, Khan AK, Masoodi SR, Laway BA, Wani AI, Bashir MI, Dar FA. Prevalence of type 2 diabetes mellitus and impaired glucose tolerance in the Kashmir Valley of the Indian subcontinent. Diabetes Res Clin Pract. 2000; 47(2): 135-46

Zato?ska K, Ilow R, Regulska-Ilow B, Ró?a?ska D, Szuba A, Wo?yniec M, Einhorn J, Vatten L, Asvold BO, Ma?czuk M, Zato?ski WA. Prevalence of diabetes mellitus and IFG in the prospective cohort 'PONS' study - baseline assessment. Ann Agric Environ Med. 2011; 18(2): 265-9

Zhang L, Qin L-Q, Liu A-P, Wang P-Y. Prevalence of risk factors for cardiovascular disease and their associations with diet and physical activity in suburban Beijing, China. J Epidemiol. 2010; 20(3): 237–43

Zhang L, Zhang W-H, Zhang L, Wang P-Y. Prevalence of overweight/obesity and its associations with hypertension, diabetes, dyslipidemia, and metabolic syndrome: a survey in the suburban area of Beijing, 2007. Obes Facts. 2011; 4(4): 284-9

Zhang W-H, Xue P, Yao M-Y, Chang H-M, Wu Y, Zhang L. Prevalence of metabolic syndrome and its relationship with physical activity in suburban Beijing, China. Ann Nutr Metab. 2013; 63(4): 298–304

Zhang YH, Ma WJ, Thomas GN, Xu YJ, Lao XQ, Xu XJ, Song XL, Xu HF, Cai QM, Xia L, Nie SP, Deng HH, Yu IT. Diabetes and pre-diabetes as determined by glycated haemoglobin A1c and glucose levels in a developing southern Chinese population. PLoS One. 2012; 7(5): e37260

Zhao X, Zhu X, Zhang H, Zhao W, Li J, Shu Y, Li S, Yang M, Cai L, Zhou J, Li Y. Prevalence of diabetes and predictions of its risks using anthropometric measures in southwest rural areas of China. BMC Public Health. 2012; 821

Zhao Y, Yang K, Wang F, Liang Y, Peng Y, Shen R, Wong T, Wang N. Associations between metabolic syndrome and syndrome components and retinal microvascular signs in a rural Chinese population: the Handan Eye Study. Graefes Arch Clin Exp Ophthalmol. 2012; 250(12): 1755–63

Zhou H, Guo Z, Yu L, Hu X, Xu B, Liu H, Wu M, Zhou Z. Evidence on the applicability of the ATPIII, IDF and CDS metabolic syndrome diagnostic criteria to identify CVD and T2DM in the Chinese population from a 6.3-year cohort study in mid-eastern China. Diabetes Res Clin Pract. 2010; 90(3): 319–25

Zhou X, Guan H, Zheng L, Li Z, Guo X, Yang H, Yu S, Sun G, Li W, Hu W, Guo L, Pan G, Xing L, Zhang Y, Sun Y. Prevalence and awareness of diabetes mellitus among a rural population in China: results from Liaoning Province. Diabet Med. 2015; 32(3): 332-42

Zhou X, Ji L, Luo Y, Han X, Zhang X, Sun X, Ren Q, Qiao Q. Risk factors associated with the presence of diabetes in Chinese communities in Beijing. Diabetes Res Clin Pract. 2009; 86(3): 233–8

Zhou X, Pang Z, Gao W, Wang S, Zhang L, Ning F, Qiao Q. Performance of an A1C and fasting capillary blood glucose test for screening newly diagnosed diabetes and pre-diabetes defined by an oral glucose tolerance test in Qingdao, China. Diabetes Care. 2010; 33(3): 545-50

Zhou XH, Ji LN, Luo YY, Zhang XY, Han XY, Qiao Q. Performance of HbA(1c) for detecting newly diagnosed diabetes and pre-diabetes in Chinese communities living in Beijing. Diabet Med. 2009; 26(12): 1262-8

Zuhaid M, Zahir KK, Diju IU. Knowledge and perceptions of diabetes in urban and semi urban population of Peshawar, Pakistan. J Ayub Med Coll Abbottabad. 2012; 24(1): 105-8

Supporting tables

Estimated observed age-standardized rates per 100,000 population of tuberculosis incidence, prevalence, and mortality by SDI values and GBD regions (1990-2015)

Region	Year	Measure name	Age-standardized rate	Age-standardized rate (upper UI)	Age-standardized rate (lower UI)	SDI
Andean Latin America	1990	Deaths	45.08776	52.48011	25.7536	0.601325
Andean Latin America	1995	Deaths	32.69077	36.5084	21.54882	0.639461
Andean Latin America	2000	Deaths	19.44014	26.61469	16.99428	0.670974
Andean Latin America	2005	Deaths	12.94081	21.85466	10.81688	0.697096
Andean Latin America	2010	Deaths	10.07427	17.26081	8.223418	0.71593
Andean Latin America	2015	Deaths	8.021127	13.7786	6.411995	0.72763
Australasia	1990	Deaths	0.6143669	0.651797	0.5800975	0.840012
Australasia	1995	Deaths	0.5183212	0.552121	0.4889815	0.85437
Australasia	2000	Deaths	0.3777746	0.4072935	0.3507334	0.868379
Australasia	2005	Deaths	0.2859598	0.3077561	0.2649559	0.877539
Australasia	2010	Deaths	0.2343584	0.2537572	0.2165849	0.883589
Australasia	2015	Deaths	0.1940164	0.2146099	0.1765191	0.899057
Caribbean	1990	Deaths	9.12256	11.36072	7.47213	0.617814
Caribbean	1995	Deaths	7.66401	10.48109	6.3658	0.643936
Caribbean	2000	Deaths	5.784394	8.426455	4.765159	0.662542
Caribbean	2005	Deaths	4.98236	7.708997	4.055435	0.684099
Caribbean	2010	Deaths	4.40973	6.872485	3.468326	0.701189
Caribbean	2015	Deaths	3.868697	6.108238	2.944126	0.718367
Central Asia	1990	Deaths	11.8257	15.07184	10.84452	0.692146
Central Asia	1995	Deaths	16.79969	18.98949	14.57928	0.710885
Central Asia	2000	Deaths	18.12281	19.50941	14.0373	0.713923
Central Asia	2005	Deaths	14.85189	16.40959	11.04766	0.717763
Central Asia	2010	Deaths	9.853176	10.99461	7.109218	0.73416
Central Asia	2015	Deaths	7.497567	8.542369	5.377879	0.75339

Region	Year	Measure name	Age-standardized rate	Age-standardized rate (upper UI)	Age-standardized rate (lower UI)	SDI
Central Europe	1990	Deaths	4.644161	4.831375	4.394154	0.790899
Central Europe	1995	Deaths	4.685606	4.878566	4.456074	0.813739
Central Europe	2000	Deaths	3.545461	3.706341	3.395306	0.830631
Central Europe	2005	Deaths	2.687033	2.827896	2.571642	0.847861
Central Europe	2010	Deaths	1.95977	2.082059	1.859713	0.864726
Central Europe	2015	Deaths	1.408289	1.54189	1.303779	0.877895
Central Latin America	1990	Deaths	12.84481	13.29431	12.35349	0.587256
Central Latin America	1995	Deaths	9.096785	9.443501	8.757981	0.626313
Central Latin America	2000	Deaths	6.071781	6.392597	5.838161	0.660288
Central Latin America	2005	Deaths	4.33181	4.632918	4.123728	0.69047
Central Latin America	2010	Deaths	3.38158	3.645358	3.211991	0.71747
Central Latin America	2015	Deaths	2.729414	2.964329	2.561658	0.750288
Central Sub-Saharan Africa	1990	Deaths	123.0931	195.227	72.13969	0.374714
Central Sub-Saharan Africa	1995	Deaths	119.8393	192.2082	70.26181	0.388388
Central Sub-Saharan Africa	2000	Deaths	122.5348	215.6073	67.4541	0.397329
Central Sub-Saharan Africa	2005	Deaths	117.4818	224.2599	61.11993	0.408764
Central Sub-Saharan Africa	2010	Deaths	102.9516	207.103	52.00188	0.429405
Central Sub-Saharan Africa	2015	Deaths	90.27635	190.2691	44.74809	0.459795
East Asia	1990	Deaths	20.62702	25.67707	14.52637	0.633319
East Asia	1995	Deaths	14.68221	18.5302	11.17895	0.666821
East Asia	2000	Deaths	10.56434	13.47117	8.347295	0.688894
East Asia	2005	Deaths	7.489061	10.09333	6.200875	0.71325
East Asia	2010	Deaths	4.803712	7.146656	4.256732	0.740004
East Asia	2015	Deaths	3.469149	5.331192	3.015945	0.763711
Eastern Europe	1990	Deaths	6.023856	6.350883	5.726793	0.803907
Eastern Europe	1995	Deaths	10.47857	11.06105	9.935015	0.821964
Eastern Europe	2000	Deaths	13.03026	13.78152	12.27318	0.822386
Eastern Europe	2005	Deaths	13.3762	14.14365	12.66825	0.836392

Region	Year	Measure name	Age-standardized rate	Age-standardized rate (upper UI)	Age-standardized rate (lower UI)	SDI
Eastern Europe	2010	Deaths	8.36956	8.83834	7.92129	0.852063
Eastern Europe	2015	Deaths	5.767319	6.282576	5.344111	0.857602
Eastern Sub-Saharan Africa	1990	Deaths	110.8959	145.7595	84.04543	0.281544
Eastern Sub-Saharan Africa	1995	Deaths	104.5656	135.2673	80.43492	0.318378
Eastern Sub-Saharan Africa	2000	Deaths	95.74614	116.7632	74.70579	0.351583
Eastern Sub-Saharan Africa	2005	Deaths	82.32882	99.95204	61.00378	0.380305
Eastern Sub-Saharan Africa	2010	Deaths	69.34613	87.56031	48.4827	0.417935
Eastern Sub-Saharan Africa	2015	Deaths	60.1277	80.10948	38.7518	0.461506
Global	1990	Deaths	38.30006	46.74847	31.88863	0.600264
Global	1995	Deaths	33.37294	40.47794	27.78737	0.627114
Global	2000	Deaths	28.79493	35.10084	24.16688	0.647289
Global	2005	Deaths	24.23698	29.85273	20.78274	0.667388
Global	2010	Deaths	19.51306	24.16982	16.43354	0.690595
Global	2015	Deaths	16.04776	20.07927	13.14564	0.714202
High-income Asia Pacific	1990	Deaths	7.170949	7.456916	6.897102	0.831853
High-income Asia Pacific	1995	Deaths	5.250116	5.463492	5.06481	0.851327
High-income Asia Pacific	2000	Deaths	3.907267	4.066556	3.749344	0.866302
High-income Asia Pacific	2005	Deaths	2.79461	2.913861	2.671279	0.877038
High-income Asia Pacific	2010	Deaths	2.088176	2.184366	1.987694	0.886374
High-income Asia Pacific	2015	Deaths	1.755804	1.864571	1.653605	0.89541
High-income North America	1990	Deaths	0.7747455	0.8042565	0.7456377	0.868053
High-income North America	1995	Deaths	0.5872544	0.607993	0.5663421	0.878745
High-income North America	2000	Deaths	0.3797887	0.3938336	0.3661008	0.883605
High-income North America	2005	Deaths	0.2668204	0.2766664	0.2578901	0.887351
High-income North America	2010	Deaths	0.2093715	0.2171541	0.2016891	0.898744
High-income North America	2015	Deaths	0.1921448	0.2009709	0.1831634	0.904939
North Africa and Middle East	1990	Deaths	11.71415	15.23024	8.842072	0.49152
North Africa and Middle East	1995	Deaths	10.60152	14.46618	8.194139	0.546441

Region	Year	Measure name	Age-standardized rate	Age-standardized rate (upper UI)	Age-standardized rate (lower UI)	SDI
North Africa and Middle East	2000	Deaths	8.668694	11.95729	6.80521	0.588397
North Africa and Middle East	2005	Deaths	7.070056	9.60323	5.734939	0.625746
North Africa and Middle East	2010	Deaths	5.824667	7.775678	4.756512	0.654996
North Africa and Middle East	2015	Deaths	4.969915	6.751378	4.073385	0.685326
Oceania	1990	Deaths	23.02934	30.42657	14.14195	0.438439
Oceania	1995	Deaths	19.85829	26.79768	12.19227	0.463919
Oceania	2000	Deaths	16.74446	22.92432	10.58704	0.485921
Oceania	2005	Deaths	15.29719	21.61117	9.870066	0.507076
Oceania	2010	Deaths	13.49468	19.77937	8.630818	0.53028
Oceania	2015	Deaths	11.07693	16.69324	6.940259	0.554062
South Asia	1990	Deaths	119.8849	147.9572	98.95502	0.476193
South Asia	1995	Deaths	102.2134	123.5927	82.27892	0.512074
South Asia	2000	Deaths	84.88206	103.4164	69.57119	0.545809
South Asia	2005	Deaths	69.7662	85.7254	58.07809	0.580087
South Asia	2010	Deaths	55.37944	68.31906	44.40387	0.623476
South Asia	2015	Deaths	44.21226	54.01417	34.04633	0.662867
Southeast Asia	1990	Deaths	103.2428	131.1842	82.96793	0.569708
Southeast Asia	1995	Deaths	84.59319	108.0541	70.25538	0.6022
Southeast Asia	2000	Deaths	69.36918	88.72087	58.99017	0.631533
Southeast Asia	2005	Deaths	57.07634	73.3271	49.62849	0.657146
Southeast Asia	2010	Deaths	45.40519	58.08747	39.03452	0.682571
Southeast Asia	2015	Deaths	35.30699	46.53773	29.44505	0.710333
Southern Latin America	1990	Deaths	5.950096	6.207684	5.677673	0.686197
Southern Latin America	1995	Deaths	4.315772	4.497155	4.131996	0.712315
Southern Latin America	2000	Deaths	3.146322	3.281912	3.011304	0.741715
Southern Latin America	2005	Deaths	2.57956	2.698499	2.465368	0.761742
Southern Latin America	2010	Deaths	2.043674	2.150949	1.94551	0.779703
Southern Latin America	2015	Deaths	1.643693	1.789897	1.52577	0.799786

Region	Year	Measure name	Age-standardized rate	Age-standardized rate (upper UI)	Age-standardized rate (lower UI)	SDI
Southern Sub-Saharan Africa	1990	Deaths	94.33802	136.1191	75.66142	0.596739
Southern Sub-Saharan Africa	1995	Deaths	87.7499	126.1095	68.88481	0.635365
Southern Sub-Saharan Africa	2000	Deaths	100.5406	125.263	82.36133	0.664257
Southern Sub-Saharan Africa	2005	Deaths	98.94093	118.0077	71.1957	0.687727
Southern Sub-Saharan Africa	2010	Deaths	83.17465	99.1334	55.313	0.709034
Southern Sub-Saharan Africa	2015	Deaths	68.42937	83.53739	48.04899	0.725498
Tropical Latin America	1990	Deaths	8.373291	10.32205	6.266958	0.569258
Tropical Latin America	1995	Deaths	7.367558	8.261088	5.207918	0.617964
Tropical Latin America	2000	Deaths	6.251776	7.054482	4.454754	0.656385
Tropical Latin America	2005	Deaths	4.654014	5.568471	3.397116	0.687499
Tropical Latin America	2010	Deaths	3.552451	4.463313	2.481029	0.718198
Tropical Latin America	2015	Deaths	3.033846	3.911268	2.105511	0.745259
Western Europe	1990	Deaths	1.691211	1.750056	1.63696	0.821363
Western Europe	1995	Deaths	1.369054	1.41532	1.319519	0.840038
Western Europe	2000	Deaths	0.9786834	1.014148	0.9435137	0.853982
Western Europe	2005	Deaths	0.6728166	0.6977046	0.6494218	0.864865
Western Europe	2010	Deaths	0.5036525	0.5253921	0.484591	0.873457
Western Europe	2015	Deaths	0.4267431	0.4507562	0.4040926	0.883729
Western Sub-Saharan Africa	1990	Deaths	74.88242	106.3735	59.96101	0.340553
Western Sub-Saharan Africa	1995	Deaths	69.30402	96.83392	58.69847	0.369715
Western Sub-Saharan Africa	2000	Deaths	63.07497	90.20752	53.59168	0.392736
Western Sub-Saharan Africa	2005	Deaths	54.04531	76.22352	45.51217	0.414057
Western Sub-Saharan Africa	2010	Deaths	45.62548	66.38252	37.79426	0.436146
Western Sub-Saharan Africa	2015	Deaths	40.2643	60.61897	32.20811	0.476049
Andean Latin America	1990	Incidence	259.2294	279.4606	241.1221	0.601325
Andean Latin America	1995	Incidence	178.0467	192.9623	165.7883	0.639461
Andean Latin America	2000	Incidence	127.2961	136.6964	118.4	0.670974
Andean Latin America	2005	Incidence	91.31606	99.06165	84.1361	0.697096

Region	Year	Measure name	Age-standardized rate	Age-standardized rate (upper UI)	Age-standardized rate (lower UI)	SDI
Andean Latin America	2010	Incidence	75.87544	82.75185	68.99511	0.71593
Andean Latin America	2015	Incidence	72.73984	82.25491	65.08868	0.72763
Australasia	1990	Incidence	9.891301	11.99215	8.010864	0.840012
Australasia	1995	Incidence	8.307131	9.609564	6.98528	0.85437
Australasia	2000	Incidence	7.204093	8.204629	6.3124	0.868379
Australasia	2005	Incidence	7.336495	8.583173	6.183693	0.877539
Australasia	2010	Incidence	7.391374	8.85867	5.971319	0.883589
Australasia	2015	Incidence	7.548313	9.164231	6.084138	0.899057
Caribbean	1990	Incidence	57.28107	61.77377	53.56923	0.617814
Caribbean	1995	Incidence	51.0696	54.26877	48.14433	0.643936
Caribbean	2000	Incidence	42.63546	45.83552	40.02717	0.662542
Caribbean	2005	Incidence	37.37204	40.0534	34.87413	0.684099
Caribbean	2010	Incidence	34.2431	36.83928	31.81842	0.701189
Caribbean	2015	Incidence	34.63864	37.79518	31.62318	0.718367
Central Asia	1990	Incidence	133.3442	143.6137	123.4556	0.692146
Central Asia	1995	Incidence	142.0388	152.3795	132.8762	0.710885
Central Asia	2000	Incidence	135.3439	145.5954	126.8971	0.713923
Central Asia	2005	Incidence	135.5666	145.1741	126.8527	0.717763
Central Asia	2010	Incidence	104.9213	113.674	97.24607	0.73416
Central Asia	2015	Incidence	96.63054	105.3926	88.70994	0.75339
Central Europe	1990	Incidence	55.84333	59.58976	51.57142	0.790899
Central Europe	1995	Incidence	60.75566	64.19437	57.03596	0.813739
Central Europe	2000	Incidence	51.62664	55.16563	48.00411	0.830631
Central Europe	2005	Incidence	42.24123	45.23769	39.10761	0.847861
Central Europe	2010	Incidence	34.31984	36.92306	31.70568	0.864726
Central Europe	2015	Incidence	31.82835	34.43869	29.17114	0.877895
Central Latin America	1990	Incidence	67.3776	70.75385	62.42073	0.587256
Central Latin America	1995	Incidence	60.02903	64.43337	54.66381	0.626313

Region	Year	Measure name	Age-standardized rate	Age-standardized rate (upper UI)	Age-standardized rate (lower UI)	SDI
Central Latin America	2000	Incidence	45.6055	48.57533	42.27019	0.660288
Central Latin America	2005	Incidence	33.79435	36.15591	31.19308	0.69047
Central Latin America	2010	Incidence	27.50171	29.0609	25.7984	0.71747
Central Latin America	2015	Incidence	26.73988	28.60224	24.87491	0.750288
Central Sub-Saharan Africa	1990	Incidence	331.8451	369.6746	303.0722	0.374714
Central Sub-Saharan Africa	1995	Incidence	318.7483	353.7802	292.6418	0.388388
Central Sub-Saharan Africa	2000	Incidence	309.0733	339.5347	283.6701	0.397329
Central Sub-Saharan Africa	2005	Incidence	300.5246	331.9109	274.8132	0.408764
Central Sub-Saharan Africa	2010	Incidence	269.0284	297.6971	245.3517	0.429405
Central Sub-Saharan Africa	2015	Incidence	270.1681	300.426	241.5851	0.459795
East Asia	1990	Incidence	137.4133	161.2563	119.1104	0.633319
East Asia	1995	Incidence	123.31	143.1047	108.1087	0.666821
East Asia	2000	Incidence	118.6791	137.025	104.591	0.688894
East Asia	2005	Incidence	118.4773	131.5945	107.3066	0.71325
East Asia	2010	Incidence	96.01259	107.6627	86.28828	0.740004
East Asia	2015	Incidence	97.54379	110.7614	88.13359	0.763711
Eastern Europe	1990	Incidence	114.8245	123.0995	105.8022	0.803907
Eastern Europe	1995	Incidence	153.0282	166.0996	141.7361	0.821964
Eastern Europe	2000	Incidence	156.5982	170.2212	141.6483	0.822386
Eastern Europe	2005	Incidence	165.9071	176.167	152.6573	0.836392
Eastern Europe	2010	Incidence	136.0426	144.9263	125.8478	0.852063
Eastern Europe	2015	Incidence	116.9392	125.3031	106.4922	0.857602
Eastern Sub-Saharan Africa	1990	Incidence	250.0283	275.9663	229.7619	0.281544
Eastern Sub-Saharan Africa	1995	Incidence	241.3245	264.4172	224.5359	0.318378
Eastern Sub-Saharan Africa	2000	Incidence	238.4631	261.1331	222.2519	0.351583
Eastern Sub-Saharan Africa	2005	Incidence	215.6448	235.5818	201.1194	0.380305
Eastern Sub-Saharan Africa	2010	Incidence	191.5941	209.8603	178.6776	0.417935
Eastern Sub-Saharan Africa	2015	Incidence	186.5245	205.6725	171.5233	0.461506

Region	Year	Measure name	Age-standardized rate	Age-standardized rate (upper UI)	Age-standardized rate (lower UI)	SDI
Global	1990	Incidence	175.4216	196.7353	158.6584	0.600264
Global	1995	Incidence	161.1128	180.67	146.4408	0.627114
Global	2000	Incidence	153.3905	172.6043	139.3232	0.647289
Global	2005	Incidence	140.4923	155.7684	129.2215	0.667388
Global	2010	Incidence	125.3419	139.6078	114.4716	0.690595
Global	2015	Incidence	119.6398	134.0403	108.138	0.714202
High-income Asia Pacific	1990	Incidence	76.95872	83.53324	69.44547	0.831853
High-income Asia Pacific	1995	Incidence	53.30511	57.7201	48.39359	0.851327
High-income Asia Pacific	2000	Incidence	40.40483	44.46853	36.35609	0.866302
High-income Asia Pacific	2005	Incidence	35.87811	39.43592	32.31981	0.877038
High-income Asia Pacific	2010	Incidence	31.8028	34.39963	29.11024	0.886374
High-income Asia Pacific	2015	Incidence	31.22259	33.79673	28.67483	0.89541
High-income North America	1990	Incidence	10.79296	13.09793	8.553621	0.868053
High-income North America	1995	Incidence	6.447406	7.30672	5.612743	0.878745
High-income North America	2000	Incidence	5.003774	5.723757	4.298214	0.883605
High-income North America	2005	Incidence	4.687837	5.387771	3.985445	0.887351
High-income North America	2010	Incidence	3.638322	4.118258	3.179199	0.898744
High-income North America	2015	Incidence	3.824385	4.508888	3.187629	0.904939
North Africa and Middle East	1990	Incidence	62.88132	69.20137	57.07645	0.49152
North Africa and Middle East	1995	Incidence	54.42453	59.5713	49.68985	0.546441
North Africa and Middle East	2000	Incidence	46.53615	50.77685	42.46517	0.588397
North Africa and Middle East	2005	Incidence	40.55809	44.4038	36.89243	0.625746
North Africa and Middle East	2010	Incidence	36.6939	40.46652	33.34383	0.654996
North Africa and Middle East	2015	Incidence	36.73334	41.18154	32.70024	0.685326
Oceania	1990	Incidence	81.80069	90.27396	75.39917	0.438439
Oceania	1995	Incidence	77.92369	85.24641	71.79372	0.463919
Oceania	2000	Incidence	74.11682	81.80111	68.25436	0.485921
Oceania	2005	Incidence	66.69882	73.87242	61.36456	0.507076

Region	Year	Measure name	Age-standardized rate	Age-standardized rate (upper UI)	Age-standardized rate (lower UI)	SDI
Oceania	2010	Incidence	62.4368	68.60679	57.22836	0.53028
Oceania	2015	Incidence	67.43916	75.25967	60.98198	0.554062
South Asia	1990	Incidence	393.9562	451.1501	348.7045	0.476193
South Asia	1995	Incidence	351.0049	404.6818	310.4736	0.512074
South Asia	2000	Incidence	318.5684	368.2442	281.5246	0.545809
South Asia	2005	Incidence	263.6429	297.2838	235.2517	0.580087
South Asia	2010	Incidence	232.7328	261.5315	207.2396	0.623476
South Asia	2015	Incidence	204.4198	231.2269	181.1451	0.662867
Southeast Asia	1990	Incidence	385.0262	416.5152	358.9582	0.569708
Southeast Asia	1995	Incidence	319.4864	344.7288	298.1847	0.6022
Southeast Asia	2000	Incidence	291.1298	316.1901	272.0249	0.631533
Southeast Asia	2005	Incidence	249.7723	269.6911	232.7942	0.657146
Southeast Asia	2010	Incidence	220.6111	241.0015	206.0863	0.682571
Southeast Asia	2015	Incidence	208.7291	229.7336	192.8496	0.710333
Southern Latin America	1990	Incidence	48.35102	52.13338	44.27235	0.686197
Southern Latin America	1995	Incidence	38.72194	41.42699	35.28246	0.712315
Southern Latin America	2000	Incidence	34.95071	37.67014	32.35303	0.741715
Southern Latin America	2005	Incidence	27.36976	29.56432	25.26999	0.761742
Southern Latin America	2010	Incidence	24.7002	26.60832	22.78005	0.779703
Southern Latin America	2015	Incidence	23.50442	25.60374	21.37259	0.799786
Southern Sub-Saharan Africa	1990	Incidence	529.8512	608.3588	448.9415	0.596739
Southern Sub-Saharan Africa	1995	Incidence	551.2653	639.822	470.6725	0.635365
Southern Sub-Saharan Africa	2000	Incidence	678.6745	790.9196	588.3496	0.664257
Southern Sub-Saharan Africa	2005	Incidence	778.6144	913.6713	682.843	0.687727
Southern Sub-Saharan Africa	2010	Incidence	787.2392	919.1752	690.5623	0.709034
Southern Sub-Saharan Africa	2015	Incidence	724.6218	860.7267	621.4088	0.725498
Tropical Latin America	1990	Incidence	49.78968	57.81276	43.35041	0.569258
Tropical Latin America	1995	Incidence	46.14678	53.31418	40.3488	0.617964

Region	Year	Measure name	Age-standardized rate	Age-standardized rate (upper UI)	Age-standardized rate (lower UI)	SDI
Tropical Latin America	2000	Incidence	42.22425	49.08737	36.69271	0.656385
Tropical Latin America	2005	Incidence	41.14827	45.81338	36.33098	0.687499
Tropical Latin America	2010	Incidence	39.86771	41.7515	37.80948	0.718198
Tropical Latin America	2015	Incidence	35.03014	38.68136	30.93093	0.745259
Western Europe	1990	Incidence	25.93523	29.54231	22.71449	0.821363
Western Europe	1995	Incidence	19.41918	21.66106	17.42294	0.840038
Western Europe	2000	Incidence	14.67069	16.55044	12.92087	0.853982
Western Europe	2005	Incidence	12.32669	14.06659	10.77141	0.864865
Western Europe	2010	Incidence	10.4406	11.98379	8.927142	0.873457
Western Europe	2015	Incidence	10.61902	12.57972	8.759188	0.883729
Western Sub-Saharan Africa	1990	Incidence	187.9556	208.0269	173.8064	0.340553
Western Sub-Saharan Africa	1995	Incidence	177.5128	194.1947	164.6614	0.369715
Western Sub-Saharan Africa	2000	Incidence	170.0641	185.7849	157.6595	0.392736
Western Sub-Saharan Africa	2005	Incidence	158.379	173.0327	147.1326	0.414057
Western Sub-Saharan Africa	2010	Incidence	146.0309	160.3641	134.7667	0.436146
Western Sub-Saharan Africa	2015	Incidence	146.6701	162.7005	133.4244	0.476049
Andean Latin America	1990	Prevalence	172.9203	184.966	161.6407	0.601325
Andean Latin America	1995	Prevalence	117.4128	125.2576	110.2029	0.639461
Andean Latin America	2000	Prevalence	82.77686	88.34705	77.5192	0.670974
Andean Latin America	2005	Prevalence	58.76284	63.69952	54.27681	0.697096
Andean Latin America	2010	Prevalence	49.37034	53.87699	45.05118	0.71593
Andean Latin America	2015	Prevalence	47.95285	54.22791	42.73396	0.72763
Australasia	1990	Prevalence	4.898344	5.912738	3.885248	0.840012
Australasia	1995	Prevalence	4.129723	4.927222	3.424284	0.85437
Australasia	2000	Prevalence	3.575875	4.107608	3.081145	0.868379
Australasia	2005	Prevalence	3.641216	4.271626	3.015849	0.877539
Australasia	2010	Prevalence	3.706639	4.497083	2.958899	0.883589
Australasia	2015	Prevalence	3.802311	4.670052	3.020769	0.899057

Region	Year	Measure name	Age-standardized rate	Age-standardized rate (upper UI)	Age-standardized rate (lower UI)	SDI
Caribbean	1990	Prevalence	36.1638	38.44574	34.07988	0.617814
Caribbean	1995	Prevalence	31.77475	33.53046	30.10872	0.643936
Caribbean	2000	Prevalence	26.61617	28.25031	25.05506	0.662542
Caribbean	2005	Prevalence	23.47883	25.09092	21.89545	0.684099
Caribbean	2010	Prevalence	21.58023	23.12715	20.07885	0.701189
Caribbean	2015	Prevalence	21.82641	23.81129	19.85592	0.718367
Central Asia	1990	Prevalence	90.61837	96.84319	85.44526	0.692146
Central Asia	1995	Prevalence	106.9065	112.9908	101.3021	0.710885
Central Asia	2000	Prevalence	105.7566	112.2226	100.2559	0.713923
Central Asia	2005	Prevalence	98.31291	104.733	92.9463	0.717763
Central Asia	2010	Prevalence	80.13216	85.23547	75.05619	0.73416
Central Asia	2015	Prevalence	74.64805	81.23103	68.71758	0.75339
Central Europe	1990	Prevalence	27.78064	29.54008	26.00828	0.790899
Central Europe	1995	Prevalence	29.80622	31.43339	28.16103	0.813739
Central Europe	2000	Prevalence	26.03681	27.60109	24.50223	0.830631
Central Europe	2005	Prevalence	21.29889	22.71775	19.93959	0.847861
Central Europe	2010	Prevalence	17.4815	18.74768	16.25724	0.864726
Central Europe	2015	Prevalence	16.21988	17.56256	14.90462	0.877895
Central Latin America	1990	Prevalence	31.15122	32.9362	29.2935	0.587256
Central Latin America	1995	Prevalence	28.03602	30.12151	26.16323	0.626313
Central Latin America	2000	Prevalence	21.2154	22.57399	19.91727	0.660288
Central Latin America	2005	Prevalence	15.92988	17.03595	14.78995	0.69047
Central Latin America	2010	Prevalence	13.02035	13.82707	12.19107	0.71747
Central Latin America	2015	Prevalence	12.73324	13.67599	11.74048	0.750288
Central Sub-Saharan Africa	1990	Prevalence	291.1527	315.791	268.3526	0.374714
Central Sub-Saharan Africa	1995	Prevalence	273.7642	296.0363	253.0469	0.388388
Central Sub-Saharan Africa	2000	Prevalence	256.1596	278.7798	235.4036	0.397329
Central Sub-Saharan Africa	2005	Prevalence	239.1515	261.6869	218.0707	0.408764

Region	Year	Measure name	Age-standardized rate	Age-standardized rate (upper UI)	Age-standardized rate (lower UI)	SDI
Central Sub-Saharan Africa	2010	Prevalence	214.7525	234.5431	195.3621	0.429405
Central Sub-Saharan Africa	2015	Prevalence	219.0231	243.7254	194.9321	0.459795
East Asia	1990	Prevalence	155.7683	174.2929	138.501	0.633319
East Asia	1995	Prevalence	144.4398	158.4106	131.0818	0.666821
East Asia	2000	Prevalence	144.88	159.1071	130.7037	0.688894
East Asia	2005	Prevalence	129.9474	141.1815	119.1106	0.71325
East Asia	2010	Prevalence	124.1988	135.4612	112.8333	0.740004
East Asia	2015	Prevalence	123.5554	135.2937	112.4274	0.763711
Eastern Europe	1990	Prevalence	56.18814	59.58078	52.72581	0.803907
Eastern Europe	1995	Prevalence	76.65881	82.28704	72.07163	0.821964
Eastern Europe	2000	Prevalence	85.43962	91.60189	80.17805	0.822386
Eastern Europe	2005	Prevalence	94.26101	100.5325	88.58375	0.836392
Eastern Europe	2010	Prevalence	78.65072	83.86189	74.04079	0.852063
Eastern Europe	2015	Prevalence	63.54656	67.80974	59.36116	0.857602
Eastern Sub-Saharan Africa	1990	Prevalence	207.2653	224.0834	191.9857	0.281544
Eastern Sub-Saharan Africa	1995	Prevalence	198.2715	213.1737	184.7058	0.318378
Eastern Sub-Saharan Africa	2000	Prevalence	192.1825	205.8545	179.715	0.351583
Eastern Sub-Saharan Africa	2005	Prevalence	173.7006	185.7431	162.6214	0.380305
Eastern Sub-Saharan Africa	2010	Prevalence	157.5915	168.2428	148.0699	0.417935
Eastern Sub-Saharan Africa	2015	Prevalence	156.3925	168.9724	144.6342	0.461506
Global	1990	Prevalence	155.6543	170.8049	142.2851	0.600264
Global	1995	Prevalence	145.572	159.3543	133.8531	0.627114
Global	2000	Prevalence	141.5317	154.8688	129.7516	0.647289
Global	2005	Prevalence	129.6428	140.7603	119.5612	0.667388
Global	2010	Prevalence	123.1604	134.1084	113.5227	0.690595
Global	2015	Prevalence	120.323	131.5991	110.0002	0.714202
High-income Asia Pacific	1990	Prevalence	38.62818	41.51797	35.8963	0.831853
High-income Asia Pacific	1995	Prevalence	27.34416	29.61689	24.99053	0.851327

Region	Year	Measure name	Age-standardized rate	Age-standardized rate (upper UI)	Age-standardized rate (lower UI)	SDI
High-income Asia Pacific	2000	Prevalence	20.56504	22.61534	18.37646	0.866302
High-income Asia Pacific	2005	Prevalence	17.47615	19.24573	15.69199	0.877038
High-income Asia Pacific	2010	Prevalence	15.37219	16.69446	13.91245	0.886374
High-income Asia Pacific	2015	Prevalence	15.14696	16.55865	13.77238	0.89541
High-income North America	1990	Prevalence	5.499299	6.586417	4.404104	0.868053
High-income North America	1995	Prevalence	3.291302	3.732962	2.805992	0.878745
High-income North America	2000	Prevalence	2.547282	2.937061	2.143066	0.883605
High-income North America	2005	Prevalence	2.417279	2.822751	2.004627	0.887351
High-income North America	2010	Prevalence	1.893345	2.20504	1.610136	0.898744
High-income North America	2015	Prevalence	2.000257	2.385088	1.616336	0.904939
North Africa and Middle East	1990	Prevalence	45.70322	49.66086	41.95093	0.49152
North Africa and Middle East	1995	Prevalence	38.71048	42.04428	35.56811	0.546441
North Africa and Middle East	2000	Prevalence	32.92978	35.76895	30.19338	0.588397
North Africa and Middle East	2005	Prevalence	28.75238	31.24828	26.29384	0.625746
North Africa and Middle East	2010	Prevalence	26.12646	28.55511	23.75426	0.654996
North Africa and Middle East	2015	Prevalence	26.29818	29.40379	23.43584	0.685326
Oceania	1990	Prevalence	79.64565	85.61469	74.16448	0.438439
Oceania	1995	Prevalence	75.84589	81.50607	70.79701	0.463919
Oceania	2000	Prevalence	70.77253	75.90002	65.7298	0.485921
Oceania	2005	Prevalence	63.50135	68.47224	58.69209	0.507076
Oceania	2010	Prevalence	60.21642	65.19266	55.40218	0.53028
Oceania	2015	Prevalence	65.9924	72.65132	59.80518	0.554062
South Asia	1990	Prevalence	324.4472	368.2253	287.0908	0.476193
South Asia	1995	Prevalence	294.9989	333.5078	262.66	0.512074
South Asia	2000	Prevalence	276.5904	314.058	245.1999	0.545809
South Asia	2005	Prevalence	242.6637	270.6833	217.0052	0.580087
South Asia	2010	Prevalence	225.4191	251.5722	202.3049	0.623476
South Asia	2015	Prevalence	210.857	235.4756	188.2512	0.662867

Region	Year	Measure name	Age-standardized rate	Age-standardized rate (upper UI)	Age-standardized rate (lower UI)	SDI
Southeast Asia	1990	Prevalence	399.9079	427.1265	375.6205	0.569708
Southeast Asia	1995	Prevalence	342.2998	364.1629	321.6325	0.6022
Southeast Asia	2000	Prevalence	305.804	324.5271	288.2515	0.631533
Southeast Asia	2005	Prevalence	263.5542	279.3815	248.7266	0.657146
Southeast Asia	2010	Prevalence	241.2851	256.7055	227.4238	0.682571
Southeast Asia	2015	Prevalence	228.7166	245.6128	213.4247	0.710333
Southern Latin America	1990	Prevalence	23.54664	25.4144	21.74816	0.686197
Southern Latin America	1995	Prevalence	22.29495	23.73417	20.84438	0.712315
Southern Latin America	2000	Prevalence	20.88479	22.66362	19.30866	0.741715
Southern Latin America	2005	Prevalence	15.43906	16.56857	14.31069	0.761742
Southern Latin America	2010	Prevalence	13.78344	14.82549	12.70107	0.779703
Southern Latin America	2015	Prevalence	12.93989	14.07426	11.84859	0.799786
Southern Sub-Saharan Africa	1990	Prevalence	452.9378	517.4634	393.3497	0.596739
Southern Sub-Saharan Africa	1995	Prevalence	465.9857	533.3058	402.2654	0.635365
Southern Sub-Saharan Africa	2000	Prevalence	579.389	656.6725	504.9592	0.664257
Southern Sub-Saharan Africa	2005	Prevalence	661.1552	750.5378	582.8415	0.687727
Southern Sub-Saharan Africa	2010	Prevalence	664.2596	748.5427	592.1942	0.709034
Southern Sub-Saharan Africa	2015	Prevalence	630.6485	718.3207	547.4907	0.725498
Tropical Latin America	1990	Prevalence	35.67221	40.96439	30.33253	0.569258
Tropical Latin America	1995	Prevalence	33.20657	38.41043	28.19482	0.617964
Tropical Latin America	2000	Prevalence	30.51859	35.28632	25.98006	0.656385
Tropical Latin America	2005	Prevalence	28.91208	32.24088	25.24616	0.687499
Tropical Latin America	2010	Prevalence	27.97399	30.34158	25.85077	0.718198
Tropical Latin America	2015	Prevalence	25.06443	28.02801	21.91971	0.745259
Western Europe	1990	Prevalence	13.21068	14.83147	11.53078	0.821363
Western Europe	1995	Prevalence	10.66963	11.70494	9.5986	0.840038
Western Europe	2000	Prevalence	7.665274	8.627129	6.763092	0.853982
Western Europe	2005	Prevalence	6.209459	7.030667	5.358189	0.864865

Region	Year	Measure name	Age-standardized rate	Age-standardized rate (upper UI)	Age-standardized rate (lower UI)	SDI
Western Europe	2010	Prevalence	5.228542	6.005465	4.416921	0.873457
Western Europe	2015	Prevalence	5.347457	6.322089	4.360903	0.883729
Western Sub-Saharan Africa	1990	Prevalence	171.6217	185.8433	158.9221	0.340553
Western Sub-Saharan Africa	1995	Prevalence	161.3871	173.8667	150.1998	0.369715
Western Sub-Saharan Africa	2000	Prevalence	153.3446	164.5603	142.9263	0.392736
Western Sub-Saharan Africa	2005	Prevalence	143.2897	153.9964	133.284	0.414057
Western Sub-Saharan Africa	2010	Prevalence	133.1871	143.2885	123.7327	0.436146
Western Sub-Saharan Africa	2015	Prevalence	134.311	147.7847	122.3665	0.476049

Expected age-standardized rates per 100,000 population of tuberculosis incidence, prevalence, and mortality among HIV-negative individuals based on SDI

SDI	Expected mortality	Expected incidence	Expected prevalence
0.065	144.2257604	271.023483	234.8630379
0.075	140.4532044	265.8961366	229.8750418
0.085	136.7277294	260.8813831	225.0213752
0.095	133.0512455	255.9764701	220.2979705
0.105	129.4255423	251.1787184	215.7008934
0.115	125.8522875	246.4855195	211.2263379
0.125	122.3330264	241.894334	206.8706221
0.135	118.869182	237.4026895	202.6301841
0.145	115.4620549	233.0081787	198.5015777
0.155	112.1128247	228.7084576	194.4814684
0.165	108.822551	224.5012436	190.5666296
0.175	105.5921749	220.3843143	186.7539392
0.185	102.4225209	216.3555049	183.0403754
0.195	99.31429956	212.4127077	179.4230139
0.205	96.26810953	208.5538694	175.8990244
0.215	93.28444059	204.7769905	172.4656673
0.225	90.36367659	201.0801234	169.1202905
0.235	87.50609863	197.4613708	165.8603269
0.245	84.71188841	193.9188846	162.683291
0.255	81.98113179	190.4508646	159.5867764
0.265	79.31382235	187.0555569	156.5684532
0.275	76.70986519	183.7312526	153.6260652
0.285	74.1663304	180.4762871	150.7574278
0.295	71.67425853	177.289038	147.9604249
0.305	69.24626366	174.167925	145.2330075
0.315	66.88183123	171.1114076	142.5731907

SDI	Expected mortality	Expected incidence	Expected prevalence
0.325	64.58038274	168.117985	139.9790518
0.335	62.34127967	165.1861945	137.4487285
0.345	60.16382745	162.3146105	134.9804166
0.355	57.6560395	159.5018436	132.572368
0.365	54.8431225	156.7465397	130.2228889
0.375	52.1383914	154.0473787	127.9303384
0.385	49.54755266	151.4030739	125.6931259
0.395	47.0632039	148.8123711	123.5097105
0.405	44.6805903	146.2740473	121.3785982
0.415	42.39765806	143.7869105	119.2983415
0.425	39.92063468	140.9868516	116.8954674
0.435	37.53632495	136.4712806	112.732758
0.445	35.27944561	132.1111248	108.7283119
0.455	33.14287834	127.9007283	104.8758219
0.465	31.12056853	123.8346531	101.1692423
0.475	29.21059367	119.9076699	97.60277741
0.485	27.38610379	116.1147497	94.17087121
0.495	25.66509192	112.4510556	90.86819681
0.505	24.04385367	108.9119348	87.68964677
0.515	22.51750599	105.4929116	84.63032373
0.525	21.08151233	102.1896797	81.68553144
0.535	19.73141434	98.99809586	78.85076621
0.545	18.46239903	95.91417297	76.12170877
0.555	17.27019692	92.93407409	73.49421639
0.565	16.15069928	89.36159355	70.04503317
0.575	15.09995884	85.07750248	65.64516293
0.585	14.11418966	81.00363285	61.52587605
0.595	12.96391245	77.12945081	57.66902412
0.605	11.8322551	73.44496073	54.05764843
0.615	10.79071346	69.94067721	50.67590095

SDI	Expected mortality	Expected incidence	Expected prevalence
0.625	9.833554958	66.60759855	47.50897064
0.635	8.955165184	63.43718154	44.54301478
0.645	8.150094804	60.42131775	41.76509478
0.655	7.413095371	57.55231094	39.1631164
0.665	6.738230926	54.8228557	36.72577394
0.675	6.12169568	52.22601724	34.44249813
0.685	5.559086407	49.75521225	32.30340759
0.695	5.046127827	46.69690371	30.07735201
0.705	4.578807409	43.81503744	28.00604542
0.715	4.197174594	41.12526054	26.08653832
0.725	3.91033179	38.6141595	24.30723257
0.735	3.64480549	36.26929035	22.65744418
0.745	3.399132632	34.07910661	21.12732944
0.755	3.171732049	32.03289274	19.70781714
0.765	2.96095672	30.12070267	18.39054642
0.775	2.765511733	28.33330293	17.16780967
0.785	2.518639731	26.66212011	16.03250015
0.795	2.258446258	25.09919229	14.97806388
0.805	2.02734896	23.63712411	13.9984555
0.815	1.82214373	22.26904525	13.08809768
0.825	1.64000532	20.98857199	12.24184392
0.835	1.478323568	19.78977168	11.45494428
0.845	1.323291839	18.66712989	10.72301396
0.855	1.164631997	17.61551996	10.04200445
0.865	1.02537168	16.63017484	9.408176911
0.875	0.903148497	15.70666108	8.818077844
0.885	0.79588117	14.84085469	8.268516614
0.895	0.701753786	14.02891882	7.756544838
0.905	0.619145005	13.26728307	7.279437419
0.915	0.546628309	12.55262438	6.834675102

SDI	Expected mortality	Expected incidence	Expected prevalence
0.925	0.482950562	11.88184928	6.419928422
0.935	0.42701239	11.25207748	6.033042948
0.945	0.377850382	10.66062663	5.672025692
0.955	0.334621027	10.10499827	5.335032607
0.965	0.296586332	9.582864711	5.020357084
0.975	0.263100968	9.092056965	4.726419348
